# Dihydroquinolines, Dihydronaphthyridines and Quinolones by Domino Reactions of Morita-Baylis-Hillman Acetates

**DOI:** 10.3390/molecules26040890

**Published:** 2021-02-08

**Authors:** Joel K. Annor-Gyamfi, Ebenezer Ametsetor, Kevin Meraz, Richard A. Bunce

**Affiliations:** Department of Chemistry, Oklahoma State University, Stillwater, OK 74078-3071, USA; jannorg@okstate.edu (J.K.A.-G.); eametse@okstate.edu (E.A.); meraz.kevin@outlook.com (K.M.)

**Keywords:** Morita-Baylis-Hillman acetates, primary amines, domino reactions, dihydroquinolines, dihydronaphthyridines, quinolones

## Abstract

An efficient synthetic route to highly substituted dihydroquinolines and dihydronaphthyridines has been developed using a domino reaction of Morita-Baylis-Hillman (MBH) acetates with primary aliphatic and aromatic amines in DMF at 50–90 °C. The MBH substrates incorporate a side chain acetate positioned adjacent to an acrylate or acrylonitrile aza-Michael acceptor as well as an aromatic ring activated toward S_N_Ar ring closure. A control experiment established that the initial reaction was an S_N_2′-type displacement of the side chain acetate by the amine to generate the alkene product with the added nitrogen nucleophile positioned trans to the S_N_Ar aromatic ring acceptor. Thus, equilibration of the initial alkene geometry is required prior to cyclization. A further double bond migration was observed for several reactions targeting dihydronaphthyridines from substrates with a side chain acrylonitrile moiety. MBH acetates incorporating a 2,5-difluorophenyl moiety were found to have dual reactivity in these annulations. In the absence of O_2_, the expected dihydroquinolines were formed, while in the presence of O_2_, quinolones were produced. All of the products were isolated in good to excellent yields (72–93%). Numerous cases (42) are reported, and mechanisms are discussed.

## 1. Introduction

We recently disclosed a synthesis of 1,3,6-trisubstituted naphthalenes and 6,8-disubstituted quinolines from Morita-Baylis-Hillman (MBH) acetates and active methylene compounds (AMCs) in DMF with K_2_CO_3_ at 23–90 °C [1]. This process formally involved S_N_2′ displacement of the acetoxy group by the stabilized AMC anion, a second deprotonation, S_N_Ar cyclization and elimination to give the aromatic product. However, contrary to previous reports, our experiments indicated that the initial adduct was formed with the added nucleophile trans to the S_N_Ar acceptor ring, necessitating double bond equilibration before cyclization. The current project sought to develop an efficient route to dihydroquinolines and dihydronaphthyridines from MBH acetates. This work differs from our earlier contribution [1] in providing a route to the title heterocycles wherein a dihydropyridine is fused to a preexisting ring and cannot aromatize. The sequence was initiated by addition of a nitrogen rather than a carbon nucleophile and required mild heating, but no added base. Despite the more active nucleophile, the reaction followed a mechanism similar to our previous report. An interesting double bond migration was observed in several naphthyridines generated from acrylonitrile-derived precursors. Finally, in addition to yielding dihydroquinolines under anaerobic conditions, a difluoro-substituted MBH substrate showed disparate reactivity in generating a quinolone when reacted in the presence of O_2_. These findings complement earlier cyclizations by our group to form 1-alkyl-2,3-dihydro-4(1*H*)-quinolinones [2] and 1-alkyl-2,3-dihydro-1,8-naphthyridine-4(1*H*)-ones [3] by a Michael-S_N_Ar process.

MBH adducts hold great potential in drug synthesis [4,5] due to their high functional density, which makes them prime candidates for domino reactions to prepare a wide range of cyclic targets. 1,2-Dihydroquinolines exhibit valuable biological activity against a wide range of human and animal health disorders [6,7,8,9]. The corresponding 1,2-dihydro-1,8-naphthyridine derivatives appear to have fewer current uses [10,11], but this may stem from a lack of procedures to generate a diverse selection of these targets. Fluoroquinolones were first introduced as antibiotics in the early 1960s and many derivatives have been reported [12,13]. Although bacteria have developed a resistance to some derivatives [12,13], there are many analogs that remain in use to treat various illnesses [14,15,16,17]. Addition of primary amines to MBH acetates permits rapid assembly of each of these privileged structures with a minimum of purifications.

**Figure 1:**
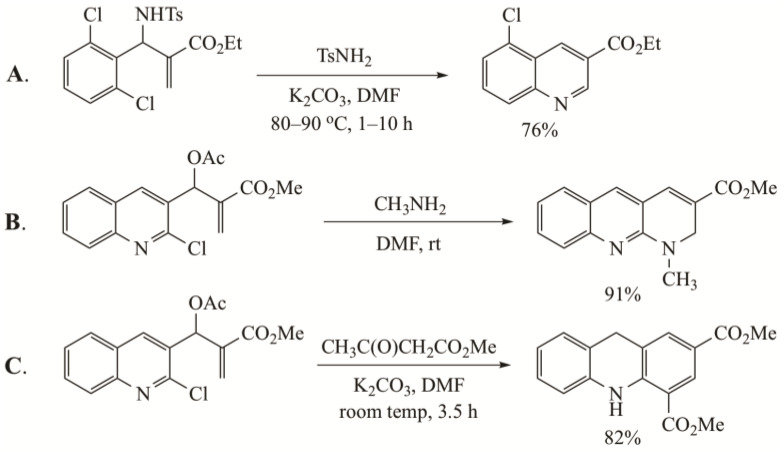

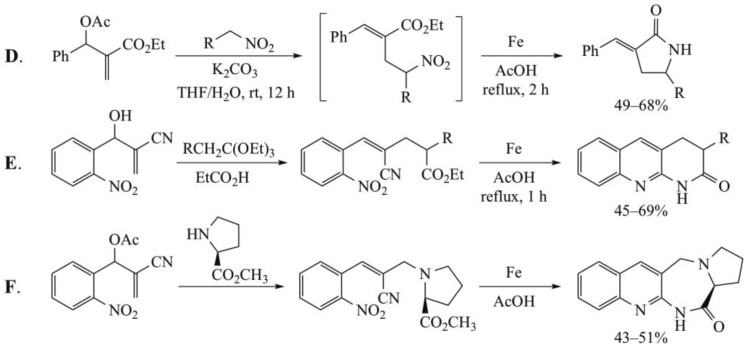
Some previous nitrogen heterocycle syntheses from MBH precursors. (**A**) Formation of quinolines; (**B**) formation of *N*-substituted-1,2-dihydrobenzo[*b*][1,8]-naphthyridines; (**C**) formation of dihydroacridines; (**D**) formation of γ-lactams; (**E**) formation of 3,4-dihydrobenzo[*b*][1,8]naphthyridin-2(1*H*)-ones; (**F**) formation of fused polycyclic quinolines.

Previous work has described a number of syntheses of nitrogen heterocycles from MBH precursors. For example, a synthesis of 3-quinolinecarboxylic esters has been reported from ethyl 2-((2-chlorophenyl)((tosylsulfonamido)methyl)acrylate and catalytic tosylsulfonamide [18] (Figure 1A). A second study described an approach to *N*-substituted-1,2-dihydrobenzo[*b*][1,8]-naphthyridines by reaction of MBH acetates derived from 2-chloroquinoline-3-carboxaldehyde with primary amines [19] (Figure 1B). A later account detailed a synthesis of dihydroacridines from this same MBH precursor with AMCs [20] (Figure 1C). Basavaiah and co-workers reacted nitroalkane anions, from nitroalkanes and K_2_CO_3_, with MBH acetates to give adducts that were subjected to a reduction-cyclization sequence to afford γ-lactams [21] (Figure 1D). In a slightly different application, this same group converted MBH alcohols to 3,4-dihydrobenzo[*b*][1,8]naphthyridin-2(1*H*)-ones by a Johnson–Claisen rearrangement with triethyl orthoesters to give ethyl (*Z*)-4-cyano-5-(2-nitrophenyl)-4-pentenoate derivatives which were converted to the heterocycles using a domino cyclization initiated by reduction of the nitro function [22] (Figure 1E). Finally, an approach to fused polycyclic quinolines starting from MBH acetates and methyl prolinate or imidazole-2-carboxaldehyde has also appeared [23] (Figure 1F). Several of the cited papers assumed the initial S_N_2′ reaction resulted in the correct alkene geometry for cyclization [18,21,22]. Only a few noted that the initial addition–elimination process afforded a *Z* alkene (or a mixture of *Z* and *E*) which lacked the geometry needed for direct cyclization [19,20]. In the current study, MBH acetates were reacted with primary amines to produce 3,6-disubstituted 1-alkyl/phenyl-1,2-dihydroquinolines and 3-substituted 1-alkyl/phenyl-1,2-dihydro-1,8-naphthyridines in good to excellent yields. In our work, only the Z alkene was observed, and thus would require a *Z*→*E* alkene equilibration prior to cyclization.

## 2. Results and Discussion

**Figure 2:**
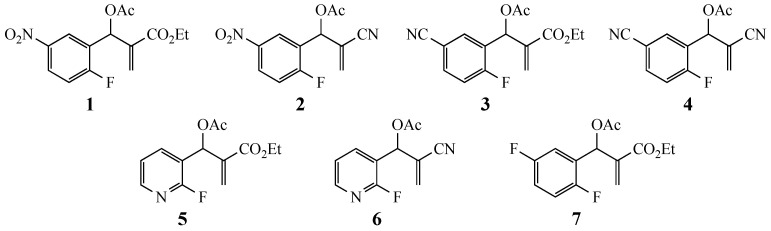
Substrates for the preparation of 3,6-disubstituted 1-alkyl/phenyl-1,2-dihydroquinolines (**1**–**4** and **7**), 3-substituted 1-alkyl/phenyl-1,2-dihydro-1,8-naphthyridines (**5**,**6**) and quinolones (**7**).

Our study focused on MBH acetates **1**–**7**, four with 2-fluoroaromatic rings activated toward S_N_Ar ring closure by C5 nitro or cyano groups, and two with a 2-fluoropyridine group and one with a 2,5-difluoroaromatic ring (Figure 2). The Michael acceptors embedded in the side chains were either acrylate esters or acrylonitriles. These precursors were prepared by a Morita-Baylis-Hillman reaction using 1 equiv of the aromatic aldehyde, 2 equiv of the activated alkene and 1.2 equiv of 1,4-diazabicyclo[2.2.2]octane (DABCO) [1] in acetonitrile (2 days, 23 °C) to afford 86–98% yields. Acylation of the alcohol adducts with no allylic inversion was achieved in ≥95% yield after 30 min at 0 °C by treatment with acetic anhydride and catalytic trimethylsilyl trifluoromethanesulfonate, as has been previously described [1,24]. These substrates required no purification and were used directly in the annulation procedure. 

**Figure 3:**
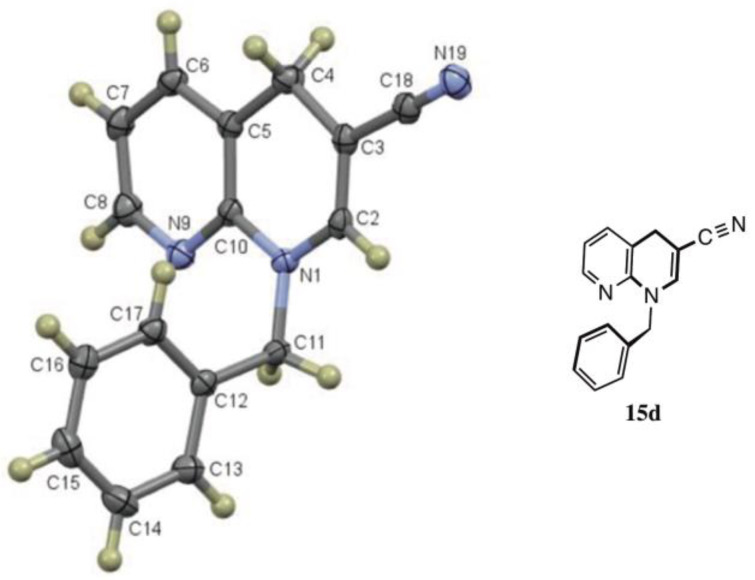
X-ray structure of 1-benzyl-1,4-dihydro-1,8-naphthyridine-3-carbonitrile (**15d**, CCDC 2035027).

The results from reactions of **1**–**4** (1 equiv) with primary aliphatic or aromatic amines **8** (1.5 equiv) in DMF (1 h, 23 °C, and 1–5 h, 50 °C) gave the 3,6-disubstituted 1-alkyl/phenyl-1,2-dihydroquinolines **9**–**12** shown in Table 1. A similar protocol was used to generate dihydronaphthyridines **13** and **14** from precursors **5** and **6**, respectively (Table 2). Finally, the difluoro-substituted substrate **7** showed dual reactivity under anaerobic versus aerobic conditions, giving dihydroquinolines **18** in the absence of O_2_ and quinolones **19** when O_2_ was present (Table 3). As in our previous work [1], it was also noted that the 2-fluoropyridine (and 2,5-difluorophenyl) precursors were less reactive, requiring higher temperatures and longer reaction times (1 h, 23 °C and 5–6 h, 90 °C) for complete conversion. This appears to reflect the decreased activation of these acceptor rings in the final S_N_Ar ring-closing step. Furthermore, while the 2-fluoropyridine-bound acrylate **5** successfully delivered dihydronaphthyridines **13**, the corresponding acrylonitrile **6** often yielded products **14** contaminated by uncyclized material. Although acrylates and acrylonitriles are among the most reactive acceptors in aza-Michael reactions, alkyl substitution α to the electron-withdrawing group (EWG) on these substrates slows the addition, and this may impact their relative reactivities toward nucleophiles [25]. Additionally, reactions of acetates **6d** and **6f** also yielded double bond-migrated products **15d** and **15f**, respectively (Figure 3). Finally, discussion of the unique reactivity of difluorophenyl substrate **7** is provided at the end of the article.

The process is very efficient, requiring only one step from the MBH acetates and one column chromatography (SiO_2_, 10% EtOAc in hexanes) for each final product. The heterocycles were formed in good to excellent yields (72–93% for all substrates except **6**, see below) and were fully characterized by analytical and spectroscopic methods. The IR spectra indicated the presence of the expected functional groups (conjugated CO_2_Et or CN, polarized conjugated double bonds and NO_2_). The ^1^H NMR of the dihydroquinolines showed a doublet (*J_1,3_* < 3.0 Hz), a doublet of doublets (*J_1,2 and 1,3_* = large and small) and a doublet (*J_1,2_* > 9.0 Hz), respectively, for protons on C5, C7 and C8 with chemical shifts in accord with those expected for protons adjacent to electron-withdrawing or donating functionality. The dihydropyridine subunit of these products showed a broad singlet at δ 7.0–7.5 for the C4 alkene proton and a small doublet (*J_allylic_* < 2 Hz) at δ 4.5–5.0 for the C2 ring methylene. The ^1^H NMR spectra of the dihydronaphthyridines showed the expected chemical shifts and coupling patterns for the protons at C5, C6 and C7 of the system, while the protons at C4 and C2 of these derivatives were similar to those of the dihydroquinolines. For all products, the ethyl ester, if present, was also clearly discernible as were proton signals for the added amine fragment. The ^13^C NMR spectra, for all derivatives, were appropriate with respect to the number of carbonyl, nitrile, aromatic and aliphatic carbons. The mass spectra showed an ion corresponding to the molecular weight of the compound and the elemental analyses confirmed the formulas and purity.

An interesting observation was noted in cyclizations of substrate **6**, which consisted of a 2-fluoropyridine having an acrylonitrile moiety in the side chain. These reactions required heating for 6 h at 90 °C to assure complete conversion. While *n*-hexylamine and phenethylamine gave normal 1,2-dihydronaphthyridine products **14b** (82%) and **14e** (80%), respectively, benzylamine and aniline gave 1,4-dihydronaphthyridines **15d** (82%) and **15f** (72%) as the major products, resulting from migration of the double bond from C3-C4 to C2-C3. X-ray analysis confirmed the structure of double bond-migrated product **15d** from benzylamine (**8d**) with **6** (see Figure 3 and the SI). The ^13^C NMR of these systems was distinguished by the presence of the β-enamine carbon bearing the CN group (C3) which appeared at δ 75–80 in the spectrum. A reason for this isomerization is not clear since all of the reactions were performed under the same conditions. However, there is stabilization available to the enaminonitrile, as the dihydropyridine π system is still conjugated with the fused pyridine ring, although the required resonance contributor places a positive charge on an electronegative nitrogen.

**Figure 4:**
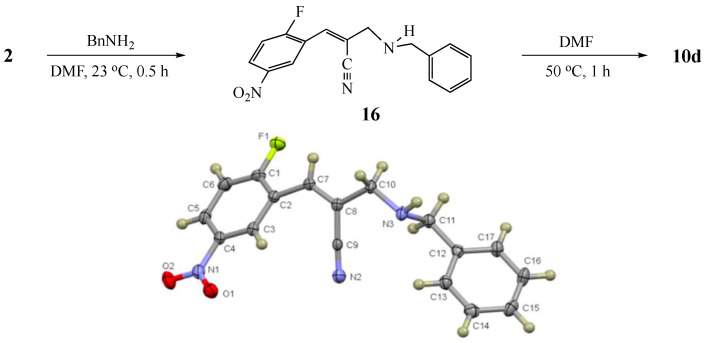
Control reaction for selective conjugate addition and formation of (*Z*)-2-((benzylamino)methyl)-3-(2-fluoro-5-nitrophenyl)acrylonitrile (**16**, CCDC 2035028) and its further conversion to **10d**.

Two basic mechanisms are possible for this transformation: (1) initial S_N_Ar attack of the amino group at the aromatic ring, followed by conjugate addition to the side chain double bond with elimination of acetoxy (OAc) or (2) initial S_N_2′-type displacement of OAc from the side chain, followed by an S_N_Ar cyclization. Option 1 would unequivocally predict the observed product of the reaction and would not be dependent on alkene geometry in the intermediate for ring closure. This was ruled out for the dihydronaphthyridines by a control experiment wherein benzylamine (**8d**) was stirred with 2-fluoropyridine for 12 h at 50 °C to afford none of the S_N_Ar product. In the dihydroquinoline series, substitution by **8d** was noted after 5 h at 50 °C for acceptor rings activated by a C4 NO_2_ (complete conversion) and CN (modest conversion), but evidently this was slower than addition to the side chain aza-Michael acceptors (see below). Option 2 would require a diastereoselective addition to give the *E* alkene that positions the amine nitrogen cis to the S_N_Ar acceptor ring. To examine this aspect of the reaction, the adduct from addition of **8d** to the acrylonitrile moiety of **2** was intercepted after 30 min at 23 °C. Although our earlier paper utilized a similar control to probe the reaction chronology for a carbon nucleophile, this same experiment was necessary in the current study since nitrogen, which is generally more reactive in the S_N_Ar reaction, could potentially attack the aryl ring first and result in a different sequence of events. From this control reaction, intermediate **16** was isolated in 76% yield and was accompanied by 11% of the ring closed dihydroquinoline product **10d**. X-ray analysis of intermediate **16** revealed it to be (*Z*)-2-((benzyl-amino)methyl)-3-(2-fluoro-5-nitrophenyl)acrylonitrile having the amine nitrogen trans to the S_N_Ar acceptor ring (see Figure 4 and the SI). Thus, Option 2 above likely predominates, followed by double bond equilibration to permit the S_N_Ar ring closure. No other intermediates were observed as the reaction proceeded. Presumably, any *E* alkene produced in the reaction rapidly cyclized and eluded detection by standard methods used to monitor reactions. Further evidence for double bond equilibration was established by heating of **16** in DMF for 1 h at 50 °C, which afforded **10d** in 60% yield (unoptimized).

**Scheme 1:**
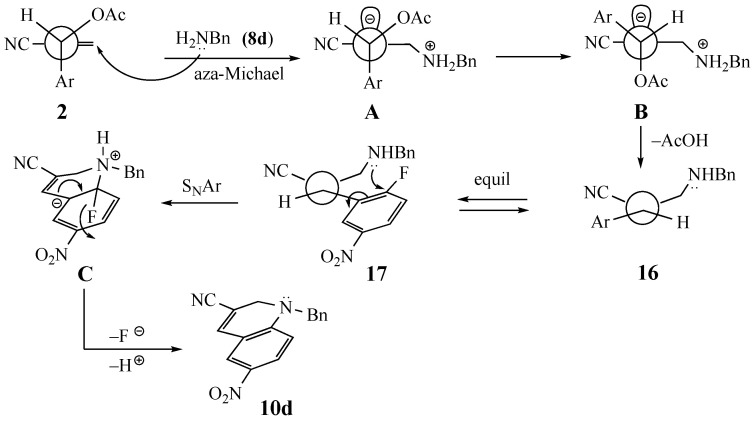
Plausible mechanism for the formation of dihydroquinoline **10d** from MBH acetate **2** and amine **8d**.

In reactions of the side chain allylic acetate, there are two mechanistic scenarios to explain the addition of amines to MBH acetates. The first involves the S_N_2′ process shown in Scheme 1 for the addition of benzylamine (**8d**) to MBH acetate **2** to give **10d**. The initial conformation of **2** would allow minimal steric interaction between the aromatic ring, the OAc substituent and the EWG [26]. While the OAc impedes approach to the top face of the Michael acceptor, the planar aryl group should not significantly hinder approach to the bottom face of this moiety. Addition of **8d** to the bottom face of the molecule would result in a build-up of negative charge on the top face of the allylic system as shown in **A**, leading to elimination of acetic acid and formation of the *Z* alkene **16** [27]. Elimination of acetic acid would require a 120° rotation to bring the OAc anti to the site of negative charge. This rotation should also move the large 2-fluoro-5-nitrophenyl S_N_Ar acceptor away from the newly added nucleophile. Such a rotation would be relatively easy when EWG is a sterically small group such as CN, but could be more difficult when EWG is a larger CO_2_Et. Equilibration of **16** to **17** [28,29,30,31,32] and attack of the side chain nitrogen at the fluorine-bearing aromatic carbon would give Meisenheimer intermediate **C**. Rearomatization via loss of fluoride and deprotonation of the ammonium nitrogen would lead to dihydroquinoline **10d**. A similar process with the fluoropyridine precursors could be invoked for the formation of dihydronaphthyridines.

**Scheme 2:**
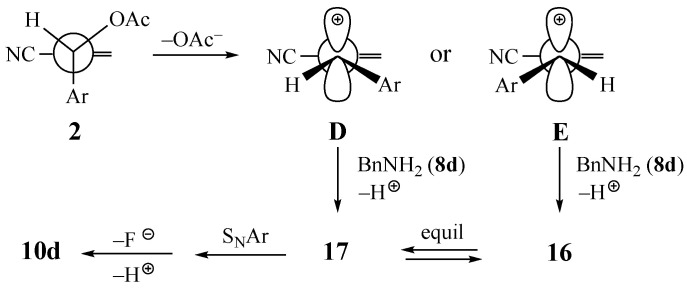
Possible carbocation mechanism for the formation of dihydroquinoline **10d**.

An alternative mechanism proposed for S_N_2′ reactions must also be considered [33]. According to this hypothesis, the substrate could lose the OAc group to give the aryl allyl stabilized carbocations **D** and **E** (Scheme 2), which should then add the amine nucleophiles to the least hindered terminal carbon of the allylic system. While steric congestion around the alkene terminus is lower in carbocation **E**, it is difficult to envision any selectivity in producing only the *Z* alkene **16** with the added amine trans to the S_N_Ar acceptor. In our studies, here and elsewhere [1], a product having the added nucleophile cis to the acceptor ring was not observed. This could potentially rule out this mechanistic option, unless the cyclization of **17** is sufficiently fast that it is not possible to observe or isolate this intermediate.

**Scheme 3:**
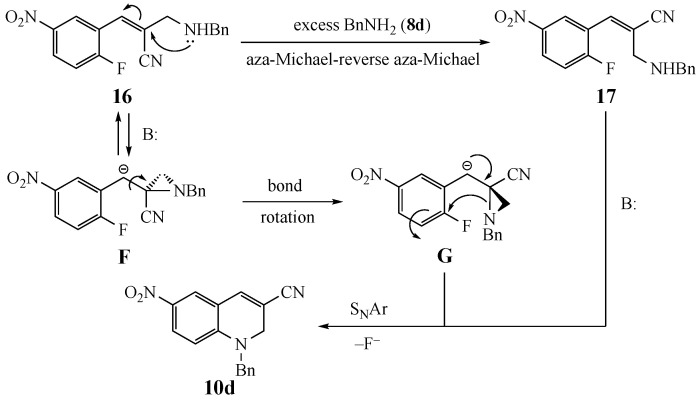
Plausible mechanisms for double bond equilibration.

Two possible pathways for the equilibration of **16** to **17** required prior to S_N_Ar cyclization are depicted in Scheme 3. The first, and most likely, is a well-precedented aza-Michael–reverse aza-Michael sequence [28,29,30,31,32] involving the excess amine in the reaction. A second option is analogous to an intramolecular addition–elimination process previously described [1]. This alternative mechanism would require addition of the amine nitrogen in **16** to the aryl-conjugated double bond to give the stabilized aziridinyl benzylic anion **F**. This scenario is viable only if the aromatic ring is sufficiently electron-deficient to stabilize the anionic center. Bond rotation to give **G** and ring opening would then deliver the *E* alkene needed for cyclization. Once equilibration occurs, ring closure and deprotonation to furnish **10d** should be facile. Even if the equilibrium does not strongly favor the necessary alkene geometry, product formation should gradually funnel the initial adduct over to the fused heterocyclic target.

In the current reaction, dihydropyridine annulation would require S_N_Ar ring closure from the *E* alkene (aryl and added amine cis). An alternative progression where the S_N_Ar occurred first would preclude the necessity for stereoselective formation of an *E* alkene for cyclization but evidence for this pathway was lacking. While several previous studies assumed the formation of the *E* alkene intermediate with the added amine proximal to the S_N_Ar acceptor ring, our experiments permitted isolation of only the *Z* alkene, and this appears to be the major pathway. If any of the *E* alkene intermediate was formed, it likely underwent rapid ring closure. Thus, an equilibration of the alkene geometry was essential to provide high yields of cyclized products. This equilibration is evidently facile as further treatment of isolated intermediate **16** in DMF for 1 h at 50 °C afforded dihydroquinoline **10d**.

**Figure 5:**
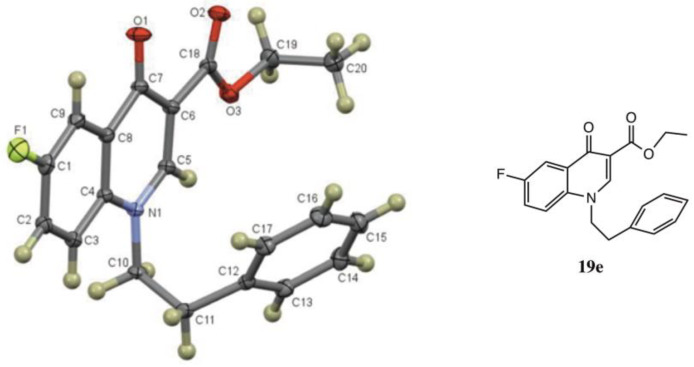
ORTEP drawing of quinolone **19e** (CCDC 2057847).

We were also interested in extending this reaction to other substrates with activating functionality. Treatment of ethyl 2-(acetoxy(2,5-difluorophenyl)methyl)acrylate (**7**) with amines **8** in the same molar ratio afforded ethyl 1-alkyl-6-fluoro-1,2-dihydroquinoline-3-carboxylates **18** (Table 3). These reactions also required heating at 90 °C to proceed to completion. An interesting discovery was made, however, when the amine reaction was run using an older bottle of anhydrous DMF. In this experiment, ethyl 1-alkyl-6-fluoro-4-oxo-1,4-dihydroquinoline-3-carboxylates (e.g., fluoroquinolones **19**) were isolated rather than the expected dihydroquinolines (Table 3). It was rationalized that this could be due to the presence of dissolved O_2_ in the older bottle of solvent. It is known that O_2_ readily dissolves in DMF up to a concentration of *ca* 4.5 mM at 25 °C [34]. Indeed, when O_2_ was bubbled through DMF from a fresh bottle of anhydrous DMF for 15 s using a gas dispersion tube, the reaction produced the quinolone as the only product. On the small scale studied, the dissolved O_2_ was sufficient for complete conversion, and an O_2_ atmosphere was not needed. This competing reaction is likely facilitated by the higher temperatures necessary to drive the ring closure. Spectroscopically, the ^1^H NMR spectra of the fluorodihydroquinolines displayed the typical signals with additional line broadening or coupling by the fluorine. The fluoroquinolones exhibited a singlet at δ 7.4 for the quinolone β-enone proton as well as a singlet at δ 8.0–8.5, a doublet of doublets at δ 8.3 (*J* = *ca* 9.0, 3.0 Hz), and a doublet at δ 7.4 (*J* = *ca* 5–6 Hz when R *=* CH_3_ or *i*-Bu; this signal was obscured when R *=* CH_2_Ph or CH_2_CH_2_Ph) for protons at C5, C7 and C8, respectively. Additionally, the C2 ring methylene, observed for the dihydroquinolines, was absent. The ^13^C NMR spectra, showed the correct number and types of carbons with added coupling in the fluoroaromatic rings. IR and mass spectra were consistent with the structures indicated. Proof of the quinolone structures was obtained by X-ray analysis of **19e**, derived from reaction of **7** with phenethylamine (**8e***,* See Figure 5s and SI).

**Scheme 4:**
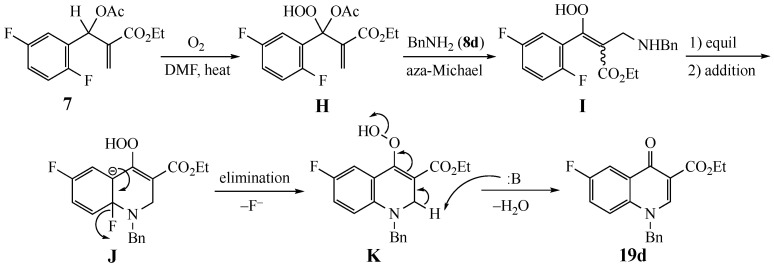
Possible mechanism for the conversion of difluoro substrate **7** to fluoroquinolone **19d**.

The oxidation process observed for the difluoro substrate **7** has no precedent under the current conditions and we have proposed a possible mechanism in Scheme 4 for its reaction with benzylamine (**8d**) to give **19d**. The MBH acetate has a very labile allylic-benzylic C–H bond between the side chain Michael acceptor and the aromatic ring. Insertion of O_2_ into this bond would generate an intermediate hydroperoxide **H** as the initial intermediate. Addition of the amine to the side chain acrylate with loss of the OAc would then give the unsaturated MBH hydroperoxide **I**. S_N_Ar ring closure (via direct addition or equilibration-addition) to Meisenheimer intermediate **J** and fluoride elimination would afford hydroperoxide **K**. Final elimination of water from this intermediate, promoted by excess amine, would then provide quinolone **19d**.

This novel oxidation extends the possible uses of MBH acetates to access heterocycles with potential medicinal value. The difluorinated acetate **7** with less S_N_Ar activation required higher temperatures for reaction and this led to a new reaction pathway when O_2_ was present. More reactive substrates **1**–**6** were converted to dihydroquinolines at lower temperature and did not undergo competitive oxidation to the quinolones. Attempts to induce this oxidation in **1**–**6** using O_2_-saturated DMF at 90 °C failed, and only dihydroquinolines were isolated, albeit in lower yields. The current reaction represents a new approach to fluoroquinolones, which may be further developed to produce previously inaccessible analogs.

## 3. Materials and Methods

### 3.1. General Methods

Unless otherwise indicated, all reactions were carried out under dry N_2_ in oven-dried glassware. All reagents and solvents were used as received. Reactions were monitored by thin layer chromatography on silica gel GF plates (Analtech No 21521, Newark, DE, USA). Preparative separations were performed by flash chromatography on silica gel (Davisil^®^, grade 62, 60–200 mesh, Sorbent Technologies, Norcross, GA, USA) containing UV-active phosphor (Sorbent Technologies No. UV-05) slurry packed into quartz columns. Band elution for all chromatographic separations was monitored using a hand-held UV lamp (Fisher Scientific, Pittsburgh, PA, USA). Melting points were obtained using a MEL-TEMP apparatus (Cambridge, MA, USA) and are uncorrected. FT-IR spectra were run using a Varian Scimitar FTS 800 spectrophotometer (Randolph, MA, USA) as thin films on NaCl disks. ^1^H- and ^13^C-NMR spectra were measured using a Bruker Avance 400 system (Billerica, MA, USA) in the indicated solvents at 400 MHz and 101 MHz, respectively, with (CH_3_)_4_Si as the internal standard; coupling constants (*J*) are given in Hz. Low-resolution mass spectra were obtained using a Hewlett-Packard Model 1800A GCD GC–MS system (Palo Alto, CA, USA). Details of the X-ray structure determinations are given in the SI. Elemental analyses (±0.4%) were determined by Atlantic Microlabs (Norcross, GA, USA).

### 3.2. Syntheses of the MBH Acetates

The syntheses of MBH acetates **1**–**5** have been previously described [1].

#### 3.2.1. 2-Cyano-1-(2-fluoropyridin-3-yl)allyl Acetate (6)

Compound **6** was prepared from the MBH alcohol according to the procedure given for the preparation of compound **5 [1]** and did not require any further purification. Yield: 209 mg (0.95 mmol, 95%) as a colorless oil; IR: 2231, 1756, 1609 cm^−1^; ^1^H NMR (400 MHz, CDCl_3_): δ 8.25 (apparent s, 1H), 7.99 (apparent t, *J* = 8.5 Hz, 1H), 7.30 (m, 1H), 6.53 (s, 1H), 6.18 (s, 1H), 6.16 (s, 1H), 2.21 (s, 3H); ^13^C NMR (101 MHz, CDCl_3_): δ 168.8, 160.2 (d, *J* = 240.0 Hz), 148.4 (d, *J* = 15.0 Hz), 138.8 (d, *J* = 3.7 Hz), 133.8, 122.4 (d, *J* = 4.4 Hz), 120.9, 118.5 (d, *J* = 27.9 Hz), 115.4, 68.6 (d, *J* = 2.9 Hz), 20.8; MS (EI): *m/z* 220; Anal. Calcd for C_11_H_9_FN_2_O_2_: C, 60.00; H, 4.12; N, 12.72. Found: C, 59.88; H, 4.08; N, 12.64. After optimization, this procedure was scaled up by a factor of five with no significant decrease in yield.

#### 3.2.2. Ethyl-2-(acetoxy(2,5-difluorophenyl)methyl)acrylate (7)

Compound **7** was prepared according to the procedure given for the preparation of compound **1** [1] and did not require any further purification. Yield: 278 mg (0.98 mmol, 98%) as a colorless oil; IR: 1752, 1723, 1638 cm^−1^; ^1^H NMR: δ 7.05–6.95 (complex, 3H), 6.88 (s, 1H), 6.47 (s, 1H), 5.82 (s, 1H), 4.18 (q, *J* = 7.1 Hz, 2H), 2.12 (s, 3H), 1.24 (t, *J* = 7.1 Hz, 3H); ^13^C NMR: δ 169.0, 166.4, 164.5, 158.5 (dd, *J* = 242.7, 2.3 Hz), 156.2 (dd, *J =* 245.6, 2.5 Hz), 138.2, 126.9 (m, 2C), 116.7 (dd, *J* = 24.7, 8.5 Hz), 115.4 (dd, *J* = 24.9, 3.7 Hz), 67.0, 61.1, 20.8, 13.9; MS (EI): *m/z* 284; Anal. Calcd for C_14_H_14_F_2_O_4_: C, 59.16; H, 4.96. Found: C, 59.19; H, 4.99. After optimization, this procedure was scaled up by a factor of five with no significant decrease in yield.

### 3.3. Representative Procedure for the Synthesis of Dihydroheteroaromatics Using MBH Acetates and 1° Alkyl or Aromatic Amines

In a 25 mL, round-bottomed flask, a solution of MBH acetate (1 mmol) in DMF (2 mL) was treated with the corresponding amine (1.5 mmol), and the mixture was stirred for 1 h at room temperature (23 °C). For the dihydroquinolines, the temperature was gradually increased to 50 °C, and the reaction was stirred for an additional 4 h. For the dihydronaphthyridines, the temperature was increased gradually to 90 °C and stirring was continued for 4 h. At this time, TLC (20% EtOAc/hexane) indicated complete consumption of the starting material, the solution was poured into de-ionized water (15 mL), and extracted with EtOAc (3 × 10 mL). The organic layer was washed with 10% aq. NH_4_Cl (2 × 10 mL), followed by saturated aq. NaHCO_3_ (2 × 10 mL). The combined organic layers were washed with saturated aq. NaCl and dried (Na_2_SO_4_). Removal of the solvent under vacuum gave a crude product, which was further purified by column chromatography (10% EtOAc in hexane) to afford the dihydroheteroaromatic compounds.

#### 3.3.1. Ethyl-1-methyl-6-nitro-1,2-dihydroquinoline-3-carboxylate (9a) from 1 and Methylamine (8a)

Yield: 244 mg (93%) as a yellow solid, m.p. 117–118 °C; IR: (nujol) 1692, 1593, 1317 cm^−1^; ^1^H NMR (400 MHz, DMSO-*d_6_*): δ 8.01 (d, *J* = 2.7 Hz, 1H), 7.95 (dd, *J* = 9.2, 2.7 Hz, 1H), 7.42 (s, 1H), 6.60 (d, *J* = 9.2 Hz_,_ 1H), 4.45 (br d, *J* = 1.1 Hz, 2H), 4.20 (q, *J* = 7.1 Hz, 2H), 2.92 (s, 3H), 1.26 (t, *J* = 7.1 Hz, 3H); ^13^C NMR (101 MHz, DMSO-*d_6_*): δ 164.5, 151.9, 136.7, 133.9, 128.9, 125.8, 123.1, 118.6, 109.8, 66.0, 51.3, 37.9, 14.6; MS (EI): *m/z* 262 [M]^+·^; Anal. Calcd for C_13_H_14_N_2_O_4_: C, 59.54; H, 5.38; N, 10.68. Found: C, 59.51; H, 5.37; N, 10.75.

#### 3.3.2. Ethyl-1-hexyl-6-nitro-1,2-dihydroquinoline-3-carboxylate (**9b**) from 1 and n-hexylamine (**8b**)

Yield: 289 mg (0.87 mmol, 87%) as a yellow solid, m.p. 98–99 °C; IR: 1703, 1656, 1600, 1516, 1321 cm^−1^; ^1^H NMR (400 MHz, CDCl_3_): δ 8.00 (dd, *J* = 9.2, 2.7 Hz, 1H), 7.84 (d, *J* = 2.7 Hz, 1H), 7.28 (s, 1H), 7.25, 6.38 (d, *J* = 9.2 Hz, 1H), 4.51 (d, *J* = 1.5 Hz, 2H), 4.27 (q, *J* = 7.1 Hz, 2H), 3.26 (t, *J* = 7.8 Hz, 2H), 1.63 (quintet, *J* = 7.8 Hz, 2H), 1.42–1.31 (complex, 9H), 0.91 (t, J = 7.0 Hz, 3H); ^13^C NMR (101 MHz, CDCl_3_): δ 164.6, 150.3, 137.8, 137.3, 133.8, 128.9, 128.7, 128.5, 127.0, 125.9, 122.3, 118.5, 108.7, 61.1, 52.5, 50.1, 31.5, 14.3; MS (EI): *m/z* 332 [M]^+·^; Anal. Calcd for C_18_H_24_N_2_O_4_: C, 65.04; H, 7.28; N, 8.43. Found: C, 64.97; H, 7.24; N, 8.34.

#### 3.3.3. Ethyl-1-isobutyl-6-nitro-1,2-dihydroquinoline-3-carboxylate (**9c**) from 1 and Isobutylamine (**8c**)

Yield: 160 mg (93%) as a yellow solid, m.p. 104–105 °C; IR: (nujol) 1704, 1654, 1601, 1575, 1322 cm^−1^; ^1^H NMR (400 MHz, CDCl_3_): δ 7.97 (dd, *J* = 9.3, 2.7 Hz, 1H), 7.84 (d, *J* = 2.7 Hz, 1H), 7.28 (s, 1H), 6.41 (d, *J* = 9.3 Hz_,_ 1H), 4.52 (d, *J* = 1.5 Hz, 2H), 4.27 (q, *J* = 7.1 Hz, 2H), 3.10 (d, *J* = 7.5 Hz, 2H), 2.15 (nonet, *J* = 6.9 Hz, 1H), 1.35 (t, *J* = 7.1 Hz, 3H), 1.01 (d, *J* = 6.7 Hz, 6H); ^13^C NMR (101 MHz, CDCl_3_): δ 164.7, 151.1, 137.0, 134.0, 128.4, 126.0, 122.1, 118.4, 109.2, 61.1, 58.3, 50.9, 26.1, 20.2, 14.3; MS (EI): *m/z* 304 [M]^+·^; Anal. Calcd for C_16_H_20_N_2_O_4_: C, 63.14; H, 6.62; N, 9.20. Found: C, 63.19; H, 6.65; N, 9.13.

#### 3.3.4. Ethyl-1-benzyl-6-nitro-1,2-dihydroquinoline-3-carboxylate (**9d**) from 1 and Benzylamine (**8d**)

Yield: 313 mg (92%) as a yellow solid, m.p. 197–199 °C; IR: 1703, 1654, 1601, 1571, 1320 cm^−1^; ^1^H NMR (400 MHz, CDCl_3_): δ 7.94 (dd, *J* = 9.2, 2.7 Hz, 1H), 7.89 (d, *J* = 2.7 Hz, 1H), 7.40–7.29 (complex, 3H), 7.38 (s, 1H), 7.25 (d, J = 7.5 Hz, 2H), 6.43 (d, *J* = 9.3 Hz, 1H), 4.57 (d, *J* = 1.1 Hz, 2H), 4.54 (s, 2H), 4.26 (q, *J* = 7.1 Hz, 2H), 1.33 (t, *J* = 7.1 Hz, 3H); ^13^C NMR (101 MHz, CDCl_3_): δ 164.5, 150.8, 137.7, 134.5, 133.7, 129.1, 128.5, 127.9, 126.7, 125.8, 122.8, 118.5, 109.4, 61.1, 54.2, 50.5, 14.3; MS (EI): *m/z* 338 [M]^+·^; Anal. Calcd for C_19_H_18_N_2_O_4_: C, 67.45; H, 5.36; N, 8.28. Found: C, 67.39; H, 5.35; N, 8.19.

#### 3.3.5. Ethyl-6-nitro-1-phenethyl-1,2-dihydroquinoline-3-carboxylate (**9e**) from **1** and Phenethylamine (**8e**)

Yield: 269 mg (83%) as a yellow solid, m.p. 139–140 °C; IR: 1704, 1652, 1601, 1572, 1324 cm^−1^; ^1^H NMR (400 MHz, CDCl_3_): δ 7.99 (dd, *J* = 9.2, 2.7 Hz, 1H), 7.86 (d, *J* = 2.7 Hz, 1H), 7.34 (t, *J* = 7.4 Hz, 2H), 7.28 (s, 1H), 7.27–7.22 (complex, 3H), 6.41 (d, *J* = 9.2 Hz, 1H), 4.47 (d, *J* = 1.5 Hz, 2H), 4.27 (q, *J* = 7.1 Hz, 2H), 3.51 (t, *J* = 7.7 Hz, 2H), 2.92 (t, *J* = 7.7. Hz, 2H), 1.34 (t, *J* = 7.1 Hz, 3H); ^13^C NMR (101 MHz, CDCl_3_): δ 164.6, 150.3, 137.8, 137.3, 133.8, 128.9, 128.7, 128.5, 127.0, 125.9, 122.3, 118.5, 108.7, 61.1, 52.5, 50.1, 31.5, 14.3; MS (EI): *m/z* 352 [M]^+·^; Anal. Calcd for C_20_H_20_N_2_O_4_: C, 68.17; H, 4.97; N, 6.84. Found: C, 68.12; H, 4.91; N, 6.77.

#### 3.3.6. Ethyl-6-nitro-1-phenyl-1,2-dihydroquinoline-3-carboxylate (**9f**) from **1** and Aniline (**8f**)

Yield: 313 mg (89%) as a yellow solid, m.p. 149–150 °C; IR: 1692,1601,1518,1318 cm^−1^; ^1^H NMR (400 MHz, CDCl_3_): δ 7.99 (dd, *J* = 9.2, 2.7 Hz, 1H), 7.86 (d, *J* = 2.7 Hz, 1H), 7.34 (t, *J* = 7.4 Hz, 2H), 7.28 (s, 1H), 7.25 (obscured, 2H), 6.41 (d, *J* = 9.2 Hz, 1H), 4.47 (d, *J* = 1.5 Hz, 2H), 4.27 (q, *J* = 7.1 Hz, 2H), 3.51 (t, *J* = 8.0 Hz, 2H), 2.92 (t, *J* = 8.0 Hz, 2H), 1.35 (t, *J* = 7.1 Hz, 3H); ^13^C NMR (101 MHz, CDCl_3_): δ 164.5, 150.7, 143.7, 138.7, 133.4, 130.4, 127.4, 125.7, 125.6, 123.2, 119.8, 112.6, 61.2, 51.1, 14.3 (1 aromatic carbon unresolved); MS (EI): *m*/*z* 324 [M]^+^; Anal. Calcd for C_18_H_16_N_2_O_4_: C, 66.66; H, 4.97; N, 8.64. Found: C, 66.59; H, 4.94; N, 8.69.

#### 3.3.7. 1-Methyl-6-nitro-1,2-dihydroquinoline-3-carbonitrile (**10a**) from **2** and **8a**

Yield: 189 mg (88%) as an orange solid, m.p. 186–187 °C; IR: 2210, 1640, 1515, 1321 cm^−1^; ^1^H NMR (400 MHz, CDCl_3_): δ 8.05 (dd, *J* = 9.2, 2.7 Hz, 1H), 7.82 (d, *J* = 2.7 Hz, 1H), 7.00 (br s, 1H), 6.47 (d, *J* = 9.2 Hz, 1H), 4.41 (d, *J* = 1.6 Hz, 2H), 2.94 (s, 3H); ^13^C NMR (100 MHz, CDCl_3_): δ 150.5, 139.0, 137.7, 128.7, 125.0, 117.5, 116.5, 109.6, 103.7, 51.5, 37.6; MS (EI): *m*/*z* 215 [M]^+·^. Anal. Calcd for C_11_H_9_N_3_O_2_: C, 61.39; H, 4.22; N, 19.53. Found: C, 61.41; H, 4.25; N, 19.44.

#### 3.3.8. 1-Hexyl-6-nitro-1,2-dihydroquinoline-3-carbonitrile (**10b**) from **2** and **8b**

Yield: 222 mg (78%) as a yellow solid, m.p. 110–111 °C; IR: 2228, 1647, 1532, 1344 cm^−1^; ^1^H NMR (400 MHz, CDCl_3_): δ 8.02 (dd, *J* = 9.3, 2.7 Hz, 1H), 7.80 (d, *J* = 2.7 Hz, 1H), 6.97 (br s, 1H), 6.44 (d, *J* = 9.3 Hz, 1H), 4.43 (s, 2H), 3.25 (t, *J* = 7.2 Hz, 2H), 1.62 (quintet, *J* = 7.5 Hz, 2H), 1.36 (m, 6H), 0.92 (t, *J* = 6.8 Hz, 3H); ^13^C NMR (101 MHz, CDCl_3_): δ 149.8, 139.2, 137.6, 128.9, 125.5, 117.4, 116.7, 109.6, 103.3, 50.9, 50.3, 31.5, 26.6, 25.0, 22.6, 14.0; MS (EI): *m*/*z* 285 [M]^+·^; Anal. Calcd for C_16_H_19_N_3_O_2_: C, 67.35; H, 6.71; N, 14.73. Found: C, 67.46; H, 6.76; N, 14.63.

#### 3.3.9. 1-Isobutyl-6-nitro-1,2-dihydroquinoline-3-carbonitrile (**10c**) from **2** and **8c**

Yield: 203 mg (79%) as a yellow solid, m.p. 158–159 ^o^C; IR: 2208, 1642, 1519, 1324 cm^−1^; ^1^H NMR (400 MHz, CDCl_3_): δ 8.00 (d, *J* = 9.3 Hz, 1H), 7.80 (s, 1H), 6.96 (s, 1H), 6.47 (d, *J* = 9.3 Hz, 1H), 4.44 (s, 2H), 3.08 (d, *J* = 7.5 Hz, 2H), 2.11 (septet, *J* = 6.7 Hz, 1H), 1.02 (d, *J* = 6.7 Hz, 6H); ^13^C NMR (101 MHz, CDCl_3_): δ 150.3, 139.3, 137.6, 128.7, 125.6, 117.4, 116.7, 110.2, 103.1, 58.4, 51.3, 26.2, 20.2, 14.3; MS (EI): *m*/*z* 257 [M]^+·^; Anal. Calcd for C_14_H_15_N_3_O_2_: C, 65.36; H, 5.88; N, 16.33. Found: C, 65.41; H, 5.91; N, 16.25.

#### 3.3.10. 1-Benzyl-6-nitro-1,2-dihydroquinoline-3-carbonitrile (**10d**) from **2** and **8d**

Yield: 233 mg (80%) as a yellow solid, m.p. 154–155 °C; IR: 2231, 1648, 1530, 1345 cm^−1^; ^1^H NMR (400 MHz, CDCl_3_): δ 7.99 (dd, *J* = 9.2, 2.6 Hz, 1H), 7.86 (d, *J* = 2.6 Hz, 1H), 7.43–7.32 (complex, 3H), 7.23 (d, *J* = 8.5 Hz, 2H), 7.04 (s, 1H), 6.54 (d, *J* = 9.2 Hz, 1H), 4.52 (s, 2H), 4.44 (d, *J* = 1.6 Hz, 2H); ^13^C NMR (101 MHz, CDCl_3_): δ 150.1, 139.0, 138.3, 133.8, 129.3, 128.9, 128.3, 126.8, 125.5, 117.6, 116.5, 110.4, 103.9, 54.1, 50.4; MS (EI): *m*/*z* 291 [M]^+·^; Anal. Calcd for C_17_H_13_N_3_O_2_: C, 70.09; H, 4.50; N, 14.42. Found: C, 70.04; H, 4.53; N, 14.38.

#### 3.3.11. 6-Nitro-1-phenethyl-1,2-dihydroquinoline-3-carbonitrile (**10e**) from **2** and **8e**

Yield: 250 mg (82%) as a yellow solid, m.p. 121–122 °C; IR: 2239, 1623, 1568, 1344 cm^−1^; ^1^H NMR (400 MHz, CDCl_3_): δ 8.02 (dd, *J* = 9.2, 2.7 Hz, 1H), 7.81 (d, *J* = 2.7 Hz, 1H), 7.38–7.18 (complex, 5H), 6.95 (s, 1H), 6.47 (d, *J* = 9.3 Hz, 1H), 4.28 (s, 2H), 3.52 (t, *J* = 7.4 Hz, 2H), 2.92 (t, *J* = 7.4 Hz, 2H); ^13^C NMR (101 MHz, CDCl_3_): δ 149.5, 139.0, 137.9, 137.5, 129.1, 128.9, 128.7, 127.2, 125.6, 117.5, 116.5, 109.6, 103.4, 52.6, 50.7, 31.8; MS (EI): *m*/*z* 305 [M]^+·^; Anal. Calcd for C_18_H_15_N_3_O_2_: C, 70.81; H, 4.95; N, 13.76. Found: C, 70.76; H, 4.89; N, 13.68.

#### 3.3.12. 6-Nitro-1-phenyl-1,2-dihydroquinoline-3-carbonitrile (**10f**) from **2** and **8f**

Yield: 224 mg (81%) as a yellow solid, m.p. 161–163 °C; IR: 2210, 1633, 1570, 1326 cm^−1^; ^1^H NMR (400 MHz, CDCl_3_): δ 7.94 (d, *J* = 2.7 Hz, 1H), 7.88 (dd, *J* = 9.3, 2.7 Hz, 1H), 7.52 (t, *J* = 7.5 Hz, 2H), 7.39 (t, *J* = 7.5 Hz, 1H), 7.28 (d, *J* = 7.5 Hz, 2H), 7.14 (s, 1H), 6.41 (d, *J* = 9.3 Hz, 1H), 4.69 (d, *J* = 1.5 Hz, 2H); ^13^C NMR (101 MHz, CDCl_3_): δ 149.9, 143.0, 139.1, 138.7, 130.6, 127.9, 127.8, 125.5, 125.2, 118.8, 116.5, 113.5, 103.9, 51.4; MS (EI): *m*/*z* 277 [M]^+·^; Anal. Calcd for C_16_H_11_N_3_O_2_: C, 69.31; H, 4.00; N, 15.15. Found: C, 69.37; H, 3.98; N, 15.03.

#### 3.3.13. Ethyl-6-cyano-1-methyl-1,2-dihydroquinoline-3-carboxylate (**11a**) from **3** and **8a**

Yield: 209 mg (91%) as a yellow solid, m.p. 140–141 °C; IR: 2201, 1708, 1652 cm^−1^; ^1^H NMR (400 MHz, CDCl_3_): δ 7.35 (dd, *J* = 8.6, 2.1 Hz, 1H), 7.23 (s, 1H), 7.17 (d, *J* = 2.1 Hz, 1H), 6.42 (d, *J* = 8.6 Hz, 1H), 4.41 (d, *J* = 1.5 Hz, 2H), 4.27 (q, *J* = 7.1 Hz, 2H), 2.87 (s, 3H), 1.34 (t, *J* = 7.1 Hz, 3H); ^13^C NMR (101 MHz, CDCl_3_): δ 164.8, 149.6, 135.9, 133.7, 133.0, 122.7, 119.9, 119.8, 109.9, 98.6, 61.0, 51.0, 37.4, 14.3; MS (EI): *m*/*z* 242 [M]^+·^; Anal. Calcd for C_14_H_14_N_2_O_2_: C, 69.41; H, 5.82; N, 11.56. Found: C, 69.36; H, 5.78; N, 11.43.

#### 3.3.14. Ethyl-6-cyano-1-hexyl-1,2-dihydroquinoline-3-carboxylate (**11b**) from **3** and **8b**

Yield: 256 mg (82%) as a yellow semisolid; IR: 2216, 1705, 1651 cm^−1^; ^1^H NMR (400 MHz, CDCl_3_): δ 7.31 (dd, *J* = 8.8, 2.1 Hz, 1H), 7.20 (s, 1H), 7.15 (d, *J* = 2.1 Hz, 1H), 6.40 (d, *J* = 8.8 Hz, 1H), 4.44 (d, *J* = 1.5 Hz, 2H), 4.26 (q, *J* = 7.1 Hz, 2H), 3.19 (t, *J* = 7.8 Hz, 2H), 1.56 (m, 2H), 1.34 (superimposed m, 6H and t, *J* = 7.1 Hz, 3H), 0.91 (t, *J* = 6.7 Hz, 3H); ^13^C NMR (101 MHz, CDCl_3_): δ 164.9, 148.8, 135.8, 133.8, 133.4, 122.0, 120.0, 119.5, 109.8, 97.7, 61.0, 50.4, 49.5, 31.6, 26.6, 24.8, 22.6, 14.3, 14.0; MS (EI): *m*/*z* 312 [M]^+·^; Anal. Calcd for C_19_H_24_N_2_O_2_: C, 73.05; H, 7.74; N, 8.97. Found: C, 73.14; H, 7,79; N, 8.89.

#### 3.3.15. Ethyl-6-cyano-1-isobutyl-1,2-dihydroquinoline-3-carboxylate (**11c**) from **3** and **8c**

Yield: 241 mg (85%) as a yellow solid, m.p. 117–118 °C; IR: 2214, 1690, 1639, 1603, 1515 cm^−1^; ^1^H NMR (400 MHz, CDCl_3_): δ 7.29 (dd, *J* = 8.8 Hz, 1H), 7.20 (s, 1H), 7.16 (d, *J* = 2.0 Hz, 1H), 6.42 (d, *J* = 8.8 Hz, 1H), 4.46 (d, *J* = 1.5 Hz, 2H), 4.26 (q, *J* = 7.1 Hz, 2H), 3.03 (d, *J* = 7.5 Hz, 2H), 2.10 (nonet, *J* = 6.7 Hz, 1H); 1.34 (t, *J* = 7.1 Hz, 3H), 0.98 (d, *J* = 6.7 Hz, 6H); ^13^C NMR (101 MHz, CDCl_3_): δ 164.9, 149.3, 135.6, 133.9, 133.5, 121.8, 119.9, 119.5, 110.3, 97.7, 61.0, 58.0, 50.5, 26.0, 20.2, 14.3; MS (EI): *m*/*z* 284 [M]^+·^; Anal. Calcd for C_17_H_20_N_2_O_2_: C, 71.81; H, 7.09; N, 9.85. Found: C, 71.85; H, 7.12; N, 9.79.

#### 3.3.16. Ethyl-1-benzyl-6-cyano-1,2-dihydroquinoline-3-carboxylate (**11d**) from **3** and **8d**

Yield: 256 mg (79%) as a yellow solid, m.p. 97–98 °C; IR: 2219, 1703, 1642, 1602, 1517 cm^−1^; ^1^H NMR (400 MHz, CDCl_3_): δ 7.36 (t, *J* = 7.5 Hz, 2H), 7.33–7.20 (complex, 6H), 6.44 (d, *J* = 8.6 Hz, 1H), 4.51 (d, *J* = 1.5 Hz, 2H), 4.48 (s, 2H), 4.25 (q, *J* = 7.1 Hz, 2H), 1.32 (t, *J* = 7.1 Hz, 3H); ^13^C NMR (101 MHz, CDCl_3_): δ 164.7, 149.0, 137.9, 134.9, 133.6, 133.3, 129.1, 127.7, 126.7, 122.4, 119.7, 119.6, 110.5, 98.7, 61.0, 53.8, 50.1, 14.3; MS (EI): *m*/*z* 318 [M]^+·^; Anal. Calcd for C_20_H_18_N_2_O_2_: C, 75.45; H, 5.70; N, 8.80. Found: C, 75.38; H, 5.68; N, 8.67.

#### 3.3.17. Ethyl-6-cyano-1-phenethyl-1,2-dihydroquinoline-3-carboxylate (**11e**) from **3** and **8e**

Yield: 270 mg (85%) as a yellow solid, m.p. 131–132 °C; IR: 2217, 1701, 1650 cm^−1^; ^1^H NMR (400 MHz, CDCl_3_): δ 7.36–7.29 (complex, 3H), 7.25–7.18 (complex, 4H), 7.18 (d, *J* = 2.1 Hz, 1H), 6.44 (d, *J* = 8.7 Hz, 1H), 4.43 (d, *J* = 1.6 Hz, 2H), 4.26 (q, *J* = 7.1 Hz, 2H), 3.45 (t, *J* = 7.9 Hz, 2H), 2.88 (t, *J* = 7.9 Hz, 2H), 1.33 (t, *J* = 7.1 Hz, 3H); ^13^C NMR (101 MHz, CDCl_3_): δ 164.8, 148.5, 138.1, 135.8, 132.7, 133.4, 128.9, 128.7, 126.8, 122.1, 119.9, 119.7, 109.8, 98.2, 61.0, 52.1, 49.6, 31.3, 14.3; MS (EI): *m*/*z* 332 [M]^+·^; Anal. Calcd for C_21_H_20_N_2_O_2_: C, 75.88; H, 6.06; N, 8.43. Found: C, 75.81; H, 6.05; N, 8.35.

#### 3.3.18. Ethyl-6-cyano-1-phenyl-1,2-dihydroquinoline-3-carboxylate (**11f**) from **3** and **8f**

Yield: 252 mg (83%) as a yellow solid, m.p. 129–130 °C; IR: 2254, 1698, 1644 cm^−1^; ^1^H NMR (400 MHz, CDCl_3_): δ 7.47 (t, *J* = 7.8 Hz, 2H), 7.36 (s, 1H), 7.33–7.24 (complex, 4H), 7.19 (dd, *J* = 8.7, 2.0 Hz, 1H), 6.44 (d, *J* = 8.7 Hz, 1H), 4.71 (d, *J* = 1.5 Hz, 2H), 4.27 (q, *J* = 7.1 Hz, 2H), 1.33 (t, *J* = 7.1 Hz, 3H); ^13^C NMR (101 MHz, CDCl_3_): δ 164.6, 148.8, 143.9, 134.8, 133.22, 133.16, 130.2, 126.8, 125.4, 123.1, 121.1, 119.6, 113.8, 100.3, 61.1, 50.4, 14.3; MS (EI): *m*/*z* 304 [M]^+·^; Anal. Calcd for C_19_H_16_N_2_O_2_: C, 74.98; H, 5.30; N, 9.20. Found: C, 75.07; H, 5.31; N, 9.08.

#### 3.3.19. 1-Methyl-1,2-dihydroquinoline-3,6-dicarbonitrile (**12a**) from **4** and **8a**

Yield: 160 mg (82%) as a yellow solid, m.p. 205–206 °C; IR: 2253, 1625 cm^−1^; ^1^H NMR (400 MHz, CDCl_3_): δ 7.39 (d, *J* = 8.6 Hz, 1H), 7.15 (s, 1H), 6.94 (s, 1H), 6.48 (d, *J* = 8.6 Hz, 1H), 4.33 (s, 2H), 2.87 (s, 3H); ^13^C NMR (101 MHz, CDCl_3_): δ 148.9, 139.0, 136.4, 132.7, 119.2, 119.0, 116.8, 110.8, 103.7, 99.8, 51.4, 37.4; MS (EI): *m*/*z* 195 [M]^+·^; Anal. Calcd for C_12_H_9_N_3_: C, 73.83; H, 4.65; N, 21.52. Found: C, 73.79; H, 4.63; N, 21.37.

#### 3.3.20. 1-Hexyl-1,2-dihydroquinoline-3,6-dicarbonitrile (**12b**) from **4** and **8b**

Yield: 204 mg (77%) as a yellow solid, m.p. 178–179 °C; IR: 2254, 1651 cm^−1^; ^1^H NMR (400 MHz, CDCl_3_): δ 8.76 (s, 1H), 8.09 (s, 1H), 7.96 (d, *J* = 8.9 Hz, 1H), 7.60 (d, *J* = 8.9 Hz, 1H), 4.22 (t, *J* = 7.4 Hz, 2H), 1.91 (quintet, *J* = 7.4 Hz, 2H), 1.50–1.28 (complex, 8H), 0.91 (t, *J* = 6.7 Hz, 3H); ^13^C NMR (101 MHz, CDCl_3_): δ 172.8, 149.5, 141.2, 135.5, 132.9, 127.0, 117.5, 117.3, 114.5, 109.7, 97.9, 54.8, 31.2, 28.8, 26.3, 22.4, 13.9; MS (EI): *m*/*z* 265 [M]^+·^; Anal. Calcd for C_17_H_19_N_3_: C, 76.95; H, 7.22; N, 15.84. Found: C, 77.03; H, 7.18; N, 15.69.

#### 3.3.21. 1-Isobutyl-1,2-dihydroquinoline-3,6-dicarbonitrile (**12c**) from **4** and **8c**

Yield 192 mg (81%) as a yellow solid, m.p. 131–132 °C; IR: 2229, 1626 cm^−1^; ^1^H NMR (400 MHz, CDCl_3_): δ 7.33 (dd, *J* = 8.8, 2.1 Hz, 1H), 7.14 (d, *J* = 2.1 Hz, 1H), 6.92 (s, 1H), 6.48 (d, *J* = 8.8 Hz, 1H), 4.38 (d, *J* = 1.5 Hz, 2H), 3.02 (d, *J* = 7.5 Hz, 2H), 2.06 (septet, *J* = 6.6 Hz, 1H), 1.00 (d, *J* = 6.6 Hz, 6H); ^13^C NMR (101 MHz, CDCl_3_): δ 148.6, 139.1, 136.1, 133.2, 119.3, 118.7, 116.9, 111.2, 102.7, 98.9, 58.1, 50.9, 26.2, 20.2; MS (EI): *m*/*z* 237 [M]^+·^; Anal. Calcd for C_15_H_15_N_3_: C, 75.92; H, 6.37; N, 17.71. Found: C, 75.97; H, 6.41; N, 17.60.

#### 3.3.22. 1-Benzyl-1,2-dihydroquinoline-3,6-dicarbonitrile (**12d**) from **4** and **8d**

Yield: 231 mg (86%) as a yellow solid, m.p. 140–141 °C; IR: 2215, 1639, 1603, 1514 cm^−1^; ^1^H NMR (400 MHz, CDCl_3_): δ 7.42–7.30 (complex, 4H), 7.23 (d, *J* = 7.0 Hz, 2H), 7.20(d, *J* = 2.0 Hz, 1H), 6.97 (s, 1H), 6.54 (d, *J* = 8.8 Hz, 1H), 4.46 (s, 2H), 4.38 (d, *J* = 1.5 Hz, 2H); ^13^C NMR (101 MHz, CDCl_3_): δ 148.4, 138.8, 136.4, 134.2, 133.0, 129.2, 128.1, 126.8, 119.1, 118.7, 116.8, 111.4, 103.6, 99.9, 53.8, 50.1; MS (EI): *m*/*z* 271 [M]^+·^; Anal. Calcd for C_18_H_13_N_3_: C, 79.68; H, 4.83; N, 15.49. Found: C, 79.73; H, 4.84; N, 15.41.

#### 3.3.23. 1-Phenethyl-1,2-dihydroquinoline-3,6-dicarbonitrile (**12e**) from **4** and **8e**

Yield: 251 mg (88%) as a yellow solid, m.p. 129–130 °C; IR: 2215, 1639, 1603, 1514 cm^−1^; ^1^H NMR (400 MHz, CDCl_3_): δ 7.37–7.27 (complex, 4H), 7.21 (d, *J* = 8.3 Hz, 2H), 7.15 (d, *J* = 2.0 Hz, 1H), 6.89 (t, *J* = 1.6 Hz, 1H), 6.50 (d, *J* = 9.0 Hz, 1H), 4.25 (d, *J* = 1.6 Hz, 2H), 3.46 (t, *J* = 7.4 Hz, 2H), 2.89 (t, *J* = 7.4 Hz, 2H); ^13^C NMR (101 MHz, CDCl_3_): δ 147.8, 138.8, 137.7, 136.3, 133.2, 129.0, 128.7, 127.1, 119.2, 118.8, 116.8, 110.7, 103.0, 99.3, 52.2, 50.3, 31.6; MS (EI): *m*/*z* 285 [M]^+·^; Anal. Calcd for C_19_H_15_N_3_: C, 79.98; H, 5.30; N, 14.73. Found: C, 79.88; H, 5.26; N, 14.61.

#### 3.3.24. Ethyl-1-methyl-1,2-dihydro-1,8-naphthyridine-3-carboxylate (**13a**) from **5** and **8a**

Yield: 191 mg (88%) as a yellow solid, m.p. 65–66 °C; IR: 1701, 1646, 1556, 1395, 1222 cm^−1^; ^1^H NMR (400 MHz, CDCl_3_): δ 7.95 (dd, *J* = 5.1, 1.8 Hz, 1H), 7.23 (t, *J* = 1.2 Hz, 1H) 7.10 (dd, *J* = 7.2, 1.8 Hz, 1H) 6.41 (dd, *J* = 7.2, 5.1 Hz, 1H) 4.48 (d, *J* = 1.2 Hz, 2H), 4.26 (q, *J* = 7.1 Hz, 2H), 3.00 (s, 3H), 1.33 (t, *J* = 7.2 Hz, 3H); ^13^C NMR (101 MHz, CDCl_3_): δ 165.1, 156.7, 150.1, 135.9, 134.5, 122.1, 114.6, 112.5, 60.8, 51.1, 35.2, 14.3; MS (EI): *m*/*z* 218 [M]^+·^; Anal. Calcd for C_12_H_14_N_2_O_2_: C, 66.04; H, 6.47; N, 12.84. Found: C, 65.95; H, 6.43; N, 12.87.

#### 3.3.25. Ethyl-1-hexyl-1,2-dihydro-1,8-naphthyridine-3-carboxylate (**13b**) from **5** and **8b**

Yield: 230 mg (80%) as a yellow oil; IR: 1703, 1647 cm^−1^; ^1^H NMR (400 MHz, CDCl_3_): δ 7.92 (dd, *J* = 5.1, 1.9 Hz, 1H), 7.19 (t, *J* = 1.4 Hz, 1H), 7.06 (dd, *J* = 7.2, 1.9 Hz, 1H), 6.35 (dd, *J* = 7.2, 5.1 Hz, 1H), 4.51 (d, *J* = 1.4 Hz, 2H), 4.25 (q, *J* = 7.1 Hz, 2H), 3.46 (t, *J* = 7.6 Hz, 2H), 1.60 (quintet, *J* = 7.6 Hz, 2H), 1.33 (overlapping t, *J* = 7.1 Hz, 3H and m, 6 H), 0.89 (t, *J* = 6.8 Hz, 3H); ^13^C NMR (101 MHz, CDCl_3_): δ 165.2, 156.3, 150.2, 136.0, 134.6, 121.6, 114.2, 112.0, 60.7, 48.9, 47.5, 31.8, 26.7, 25.6, 22.7, 14.3, 14.1; MS (EI): *m*/*z* 288 [M]^+·^; Anal. Calcd for C_17_H_24_N_2_O_2_: C, 70.80; H, 8.39; N, 9.71. Found: C, 70.92; H, 8.34; N, 9.66.

#### 3.3.26. Ethyl-1-isobutyl-1,2-dihydro-1,8-naphthyridine-3-carboxylate (**13c**) from **5** and **8c**

Yield: 208 mg (80%) as a yellow oil; IR: 1702, 1648 cm^−1^; ^1^H NMR (400 MHz, CDCl_3_): δ 7.91 (dd, *J* = 5.0, 1.9 Hz, 1H), 7.20 (t, *J* = 1.5 Hz, 1H), 7.07 (dd, *J* = 7.2, 1.9 Hz, 1H), 6.36 (dd, *J* = 7.2, 5.0 Hz, 1H), 4.50 (d, *J* = 1.5 Hz, 2H), 4.25 (q, *J* = 7.2 Hz, 2H), 3.34 (d, *J* = 7.6 Hz, 2H), 2.13 (nonet, *J* = 6.9 Hz, 1H), 1.33 (t, *J* = 7.2 Hz, 3H), 0.95 (d, *J* = 6.7 Hz, 6H); ^13^C NMR (101 MHz, CDCl_3_): δ 165.2, 156.7, 150.1, 136.1, 134.7, 121.5, 114.1, 112.0, 60.7, 54.5, 49.5, 25.7, 20.1, 14.3; MS (EI): *m*/*z* 260 [M]^+·^; Anal. Calcd for C_15_H_20_N_2_O_2_: C, 69.20; H, 7.74; N, 10.76. Found: C, 69.29; H, 7.70; N, 10.63.

#### 3.3.27. Ethyl-1-benzyl-1,2-dihydro-1,8-naphthyridine-3-carboxylate (**13d**) from **5** and **8d**

Yield: 238 mg (81%) as a yellow oil; IR: 1702, 1651 cm^−1^; ^1^H NMR (400 MHz, CDCl_3_): δ 7.96 (dd, *J* = 5.0, 1.9 Hz, 1H), 7.35–7.26 (complex, 4H), 7.25 (m, 1H), 7.22 (s, 1H), 7.13 (dd, *J* = 7.2, 1.9 Hz, 1H), 6.44 (dd, *J* = 7.2, 5.0 Hz, 1H), 4.78 (s, 2H), 4.42 (d, *J* = 1.5 Hz, 2H), 4.21 (q, *J* = 7.1 Hz, 2H), 1.29 (t, *J* = 7.1 Hz, 3H); ^13^C NMR (101 MHz, CDCl_3_): δ 165.1, 156.2, 150.2,. 137.1, 136.3, 134.3, 128.6, 128.0, 127.2, 122.0, 114.0, 112.7, 60.8, 50.4, 48.6, 14.3; MS (EI): *m*/*z* 294 [M]^+·^; Anal. Calcd for C_18_H_18_N_2_O_2_: C, 73.45: H, 6.16; N, 9.52. Found: C, 73.51; H, 6.11; N, 9.45.

#### 3.3.28. Ethyl-1-phenethyl-1,2-dihydro-1,8-naphthyridine-3-carboxylate (**13e**) from **5** and **8e**

Yield: 256 mg (83%) as a yellow oil; IR: 1702, 1647 cm^−1^; ^1^H NMR (400 MHz, CDCl_3_): δ 7.97 (dd, *J* = 5.1, 1.9 Hz, 1H), 7.30 (apparent s, 2H), 7.29 (apparent s, 2H), 7.19 (superimposed m, 1H and s, 1H), 7.08 (dd, *J* = 7.2, 1.9 Hz, 1H), 6.40 (dd, *J* = 7.2, 5.1 Hz, 1H), 4.48 (d, *J* = 1.5 Hz, 2H), 4.24 (q, *J* = 7.1 Hz, 2H), 3.70 (t, *J* = 7.7 Hz, 2H), 2.92 (t, *J* = 7.7 Hz, 2H), 1.32 (t, *J* = 7.1 Hz, 3H); ^13^C NMR (101 MHz, CDCl_3_): δ 165.1, 156.0, 150.3, 139.6, 136.0, 134.5, 128.9, 128.4, 126.2, 121.7, 114.3, 112.3, 60.8, 49.5, 49.3, 32.1, 14.3; MS (EI): *m*/*z* 308 [M]^+·^; Anal. Calcd for C_19_H_20_N_2_O_2_: C, 74.00: H, 6.54; N, 9.08. Found: C, 73.88; H, 6.51; N, 8.96.

#### 3.3.29. Ethyl-1-phenyl-1,2-dihydro-1,8-naphthyridine-3-carboxylate (**13f**) from **5** and **8f**

Yield: 215 mg (77%) as a yellow oil; IR: 1703, 1647 cm^−1^; ^1^H NMR (400 MHz, CDCl_3_): δ 7.95 (dd, *J* = 5.0, 1.8 Hz, 1H), 7.42 (t, *J* = 7.8 Hz, 2H), 7.38–7.32 (complex, 3H), 7.28 (dd, *J* = 7.3, 1.8 Hz, 1H), 7.21 (t, *J* = 7.2 Hz, 1H), 6.59 (dd, *J* = 7.3, 5.0 Hz, 1H), 4.84 (d, *J* = 1.4 Hz, 2H), 4.26 (q, *J* = 7.2 Hz, 2H), 1.32 (t, *J* = 7.2 Hz, 3H); ^13^C NMR (101 MHz, CDCl_3_): δ 164.8, 155.8, 150.0, 144.1, 136.5, 133.8, 129.1, 125.5, 125.0, 123.0, 116.1, 114.7, 60.9, 50.5, 14.3; MS (EI): *m*/*z* 280 [M]^+·^; Anal. Calcd for C_17_H_16_N_2_O_2_: C, 72.84: H, 5.75: N, 9.99. Found: C, 72.71; H, 5.68; N, 9.92.

#### 3.3.30. 1-Hexyl-1,2-dihydro-1,8-naphthyridine-3-carbonitrile (**14b**) from **6** and **8b**

Yield: 198 mg (0.82 mmol, 82%) as a yellow semisolid; IR: 2254, 1639 cm^−1^; ^1^H NMR (400 MHz, CDCl_3_): δ 7.97 (dd, *J* = 5.0, 1.8 Hz, 1H), 7.04 (dd, *J* = 7.3, 1.8 Hz, 1H), 6.87 (t, *J* = 1.4 Hz, 1H), 6.42 (dd, *J* = 7.3, 5.0 Hz, 1H), 4.44 (d, *J* = 1.4 Hz, 2H), 3.45 (t, *J* = 7.4 Hz, 2H), 1.58 (quintet, *J* = 7.2 Hz, 2H), 1.38–1.28 (complex, 6H), 0.90 (t, *J* = 6.9 Hz, 3H); ^13^C NMR (100 MHz, CDCl_3_): δ 155.5, 151.0, 139.8, 135.9, 117.5, 113.4, 112.7, 102.4, 49.6, 47.4, 31.7, 26.6, 25.7, 22.6, 14.1; MS (EI): *m*/*z* 241 [M]^+·^; Anal. Calcd for C_15_H_19_N_3_: C, 74.65; H, 7.94; N, 17.41. Found: C, 74.77; H, 7.98; N, 17.29.

#### 3.3.31. 1-Benzyl-1,2-dihydro-1,8-naphthyridine-3-carbonitrile (**14d**) from **6** and **8d**

Yield: 14 mg (0.057 mmol, 5.7%) as a yellow oil; IR: 2209, 1638 cm^−1^: ^1^H NMR (400 MHz, CDCl_3_): δ 8.02 (dd, *J* = 5.0, 1.9 Hz, 1H), 7.37–7.28 (complex, 5H), 7.11 (dd, *J* = 7.3, 1.9 Hz, 1H), 6.90 (t, *J* = 1.5 Hz, 1H), 6.51 (dd, *J* = 7.3, 5.0 Hz, 1H), 4.76 (s, 2H), 4.31 (d, *J* = 1.5 Hz, 2H); ^13^C NMR (101 MHz, CDCl_3_): δ 155.4, 150.9, 139.4, 136.3, 136.1, 128.7, 128.2, 127.6, 117.3, 113.4, 113.2, 102.9, 50.2, 49.8; MS (EI): *m*/*z* 247 [M]^+·^; Anal. Calcd for C_16_H_13_N_3_: C, 77.71; H, 5.30; N, 16.99. Found: C, 77.67; H, 5.24; N, 16.87.

#### 3.3.32. 1-Benzyl-1,4-dihydro-1,8-naphthyridine-3-carbonitrile (**15d**) from **6** and **8d**

Double bond-migrated product; Yield: 203 mg (0.82 mmol, 82%) as a white powder (slowly darkens on exposure to air and light), m.p. 117–118 °C; IR: 2000, 1651 cm^−1^; ^1^H NMR (400 MHz, CDCl_3_): δ 8.09 (d, *J* = 4.9 Hz, 1H), 7.37–7.25 (complex, 6H), 6.87 (dd, *J* = 7.4, 4.9 Hz, 1H), 6.84 (s, 1H), 4.99 (s, 2H), 3.81 (s, 2H); ^13^C NMR (101 MHz, CDCl_3_): δ 149.6, 146.4, 144.0, 137.3, 137.1, 128.8, 127.7, 127.6, 120.3, 119.2, 115.3, 78.8, 51.0, 27.9; MS (EI): *m*/*z* 247 [M]^+·^; Anal. Calcd for C_16_H_13_N_3_: C, 77.71; H, 5.30; N, 16.99. Found: C,77.69; H, 5.29; N, 16.94. The X-ray structure for **15d** (CCDC 2035027) and the thermal ellipsoid plot appears in Figure 3 and the SI.

#### 3.3.33. 1-Phenethyl-1,2-dihydro-1,8-naphthyridine-3-carbonitrile (**14e**) from **6** and **8e**

Yield: 209 mg (0.80 mmol, 80%) as a yellow semisolid; IR: 2254, 1639 cm^−1^; ^1^H NMR (400 MHz, CDCl_3_): δ 8.02 (dd, *J* = 5.0, 1.9 Hz, 1H), 7.35–7.21 (complex, 5H), 7.06 (dd, *J* = 7.3, 1.9 Hz, 1H), 6.86 (t, *J* = 1.4 Hz, 1H), 6.46 (dd, *J* = 7.3, 5.0 Hz, 1H), 4.32 (d, *J* = 1.4 Hz, 2H), 3.68 (t, *J* = 7.4 Hz, 2H), 2.92 (t, *J* = 7.1 Hz, 2H); ^13^C NMR (100 MHz, CDCl_3_): δ 155.2, 151.1, 139.6, 135.8, 128.9, 128.6, 126.5, 117.3, 113.5, 113.0, 102.5, 50.3, 49.6, 32.4; MS (EI): *m*/*z* 261 [M]^+·^; Anal. Calcd for C_17_H_15_N_3_: C, 78.13; H, 5.79; N, 16.08. Found: C, 78.06; H, 5.77; N, 16.01.

#### 3.3.34. 1-Phenyl-1,4-dihydro-1,8-naphthyridine-3-carbonitrile (**15f**) from **6** and **8f**

Double bond-migrated product; Yield: 168 mg (0.72 mmol, 72%) as a white powder (slowly darkens on exposure to air and light), m.p. 162–163 °C; IR: 2193, 1645 cm^−1^; ^1^H NMR (400 MHz, CDCl_3_): δ 8.15 (dd, *J* = 5.0, 1.6 Hz, 1H), 7.82 (ddd, *J* = 9.5, 7.3, 1.9 Hz, 1H), 7.39–7.30 (complex, 3H), 7.23 (ddd, *J* = 7.3, 5.0, 1.6 Hz, 1H), 7.06 (t, *J* = 7.4 Hz, 1H), 6.93 (d, *J* = 7.4 Hz, 2H), 3.53 (s, 2H); ^13^C NMR (101 MHz, CDCl_3_): δ 146.5, 146.3, 141.1, 140.9, 139.9, 129.9, 123.4, 122.3, 121.8, 119.4, 115.6, 80.5, 25.7; MS (EI): *m*/*z* 233 [M]^+·^; Anal. Calcd for C_15_H_11_N_3_: C, 77.23; H, 4.75; N, 18.01. Found: C, 77.12; H, 4.69; N, 17.93.

### 3.4. Control Experiment Attempted Substitution of Benzylamine (**8d**) on 2-fluoropyridine and other S_N_Ar Activated Rings

A 50 mL round-bottomed flask was charged with 2-fluoropyridine (97 mg, 1.0 mmol) and benzylamine (**8d**, 164 mg, 1.5 mmol) in DMF (2 mL) under N_2_. The mixture was stirred for 1 h at 23 °C and then for 12 h at 50 °C. Analysis of the mixture by TLC indicated that no substitution had occurred. Substitution was noted under these conditions, however, for fluoroaromatic rings activated by a C4 NO_2_ (complete conversion) and CN (modest conversion).

### 3.5. Control Experiment Capture of Intermediate (Z)-2-((benzylamino)methyl)-3-(2-fluoro-5-nitrophenyl)acrylonitrile (**16**)

A 50 mL, round-bottomed flask equipped with a condenser and a stir bar was charged with 2-cyano-1-(2-fluoro-5-nitrophenyl)allyl acetate (**2**, 264 mg, 1.0 mmol) in DMF (2 mL) under N_2_. Benzylamine (**8d**, 164 mg, 1.5 mmol) was added at 23 °C with continued stirring. After 30 min, the solution was poured into de-ionized water (15 mL), and the mixture was extracted with EtOAc (3 × 15 mL). The combined organic layers were washed with saturated aq. NaCl and dried (Na_2_SO_4_). Removal of the solvent under vacuum gave the crude product, which was purified by silica gel column chromatography (10% EtOAc in hexane) to afford **10d** (band 1, 46 mg, 11%) as a yellow solid and (*Z*)-2-((benzylamino)methyl)-3-(2-fluoro-5-nitrophenyl)acrylonitrile (**16**, band 2, 236 mg, 76%) as a light yellow solid, m.p. 51–52 °C; IR: 3344, 2216, 1623, 1530, 1351 cm^−1^; ^1^H NMR (400 MHz, CDCl_3_): δ 8.92 (dd, *J* = 6.3, 2.8 Hz, 1H), 8.30 (ddd, *J* = 9.1, 4.5, 2.8 Hz, 1H), 7.37–7.34 (complex, 4H), 7.33 (br s, 1H), 7.29 (t, *J* = 9.1 Hz, 1H), 7.29 (obscured, 1H), 3.87 (s, 2H), 3.65 (d, *J* = 1.6 Hz, 2H), 1.70 (br s, 1H); ^13^C NMR (101 MHz, CDCl_3_): δ 163.3 (d, *J* = 262.1 Hz), 144.4, 139.1, 133.1 (d, *J* = 5.1 Hz), 128.6, 128.2, 127.5, 127.0 (d, *J* = 10.5 Hz), 124.7 (d, *J* = 4.1 Hz), 122.9 (d, *J* = 14.7 Hz), 117.2, 117.1 (d, *J* = 21.2 Hz), 116.8 (d, *J* = 10.0 Hz), 52.5, 52.3; MS: *m*/*z* 311 [M]^+·^. Anal. Calcd for C_17_H_14_FN_3_O_2_: C, 65.59; H, 4.53; N, 13.50. Found: C, 65.51; H, 4.54; N, 13.38. The X-ray structure of **16** (CCDC 2035028) was obtained and the thermal ellipsoid plot is shown in Figure 4 and the SI. Further heating of **16** (200 mg, 0.64 mmol) at 50 °C in DMF (2 mL) for 1 h afforded **10d** (112 mg, 60% unoptimized).

### 3.6. Representative Procedure for Preparation of Fluorodihydroquinolines Using MBH Acetates and Primary Amines

A 50 mL, round-bottomed flask equipped with a condenser, stir bar and vacuum adapter, was charged with MBH acetate **7** (1 mmol) in DMF (2 mL) under N_2_. The amine (1.5 mmol) was added in DMF (2 mL). The reaction was stirred at 23 °C for 1 h and gradually increased to 90 °C for 6 h. After TLC analysis (30% EtOAc/hexane) indicated completion of reaction, the solution was poured into de-ionized water (15 mL), and the mixture was extracted with EtOAc (3 × 10 mL). The combined organic layers were washed with saturated NaCl and dried (Na_2_SO_4_). Removal of the solvent under vacuum gave the crude product, which was purified by silica gel column chromatography (10% EtOAc in hexane) to afford the pure fluorodihydroquinoline analogs.

#### 3.6.1. Ethyl-6-fluoro-1-methyl-1,2-dihydroquinoline-3-carboxylate (**18a**) from **7** and **8a**

Yield: 214 mg (0.91 mmol, 91%) as a light yellow solid, m.p. 61–62 °C; IR: 1690, 1639, 1236 cm^−1^; ^1^H NMR (400 MHz, CDCl_3_): δ 7.21 (s, 1H), 6.84–6.75 (complex, 2H), 6.66 (dd, *J* = 8.8, 4.7 Hz, 1H), 4.19 (q, *J* = 7.1 Hz, 2H), 3.75 (s, 2H), 3.20 (s, 3H), 1.29 (t, *J* = 7.1 Hz, 3H); ^13^C NMR (100 MHz, CDCl_3_): δ 167.7, 158.8 (d, *J* = 242.0 Hz), 142.9, 135.3 (d, *J* = 2.3 Hz), 125.3 (d, *J* = 7.4 Hz), 116.2 (d, *J* = 22.5 Hz), 113.5 (d, *J* = 8.1 Hz), 113.3 (d, *J* = 22.4 Hz), 96.1, 59.6, 39.1, 26.6, 14.6; MS (EI): *m*/*z* 235 [M]^+·^; Anal. Calcd for C_13_H_14_FNO_2_: C, 66.37; H, 6.00; N, 5.95. Found: C, 66.40; H, 5.96; N, 5.87.

#### 3.6.2. Ethyl-6-fluoro-1-hexyl-1,2-dihydroquinoline-3-carboxylate (**18b**) from **7** and **8b**

Yield: 244 mg (0.80 mmol, 80%) as a yellow oil; IR: 1688, 1637, 1237 cm^−1^; ^1^H NMR (400 MHz, CDCl_3_): δ 7.22 (s, 1H), 6.68 (apparent d, J = 8.1 Hz, 2H), 6.67 (dd, *J* = 8.9, 4.8 Hz, 1H), 4.20 (q, *J* = 7.1 Hz, 2H), 3.75 (s, 2H), 3.51 (t, *J* = 7.4 Hz, 2H), 1.66 (quintet, *J* = 7.4 Hz, 2H), 1.39–1.26 (complex, 6H), 1.30 (t, J = 7.1 Hz, 3H), 0.89 (t, *J* = 6.6 Hz, 3H); ^13^C NMR (100 MHz, CDCl_3_): δ 167.8, 158.6 (d, *J* = 242.0 Hz), 142.3, 134.1 (d, *J* = 2.5 Hz), 125.6 (d, *J* = 7.2 Hz), 116.5 (d, *J* = 22.3 Hz), 113.8 (d, *J* = 8.1 Hz), 113.3 (d, *J* = 22.4 Hz), 95.5, 59.6, 51.6, 31.5, 27.8, 26.7, 26.4, 22.6, 14.6, 14.0; MS (EI): *m*/*z* 305 [M]^+·^; Anal. Calcd for C_18_H_24_FNO_2_: C, 70.79; H, 7.92; N, 4.59. Found: C, 70.72; H, 7.95; N, 4.49.

#### 3.6.3. Ethyl-6-fluoro-1-isobutyl-1,2-dihydroquinoline-3-carboxylate (**18c**) from **7** and **8c**

Yield: 244 mg (0.88 mmol, 88%) as a yellow oil; IR: 1688, 1639, 1234 cm^−1^; ^1^H NMR (400 MHz, CDCl_3_): δ 7.19 (s, 1H), 6.82–6.74 (complex, 2H), 6.65 (dd, *J* = 9.0, 4.7 Hz, 1H), 4.19 (q, *J* = 7.1 Hz, 2H), 3.75 (s, 2H), 3.30 (d, *J* = 7.3 Hz, 2H), 2.07 (nonet, *J* = 6.7 Hz, 1H), 1.29 (t, *J* = 7.1 Hz, 3H), 0.96 (d, *J* = 7.0 Hz, 6H); ^13^C NMR (100 MHz, CDCl_3_): δ 167.8, 158.6 (d, *J* = 242.1 Hz), 142.9, 134.3 (d, *J* = 2.5 Hz), 125.5 (d, *J* = 7.2 Hz), 116.4 (d, *J* = 22.1 Hz), 114.1 (d, *J* = 8.0 Hz), 113.3 (d, *J* = 22.4 Hz), 95.4, 59.6, 59.3, 26.7, 26.6, 20.0, 14.6; MS (EI): *m*/*z* 277 [M]^+·^; Anal. Calcd for C_16_H_20_FNO_2_: C, 69.29; H, 7.27; N, 5.05. Found: C, 69.18; H, 7.29; N, 4.98.

#### 3.6.4. Ethyl-1-benzyl-6-fluoro-1,2-dihydroquinoline-3-carboxylate (**18d**) from **7** and **8d**

Yield: 261 mg (0.84 mmol, 84%) as a light yellow solid, m.p. 57–58 °C; IR: 1686, 1643, 1242 cm^−1^; ^1^H NMR (400 MHz, CDCl_3_): δ 7.37 (s, 1H), 7.37–7.32 (complex, 2H), 7.30–7.22 (complex, 3H), 6.79 (dd, *J* = 8.8, 2.9 Hz, 1H), 6.64 (td, *J* = 8.4, 2.9 Hz, 1H), 6.52 (dd, *J* = 8.9, 4.7 Hz, 1H), 4.76 (s, 2H), 4.21 (q, *J* = 7.1 Hz, 2H), 3.83 (s, 2H), 1.30 (t, *J* = 7.1 Hz, 3H); ^13^C NMR (100 MHz, CDCl_3_): δ 167.7, 158.7 (d, *J* = 242.3 Hz), 142.8, 136.4, 134.2 (d, *J* = 2.4 Hz), 129.0, 127.7, 126.1, 125.4 (d, *J* = 7.4 Hz), 116.4 (d, *J* = 22.4 Hz), 114.9 (d, *J* = 8.2 Hz), 113.4 (d, *J* = 22.4 Hz), 96.9, 59.7, 55.3, 26.7, 14.6; MS (EI): *m*/*z* 311 [M]^+·^; Anal. Calcd for C_19_H_18_FNO_2_: C, 73.30; H, 5.83; N, 4.50. Found: C, 73.22; H, 5.78; N, 4.48.

#### 3.6.5. Ethyl-6-fluoro-1-phenethyl-1,2-dihydroquinoline-3-carboxylate (**18e**) from **7** and **8e**

Yield: 289 mg (0.89 mmol, 89%) as a yellow oil; IR: 1687, 1643, 1241 cm^−1^; ^1^H NMR (400 MHz, CDCl_3_): δ 7.32 (t, *J* = 7.5 Hz, 2H), 7.27–7.22 (complex, 1H), 7.19 (d, *J* = 7.5 Hz, 2H), 7.08 (s, 1H), 6.86–6.74 (complex, 3H), 4.16 (d, *J* = 7.1 Hz, 2H), 3.74 (t, *J* = 7.4 Hz, 2H), 3.73 (s, 2H), 2.94 (t, *J* = 7.0 Hz, 2H), 1.27 (t, *J* = 7.1 Hz, 3H); ^13^C NMR (100 MHz, CDCl_3_): δ 167.7, 158.7 (d, *J* = 242.2 Hz), 142.0, 137.9, 133.9 (d, *J* = 2.5 Hz), 128.81, 128.75, 126.9, 125.6 (d, *J* = 7.3 Hz), 116.7 (d, *J* = 21.8 Hz), 113.7 (d, *J* = 8.1 Hz), 113.5 (d, *J* = 22.4 Hz), 96.4, 59.6, 52.9, 34.3, 26.6, 14.6; MS (EI): *m*/*z* 325 [M]^+·^; Anal. Calcd for C_20_H_20_FNO_2_: C, 73.83; H, 6.20; N, 4.30. Found: C, 73.80; H, 6.22; N, 4.33.

### 3.7. Representative Procedure for Synthesis of Fluoroquinolones

Prior to the reaction, the anhydrous DMF solvent was treated with dry O_2_, introduced via a gas dispersion tube, for 15 s. A 50 mL, round-bottomed flask equipped with a condenser, stir bar and vacuum adapter, was charged with MBH acetate **7** (1 mmol) in DMF/O_2_ (2 mL) under N_2_. The (1.5 mmol) amine was added in DMF/O_2_ (2 mL). The reaction was stirred at 23 °C for 1 h and then at 90 °C for 6 h. After TLC analysis (30% EtOAc/hexane) indicated completion of the reaction, the solution was poured into de-ionized water (15 mL), and the mixture was extracted with EtOAc (3 × 10 mL). The combined organic layers were washed with saturated NaCl and dried (Na_2_SO_4_). Removal of the solvent under vacuum gave the crude product, which was purified by trituration with 5% pentane in ether to afford the fluoroquinolone derivatives.

#### 3.7.1. Ethyl-6-fluoro-1-methyl-4-oxo-1,4-dihydroquinoline-3-carboxylate (**19a**) from **7** and **8a**

Yield: 212 mg (0.85 mmol, 85%) as a light tan solid, m.p. 203–204 °C; IR: 1728, 1694 cm^−1^; ^1^H NMR (400 MHz, CDCl_3_): δ 8.46 (s, 1H), 8.18 (d, *J* = 8.9 Hz, 1H), 7.44 (d, *J* = 5.7 Hz, 2H), 4.40 (q, *J* = 7.1 Hz, 2H), 3.90 (s, 3H), 1.42 (t, *J* = 7.1 Hz, 3H); ^13^C NMR (100 MHz, CDCl_3_): δ 173.5 (d, *J* = 2.0 Hz), 165.7, 160.1 (d, *J* = 232.0 Hz), 149.6, 136.3 (d, *J* = 6.6 Hz), 130.8 (d, *J* = 6.8 Hz), 121.0 (d, *J* = 24.7 Hz), 117.8 (d, *J* = 7.7 Hz), 113.0 (d, *J* = 23.2 Hz), 110.4, 61.6, 41.6, 14.4; MS (EI): *m*/*z* 249; Anal. Calcd for C_13_H_12_FNO_3_: C, 62.65; H, 4.85; N, 5.62. Found: C, 62.74; H, 4.79; N, 5.56.

#### 3.7.2. Ethyl-6-fluoro-1-isobutyl-4-oxo-1,4-dihydroquinoline-3-carboxylate (**19c**) from **7** and **8c**

Yield: 250 mg (0.86 mmol, 86%) as a light tan solid, m.p. 150–151 °C; IR: 1727, 1695 cm^−1^; ^1^H NMR (400 MHz, CDCl_3_): δ 8.43 (s, 1H), 8.21 (m, 1H), 7.42 (m, 2H), 4.41 (q, *J* = 7.1 Hz, 2H), 3.97 (d, *J* = 7.5 Hz, 2H), 2.26 (nonet, *J* = 7.0 Hz, 1H), 1.42 (t, *J* = 7.1 Hz, 3H), 1.02 (d, *J* = 6.6 Hz, 6H); ^13^C NMR (100 MHz, CDCl_3_): δ 173.4 (d, *J* = 2.4 Hz), 165.9, 159.9 (d, *J* = 247.8 Hz), 149.4, 135.4 (d, *J* = 1.9 Hz), 131.2 (d, *J* = 6.8 Hz), 120.8 (d, *J* = 28.1 Hz), 118.1 (d, *J* = 7.8 Hz), 113.1 (d, *J* = 23.0 Hz), 109.9, 61.6, 61.0, 27.7, 19.9, 14.4; MS (EI): *m*/*z* 291; Anal. Calcd for C_16_H_18_FNO_3_: C, 65.97; H, 6.23; N, 4.81. Found: C, 66.01; H, 6.20; N, 4.76.

#### 3.7.3. Ethyl-1-benzyl-6-fluoro-4-oxo-1,4-dihydroquinoline-3-carboxylate (**19d**) from **7** and **8d**

Yield: 269 mg (0.83 mmol, 83%) as a light tan solid, m.p. 169–170 °C; IR: 1727, 1691, 1619 cm^−1^; ^1^H NMR (400 MHz, CDCl_3_): δ 8.60 (s, 1H), 8.18 (dd, *J* = 8.9, 2.9 Hz, 1H), 7.41–7.24 (complex, 5H), 7.16 (d, *J* = 6.3 Hz, 2H), 5.40 (s, 2H), 4.41 (q, *J* = 7.1 Hz, 2H), 1.42 (t, *J* = 7.1 Hz, 3H); ^13^C NMR (100 MHz, CDCl_3_): δ 173.5 (d, *J* = 2.3 Hz), 165.8, 160.0 (d, *J* = 248.0 Hz), 149.6, 135.6 (d, *J* = 1.8 Hz), 133.9, 131.2 (d, *J* = 7.0 Hz), 129.5, 128.8, 126.0, 121.0 (d, *J* = 25.3 Hz), 118.9 (d, *J* = 7.8 Hz), 113.0 (d, *J* = 23.0 Hz), 110.6, 61.1, 57.8, 14.4; MS (EI): *m*/*z* 325; Anal. Calcd for C_19_H_16_FNO_3_: C, 70.14; H, 4.96; N, 4.31. Found: C, 70.04; H, 4.92; N, 4.23.

#### 3.7.4. Ethyl-6-fluoro-4-oxo-1-phenethyl-1,4-dihydroquinoline-3-carboxylate (**19e**) from **7** and **8e**

Yield: 284 mg (0.84 mmol, 84%) as a light tan solid, m.p. 151–152 °C; IR: 1736, 1691, 1618 cm^−1^; ^1^H NMR (400 MHz, CDCl_3_): δ 8.21 (dd, *J* = 8.9, 3.0 Hz, 1H), 8.14 (s, 1H), 7.50 (dd, *J* = 9.2, 4.1 Hz, 1H), 7.44 (m, 1H), 7.33–7.23 (complex, 3H), 7.06 (d, *J* = 7.5 Hz, 2H), 4.40 (t, *J* = 7.1 Hz, 2H), 4.34 (q, *J* = 7.1 Hz, 2H), 3.16 (t, *J* = 7.1 Hz, 2H), 1.36 (t, *J* = 7.1 Hz, 3H); ^13^C NMR (100 MHz, CDCl_3_): δ 173.3 (d, *J* = 2.3 Hz), 165.4, 159.9 (d, *J* = 247.9 Hz), 149.0, 136.1, 135.1 (d, *J* = 1.8 Hz), 131.2 (d, *J* = 6.8 Hz), 129.1, 128.7, 127.6, 121.0 (d, *J* = 25.2 Hz), 117.7 (d, *J* = 7.7 Hz), 113.3 (d, *J* = 23.0 Hz), 110.1, 60.9, 55.4, 35.2, 14.4; MS (EI): *m*/*z* 339; Anal. Calcd for C_20_H_18_FNO_3_: C, 70.78; H, 5.35; N, 4.13. Found: C, 70.69; H, 5.33; N, 4.08. The X-ray structure for **19e** (CCDC 2057847) and the thermal ellipsoid plot appears in Figure 5 and the SI.

## 4. Conclusions

We have investigated the synthesis of dihydroquinolines and dihydronaphthyridines from Morita-Baylis-Hillman acetates and primary amines in DMF. The formation of dihydroquinolines occurs at 50 °C while dihydronaphthyridines required heating to 90 °C. Substrates for the dihydroquinolines were MBH acetates bearing 2-fluorophenyl rings activated toward S_N_Ar ring closure by a C5 nitro or cyano group. Dihydronaphthyridine precursors were activated only by the electron-withdrawing nitrogen in a 2-fluoropyridine moiety, and were thus less reactive in the final cyclization. The transformation most likely involves a domino S_N_2′-S_N_Ar process. An initial S_N_2′ mechanism yields the alkene having the nitrogen nucleophile predominantly trans to the S_N_Ar acceptor ring. Under the reaction conditions, a reversible Michael addition by the excess amine or an internal addition–elimination process can equilibrate the double bond geometry to yield the alkene isomer needed for ring closure. The isolation of intermediates from the reaction disputes several earlier reports that assumed the reaction directly generated the alkene needed for cyclization. Once equilibrated, subsequent S_N_Ar ring closure by the nitrogen nucleophile and deprotonation affords the product. Much of this selectivity appears to be guided by steric factors. Two dihydronaphthyridines derived from precursors incorporating a side chain acrylonitrile inexplicably underwent migration of the C3–C4 double bond to the C2–C3 position to afford an enaminonitrile. We have extended our study to a difluorophenyl MBH acetate and found divergent reactivity, depending upon the presence or absence of O_2_ in the DMF solvent. Under anaerobic conditions at 90 °C, these substrates afforded the expected dihydroquinolines while aerobic conditions led to the formation of fluoroquinolones. The difluoro MBH derivatives represent a new approach to fluoroquinolones affording yet another valuable ring system that can be generated from these versatile precursors. Good to excellent yields (72–93%) were isolated for all examples.

## Figures and Tables

**Table 1:**
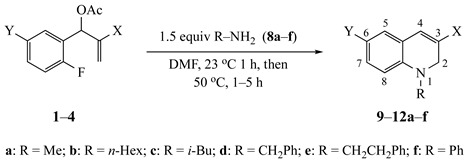
Synthesis of 3,6-disubstituted 1-alkyl/phenyl-1,2-dihydroquinolines.

Substrate	Amine	R ^a^	X	Y	Product (%)
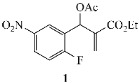	**8a**	CH_3_	CO_2_Et	NO_2_	**9a** (93)
	**8b**	*n*-Hex	CO_2_Et	NO_2_	**9b** (87)
	**8c**	*i*-Bu	CO_2_Et	NO_2_	**9c** (93)
	**8d**	CH_2_Ph	CO_2_Et	NO_2_	**9d** (92)
	**8e**	CH_2_CH_2_Ph	CO_2_Et	NO_2_	**9e** (83)
	**8f**	Ph	CO_2_Et	NO_2_	**9f** (89)
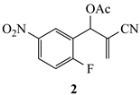	**8a**	CH_3_	CN	NO_2_	**10a** (88)
	**8b**	*n*-Hex	CN	NO_2_	**10b** (78)
	**8c**	*i*-Bu	CN	NO_2_	**10c** (79)
	**8d**	CH_2_Ph	CN	NO_2_	**10d** (80)
	**8e**	CH_2_CH_2_Ph	CN	NO_2_	10e (82)
	**8f**	Ph	CN	NO_2_	**10f** (81)
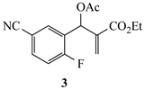	**8a**	CH_3_	CO_2_Et	CN	**11a** (91)
	**8b**	*n*-Hex	CO_2_Et	CN	**11b** (82)
	**8c**	*i*-Bu	CO_2_Et	CN	**11c** (85)
	**8d**	CH_2_Ph	CO_2_Et	CN	**11d** (79)
	**8e**	CH_2_CH_2_Ph	CO_2_Et	CN	**11e** (85)
	**8f**	Ph	CO_2_Et	CN	**11f** (83)
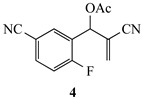	**8a**	CH_3_	CN	CN	**12a** (82)
	**8b**	*n*-Hex	CN	CN	**12b** (77)
	**8c**	*i*-Bu	CN	CN	**12c** (81)
	**8d**	CH_2_Ph	CN	CN	**12d** (86)
	**8e**	CH_2_CH_2_Ph	CN	CN	**12e** (88)

^a^ R = alkyl/phenyl group of a primary amine.

**Table 2:**
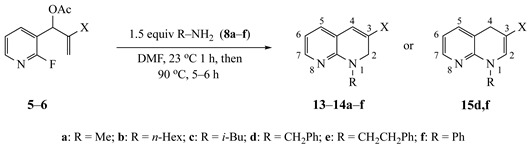
Synthesis of 3-substituted 1-alkyl/phenyl-1,2 and 1,4-dihydronaphthyridines.

Substrate	Amine	R ^a^	X	Product (%)
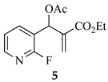	**8a**	CH_3_	CO_2_Et	**13a** (88)
	**8b**	*n*-Hex	CO_2_Et	**13b** (80)
	**8c**	*i*-Bu	CO_2_Et	**13c** (80)
	**8d**	CH_2_Ph	CO_2_Et	**13d** (81)
	**8e**	CH_2_CH_2_Ph	CO_2_Et	**13e** (83)
	**8f**	Ph	CO_2_Et	**13f** (77)
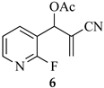	**8b**	*n*-Hex	CN	**14b** (82)
	**8d**	CH_2_Ph	CN	**14d** (6) + **15d** (82)
	**8e**	CH_2_CH_2_Ph	CN	**14e** (80)
	**8f**	Ph	CN	**15f** (72) ^b^

^a^ R = alkyl/phenyl group of a primary amine. ^b^ The double bond-migrated product **15f** was the only product.

**Table 3:**
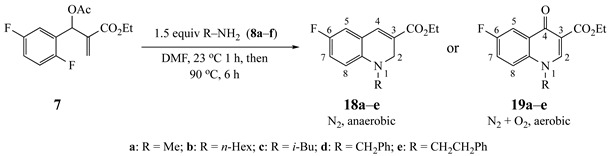
Synthesis of ethyl 1-alkyl-1,2 and 1,4-dihydronaphthyridine-3-carboxylates.

Substrate	Amine	R ^a^	Product (%)
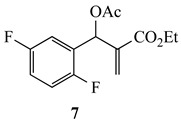	**8a**	CH_3_	**18a** (91)
	**8b**	*n*-Hex	**18b** (80)
	**8c**	*i*-Bu	**18c** (88)
	**8d**	CH_2_Ph	**18d** (84)
	**8e**	CH_2_CH_2_Ph	**18e** (89)
	**8a**	CH_3_	**19a** (85)
	**8b**	*n*-Hex	^b^
	**8c**	*i*-Bu	**19c** (86)
	**8d**	CH_2_Ph	**19d** (83)
	**8e**	CH_2_CH_2_Ph	**19e** (84)

^a^ R = alkyl/phenyl group of a primary amine. ^b^ An inseparable mixture of dihydroquinoline and quinolone was isolated.

## Data Availability

Copies of ^1^H and ^13^C NMR spectra for **6**, **7**, **9a–f**, **10a–f**, **11a–f**, **12a–e**, **13a–f**, **14b**, **14d,e**, **15d**, **15f**, **16**, **18a–e** and **19a–e** compounds are given in the SI. Data pertaining to the X-ray crystal structures for **15d**, **16** and **19e** are also included in the SI.

## Supplementary Materials

The following are available online: copies of ^1^H NMR and ^13^C NMR spectra for **6**, **7**, **9a–f**, **10a–f**, **11a–f**, **12a–e**, **13a–f**, **14b**, **14d,e**, **15d**, **15f**, **16**, **18a–e** and **19a–e** and tables of crystal data for compounds **15d**, **16** and **19e**.
