# Supplementary material for: Dihydroquinolines, Dihydronaphthyridines and Quinolones by Domino Reactions of Morita-Baylis-Hillman Acetates
Source: Molecules. 2021 Feb 8;26(4):890. doi: 10.3390/molecules26040890 (PMC7914960; doi:10.3390/molecules26040890)
Supplement: Supplementary file 1 [file molecules-26-00890-s001.pdf]

Dihydroquinolines, Dihydronaphthyridines and Quinolones by Domino Reactions of Morita-Baylis-Hillman Acetates

Joel K. Annor-Gyamfi, Eben Ametsetor, Kevin Meraz and Richard A. Bunce*

Department of Chemistry, Oklahoma State University, Stillwater, OK. 74078-3071, USA
jannorg@okstate.edu (J.K.A.), eametse@okstate.edu (E.A.), meraz.kevin@outlook.com (K.M.)

* Author to whom correspondence should be addressed; E-mail: rab@okstate.edu;
Tel.: +1-405-744-5952

Supplementary Material

Compound **page**

^{1}H NMR for 2-Cyano-1-(2-fluoropyridin-3-yl)allyl acetate (**6**) 4
^{13}C NMR for 2-Cyano-1-(2-fluoropyridin-3-yl)allyl acetate (**6**) 4
^{1}H NMR for Ethyl 2-(acetoxy(2,5-difluorophenyl)methyl)acrylate (**7**).... 5
^{13}C NMR for Ethyl 2-(acetoxy(2,5-difluorophenyl)methyl)acrylate (**7**).... 5
^{1}H NMR for Ethyl 1-methyl-6-nitro-1,2-dihydroquinoline-3-carboxylate (**9a**) 6
^{13}C NMR for Ethyl 1-methyl-6-nitro-1,2-dihydroquinoline-3-carboxylate (**9a**) 6
^{1}H NMR for Ethyl 1-hexyl-6-nitro-1,2-dihydroquinoline-3-carboxylate (**9b**) 7
^{13}C NMR for Ethyl 1-hexyl-6-nitro-1,2-dihydroquinoline-3-carboxylate (**9b**) 7
^{1}H NMR for Ethyl 1-isobutyl-6-nitro-1,2-dihydroquinoline-3-carboxylate (**9c**) 8
^{13}C NMR for Ethyl 1-isobutyl-6-nitro-1,2-dihydroquinoline-3-carboxylate (**9c**) 8
^{1}H NMR for Ethyl 1-benzyl-6-nitro-1,2-dihydroquinoline-3-carboxylate (**9d**) 9
^{13}C NMR for Ethyl 1-benzyl-6-nitro-1,2-dihydroquinoline-3-carboxylate (**9d**) 9
^{1}H NMR for Ethyl 6-nitro-1-phenethyl-1,2-dihydroquinoline-3-carboxylate (**9e**) 10
^{13}C NMR for Ethyl 6-nitro-1-phenethyl-1,2-dihydroquinoline-3-carboxylate (**9e**) 10
^{1}H NMR for Ethyl 6-nitro-1-phenyl-1,2-dihydroquinoline-3-carboxylate (**9f**) 11
^{13}C NMR for Ethyl 6-nitro-1-phenyl-1,2-dihydroquinoline-3-carboxylate (**9f**) 11
^{1}H NMR for 1-Methyl-6-nitro-1,2-dihydroquinoline-3-carbonitrile (**10a**) 12
^{13}C NMR for 1-Methyl-6-nitro-1,2-dihydroquinoline-3-carbonitrile (**10a**) 12
^{1}H NMR for 1-Hexyl-6-nitro-1,2-dihydroquinoline-3-carbonitrile (**10b**) 13
^{13}C NMR for 1-Hexyl-6-nitro-1,2-dihydroquinoline-3-carbonitrile (**10b**) 13
^{1}H NMR for 1-Isobutyl-6-nitro-1,2-dihydroquinoline-3-carbonitrile (**10c**) 14
^{13}C NMR for 1-Isobutyl-6-nitro-1,2-dihydroquinoline-3-carbonitrile (**10c**) 14
^{1}H NMR for 1-Benzyl-6-nitro-1,2-dihydroquinoline-3-carbonitrile (**10d**) 15
^{13}C NMR for 1-Benzyl-6-nitro-1,2-dihydroquinoline-3-carbonitrile (**10d**) 15
^{1}H NMR for 6-Nitro-1-phenethyl-1,2-dihydroquinoline-3-carbonitrile (**10e**) 16

^{13}C NMR for 6-Nitro-1-phenethyl-1,2-dihydroquinoline-3-carbonitrile (**10e**) 16
^{1}H NMR for 6-Nitro-1-phenyl-1,2-dihydroquinoline-3-carbonitrile (**10f**) 17
^{13}C NMR for 6-Nitro-1-phenyl-1,2-dihydroquinoline-3-carbonitrile (**10f**) 17
^{1}H NMR for Ethyl 6-cyano-1-methyl-1,2-dihydroquinoline-3-carboxylate (**11a**) 18
^{13}C NMR for Ethyl 6-cyano-1-methyl-1,2-dihydroquinoline-3-carboxylate (**11a**) 18
^{1}H NMR for Ethyl 6-cyano-1-hexyl-1,2-dihydroquinoline-3-carboxylate (**11b**) 19
^{13}C NMR for Ethyl 6-cyano-1-hexyl-1,2-dihydroquinoline-3-carboxylate (**11b**) 19
^{1}H NMR for Ethyl 6-cyano-1-isobutyl-1,2-dihydroquinoline-3-carboxylate (**11c**) 20
^{13}C NMR for Ethyl 6-cyano-1-isobutyl-1,2-dihydroquinoline-3-carboxylate (**11c**) 20
^{1}H NMR for Ethyl 1-benzyl-6-cyano-1,2-dihydroquinoline-3-carboxylate (**11d**) 21
^{13}C NMR for Ethyl 1-benzyl-6-cyano-1,2-dihydroquinoline-3-carboxylate (**11d**) 21
^{1}H NMR for Ethyl 6-cyano-1-phenethyl-1,2-dihydroquinoline-3-carboxylate (**11e**) 22
^{13}C NMR for Ethyl 6-cyano-1-phenethyl-1,2-dihydroquinoline-3-carboxylate (**11e**) 22
^{1}H NMR for Ethyl 6-cyano-1-phenyl-1,2-dihydroquinoline-3-carboxylate (**11f**) 23
^{13}C NMR for Ethyl 6-cyano-1-phenyl-1,2-dihydroquinoline-3-carboxylate (**11f**) 23
^{1}H NMR for 1-Methyl-1,2-dihydroquinoline-3,6-dicarbonitrile (**12a**) 24
^{13}C NMR for 1-Methyl-1,2-dihydroquinoline-3,6-dicarbonitrile (**12a**) 24
^{1}H NMR for 1-Hexyl-1,2-dihydroquinoline-3,6-dicarbonitrile (**12b**) 25
^{13}C NMR for 1-Hexyl-1,2-dihydroquinoline-3,6-dicarbonitrile (**12b**) 25
^{1}H NMR for 1-Isobutyl-1,2-dihydroquinoline-3,6-dicarbonitrile (**12c**) 26
^{13}C NMR for 1-Isobutyl-1,2-dihydroquinoline-3,6-dicarbonitrile (**12c**) 26
^{1}H NMR for 1-Benzyl-1,2-dihydroquinoline-3,6-dicarbonitrile (**12d**) 27
^{13}C NMR for 1-Benzyl-1,2-dihydroquinoline-3,6-dicarbonitrile (**12d**) 27
^{1}H NMR for 1-Phenethyl-1,2-dihydroquinoline-3,6-dicarbonitrile (**12e**) 28
^{13}C NMR for 1-Phenethyl-1,2-dihydroquinoline-3,6-dicarbonitrile (**12e**) 28
^{1}H NMR for Ethyl 1-methyl-1,2-dihydro-1,8-naphthyridine-3-carboxylate (**13a**) 29
^{13}C NMR for Ethyl 1-methyl-1,2-dihydro-1,8-naphthyridine-3-carboxylate (**13a**) 29
^{1}H NMR for Ethyl 1-hexyl-1,2-dihydro-1,8-naphthyridine-3-carboxylate (**13b**) 30
^{13}C NMR for Ethyl 1-hexyl-1,2-dihydro-1,8-naphthyridine-3-carboxylate (**13b**) 30
^{1}H NMR for Ethyl 1-isobutyl-1,2-dihydro-1,8-naphthyridine-3-carboxylate (**13c**) 31
^{13}C NMR for Ethyl 1-isobutyl-1,2-dihydro-1,8-naphthyridine-3-carboxylate (**13c**) 31
^{1}H NMR for Ethyl 1-benzyl-1,2-dihydro-1,8-naphthyridine-3-carboxylate (**13d**) 32
^{13}C NMR for Ethyl 1-benzyl-1,2-dihydro-1,8-naphthyridine-3-carboxylate (**13d**) 32
^{1}H NMR for Ethyl 1-phenethyl-1,2-dihydro-1,8-naphthyridine-3-carboxylate (**13e**) 33
^{13}C NMR for Ethyl 1-phenethyl-1,2-dihydro-1,8-naphthyridine-3-carboxylate (**13e**) 33
^{1}H NMR for Ethyl 1-phenyl-1,2-dihydro-1,8-naphthyridine-3-carboxylate (**13f**) 34
^{13}C NMR for Ethyl 1-phenyl-1,2-dihydro-1,8-naphthyridine-3-carboxylate (**13f**) 34
^{1}H NMR for 1-Hexyl-1,2-dihydro-1,8-naphthyridine-3-carbonitrile (**14b**) 35
^{13}C NMR for 1-Hexyl-1,2-dihydro-1,8-naphthyridine-3-carbonitrile (**14b**) 35
^{1}H NMR for 1-Benzyl-1,2-dihydro-1,8-naphthyridine-3-carbonitrile (**14d**) 36
^{13}C NMR for 1-Benzyl-1,2-dihydro-1,8-naphthyridine-3-carbonitrile (**14d**) 36

^{1}H NMR for 1-Phenethyl-1,2-dihydro-1,8-naphthyridine-3-carbonitrile (**14e**) 37
^{13}C NMR for 1-Phenethyl-1,2-dihydro-1,8-naphthyridine-3-carbonitrile (**14e**) 37

Unexpected double bond-migrated products:
^{1}H NMR for 1-Benzyl-1,4-dihydroquinoline-3-carbonitrile (**15d**) 38
^{13}C NMR for 1-Benzyl-1,4-dihydroquinoline-3-carbonitrile (**15d**) 38
^{1}H NMR for 1-Phenyl-1,4-dihydroquinoline-3-carbonitrile (**15f**) 39
^{13}C NMR for 1-Phenyl-1,4-dihydroquinoline-3-carbonitrile (**15f**) 39

Control Reaction:
^{1}H for (Z)-2-((Benzylamino)methyl)-3-(2-fluoro-5-nitrophenyl)acrylonitrile (**16**) 40
^{13}C for (Z)-2-((Benzylamino)methyl)-3-(2-fluoro-5-nitrophenyl)acrylonitrile (**16**) 40

^{1}H NMR for ethyl 6-fluoro-1-methyl-1,2-dihydroquinoline-3-carboxylate (**18a**) 41
^{13}C NMR for ethyl 6-fluoro-1-methyl-1,2-dihydroquinoline-3-carboxylate (**18a**) 41
^{1}H NMR for ethyl 6-fluoro-1-hexyl-1,2-dihydroquinoline-3-carboxylate (**18b**) 42
^{13}C NMR for ethyl 6-fluoro-1-hexyl-1,2-dihydroquinoline-3-carboxylate (**18b**) 42
^{1}H NMR for ethyl 6-fluoro-1-isobutyl-1,2-dihydroquinoline-3-carboxylate (**18c**) 43
^{13}C NMR for ethyl 6-fluoro-1-isobutyl-1,2-dihydroquinoline-3-carboxylate (**18c**) 43
^{1}H NMR for ethyl 1-benzyl-6-fluoro-1,2-dihydroquinoline-3-carboxylate (**18d**) 44
^{13}C NMR for ethyl 1-benzyl-6-fluoro-1,2-dihydroquinoline-3-carboxylate (**18d**) 44
^{1}H NMR for ethyl 6-fluoro-1-phenethyl-1,2-dihydroquinoline-3-carboxylate (**18e**) 45
^{13}C NMR for ethyl 6-fluoro-1-phenethyl-1,2-dihydroquinoline-3-carboxylate (**18e**) 45
^{1}H NMR for ethyl 6-fluoro-1-methyl-4-oxo-1,4-dihydroquinoline-3-carboxylate (**19a**) 46
^{13}C NMR for ethyl 6-fluoro-1-methyl-4-oxo-1,4-dihydroquinoline-3-carboxylate (**19a**) 46
^{1}H NMR for ethyl 6-fluoro-1-isobutyl-4-oxo-1,4-dihydroquinoline-3-carboxylate (**19c**) 47
^{13}C NMR for ethyl 6-fluoro-1-isobutyl-4-oxo-1,4-dihydroquinoline-3-carboxylate (**19c**) 47
^{1}H NMR for ethyl 1-benzyl-6-fluoro-4-oxo-1,4-dihydroquinoline-3-carboxylate (**19d**) 48
^{13}C NMR for ethyl 1-benzyl-6-fluoro-4-oxo-1,4-dihydroquinoline-3-carboxylate (**19d**) 48
^{1}H NMR for ethyl 6-fluoro-4-oxo-1-phenethyl-1,4-dihydroquinoline-3-carboxylate (**19e**) 49
^{13}C NMR for ethyl 6-fluoro-4-oxo-1-phenethyl-1,4-dihydroquinoline-3-carboxylate (**19e**) 49

X-ray Data:
1-Benzyl-1,4-dihydroquinoline-3-carbonitrile (**15d**, EB-1- UNK-Bn, CCDC 2035027) 50-59
(Z)-2-((Benzylamino)methyl)-3-(2-fluoro-5-nitrophenyl)acrylonitrile (**16**, EB-1-INT-6, CCDC 2035028) 60-69
Ethyl 6-fluoro-4-oxo-1-phenethyl-1,4-dihydroquinoline-3-carboxylate (**19e**, KEVJAG-1-64, CCDC 2057847)... 70-79

^{1}H NMR for 2-Cyano-1-(2-fluoropyridin-3-yl)allyl acetate (**6**)

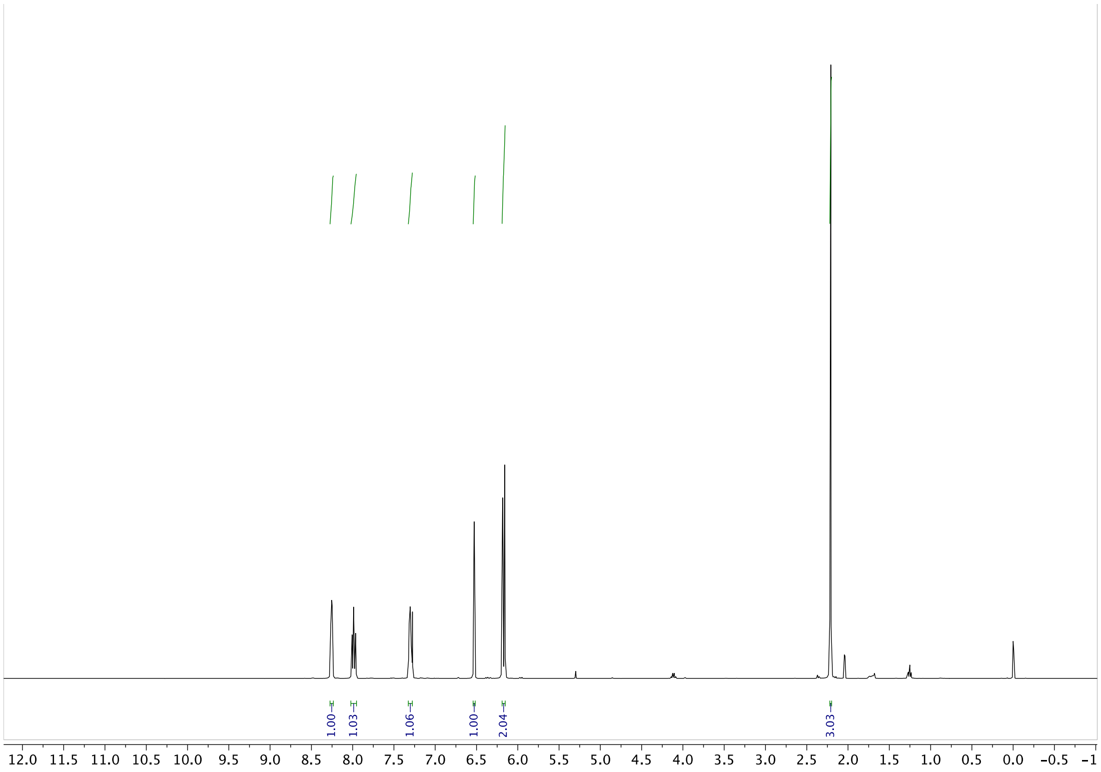

^{13}C NMR for *2-cyano-1-(2-fluoropyridin-3-yl)allyl acetate* (**6**)

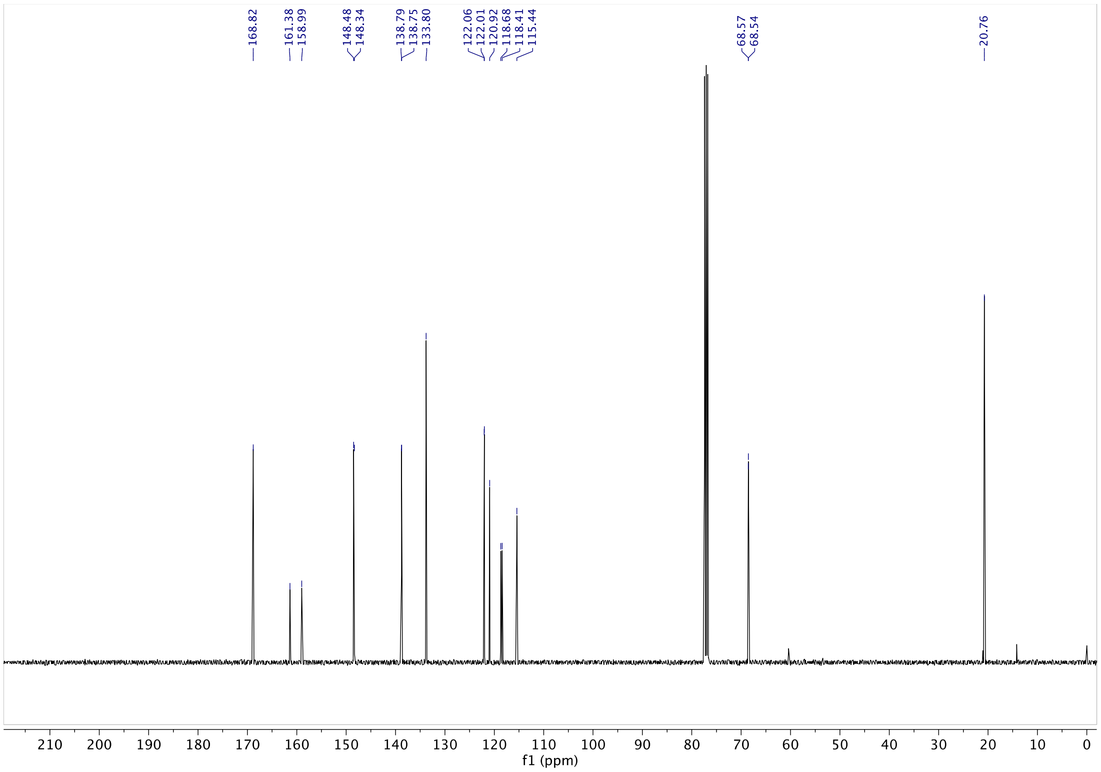

^{1}H NMR for ethyl 2-(acetoxy(2,5-difluorophenyl)methyl)acrylate (**7**)

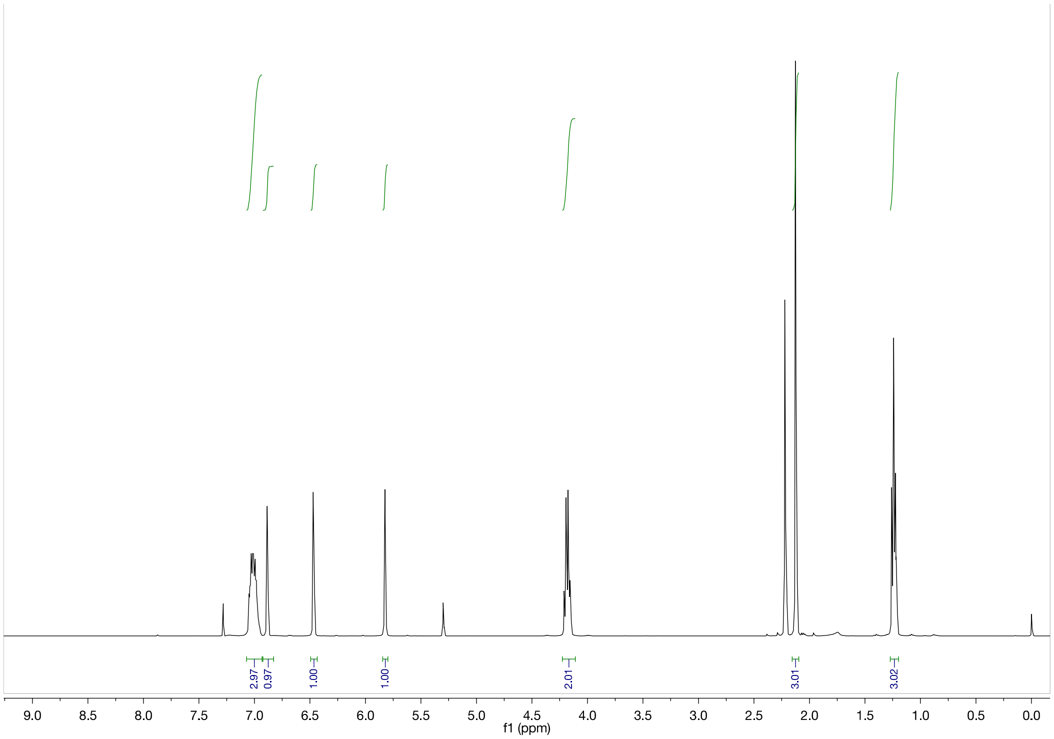

^{13}C NMR for ethyl 2-(acetoxy(2,5-difluorophenyl)methyl)acrylate (**7**)

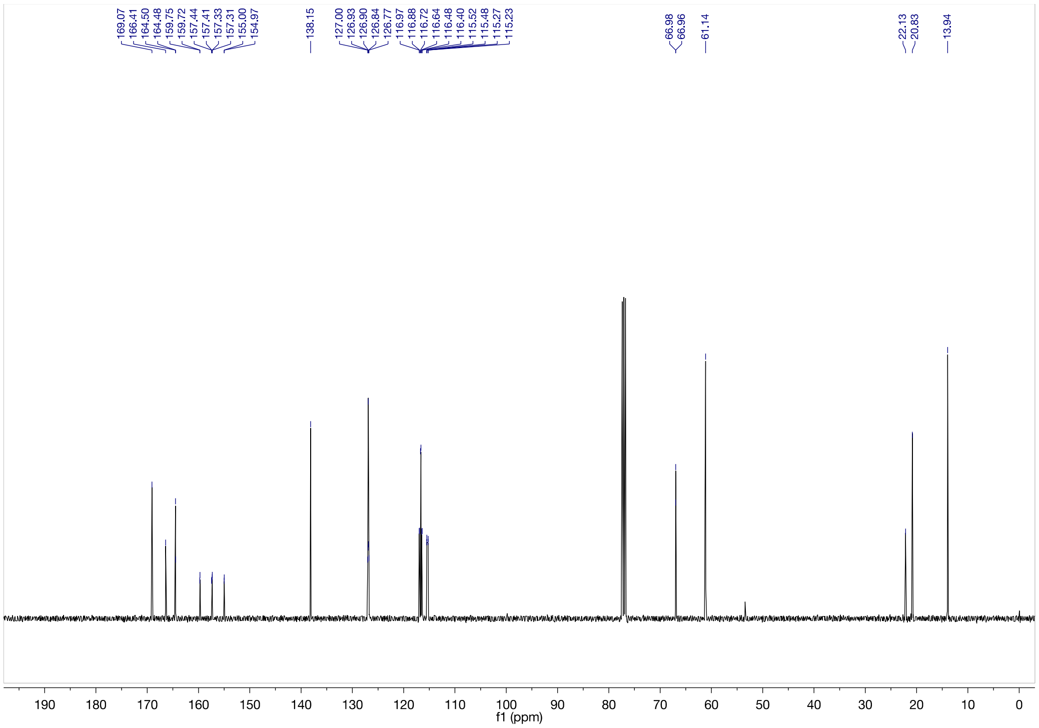

^{1}H NMR for Ethyl 1-methyl-6-nitro-1,2-dihydroquinoline-3-carboxylate (**9a**)

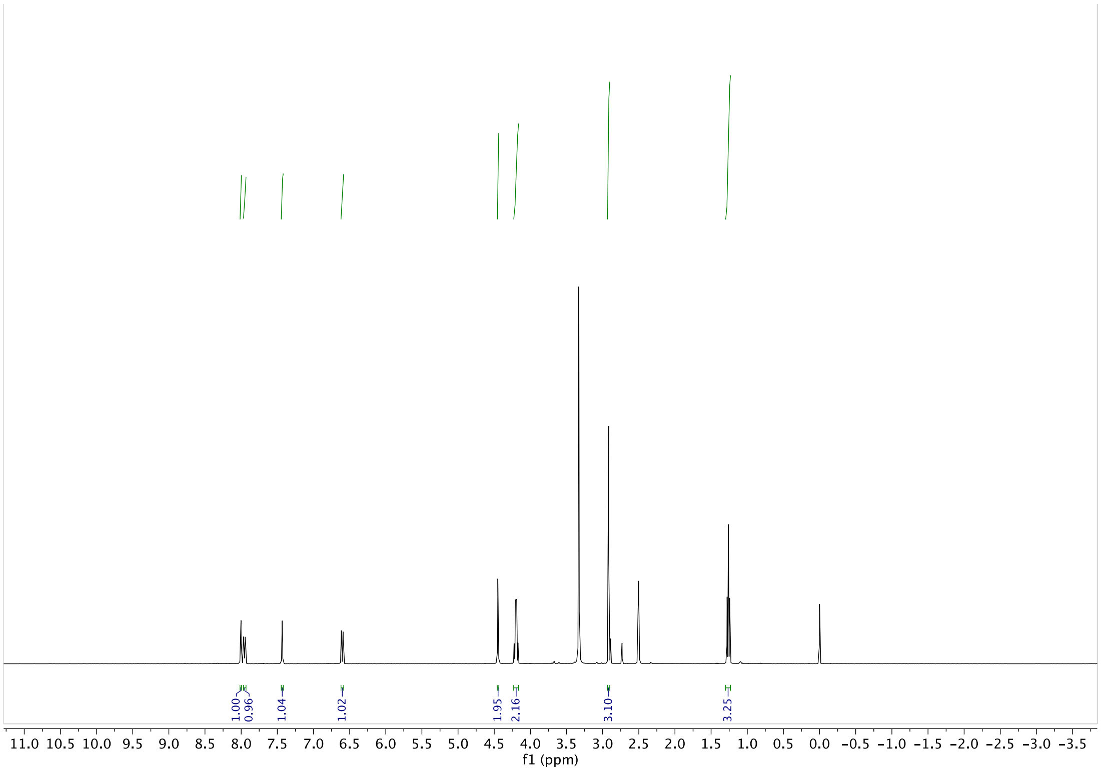

^{13}C NMR for Ethyl 1-methyl-6-nitro-1,2-dihydroquinoline-3-carboxylate (**9a**)

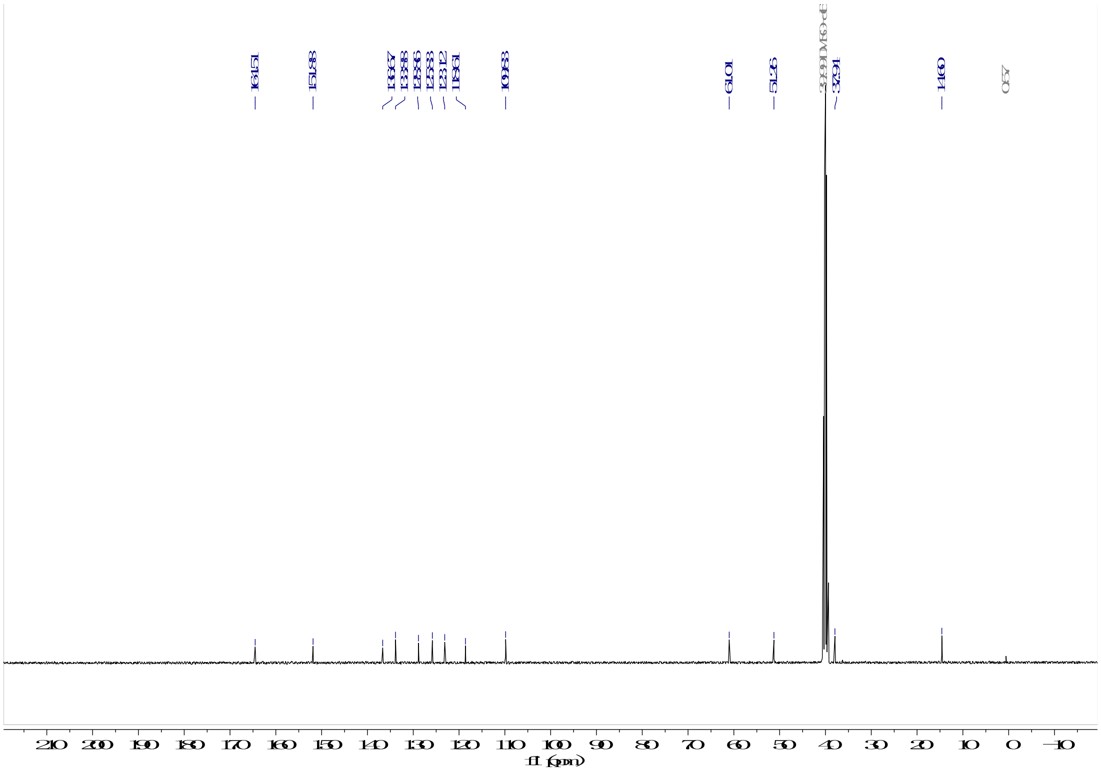

^{1}H NMR for Ethyl 1-hexyl-6-nitro-1,2-dihydroquinoline-3-carboxylate (**9b**)

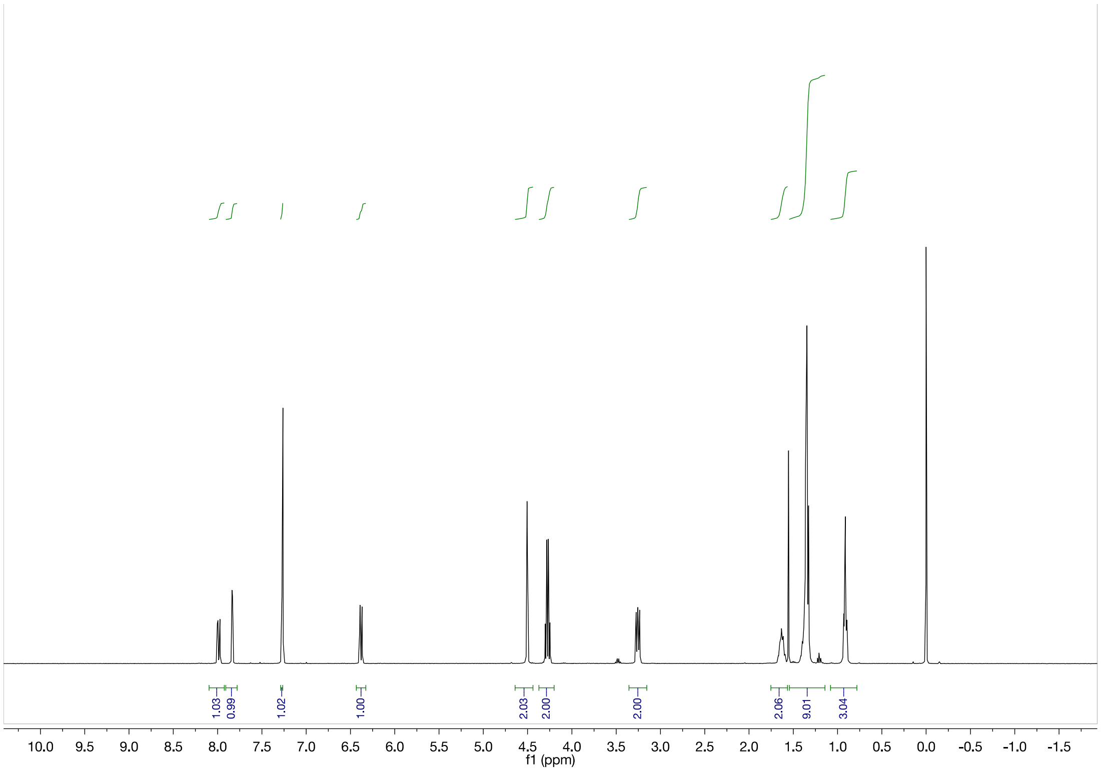

^{13}C NMR for Ethyl 1-hexyl-6-nitro-1,2-dihydroquinoline-3-carboxylate (**9b**)

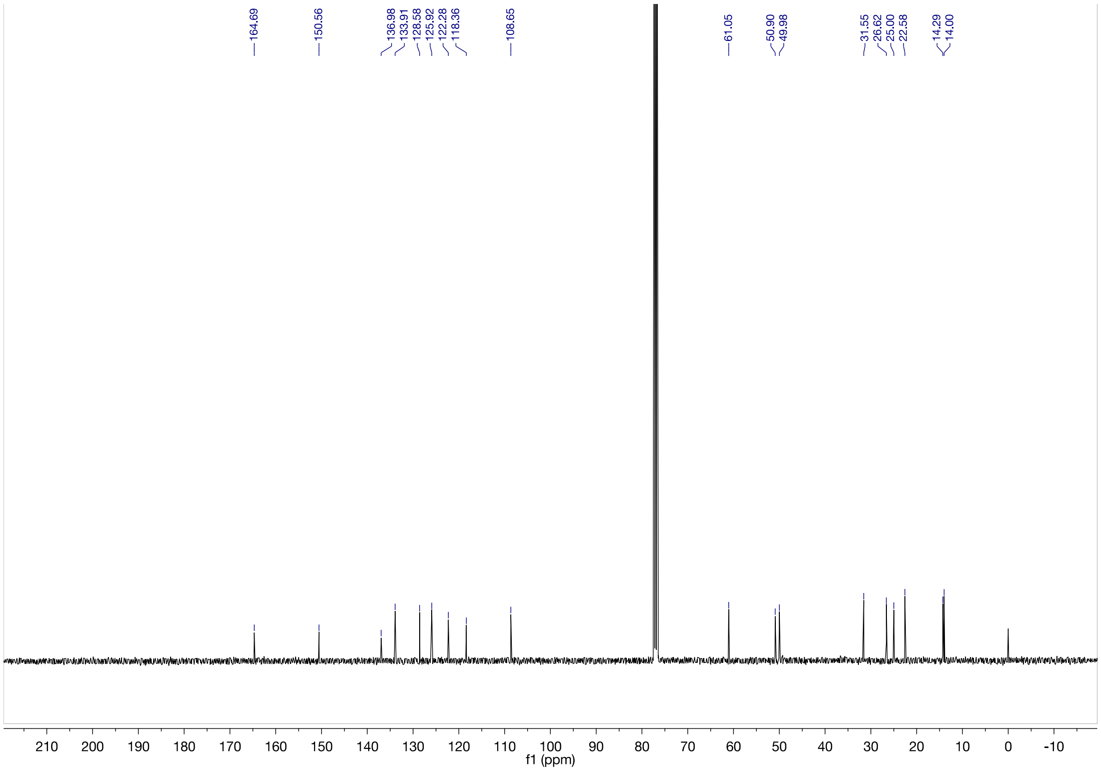

^{1}H NMR for Ethyl 1-isobutyl-6-nitro-1,2-dihydroquinoline-3-carboxylate (**9c**)

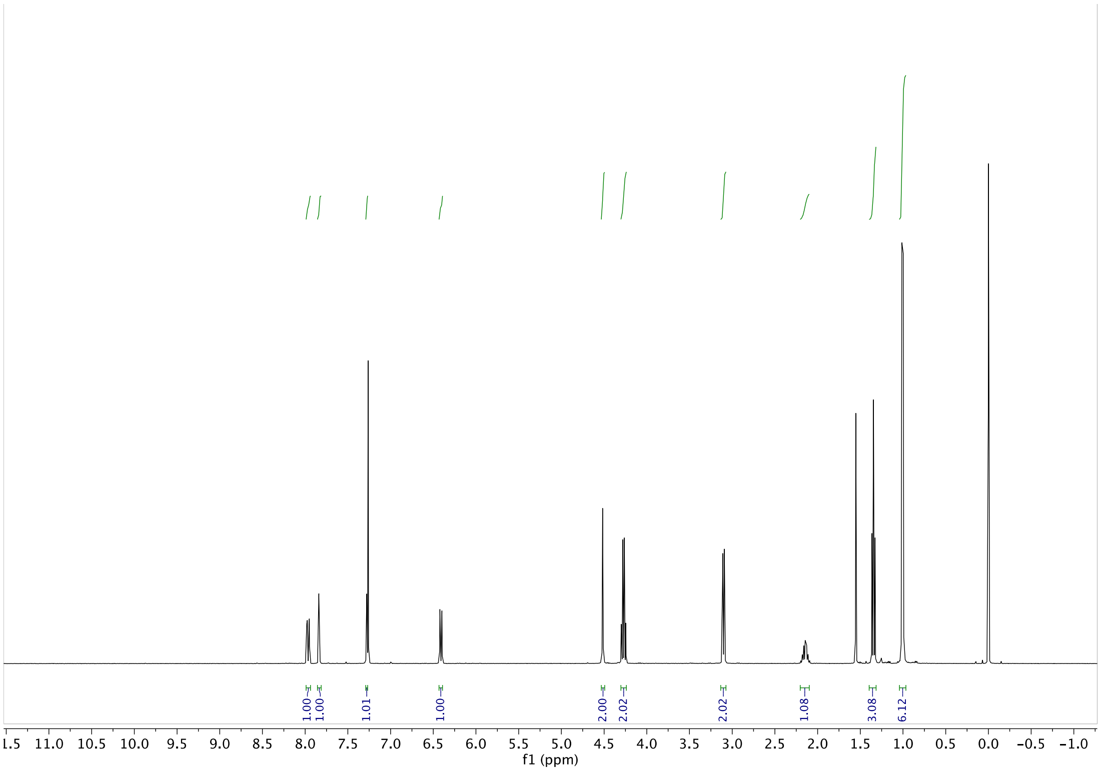

^{13}C NMR for Ethyl 1-isobutyl-6-nitro-1,2-dihydroquinoline-3-carboxylate (**9c**)

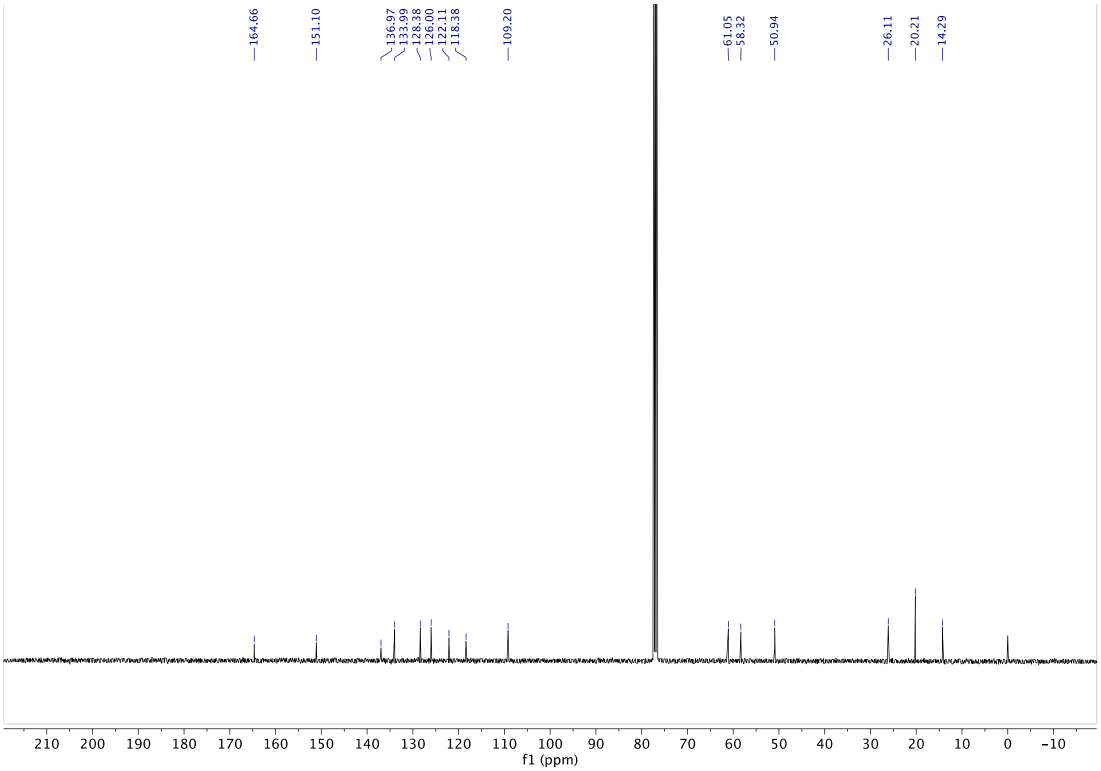

^{1}H NMR for Ethyl 1-benzyl-6-nitro-1,2-dihydroquinoline-3-carboxylate (**9d**)

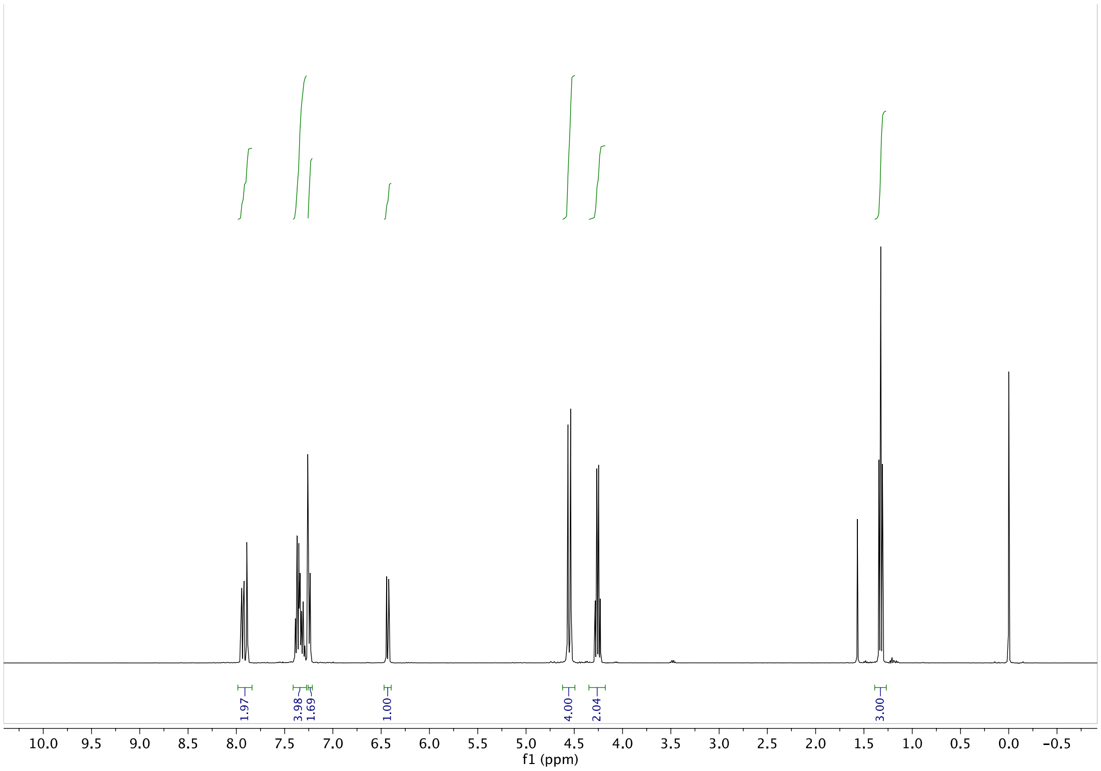

^{13}C NMR for Ethyl 1-benzyl-6-nitro-1,2-dihydroquinoline-3-carboxylate (**9d**)

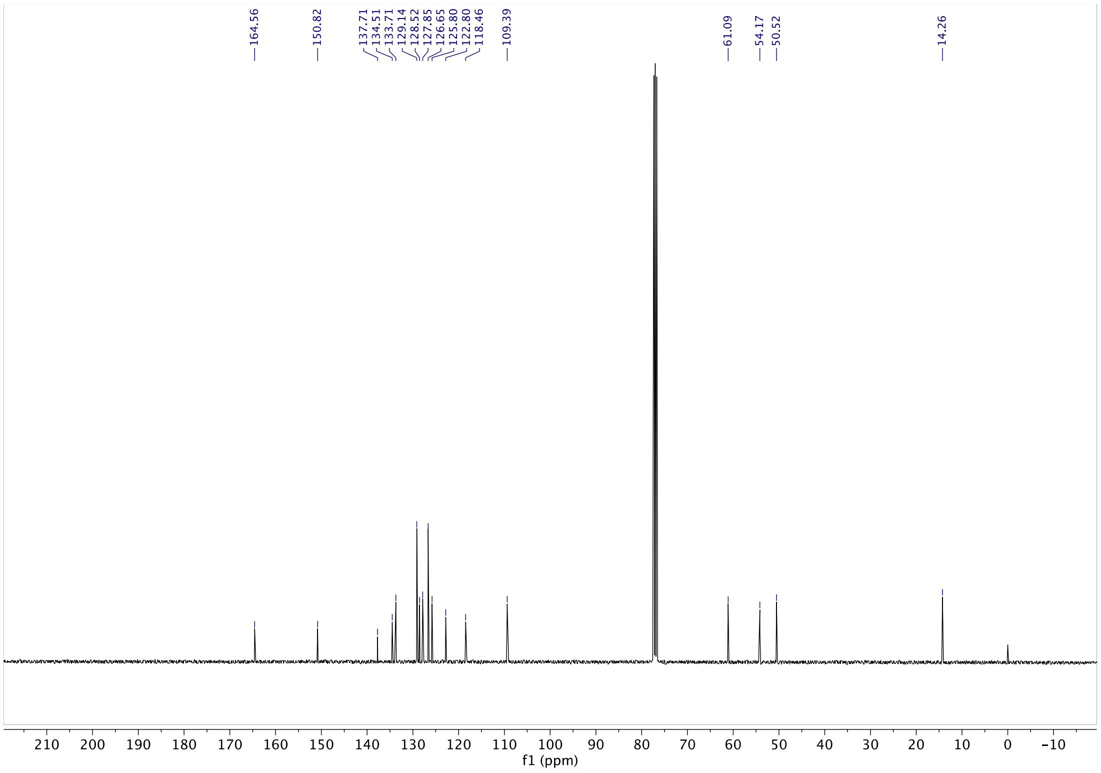

^{1}H NMR for Ethyl 6-nitro-1-phenethyl-1,2-dihydroquinoline-3-carboxylate (**9e**)

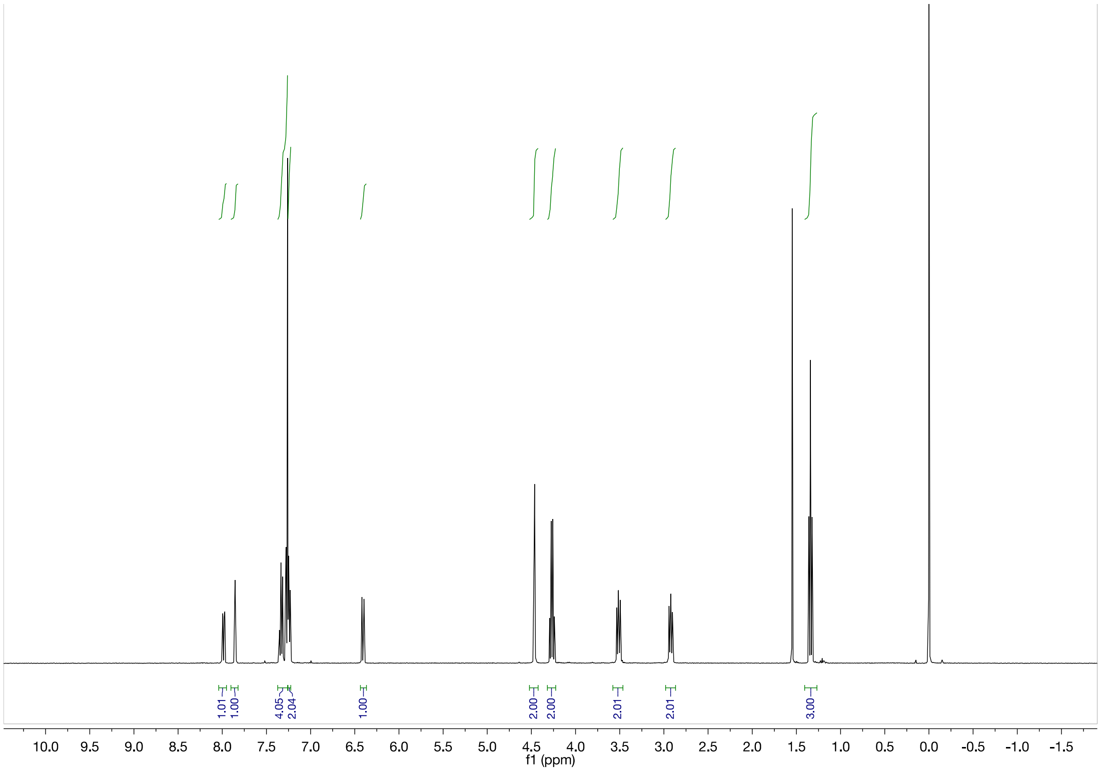

^{13}C NMR for Ethyl 6-nitro-1-phenethyl-1,2-dihydroquinoline-3-carboxylate (**9e**)

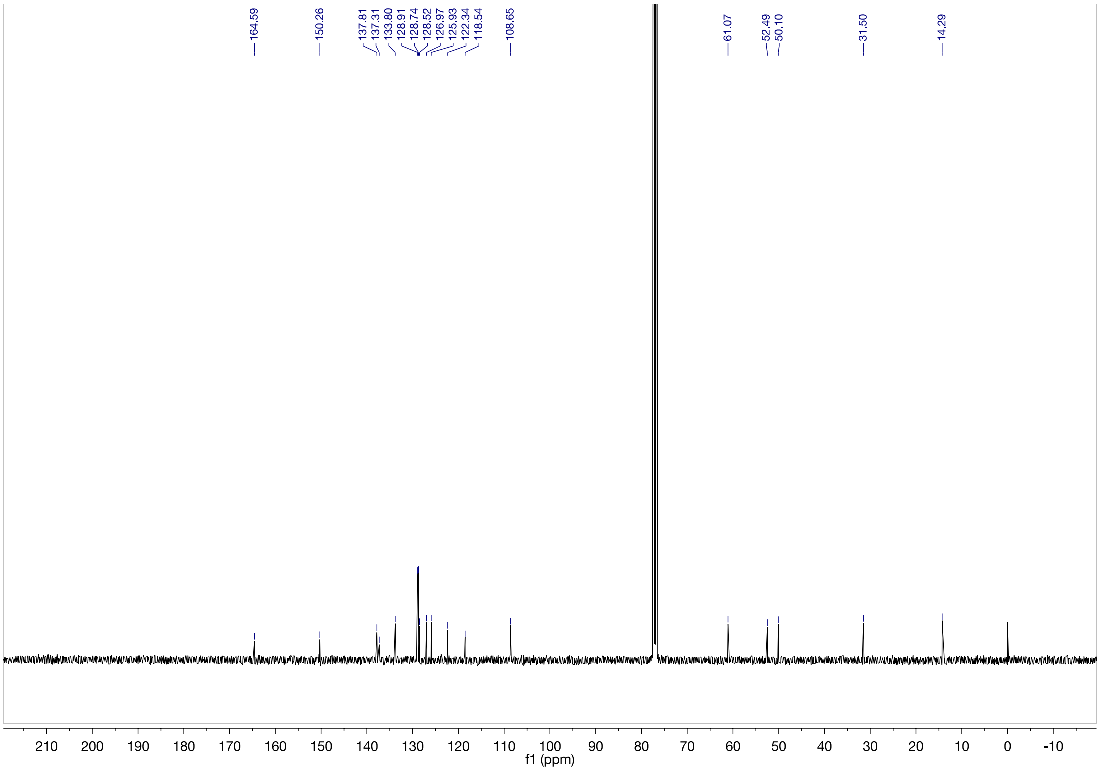

^{1}H NMR for Ethyl 6-nitro-1-phenyl-1,2-dihydroquinoline-3-carboxylate (**9f**)

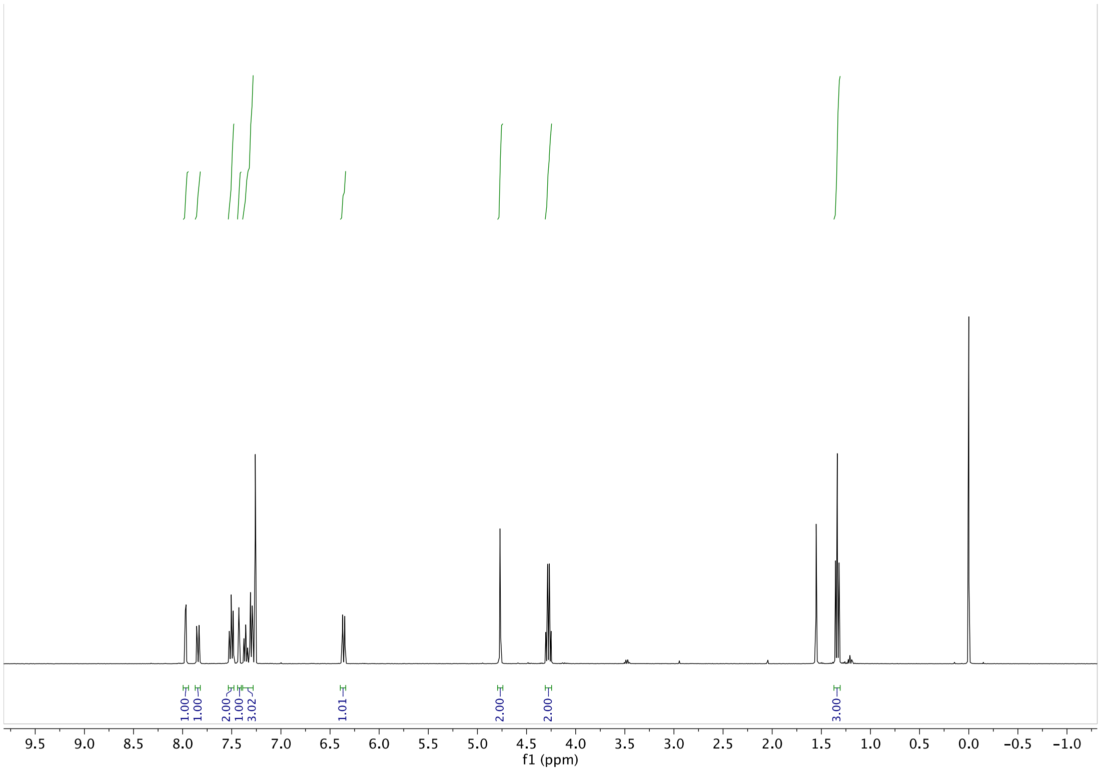

^{13}C NMR for Ethyl 6-nitro-1-phenyl-1,2-dihydroquinoline-3-carboxylate (**9f**)

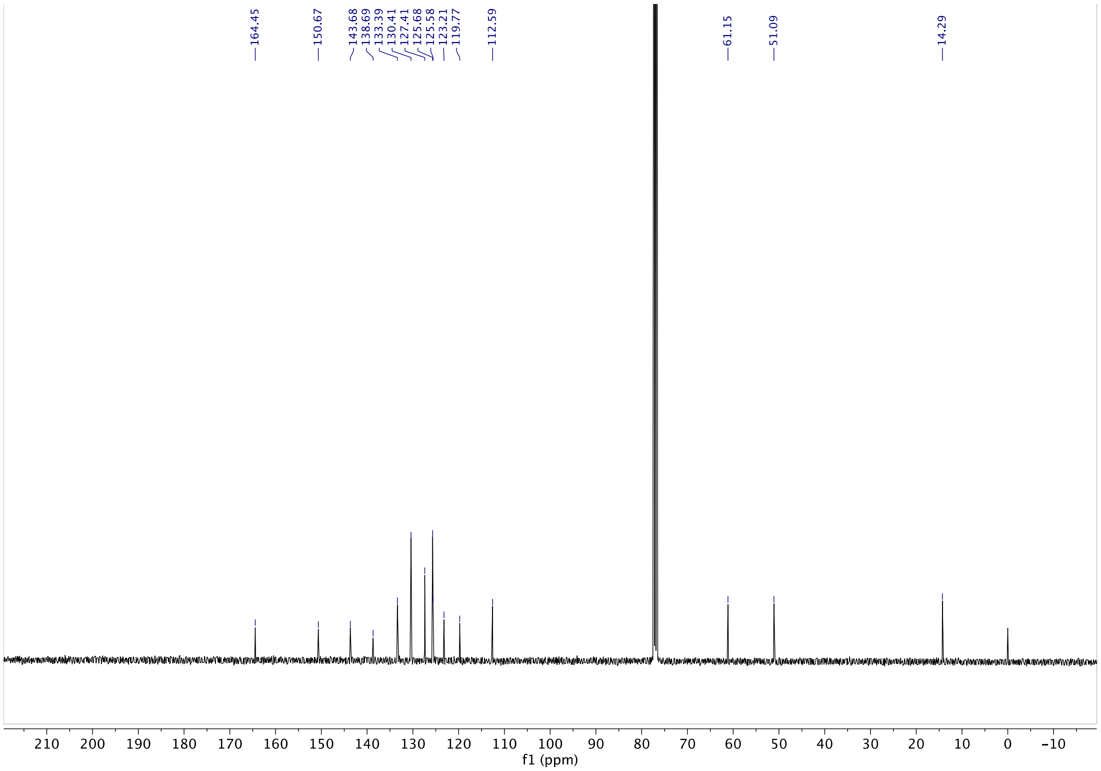

^{1}H NMR for 1-Methyl-6-nitro-1,2-dihydroquinoline-3-carbonitrile (**10a**)

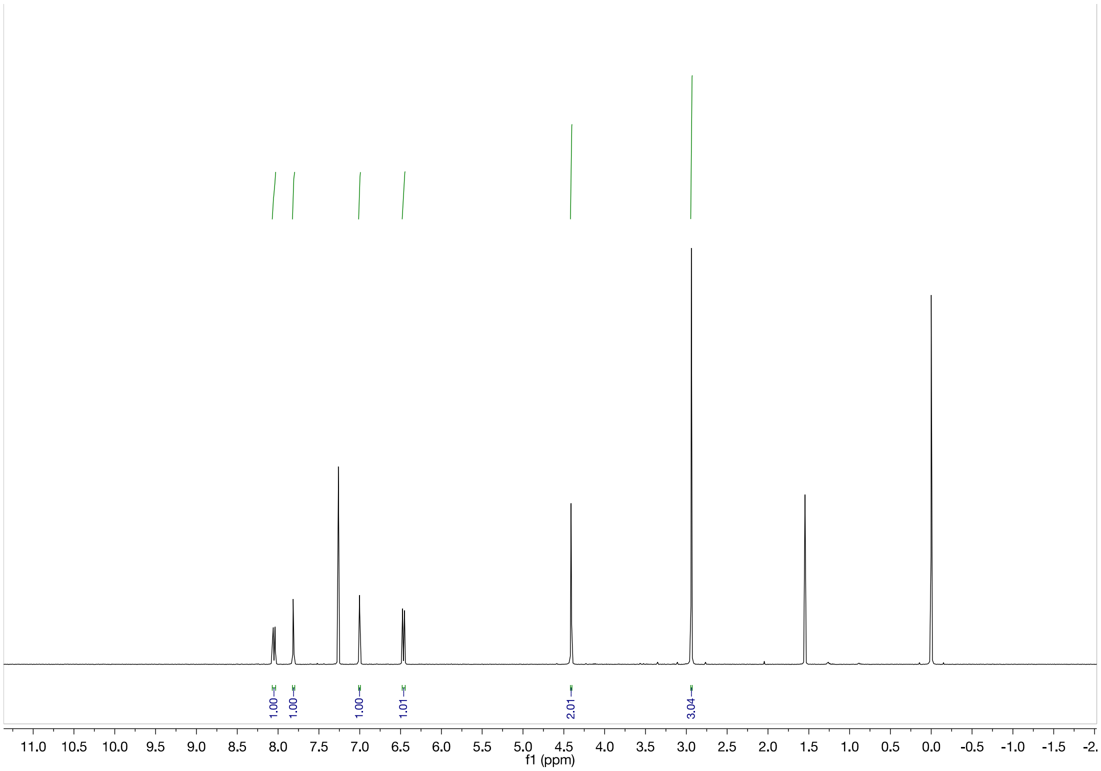

^{13}C NMR for 1-Methyl-6-nitro-1,2-dihydroquinoline-3-carbonitrile (**10a**)

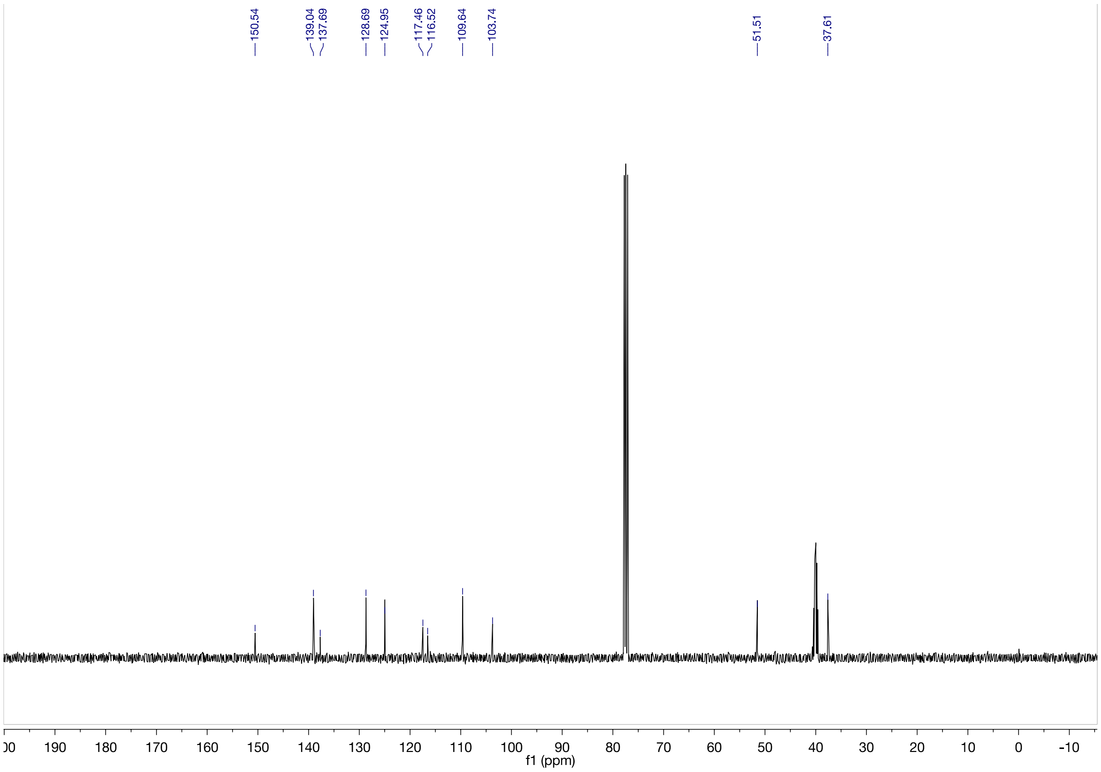

^{1}H NMR for 1-Hexyl-6-nitro-1,2-dihydroquinoline-3-carbonitrile (**10b**)

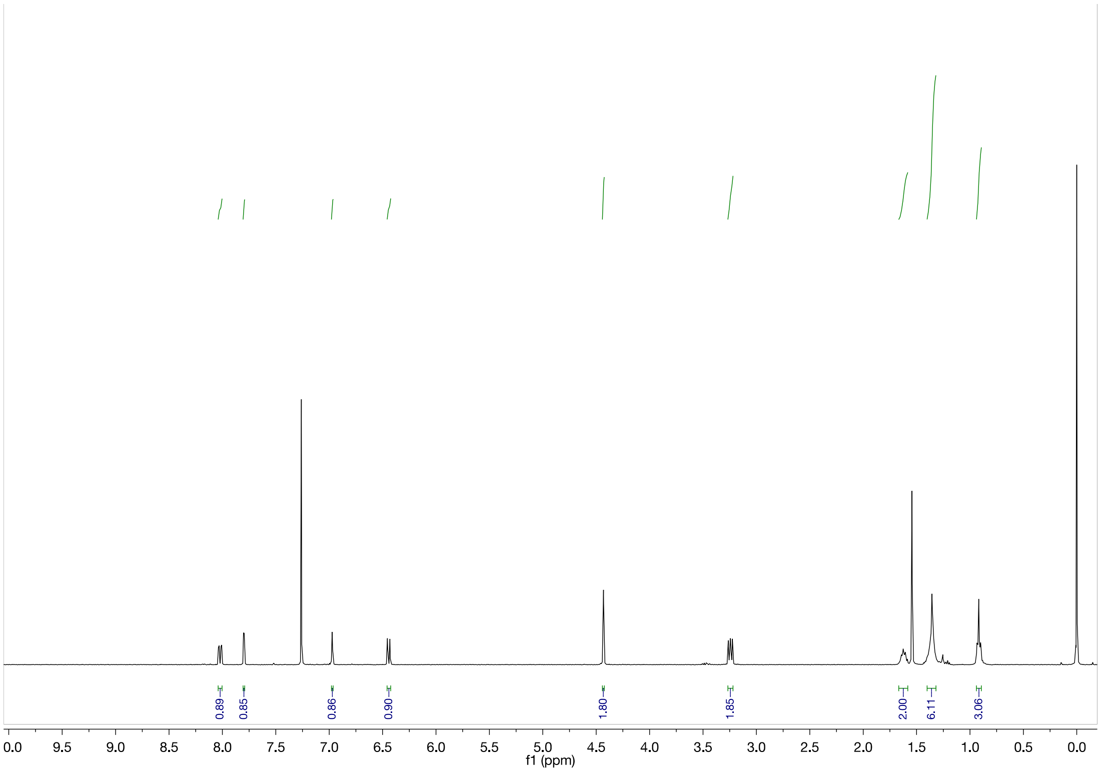

^{13}C NMR for 1-Hexyl-6-nitro-1,2-dihydroquinoline-3-carbonitrile **(10b)**

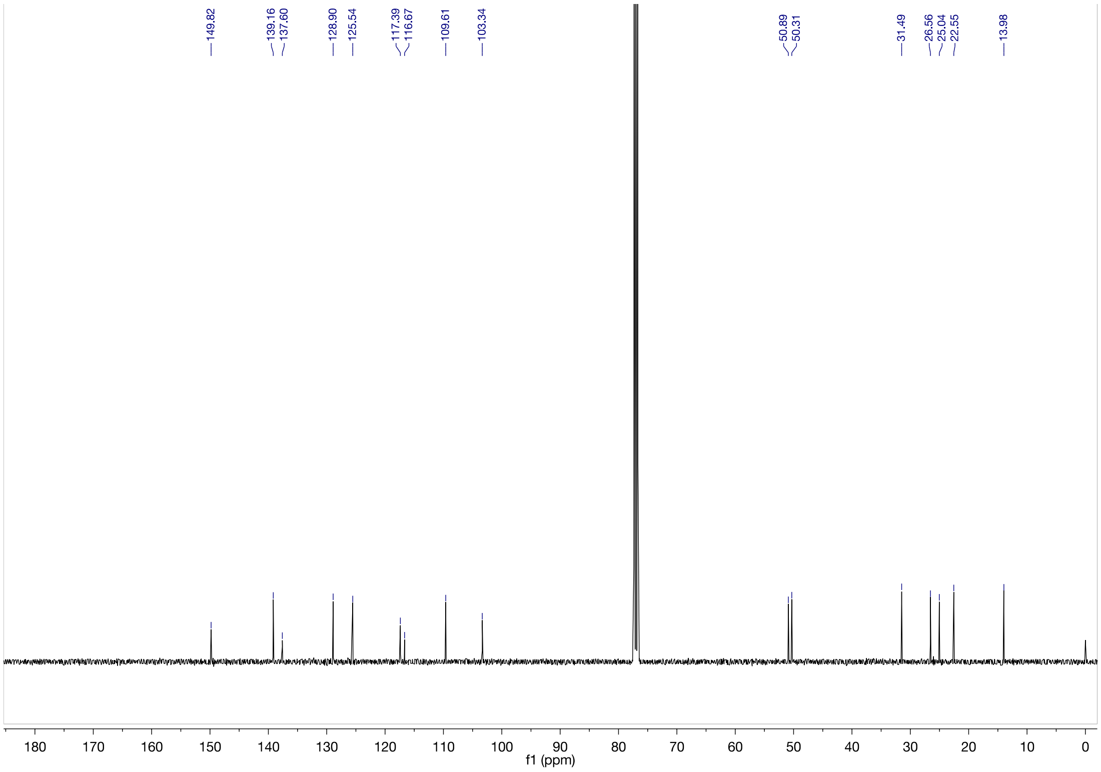

^{1}H NMR for 1-Isobutyl-6-nitro-1,2-dihydroquinoline-3-carbonitrile (**10c**)

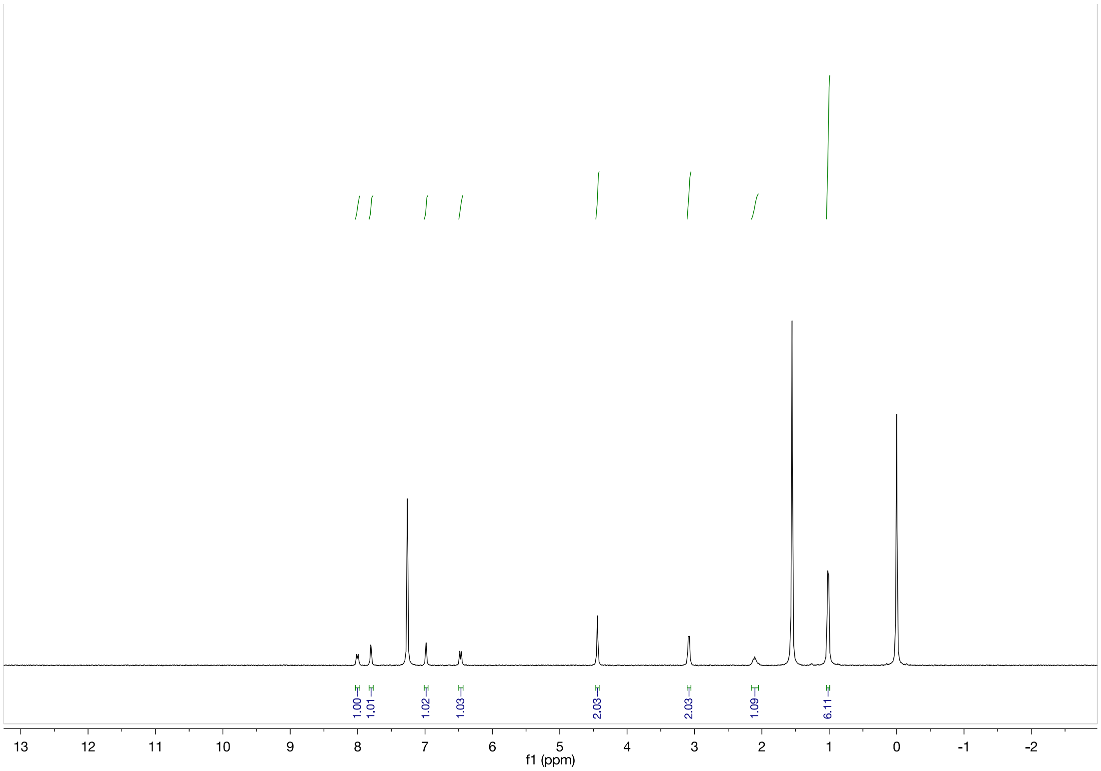

^{13}C NMR for 1-Isobutyl-6-nitro-1,2-dihydroquinoline-3-carbonitrile (**10c**)

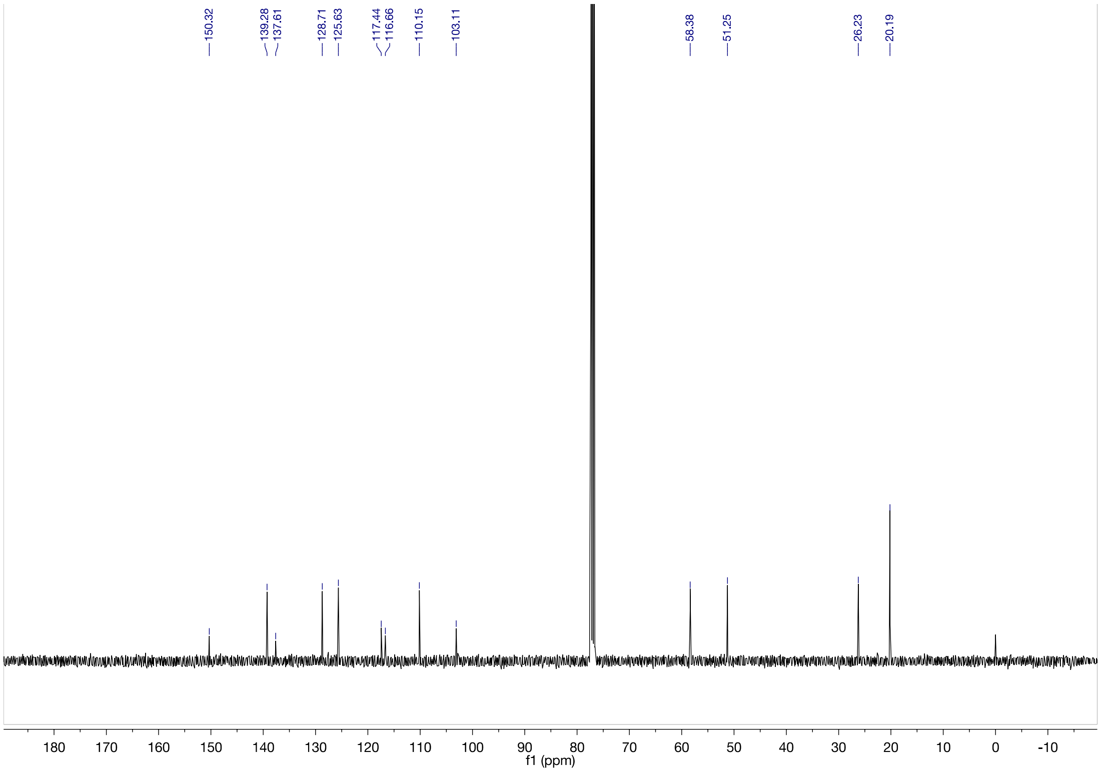

^{1}H NMR for 1-Benzyl-6-nitro-1,2-dihydroquinoline-3-carbonitrile (**10d**)

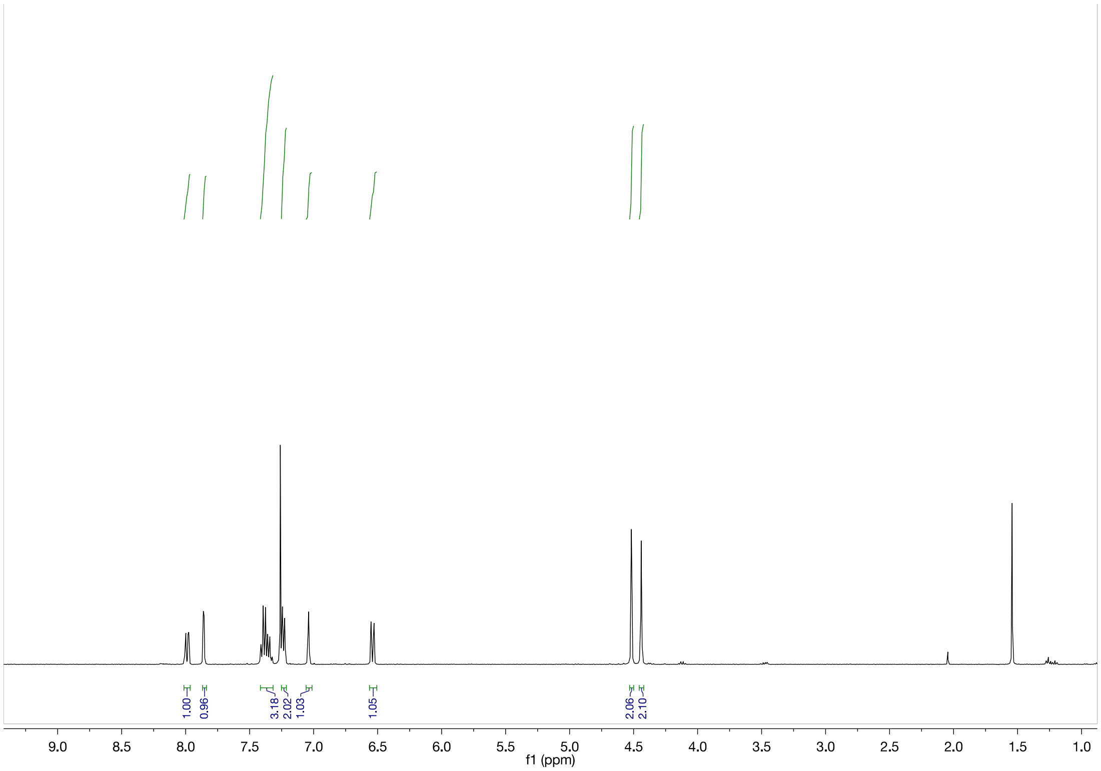

^{13}C NMR for 1-Benzyl-6-nitro-1,2-dihydroquinoline-3-carbonitrile (**10d**)

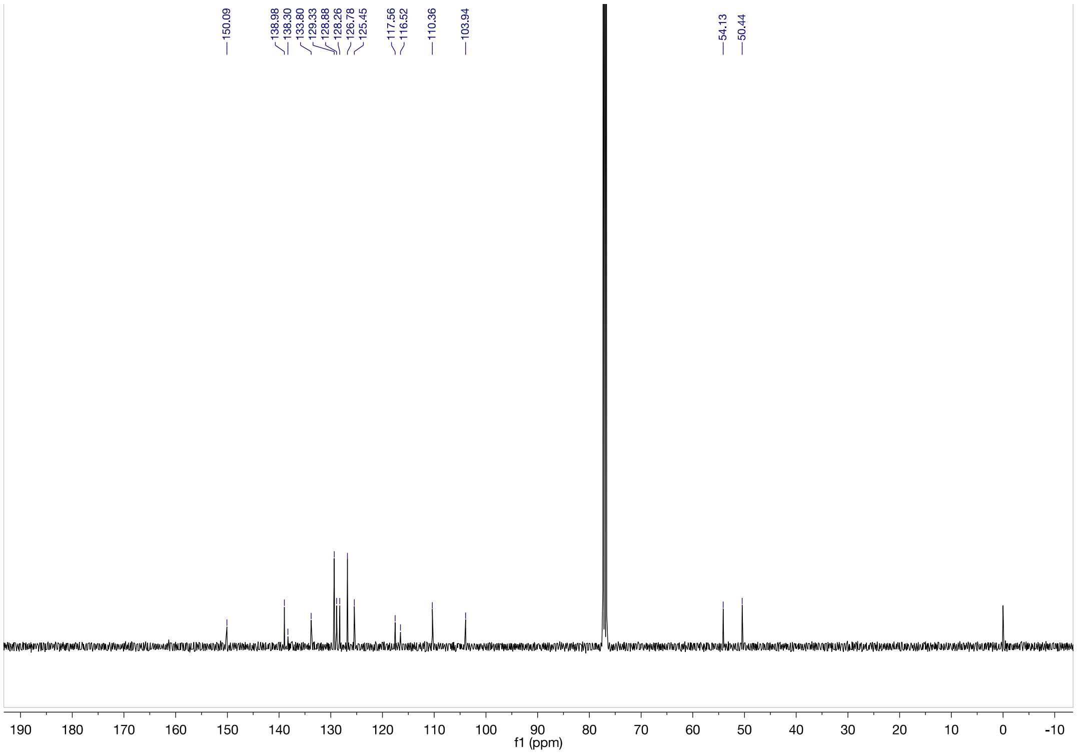

^{1}H NMR for 6-Nitro-1-phenethyl-1,2-dihydroquinoline-3-carbonitrile (**10e**)

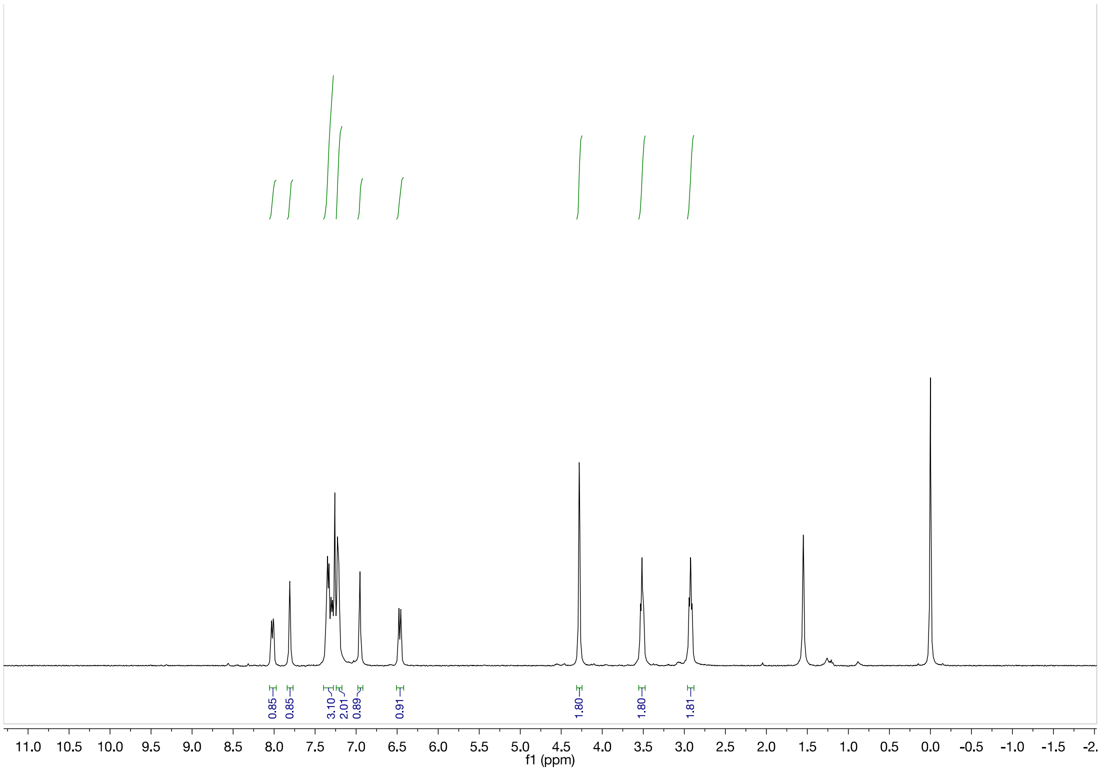

^{13}C NMR for 6-Nitro-1-phenethyl-1,2-dihydroquinoline-3-carbonitrile (**10e**)

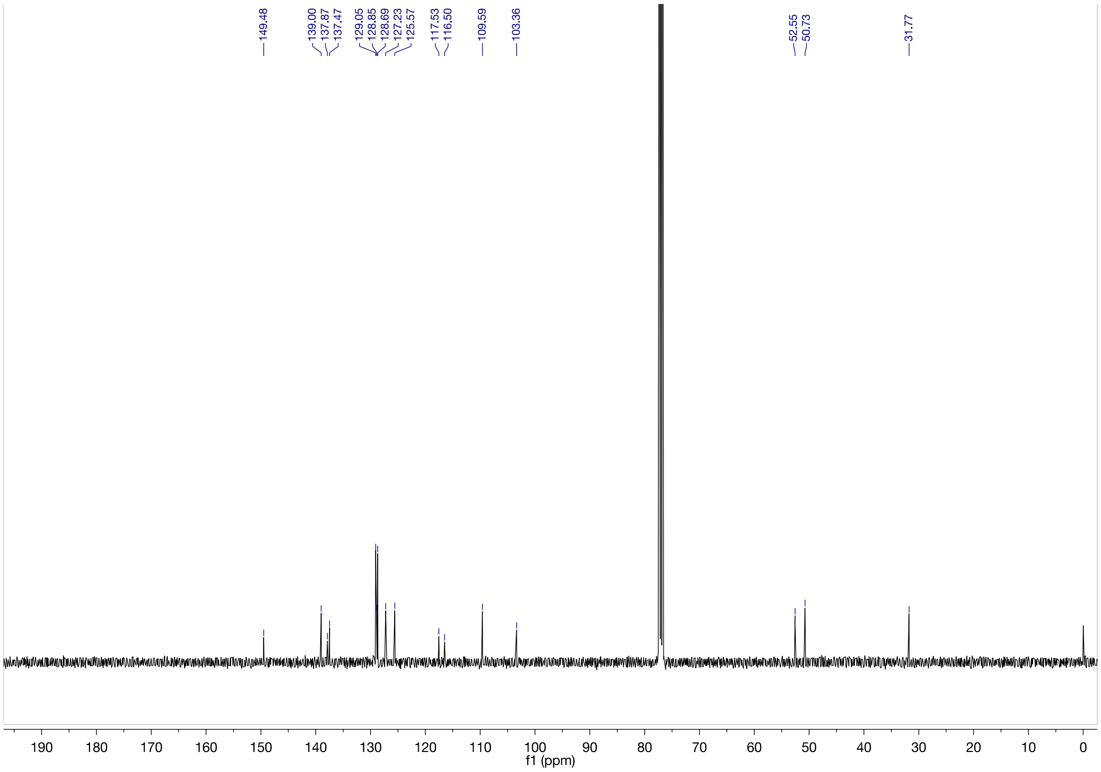

^{1}H NMR for 6-Nitro-1-phenyl-1,2-dihydroquinoline-3-carbonitrile (**10f**)

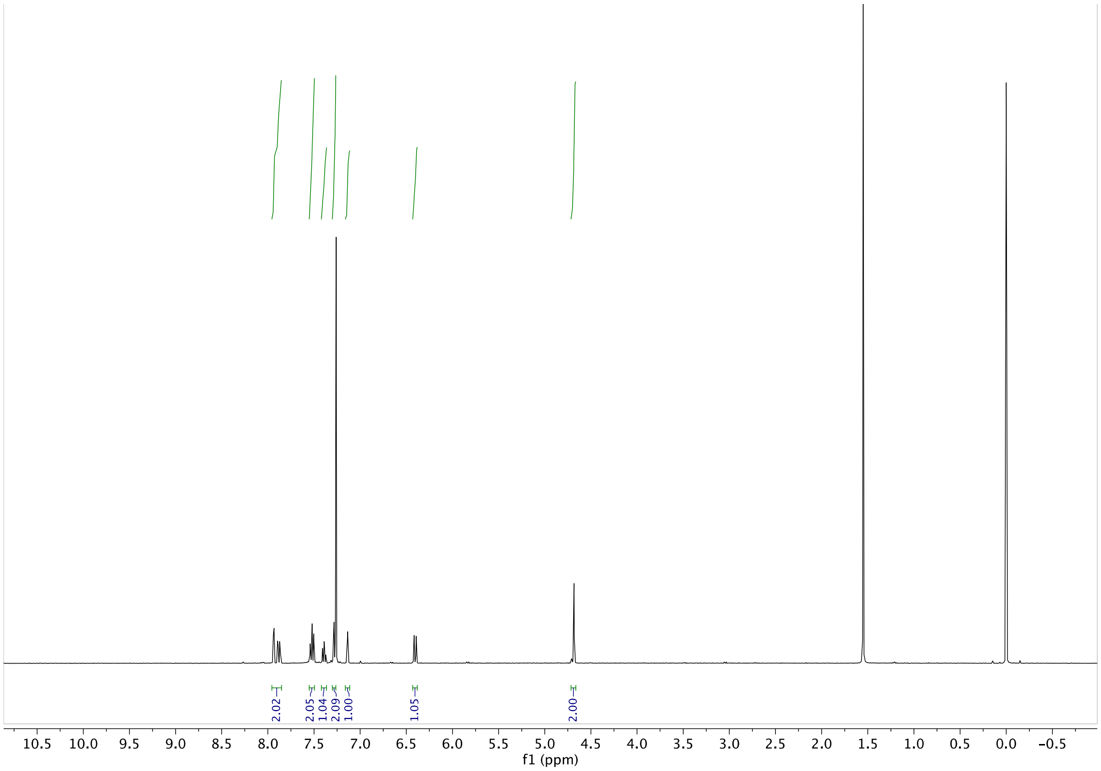

^{13}C NMR for 6-Nitro-1-phenyl-1,2-dihydroquinoline-3-carbonitrile (**10f**)

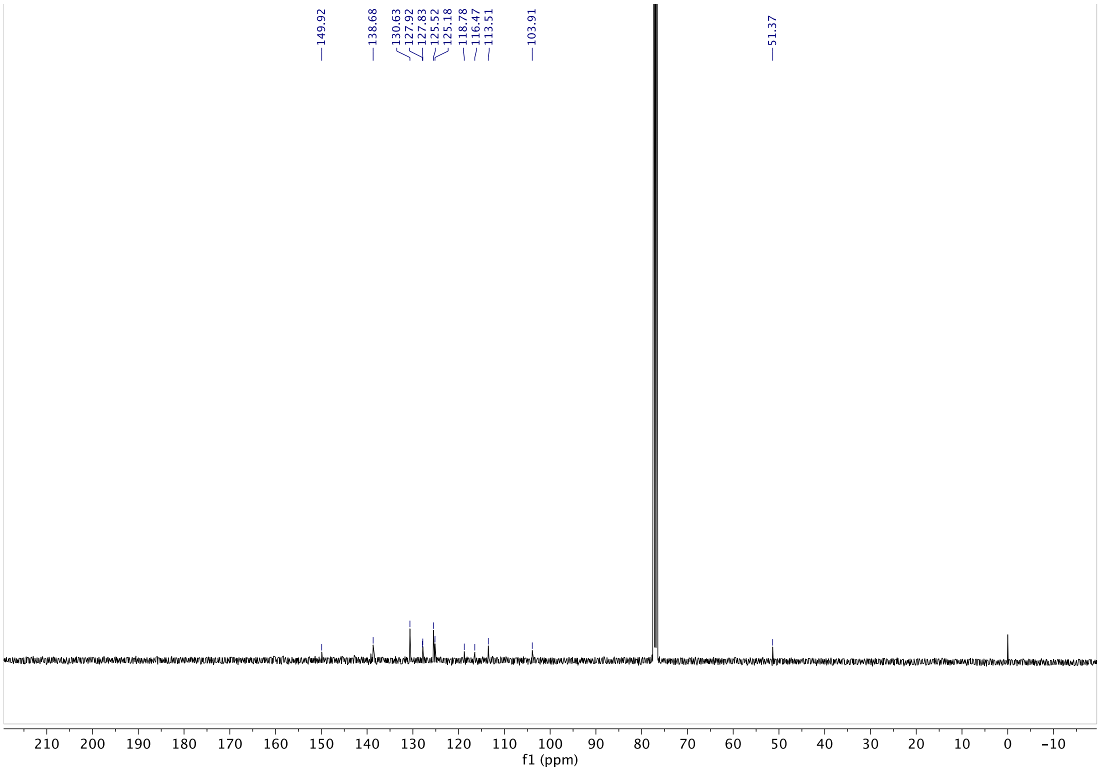

^{1}H NMR for Ethyl 6-cyano-1-methyl-1,2-dihydroquinoline-3-carboxylate (**11a**)

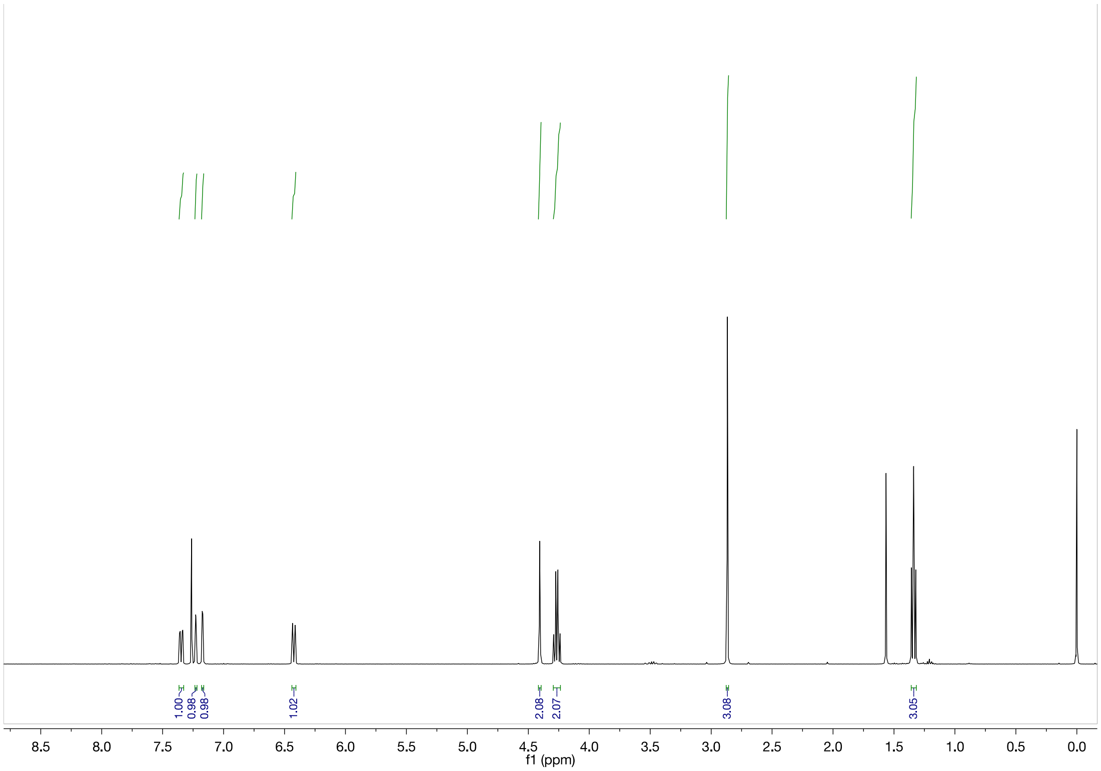

^{13}C NMR for Ethyl 6-cyano-1-methyl-1,2-dihydroquinoline-3-carboxylate (**11a**)

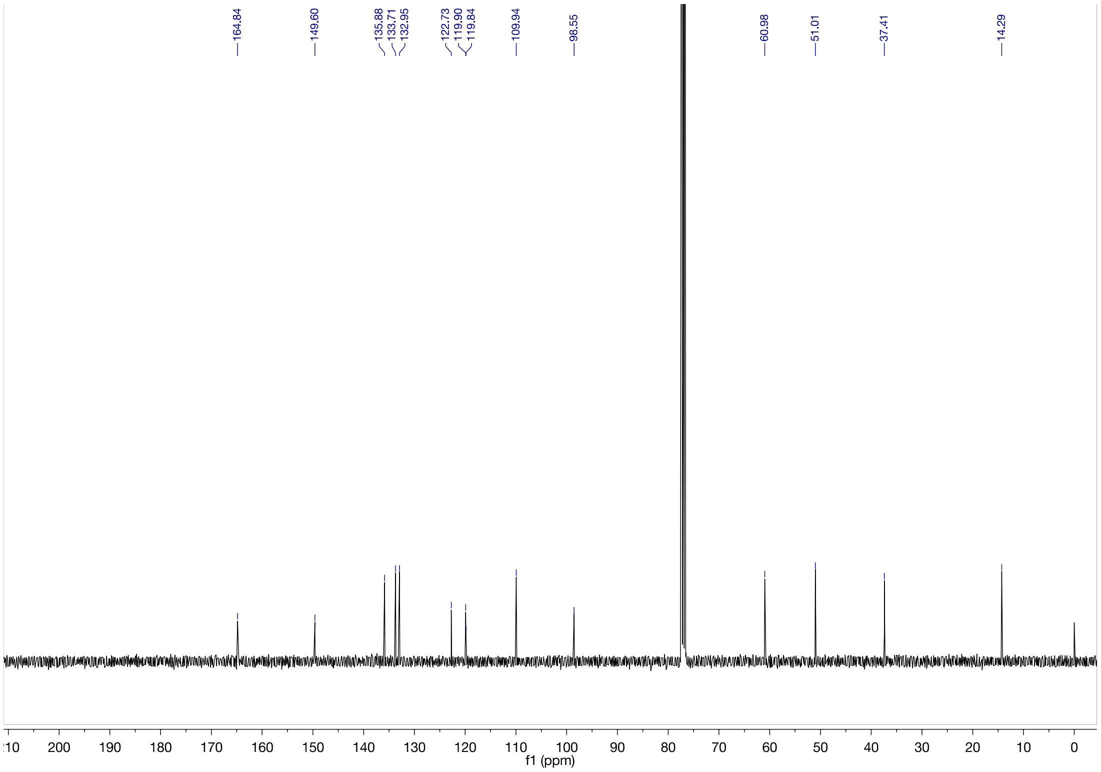

^{1}H NMR for Ethyl 6-cyano-1-hexyl-1,2-dihydroquinoline-3-carboxylate (**11b**)

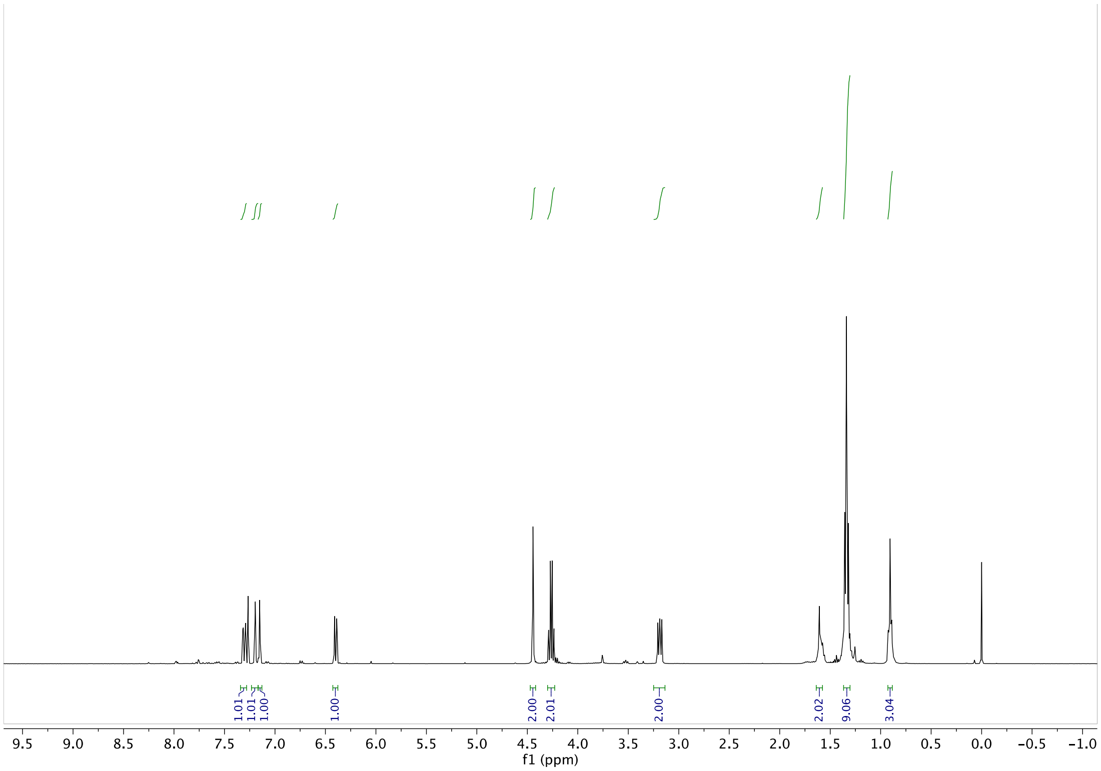

^{13}C NMR for Ethyl 6-cyano-1-hexyl-1,2-dihydroquinoline-3-carboxylate (**11b**)

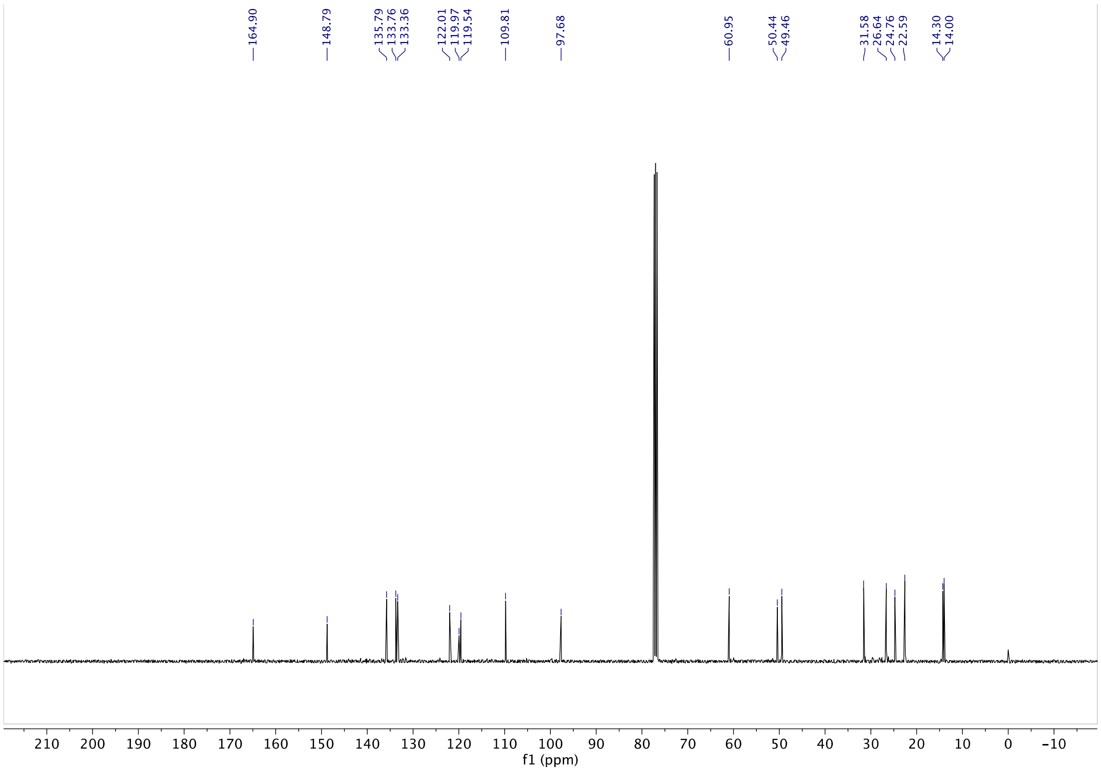

^{1}H NMR for Ethyl 6-cyano-1-isobutyl-1,2-dihydroquinoline-3-carboxylate (**11c**)

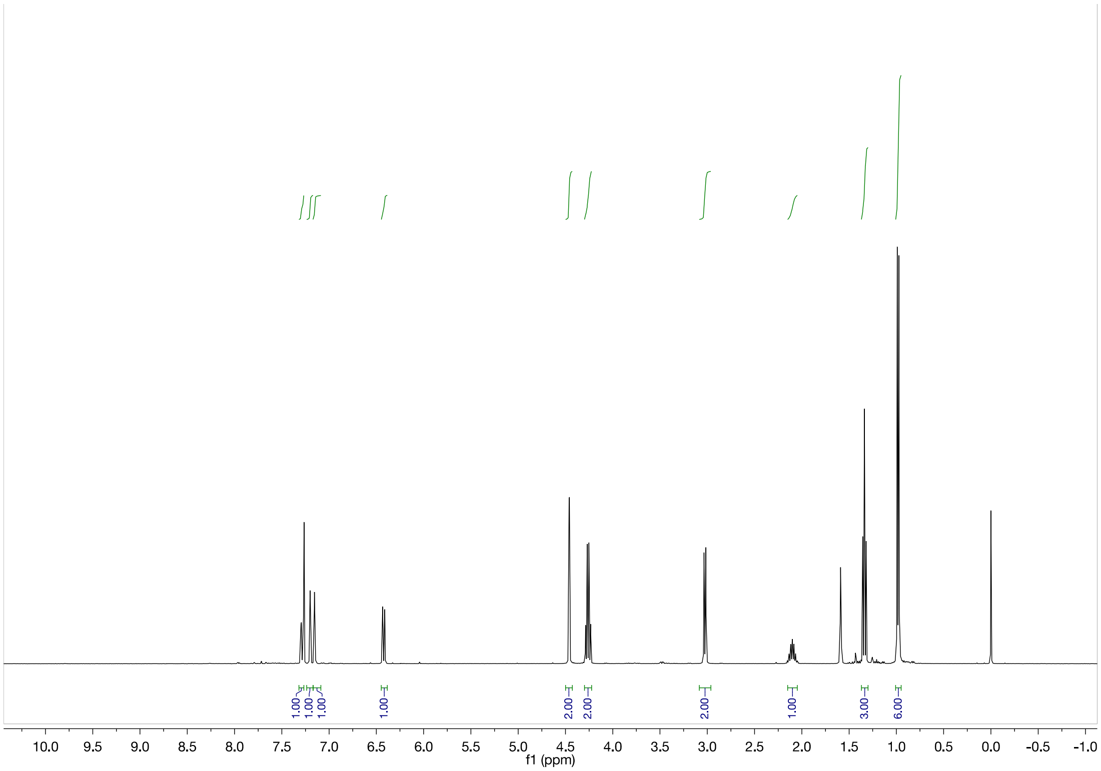

^{13}C NMR for Ethyl 6-cyano-1-isobutyl-1,2-dihydroquinoline-3-carboxylate (**11c**)

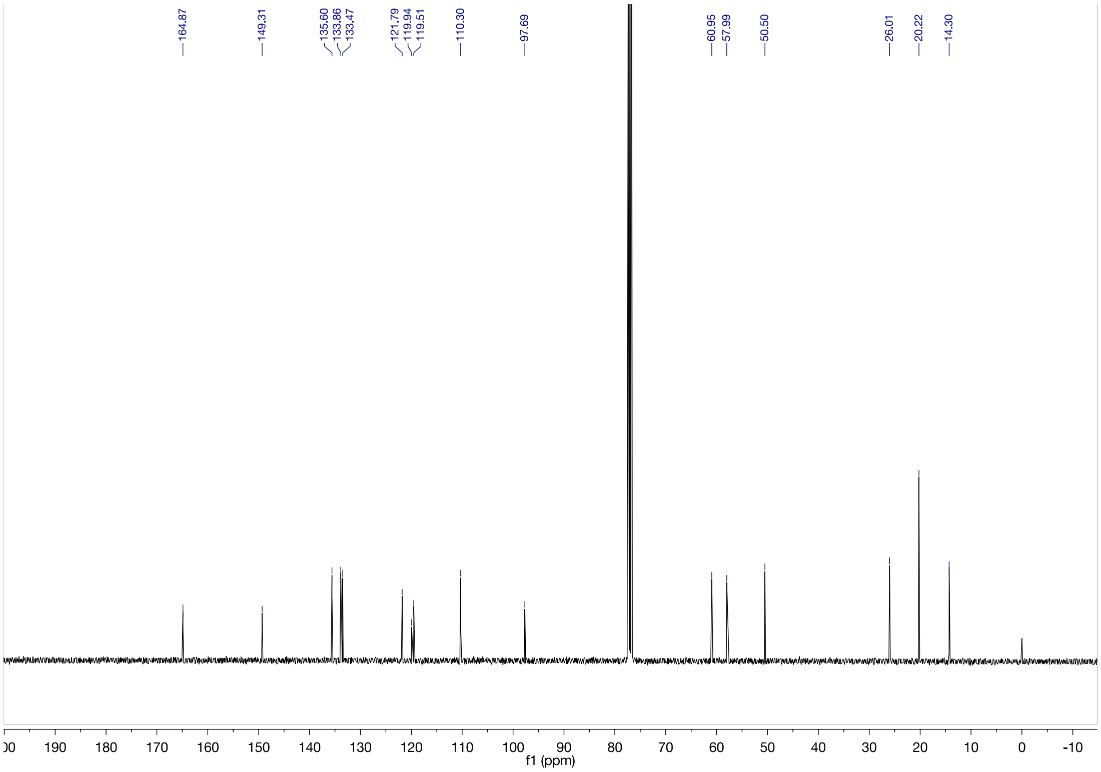

^1H NMR for Ethyl 1-benzyl-6-cyano-1,2-dihydroquinoline-3-carboxylate (**11d**)

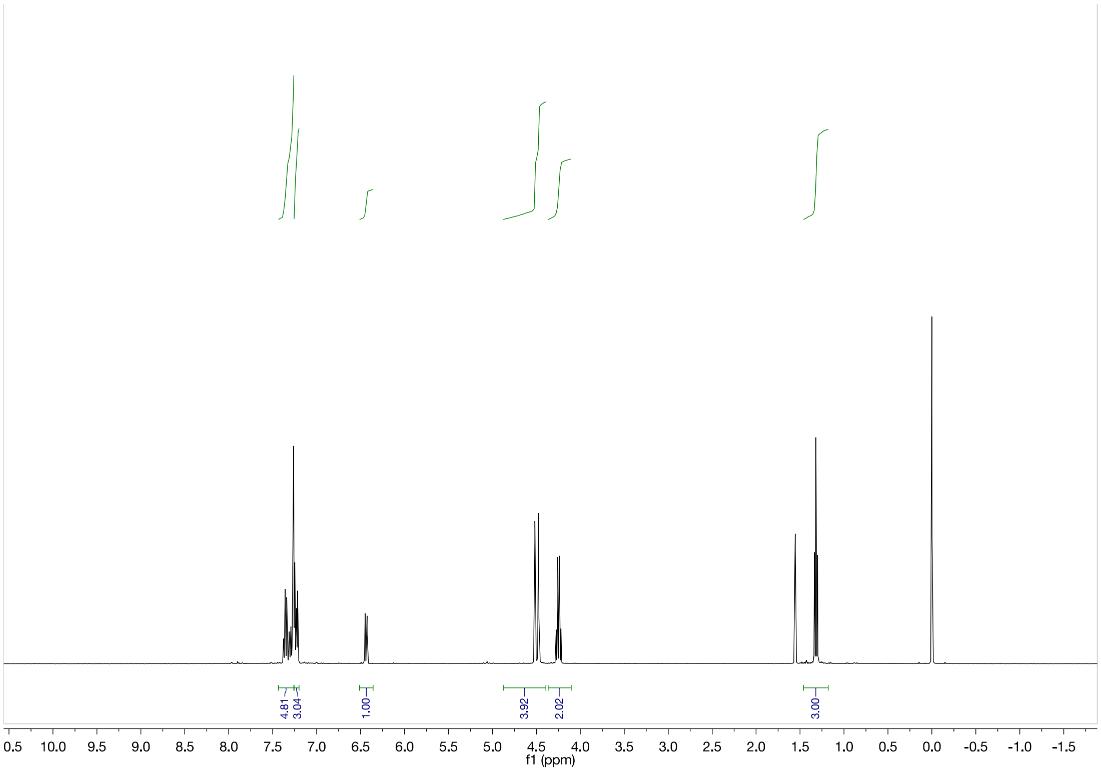

^{13}C NMR for Ethyl 1-benzyl-6-cyano-1,2-dihydroquinoline-3-carboxylate (**11d**)

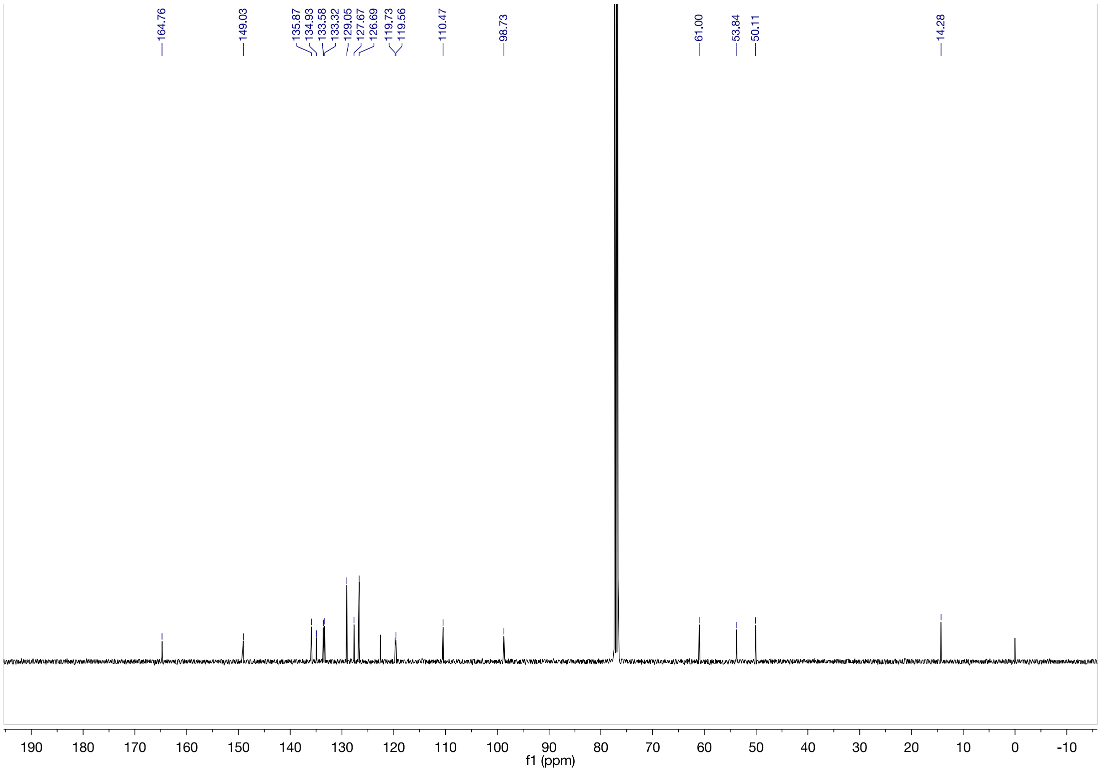

¹H NMR for Ethyl 6-cyano-1-phenethyl-1,2-dihydroquinoline-3-carboxylate (**11e**)

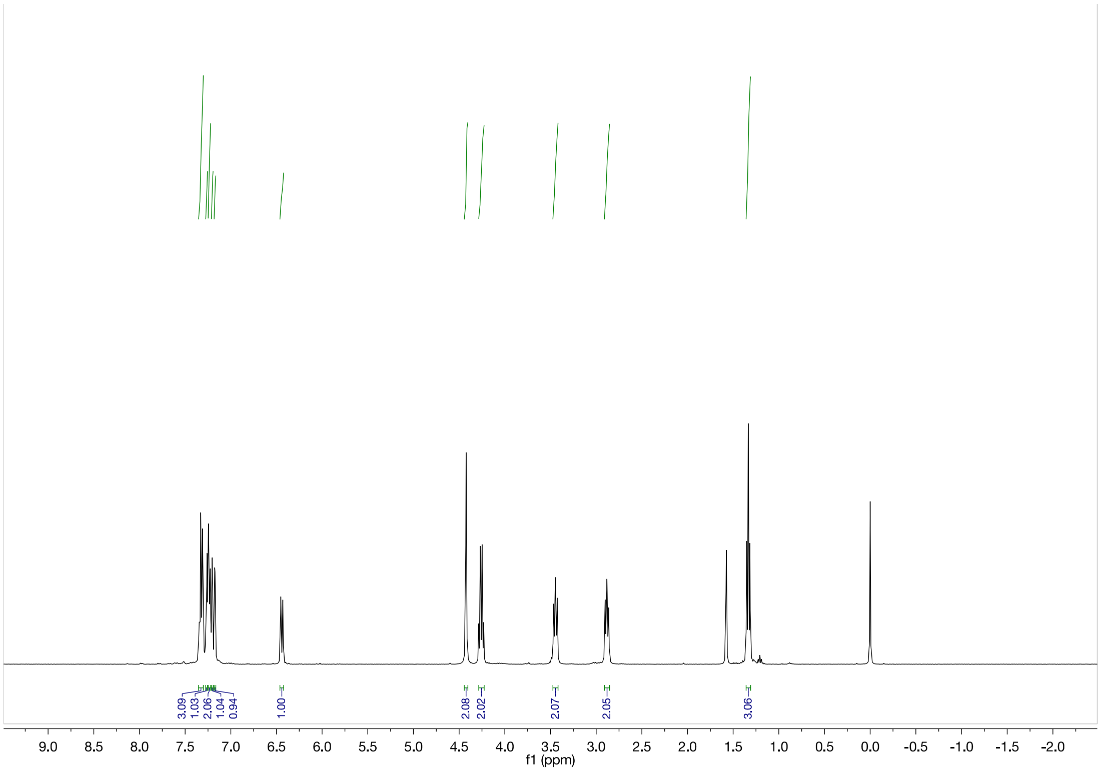

¹³C NMR for Ethyl 6-cyano-1-phenethyl-1,2-dihydroquinoline-3-carboxylate (**11e**)

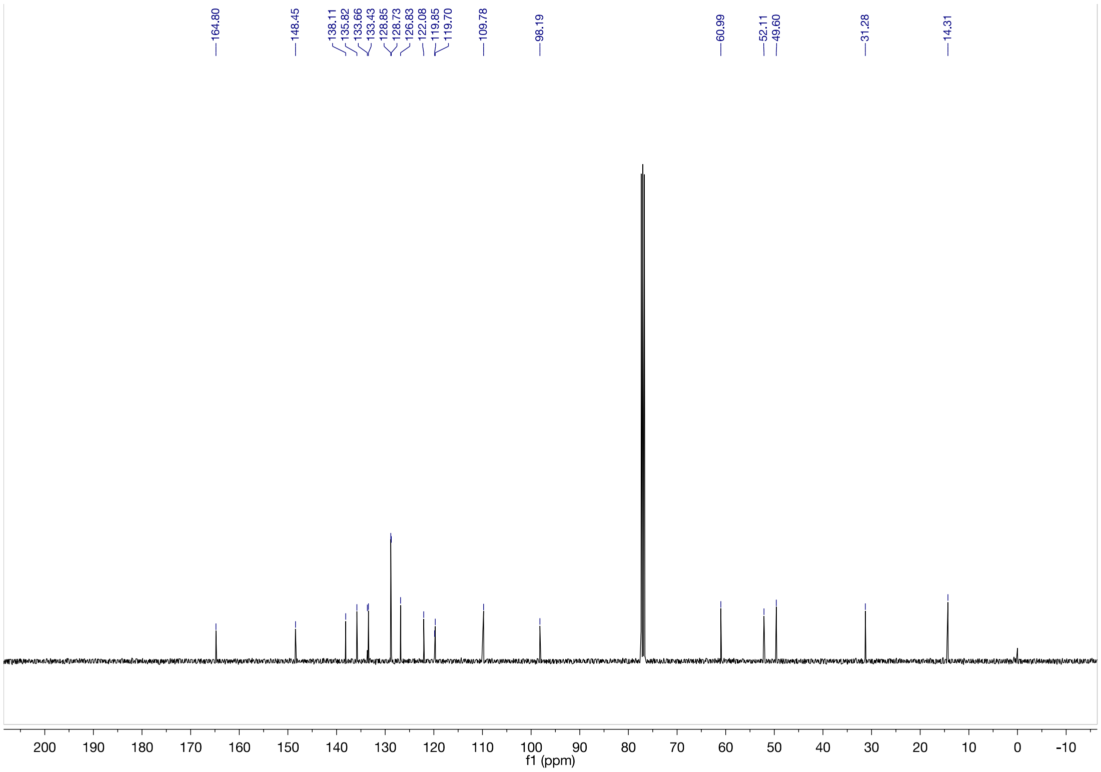

^{1}H NMR for Ethyl 6-cyano-1-phenyl-1,2-dihydroquinoline-3-carboxylate (**11f**)

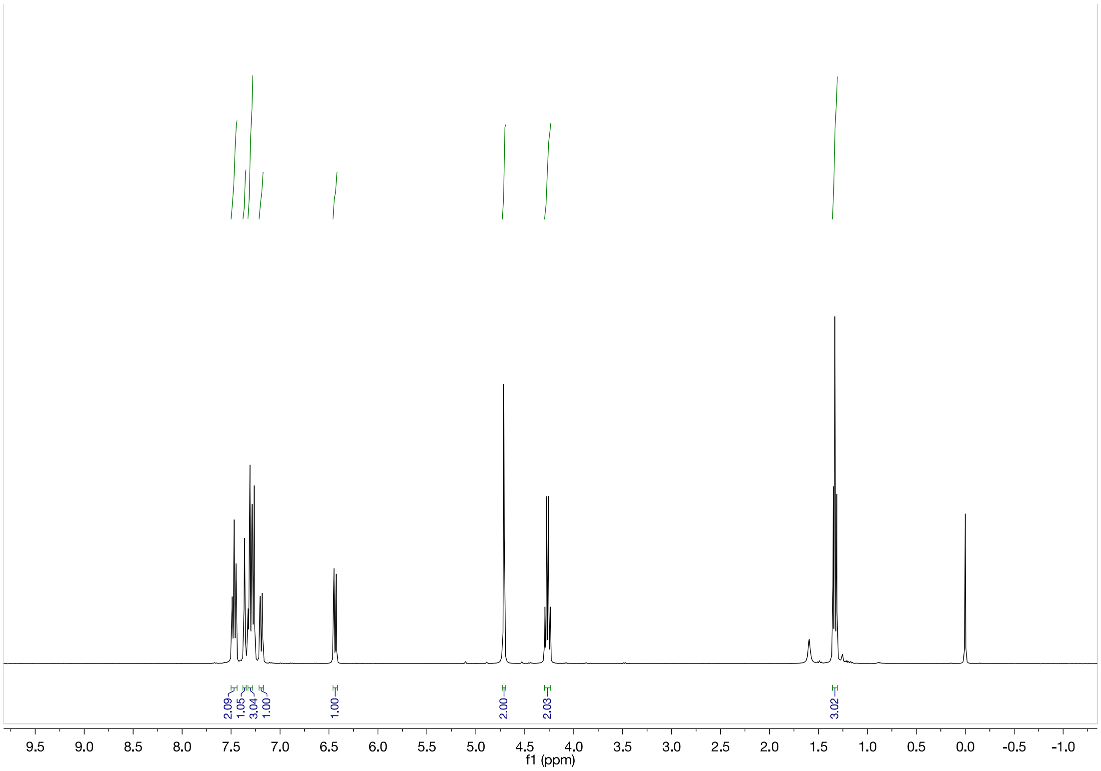

^{13}C NMR for Ethyl 6-cyano-1-phenyl-1,2-dihydroquinoline-3-carboxylate (**11f**)

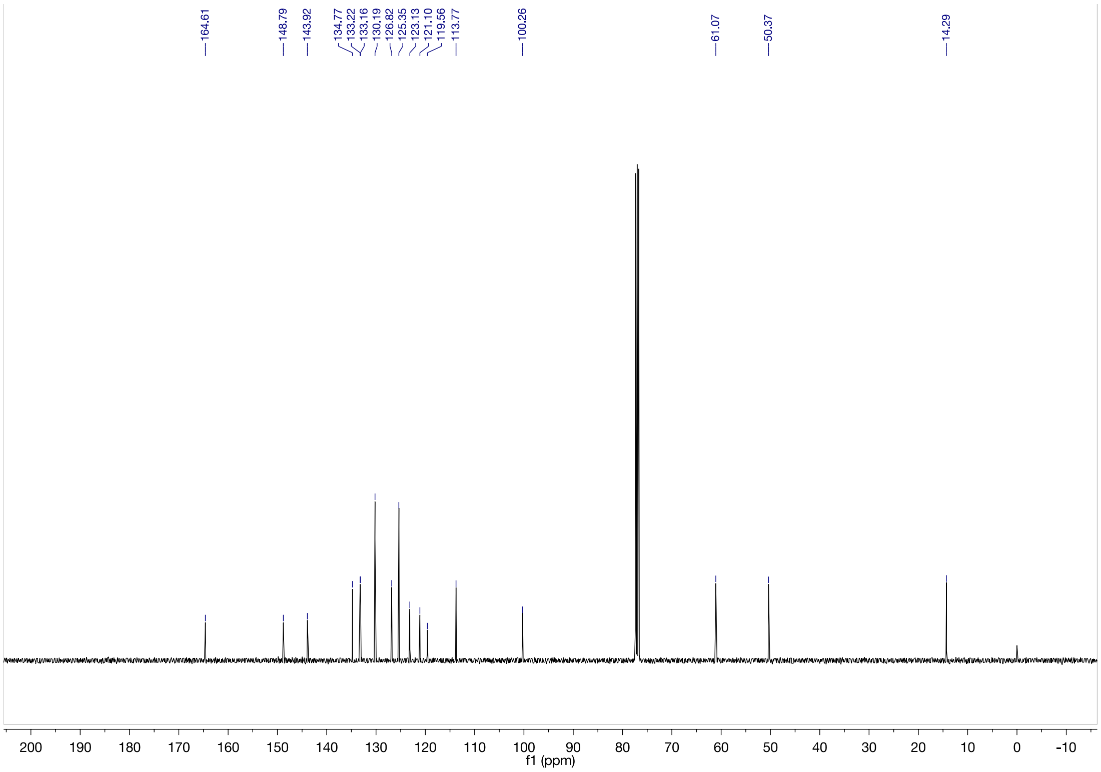

^{1}H NMR for 1-Methyl-1,2-dihydroquinoline-3,6-dicarbonitrile (**12a**)

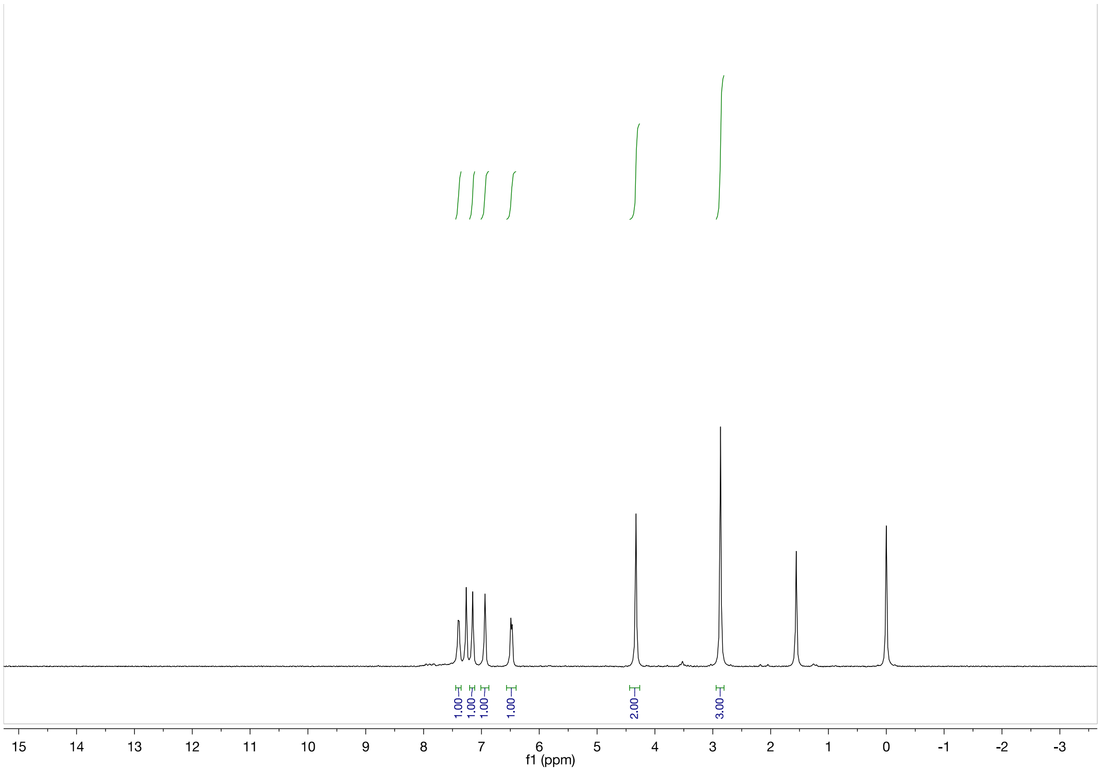

^{13}C NMR for 1-Methyl-1,2-dihydroquinoline-3,6-dicarbonitrile (**12a**)

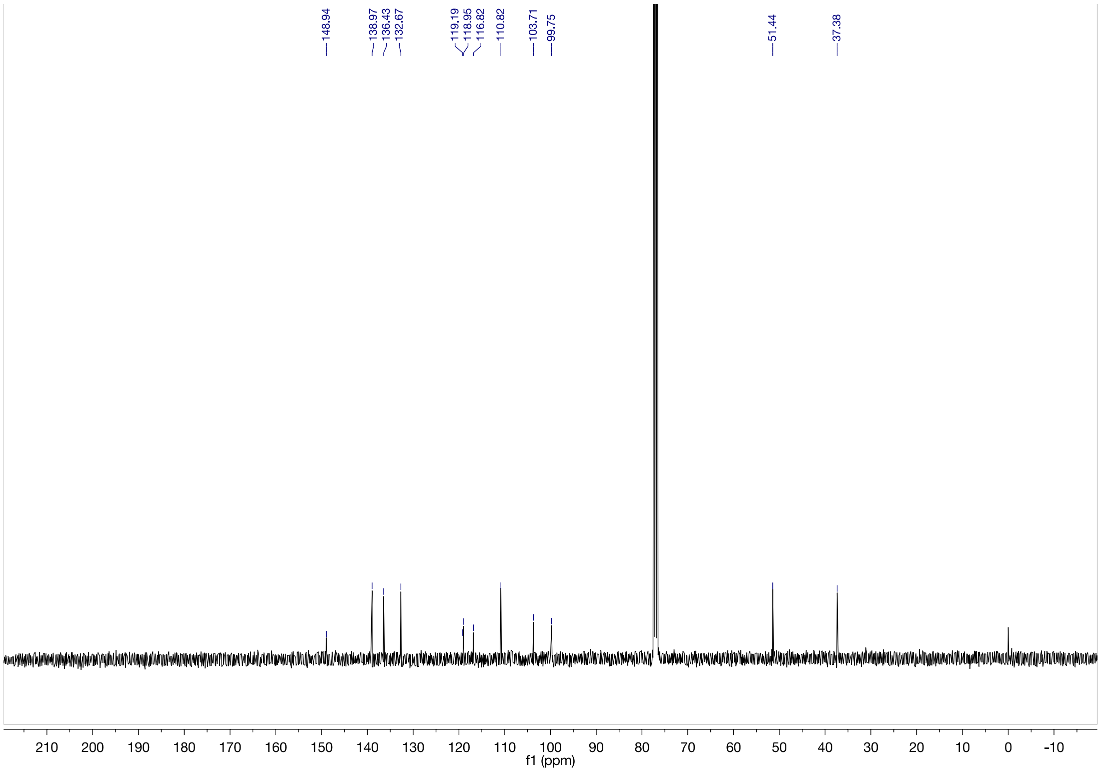

^{1}H NMR for 1-Hexyl-1,2-dihydroquinoline-3,6-dicarbonitrile (**12b**)

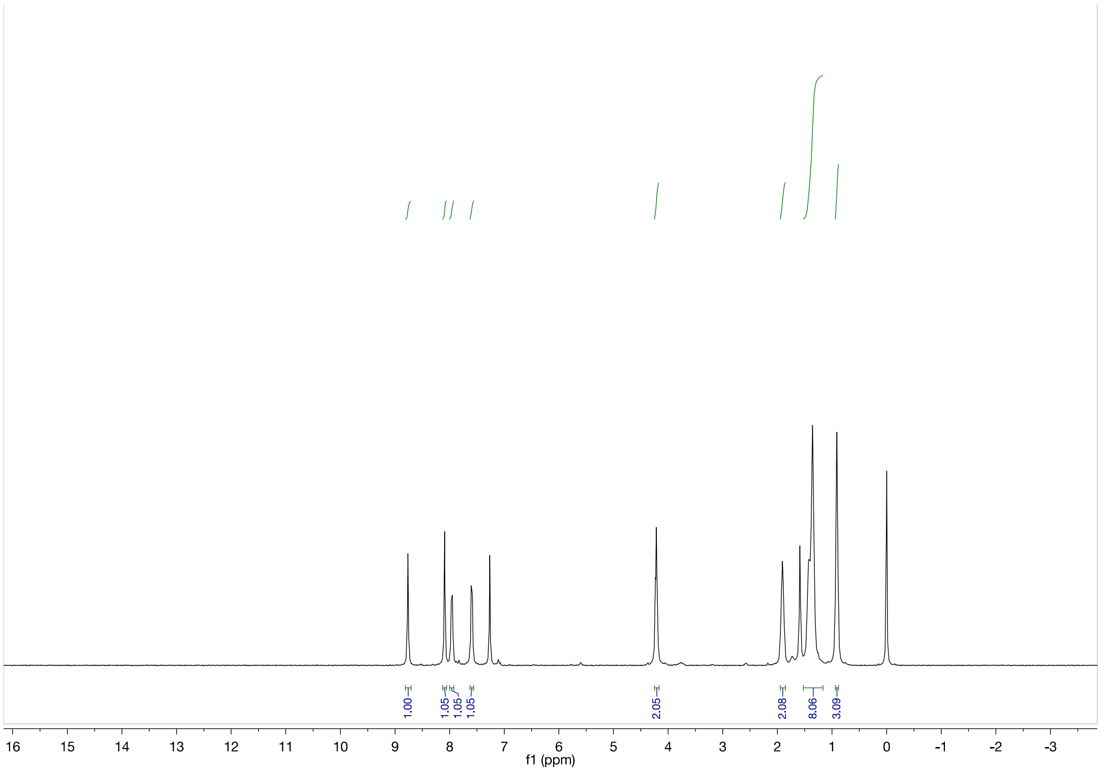

^{13}C NMR for 1-Hexyl-1,2-dihydroquinoline-3,6-dicarbonitrile (**12b**)

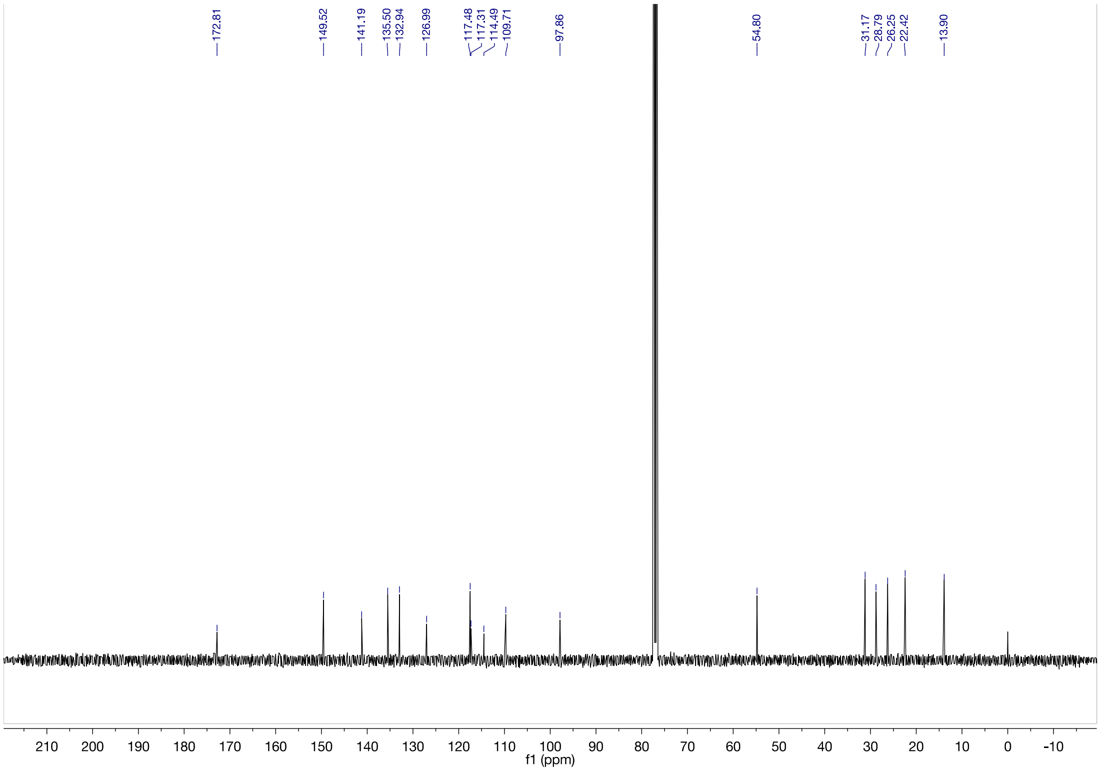

^{1}H NMR for 1-Isobutyl-1,2-dihydroquinoline-3,6-dicarbonitrile (**12c**)

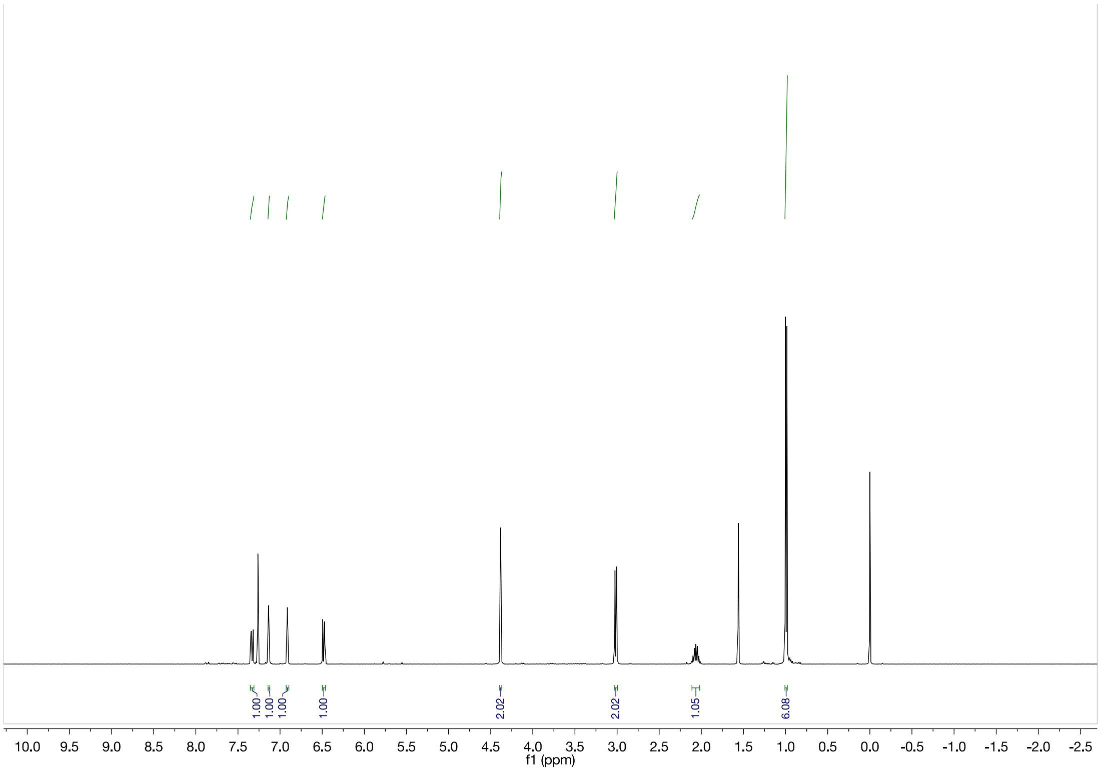

^{13}C NMR for 1-Isobutyl-1,2-dihydroquinoline-3,6-dicarbonitrile (**12c**)

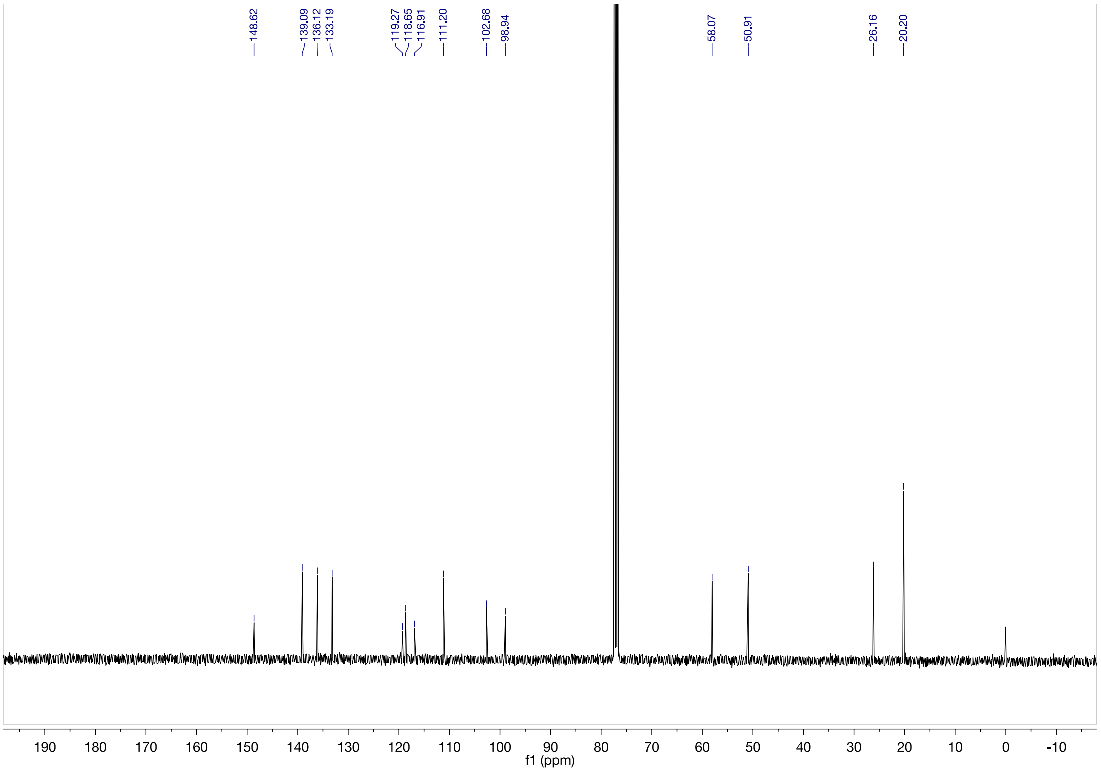

^{1}H NMR for 1-Benzyl-1,2-dihydroquinoline-3,6-dicarbonitrile (**12d**)

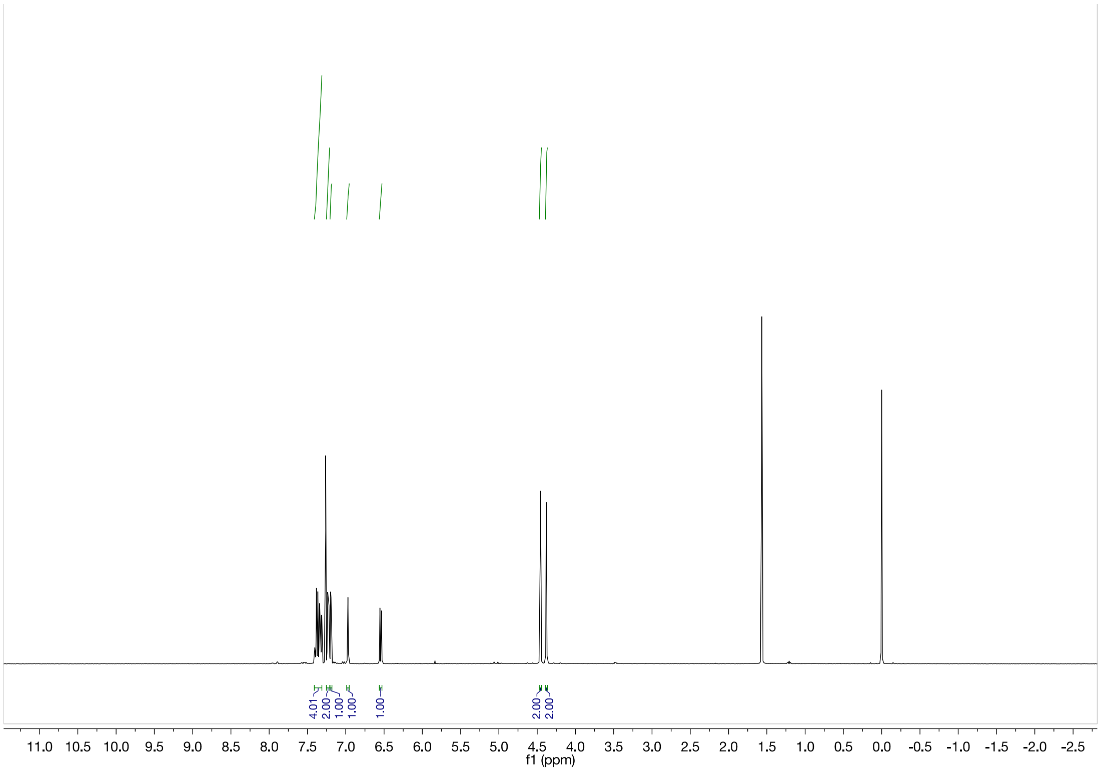

^{13}C NMR for 1-Benzyl-1,2-dihydroquinoline-3,6-dicarbonitrile (**12d**)

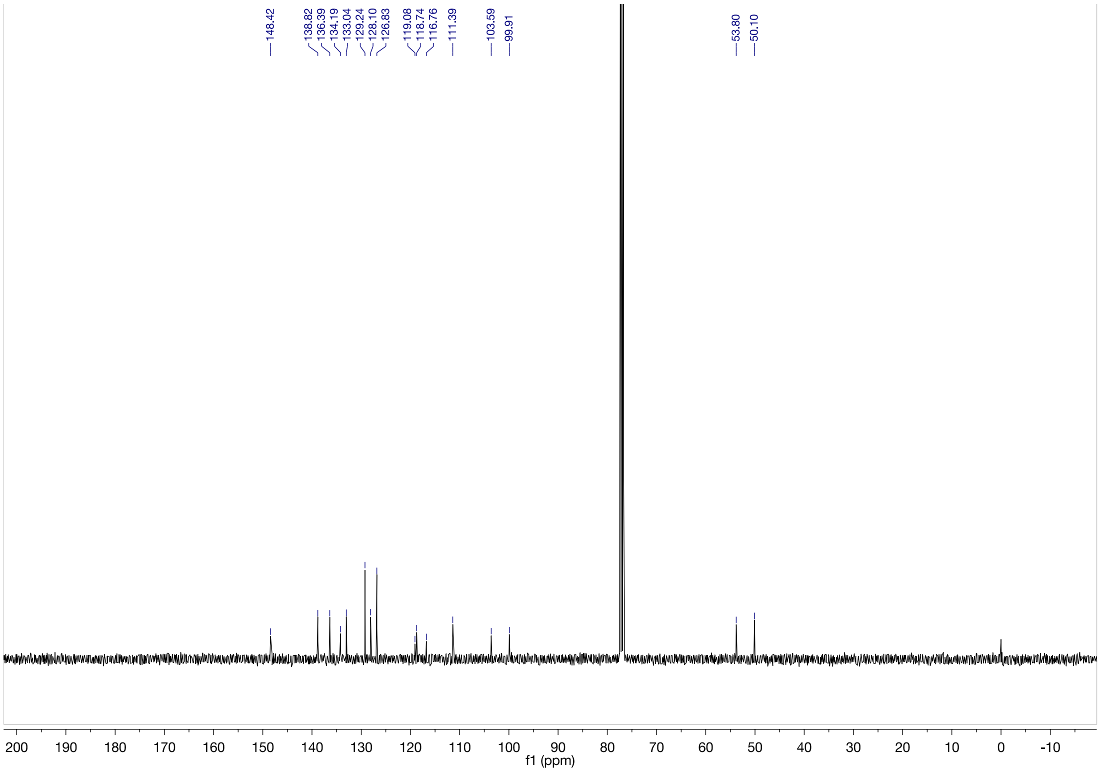

^{1}H NMR for 1-Phenethyl-1,2-dihydroquinoline-3,6-dicarbonitrile (**12e**)

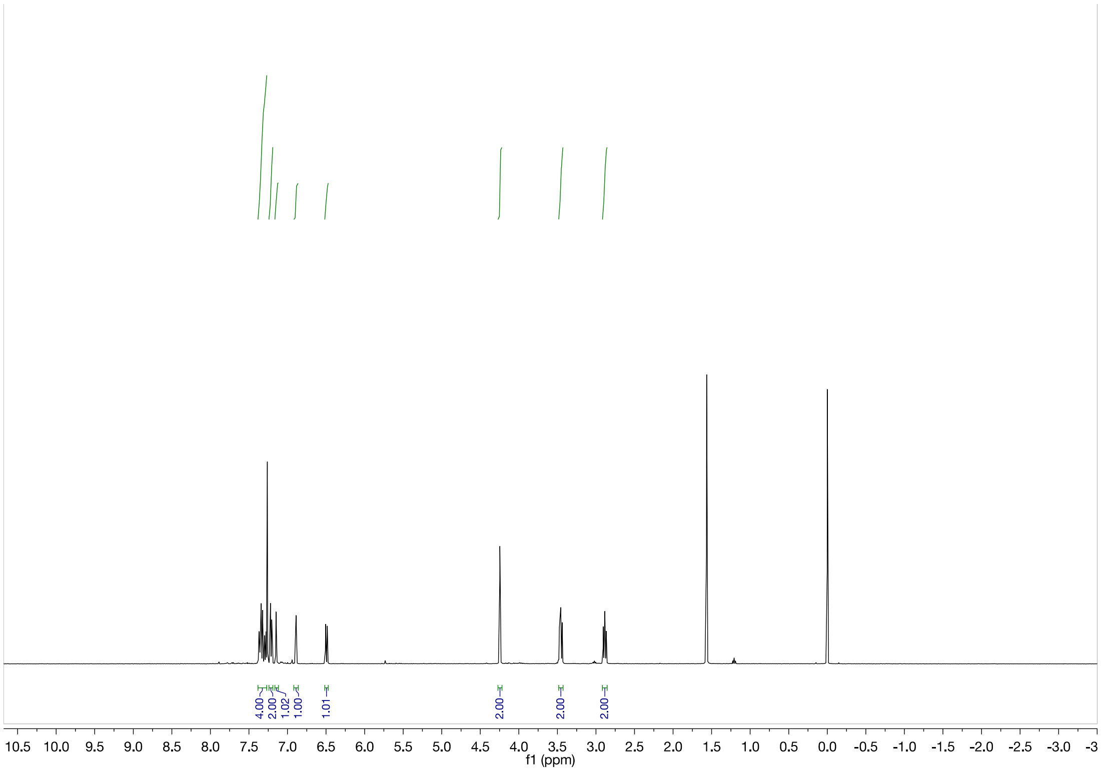

^{13}C NMR for 1-Phenethyl-1,2-dihydroquinoline-3,6-dicarbonitrile (**12e**)

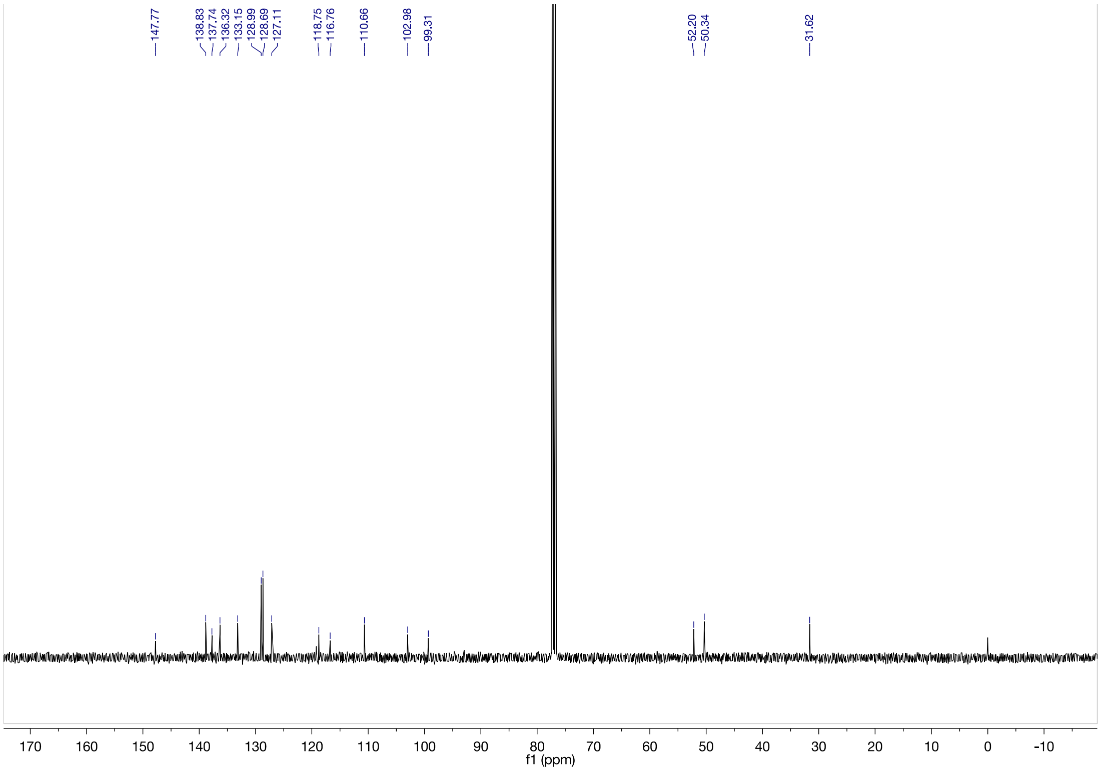

^{1}H NMR for Ethyl 1-methyl-1,2-dihydro-1,8-naphthyridine-3-carboxylate (**13a**)

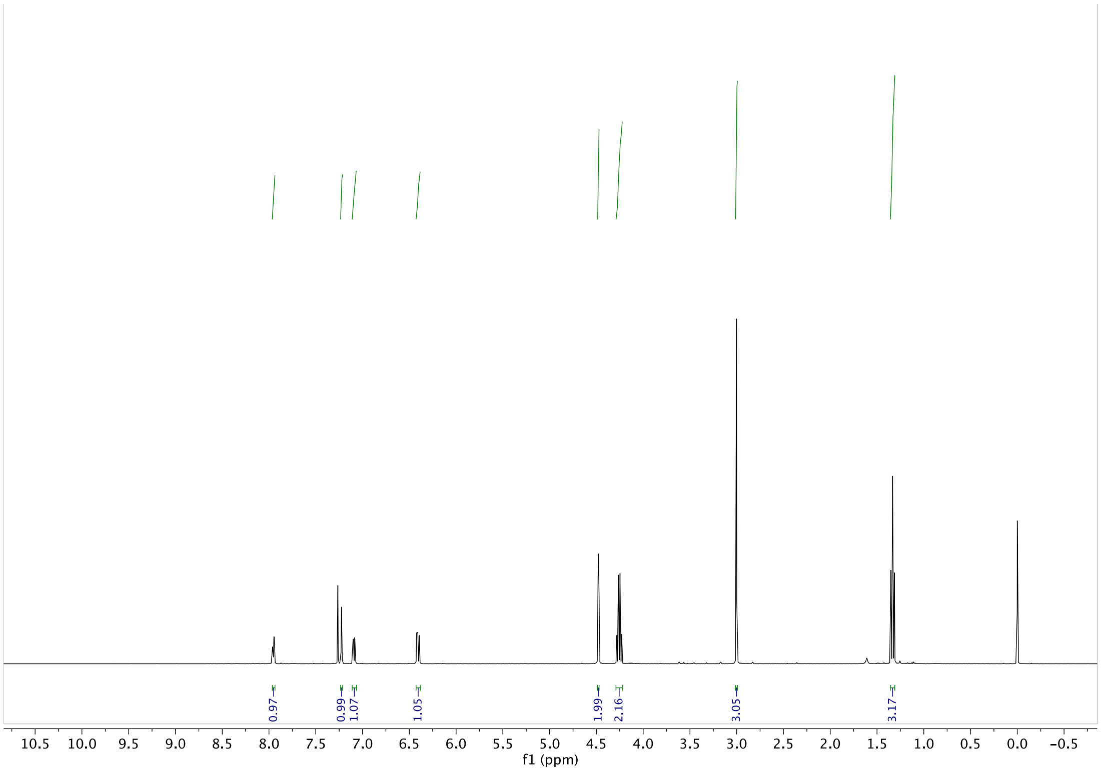

^{13}C NMR for Ethyl 1-methyl-1,2-dihydro-1,8-naphthyridine-3-carboxylate (**13a**)

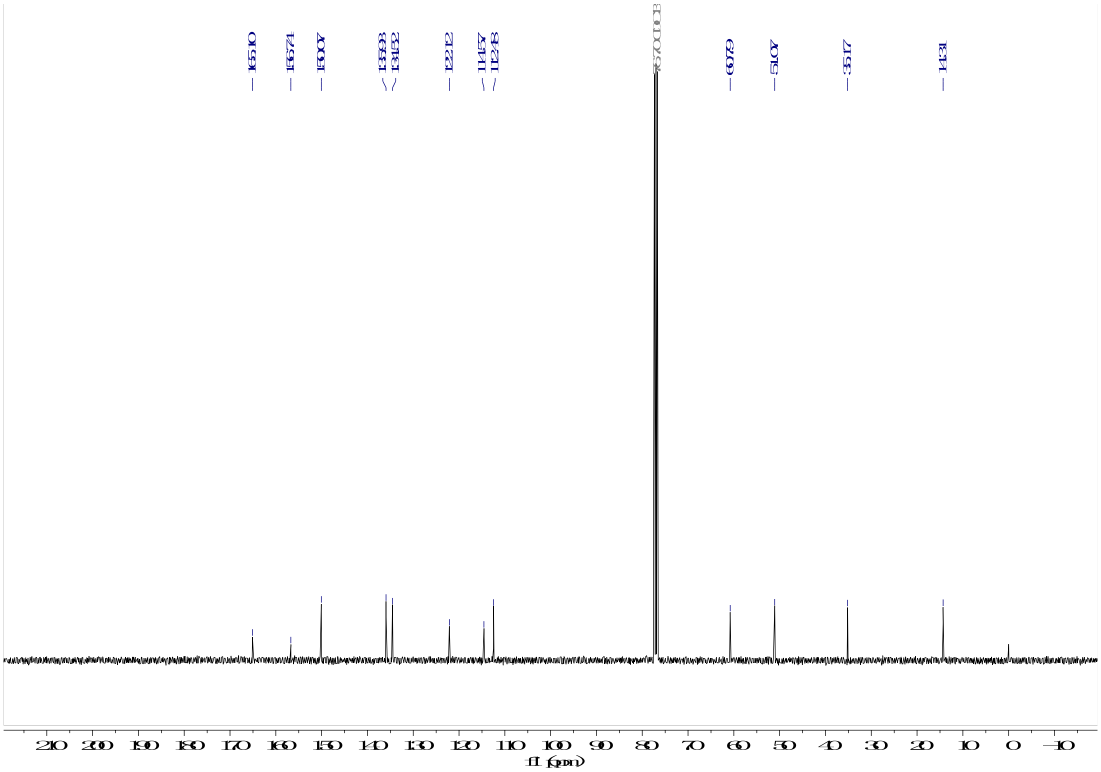

^{1}H NMR for Ethyl 1-hexyl-1,2-dihydro-1,8-naphthyridine-3-carboxylate (**13b**)

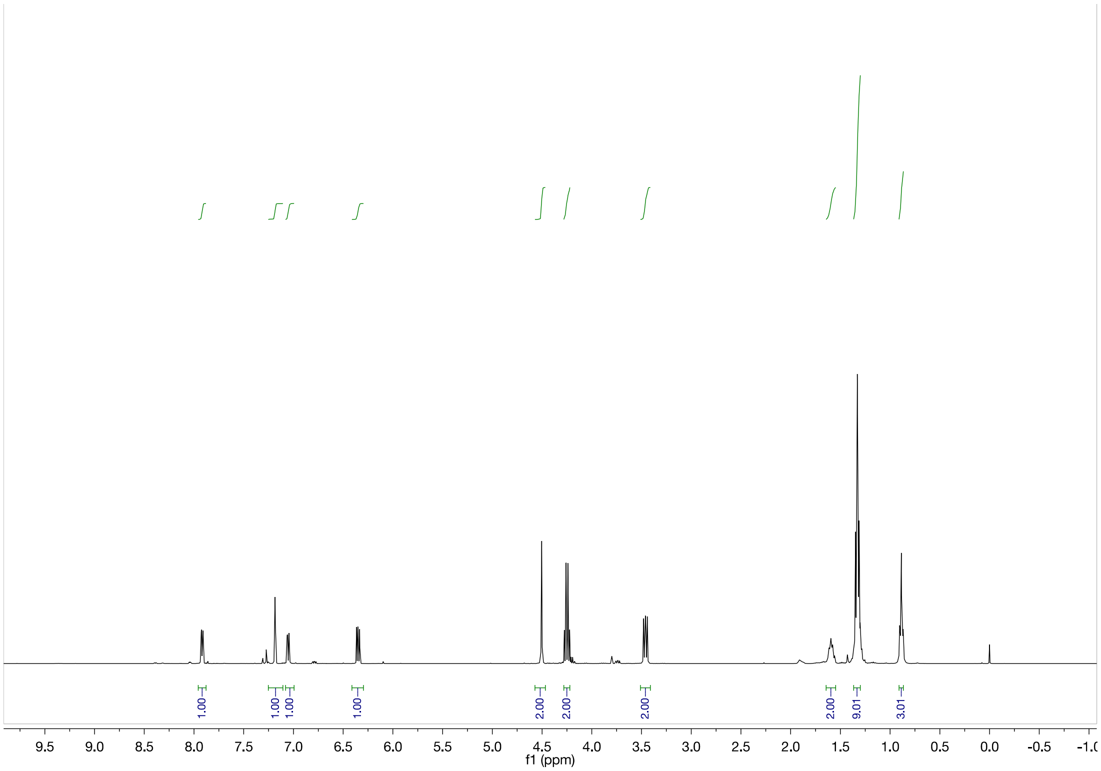

^{13}C NMR for Ethyl 1-hexyl-1,2-dihydro-1,8-naphthyridine-3-carboxylate (**13b**)

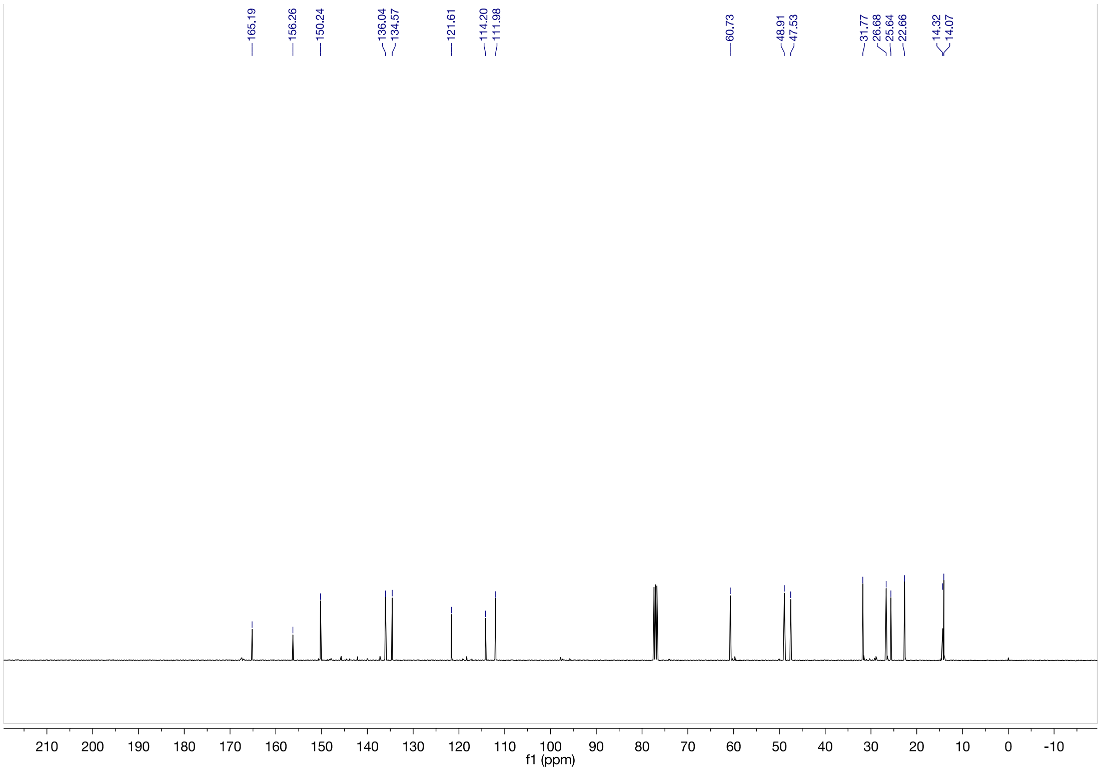

^{1}H NMR for Ethyl 1-isobutyl-1,2-dihydro-1,8-naphthyridine-3-carboxylate (**13c**)

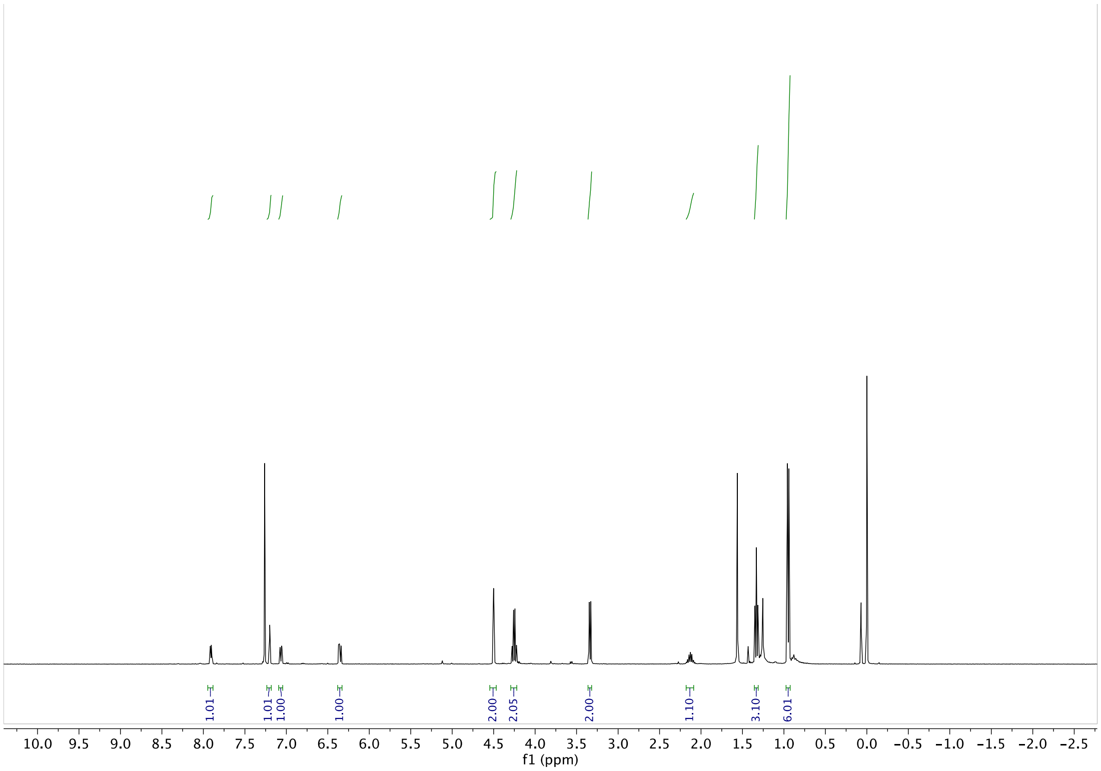

^{13}C NMR for Ethyl 1-isobutyl-1,2-dihydro-1,8-naphthyridine-3-carboxylate (**13c**)

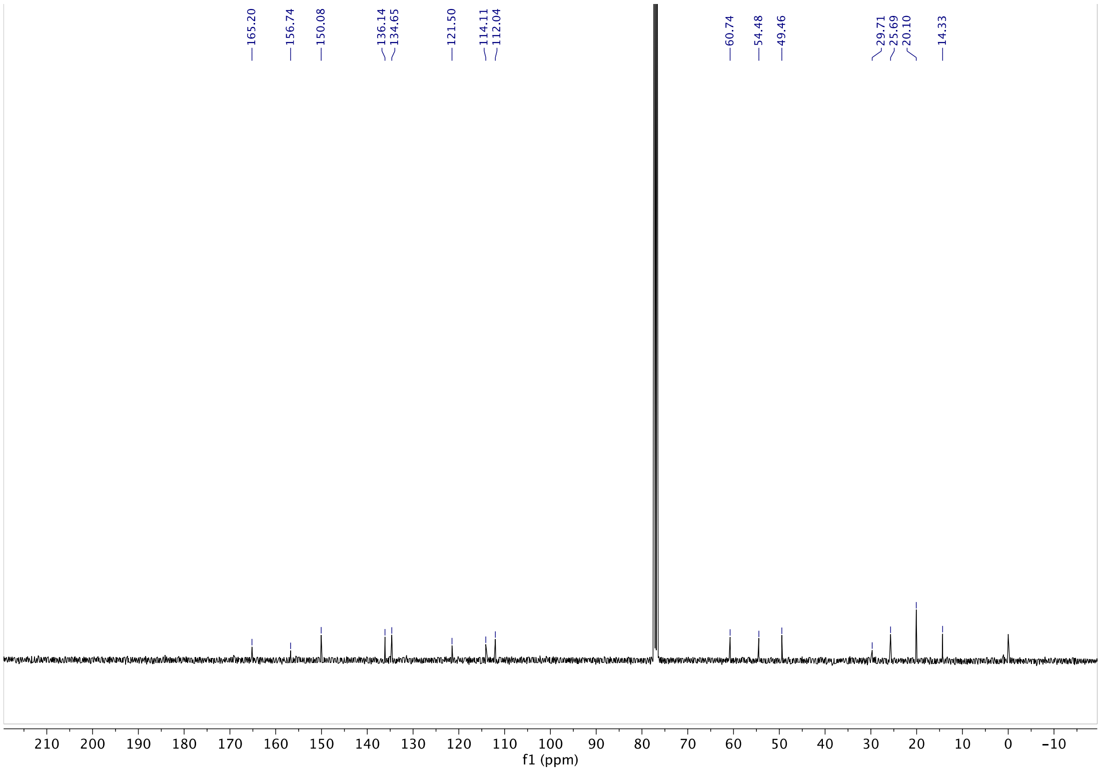

^{1}H NMR for Ethyl 1-benzyl-1,2-dihydro-1,8-naphthyridine-3-carboxylate (**13d**)

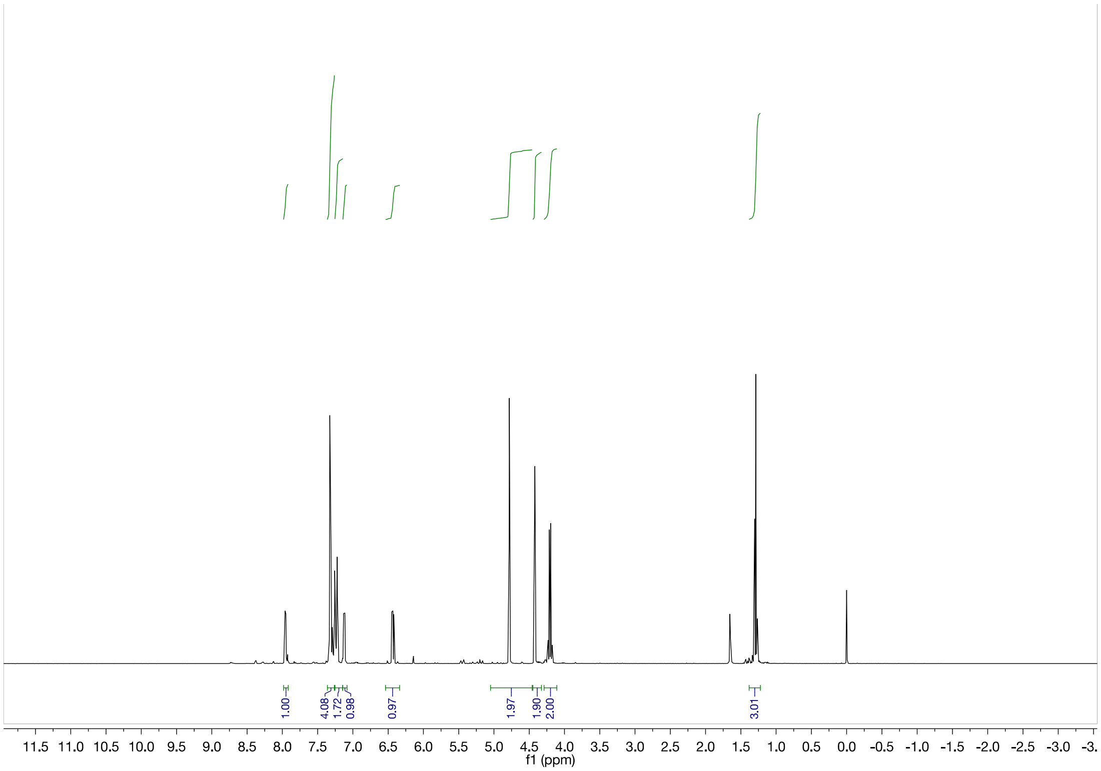

^{13}C NMR for Ethyl 1-benzyl-1,2-dihydro-1,8-naphthyridine-3-carboxylate (**13d**)

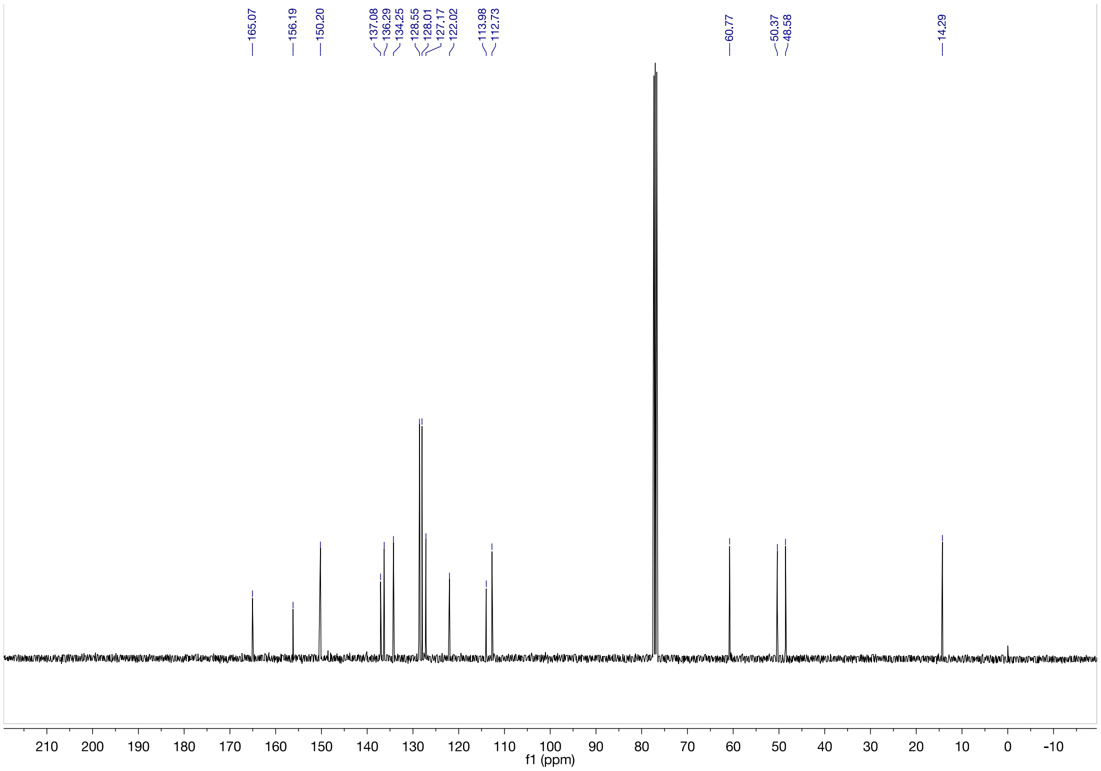

^{1}H NMR for Ethyl 1-phenethyl-1,2-dihydro-1,8-naphthyridine-3-carboxylate (**13e**)

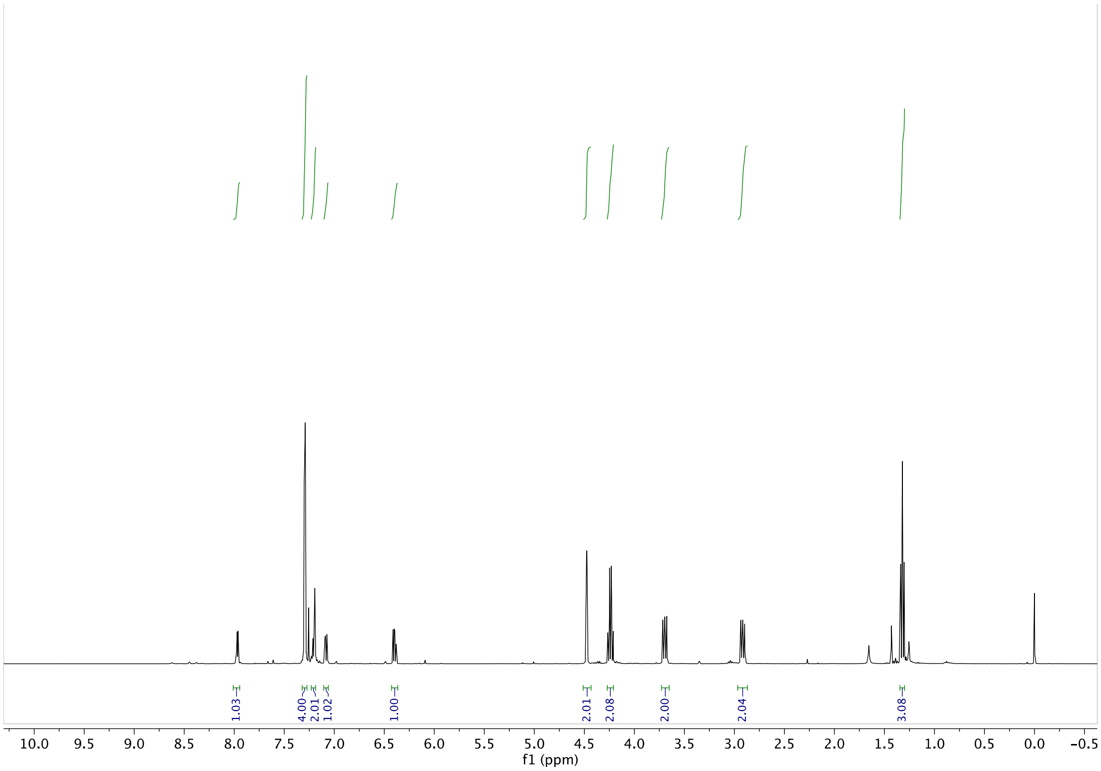

^{13}C NMR for Ethyl 1-phenethyl-1,2-dihydro-1,8-naphthyridine-3-carboxylate (**13e**)

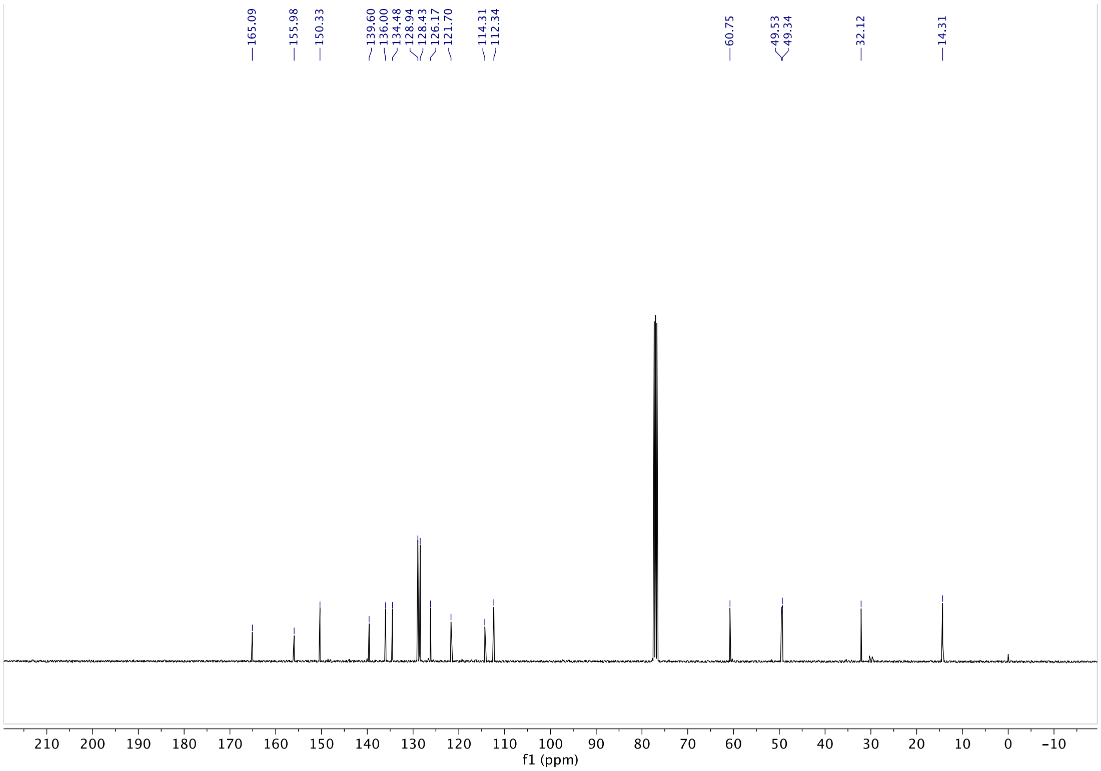

^{1}H NMR for Ethyl 1-phenyl-1,2-dihydro-1,8-naphthyridine-3-carboxylate (**13f**)

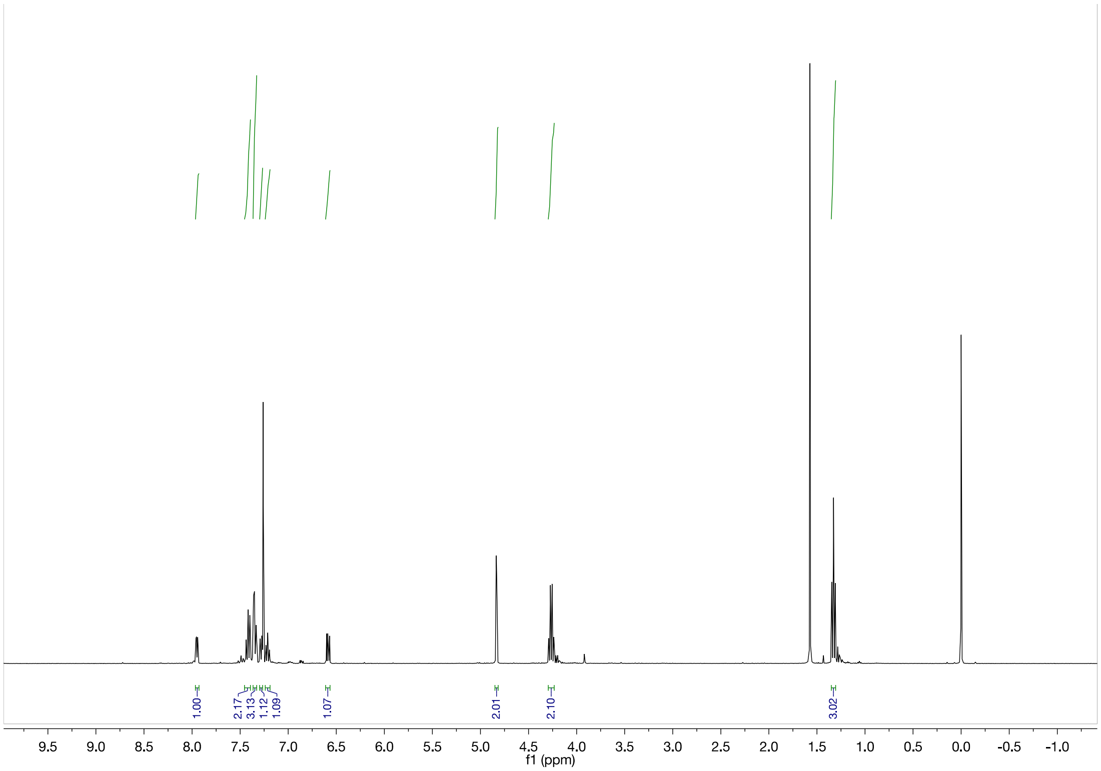

^{13}C NMR for Ethyl 1-phenyl-1,2-dihydro-1,8-naphthyridine-3-carboxylate (**13f**)

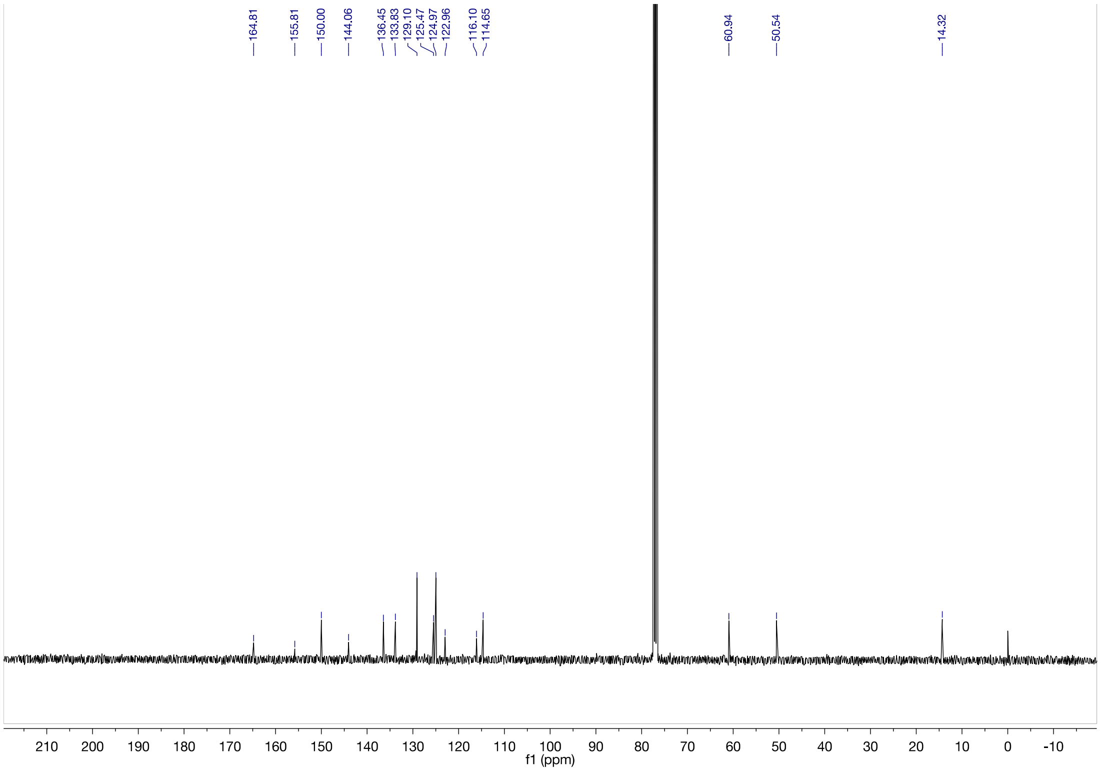

^1H NMR for 1-Hexyl-1,2-dihydro-1,8-naphthyridine-3-carbonitrile (**14b**)

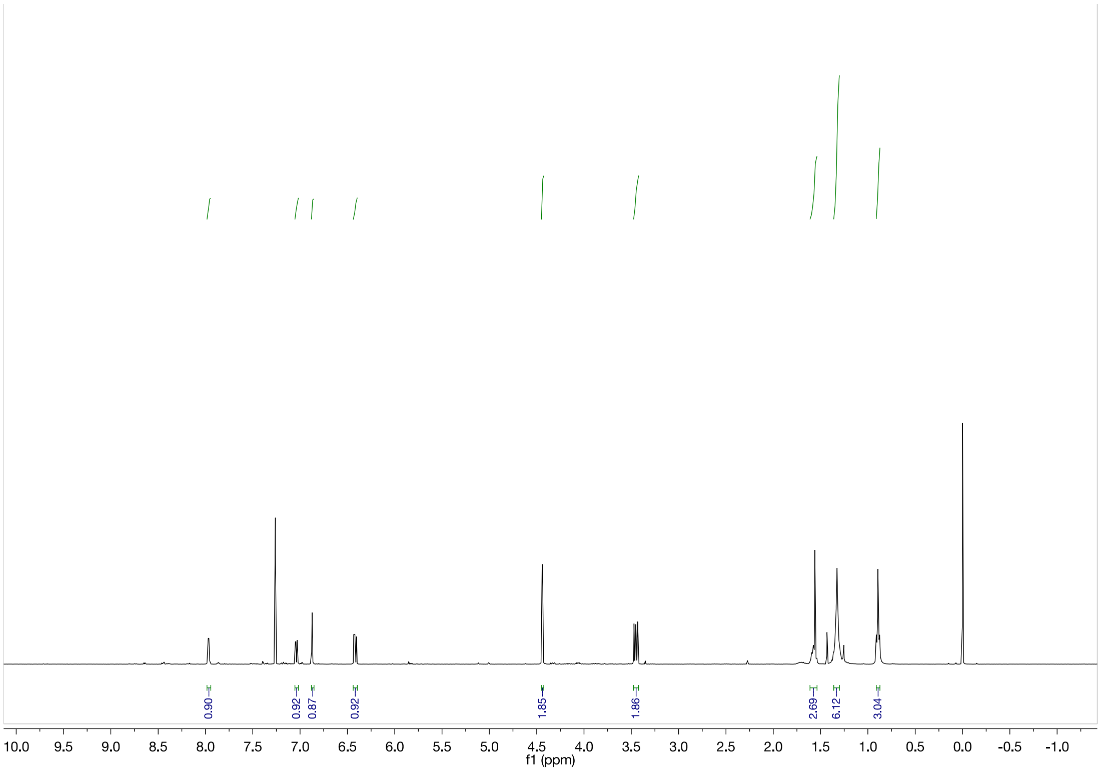

^{13}C NMR for 1-Hexyl-1,2-dihydro-1,8-naphthyridine-3-carbonitrile (**14b**)

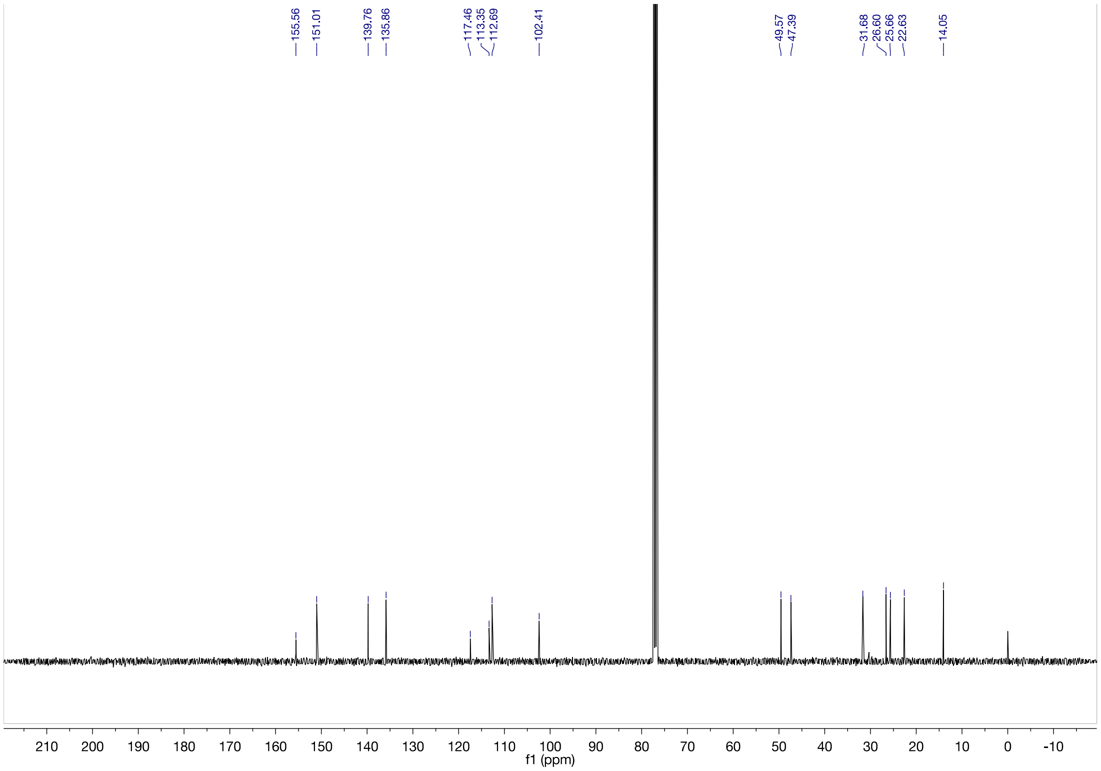

^{1}H NMR for 1-Benzyl-1,2-dihydro-1,8-naphthyridine-3-carbonitrile (**14d**)

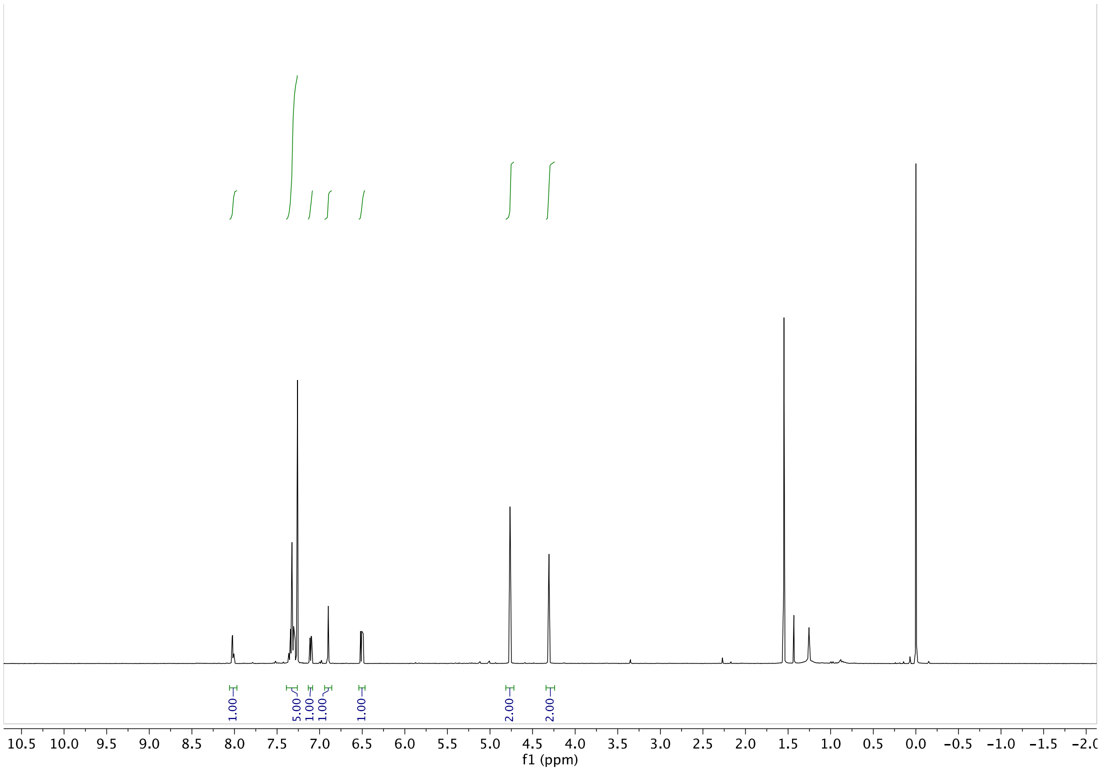

^{13}C NMR for 1-Benzyl-1,2-dihydro-1,8-naphthyridine-3-carbonitrile (**14d**)

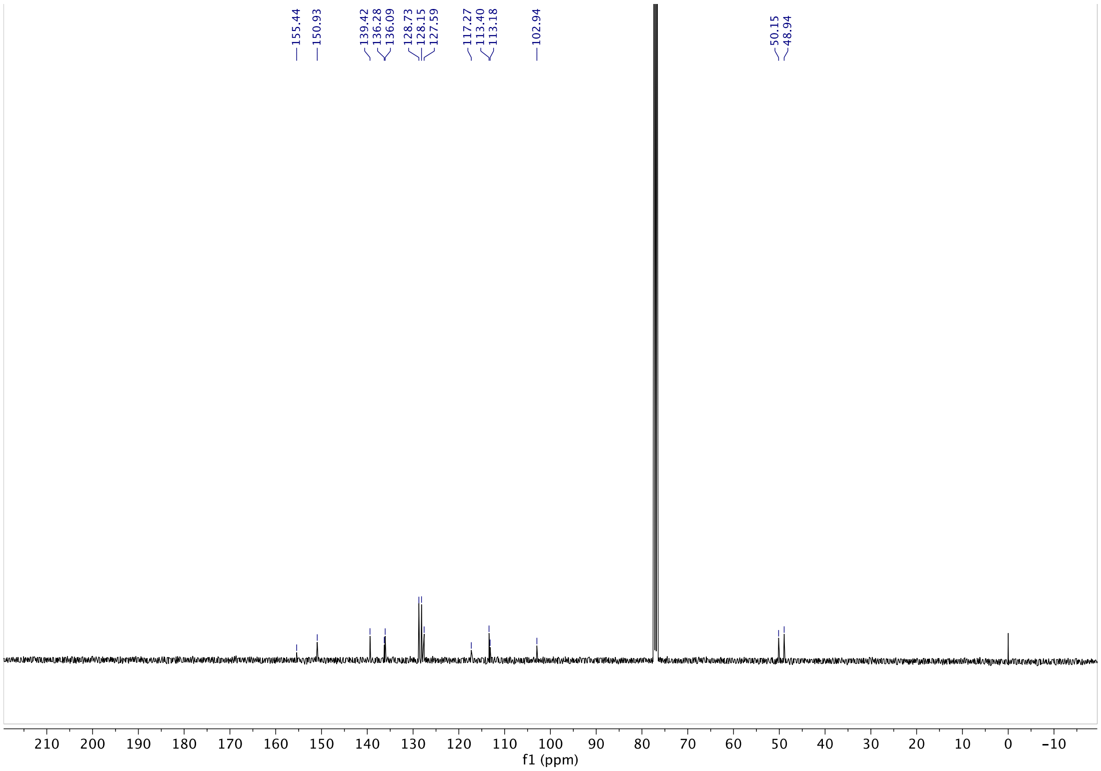

^{1}H NMR for 1-Phenethyl-1,2-dihydro-1,8-naphthyridine-3-carbonitrile **(14e)**

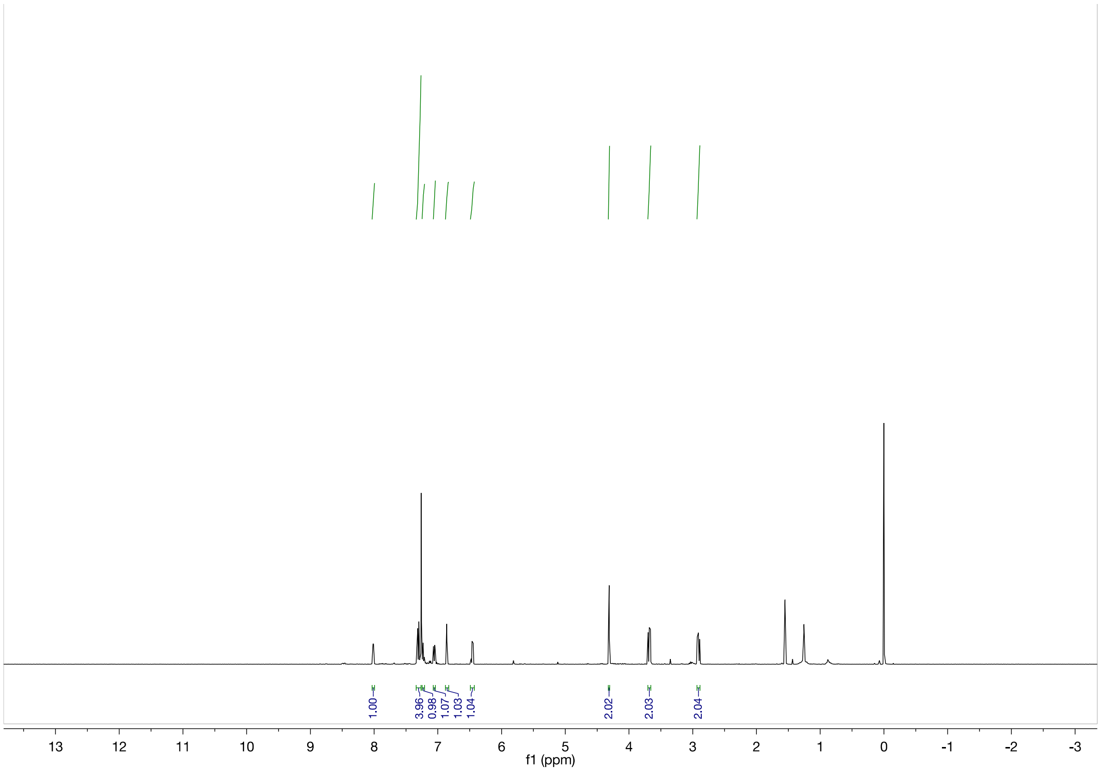

^{13}C NMR for 1-Phenethyl-1,2-dihydro-1,8-naphthyridine-3-carbonitrile **(14e)**

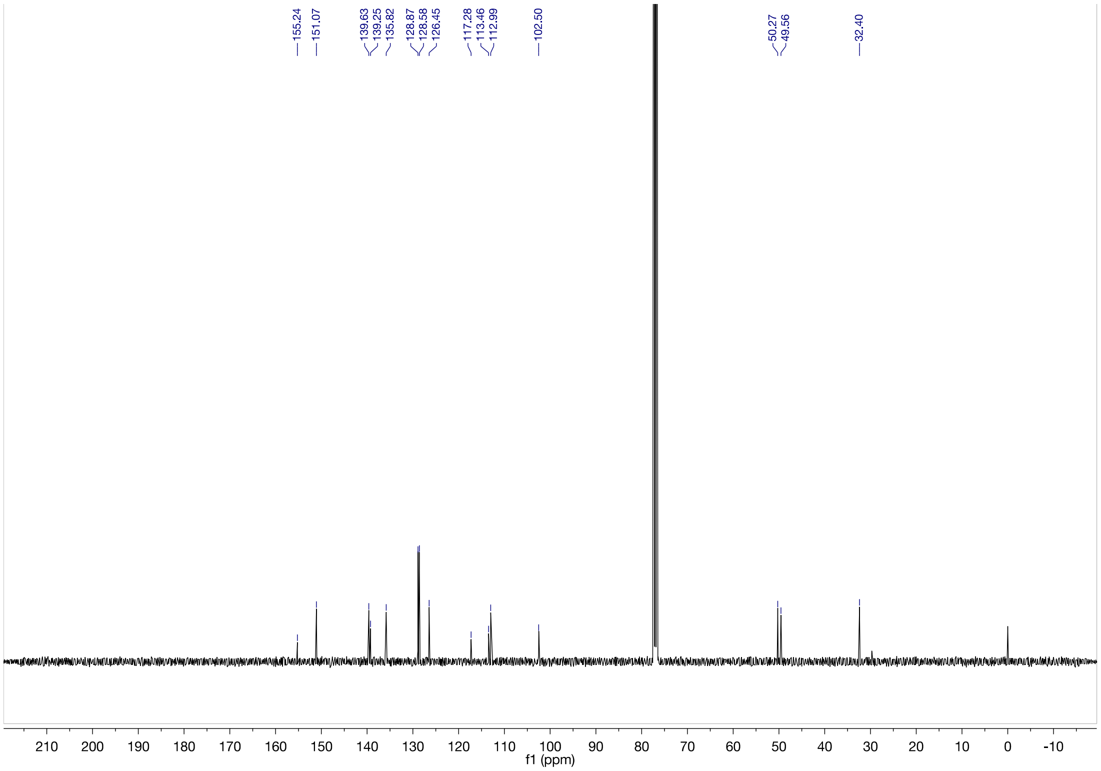

Unexpected double bond migrated products

^{1}H NMR for 1-Benzyl-1,4-dihydroquinoline-3-carbonitrile (**15d**)

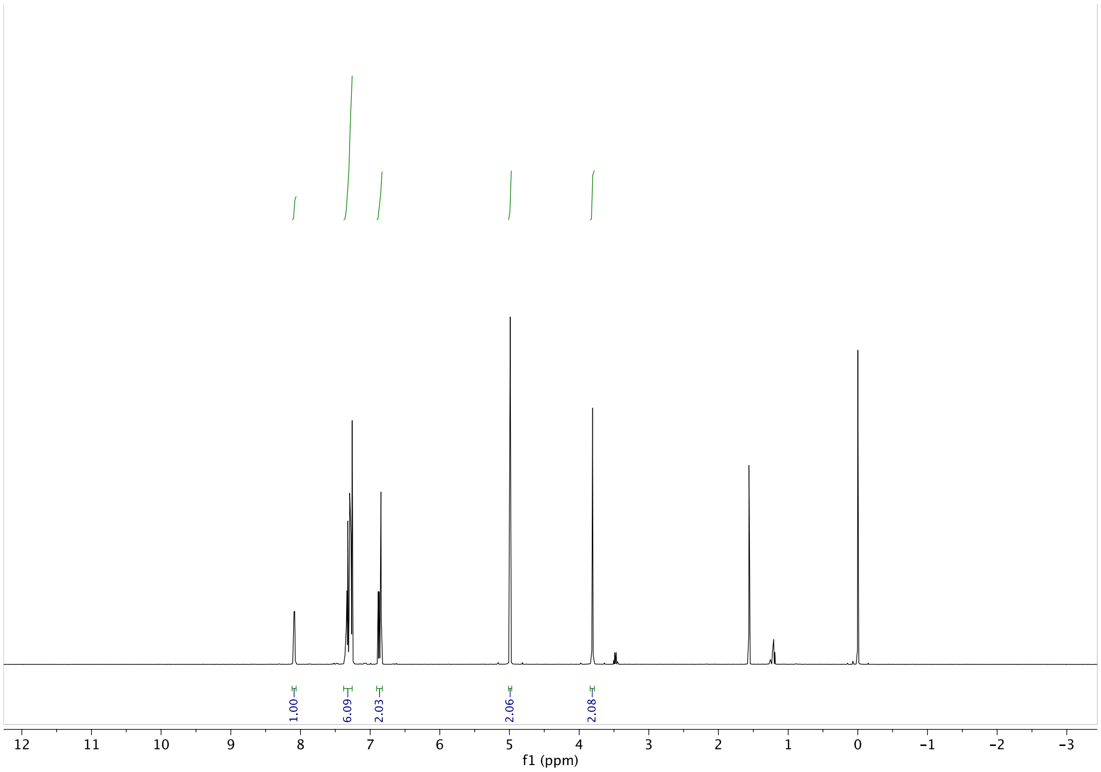

^{13}C NMR for 1-Benzyl-1,4dihydroquinoline-3-carbonitrile (**15d**)

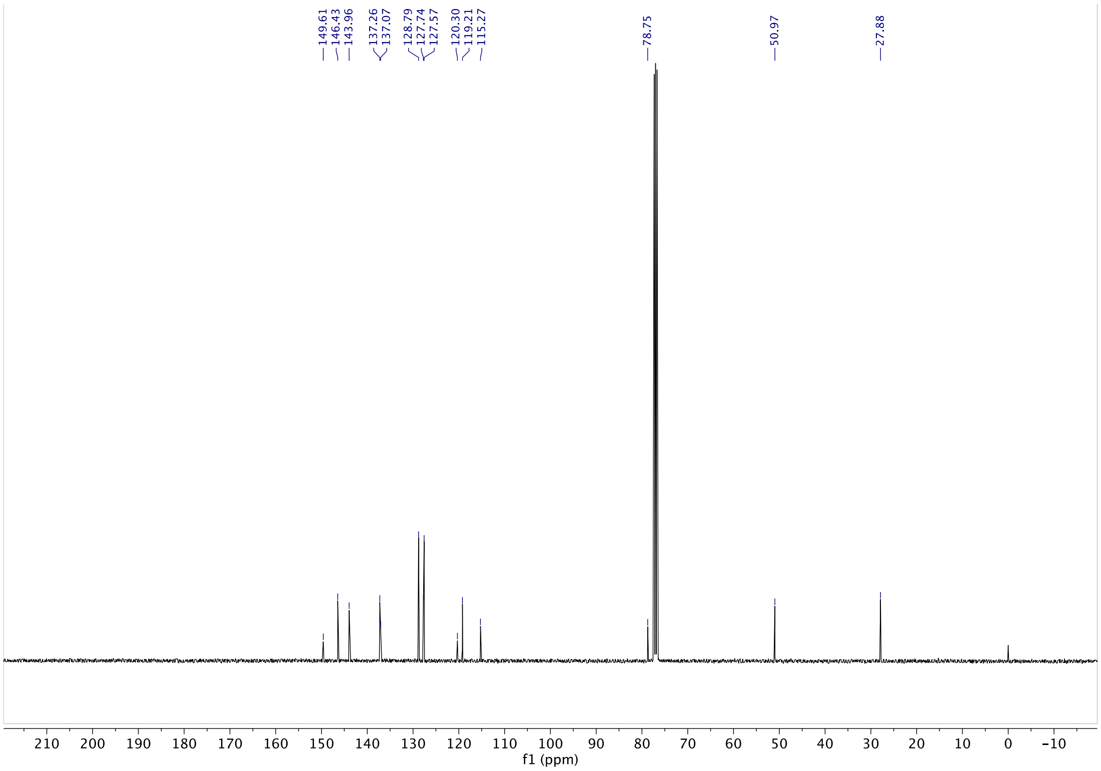

^{1}H NMR for 1-Phenyl-1,4-dihydroquinoline-3-carbonitrile (**15f**)

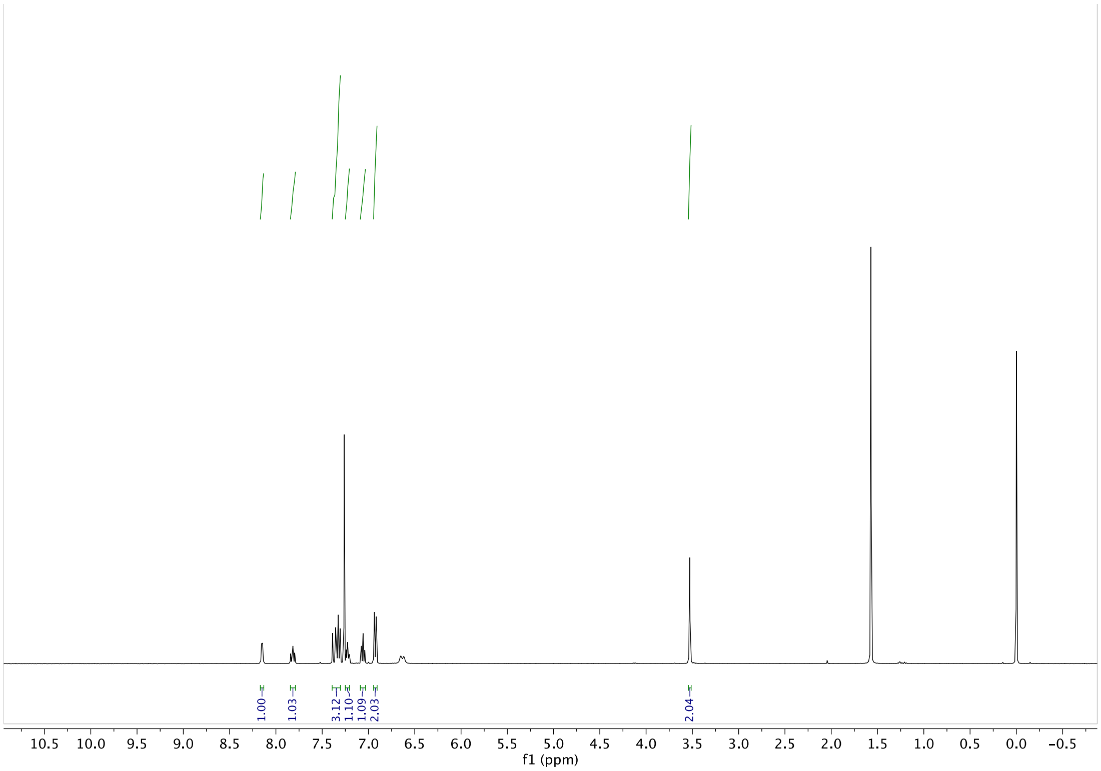

^{13}C NMR for 1-Phenyl-1,4-dihydroquinoline-3-carbonitrile (**15f**)

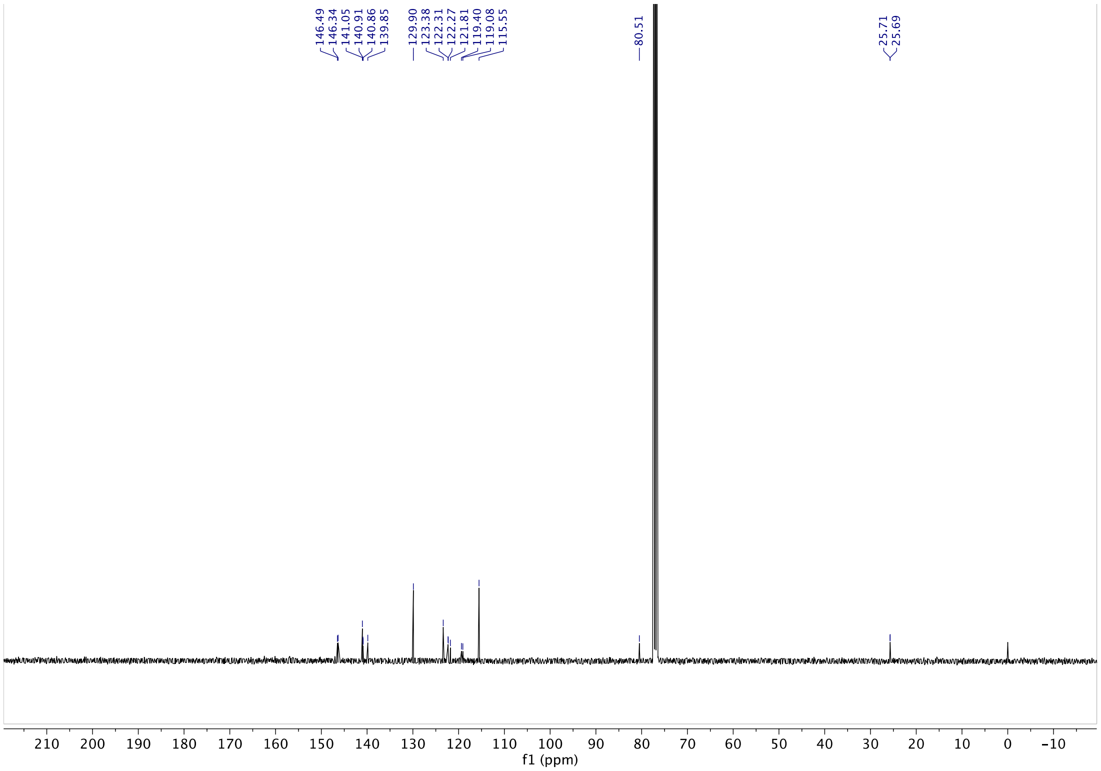

^{1}H NMR for (*Z*)-2-((Benzylamino)methyl)-3-(2-fluoro-5-nitrophenyl)acrylonitrile (**16**)

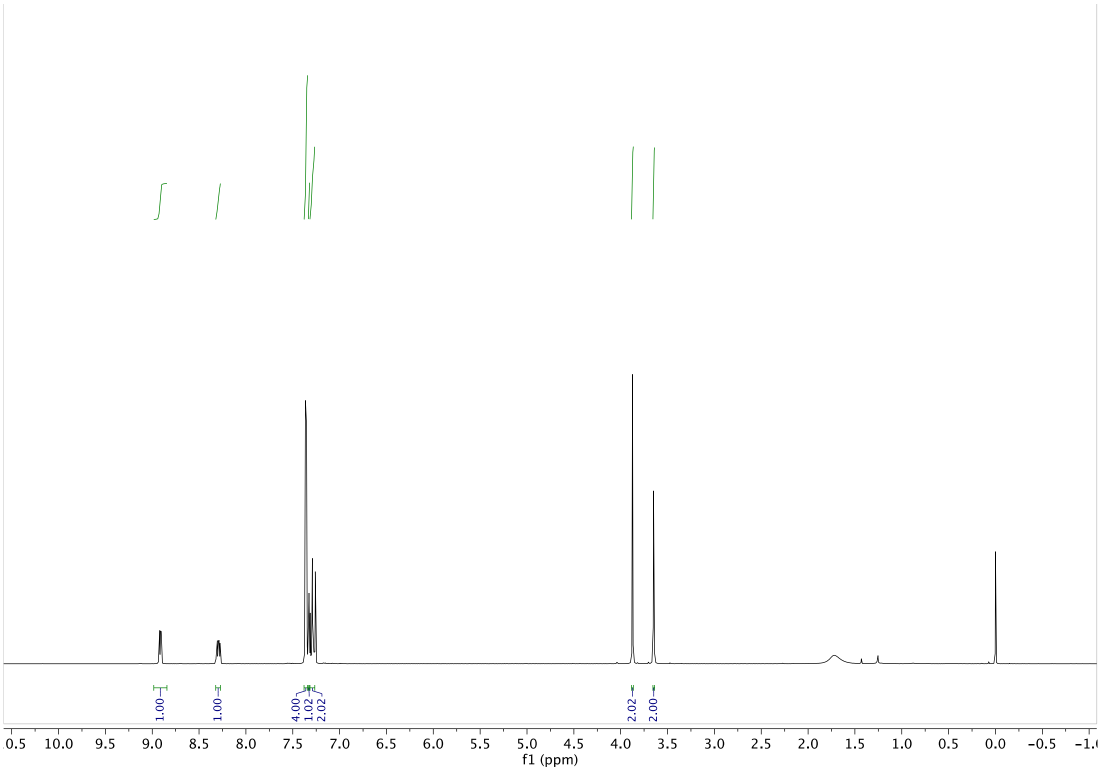

^{13}C NMR for (*Z*)-2-((Benzylamino)methyl)-3-(2-fluoro-5-nitrophenyl) acrylonitrile (**16**)

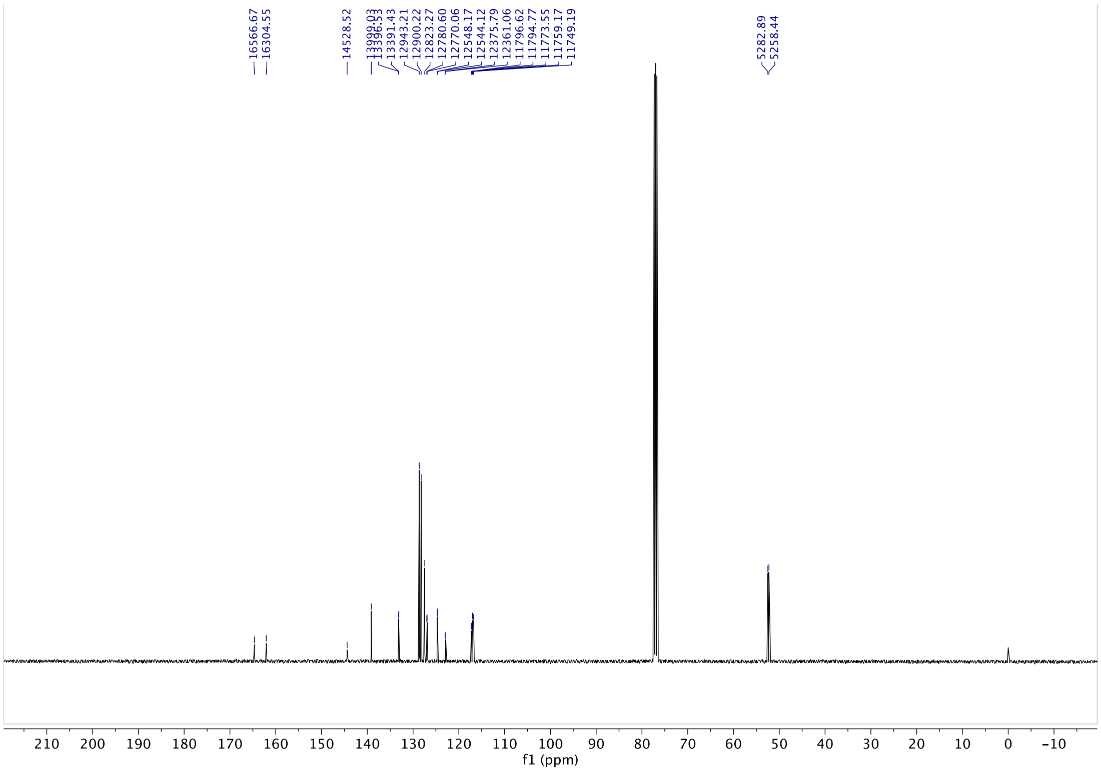

^{1}H NMR for Ethyl 6-fluoro-1-methyl-1,2-dihydroquinoline-3-carboxylate (**18a**)

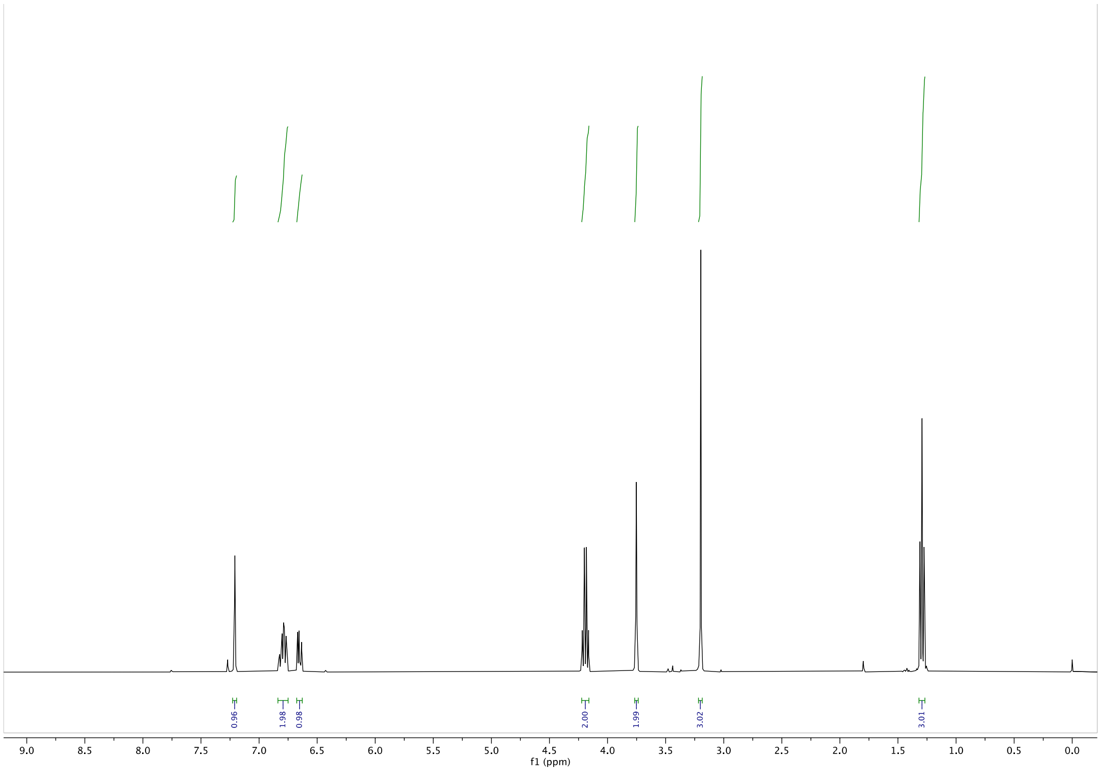

^{13}C NMR for Ethyl 6-fluoro-1-methyl-1,2-dihydroquinoline-3-carboxylate (**18a**)

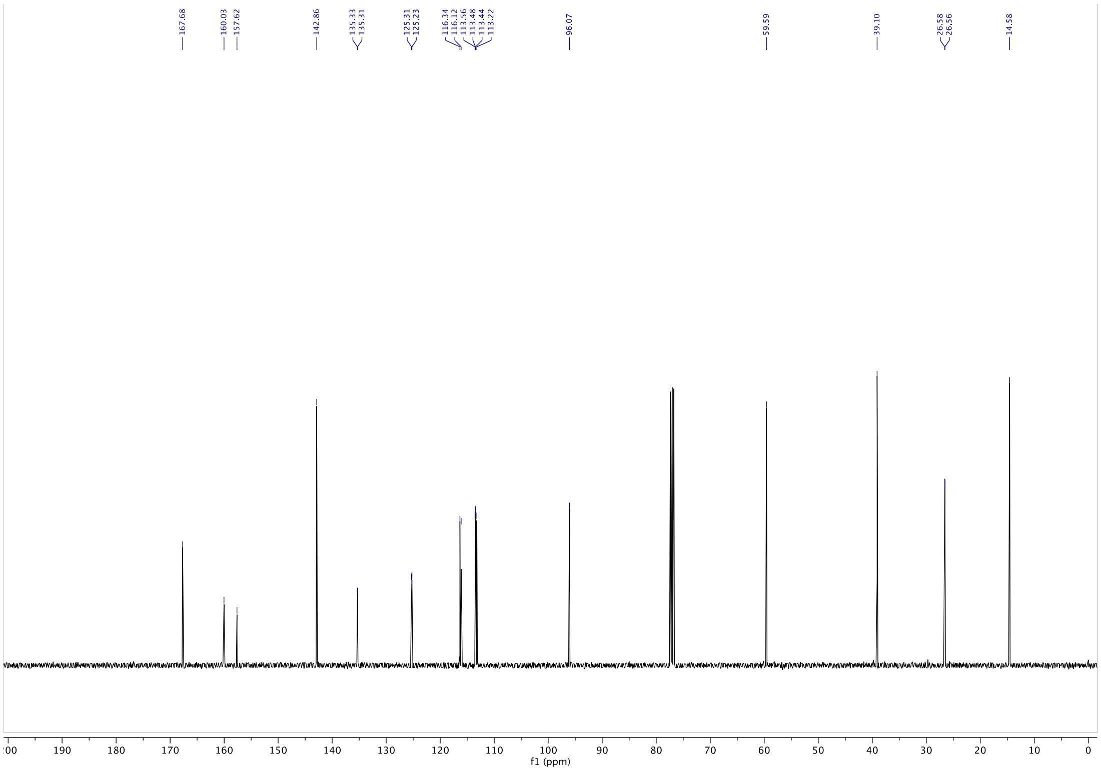

^{1}H NMR for Ethyl 6-fluoro-1-hexyl-1,2-dihydroquinoline-3-carboxylate (**18b**)

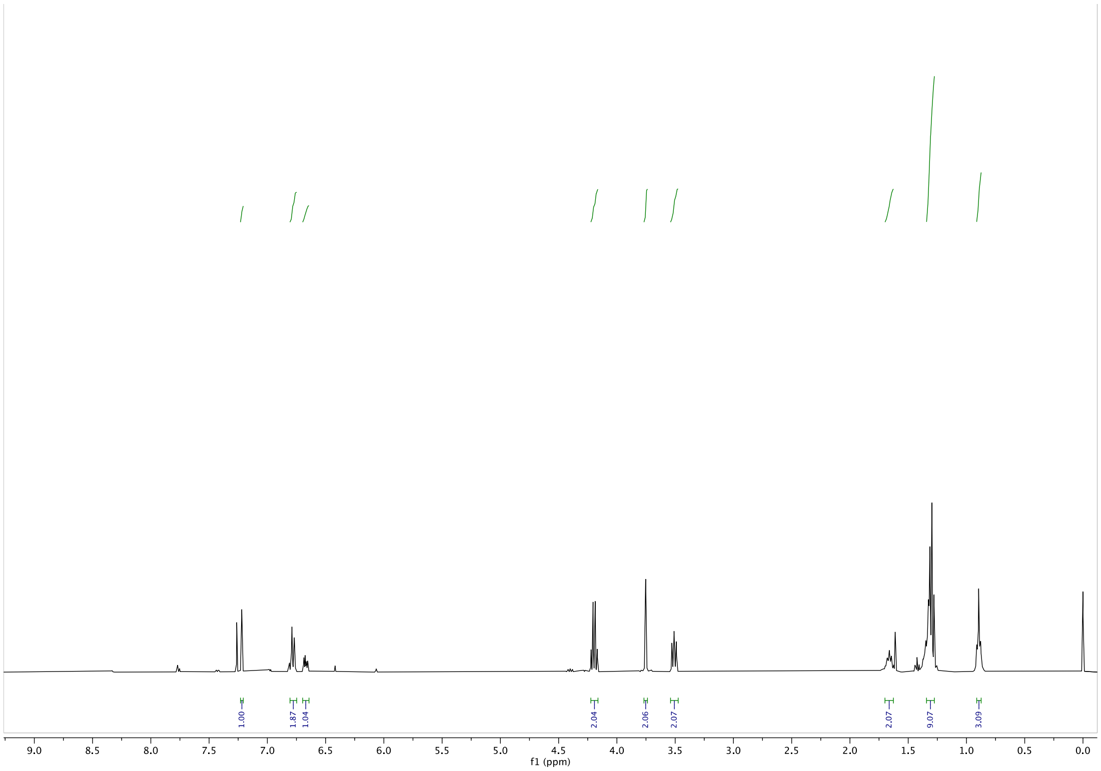

^{13}C NMR for Ethyl 6-fluoro-1-hexyl-1,2-dihydroquinoline-3-carboxylate (**18b**)

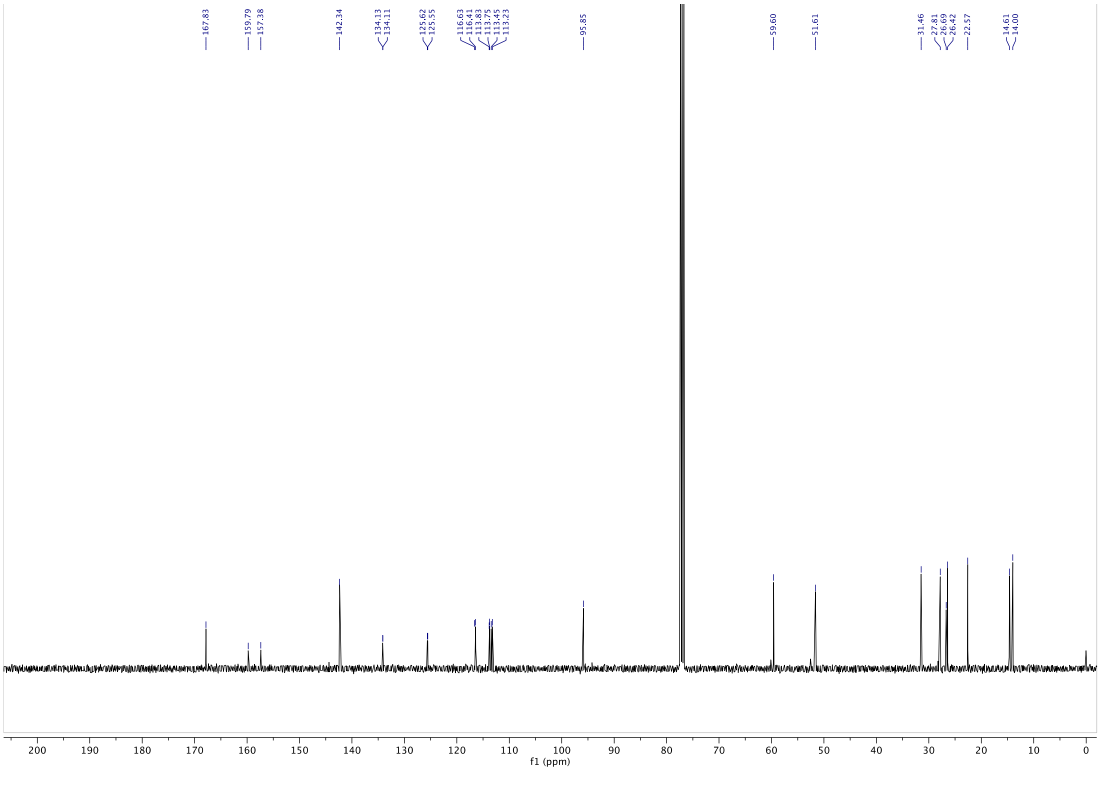

^{1}H NMR for Ethyl 6-fluoro-1-isobutyl-1,2-dihydroquinoline-3-carboxylate (**18c**)

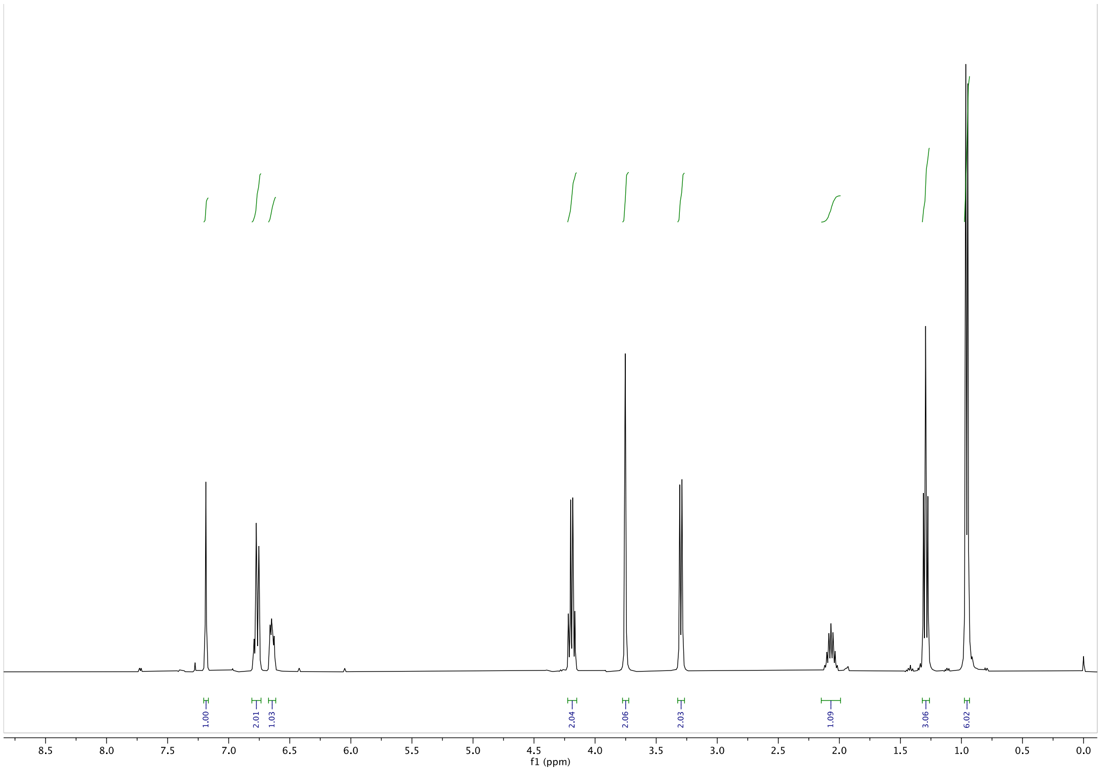

^{13}C NMR for Ethyl 6-fluoro-1-isobutyl-1,2-dihydroquinoline-3-carboxylate (**18c**)

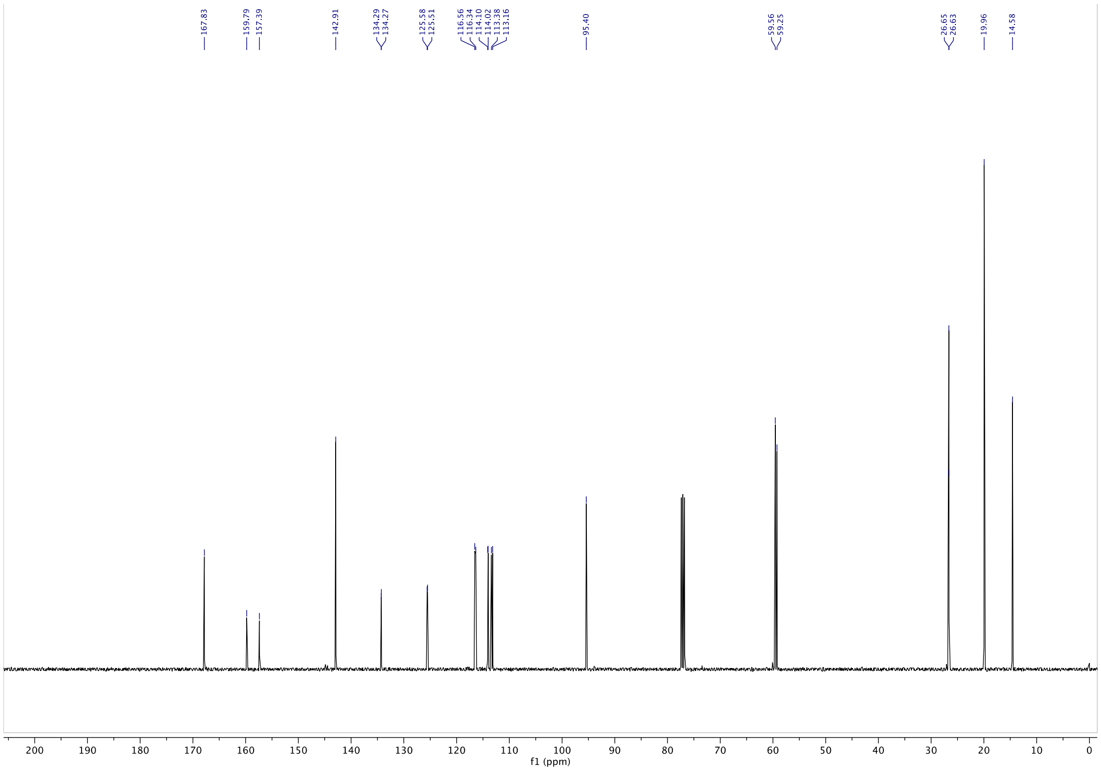

^{1}H NMR for Ethyl 1-benzyl-6-fluoro-1,2-dihydroquinoline-3-carboxylate (**18d**)

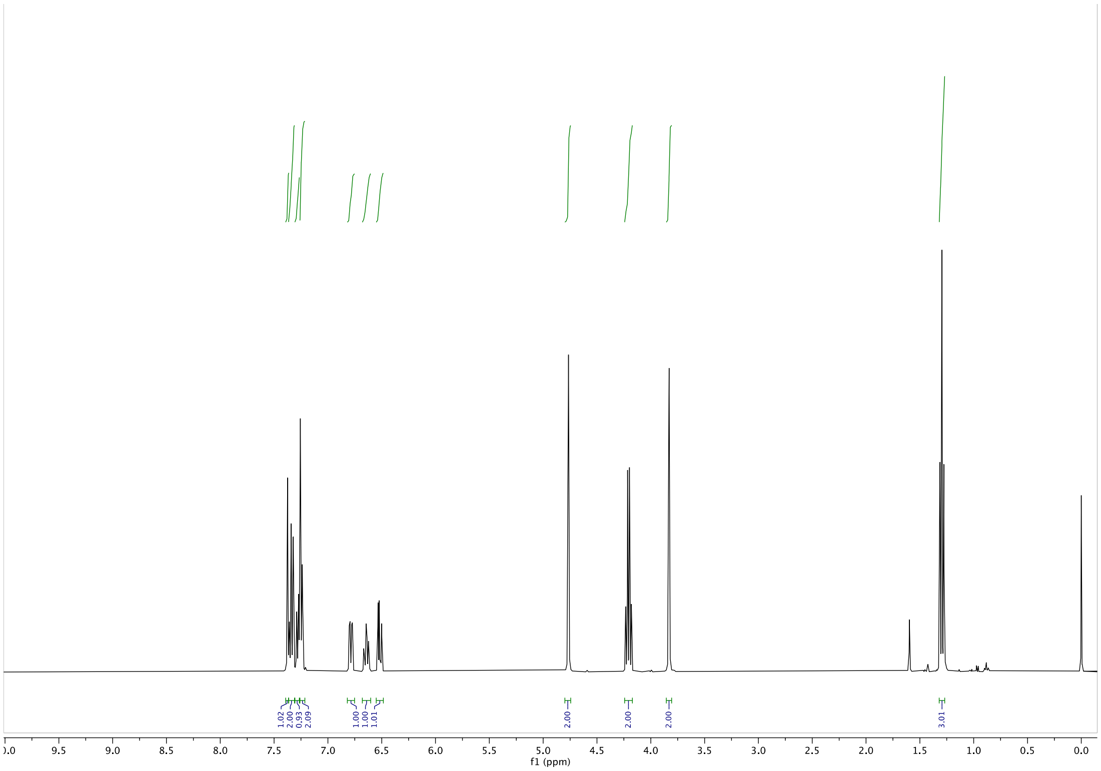

^{13}C NMR for Ethyl 1-benzyl-6-fluoro-1,2-dihydroquinoline-3-carboxylate (**18d**)

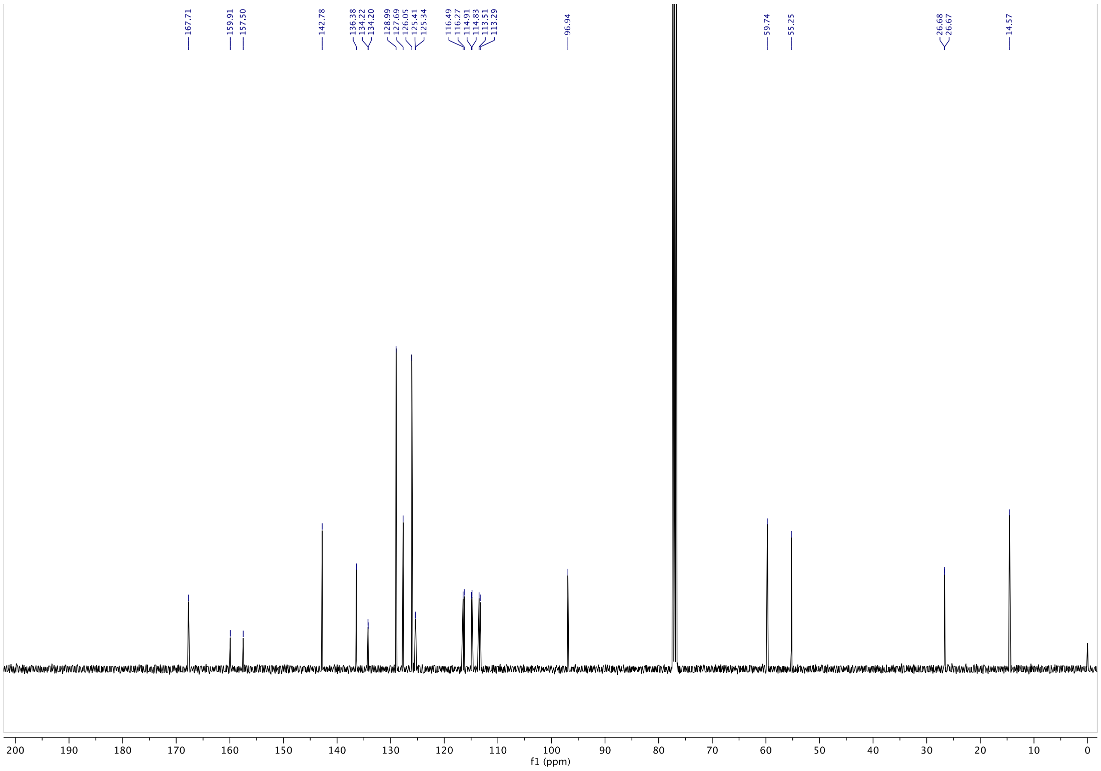

^{1}H NMR for Ethyl 6-fluoro-1-phenethyl-1,2-dihydroquinoline-3-carboxylate (**18e**)

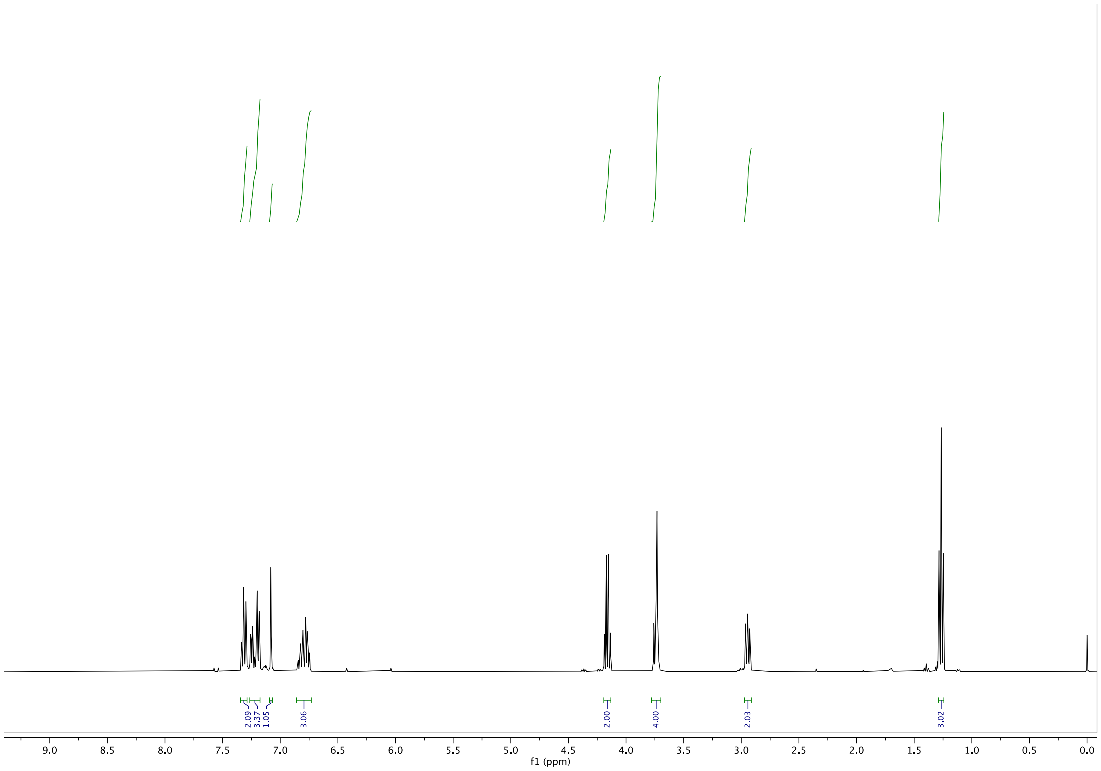

^{13}C NMR for Ethyl 6-fluoro-1-phenethyl-1,2-dihydroquinoline-3-carboxylate (**18e**)

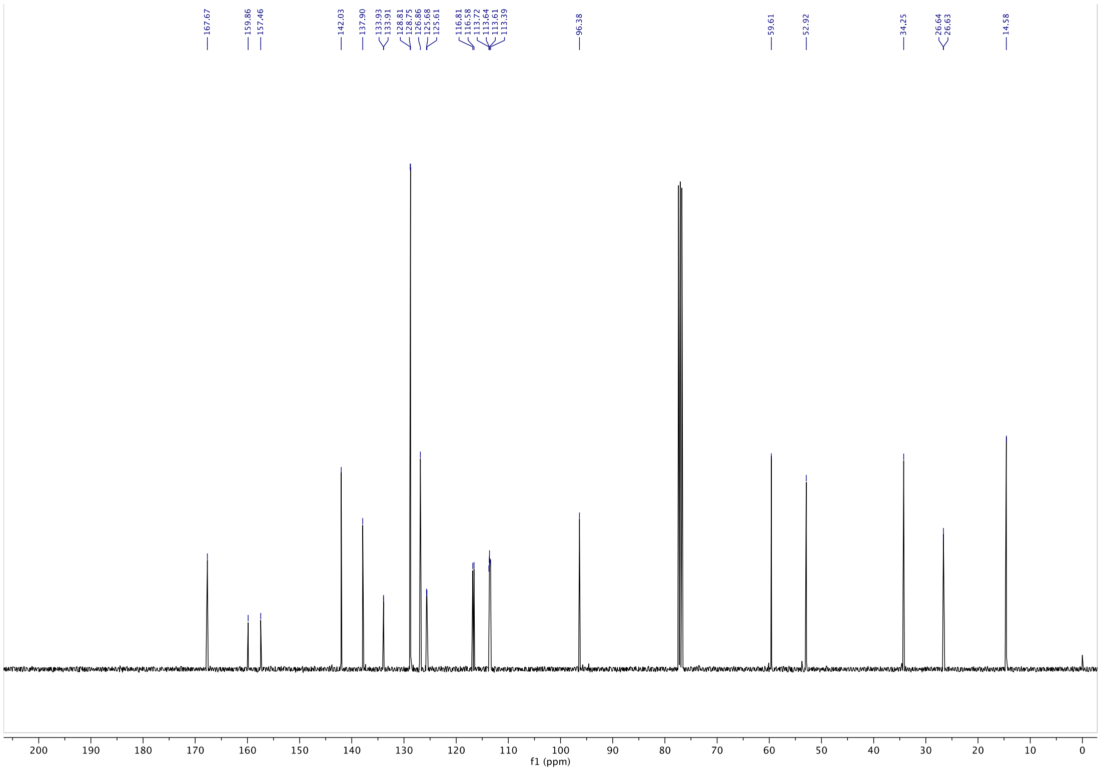

^{1}H NMR for Ethyl 6-fluoro-1-methyl-4-oxo-1,4-dihydroquinoline-3-carboxylate (**19a**)

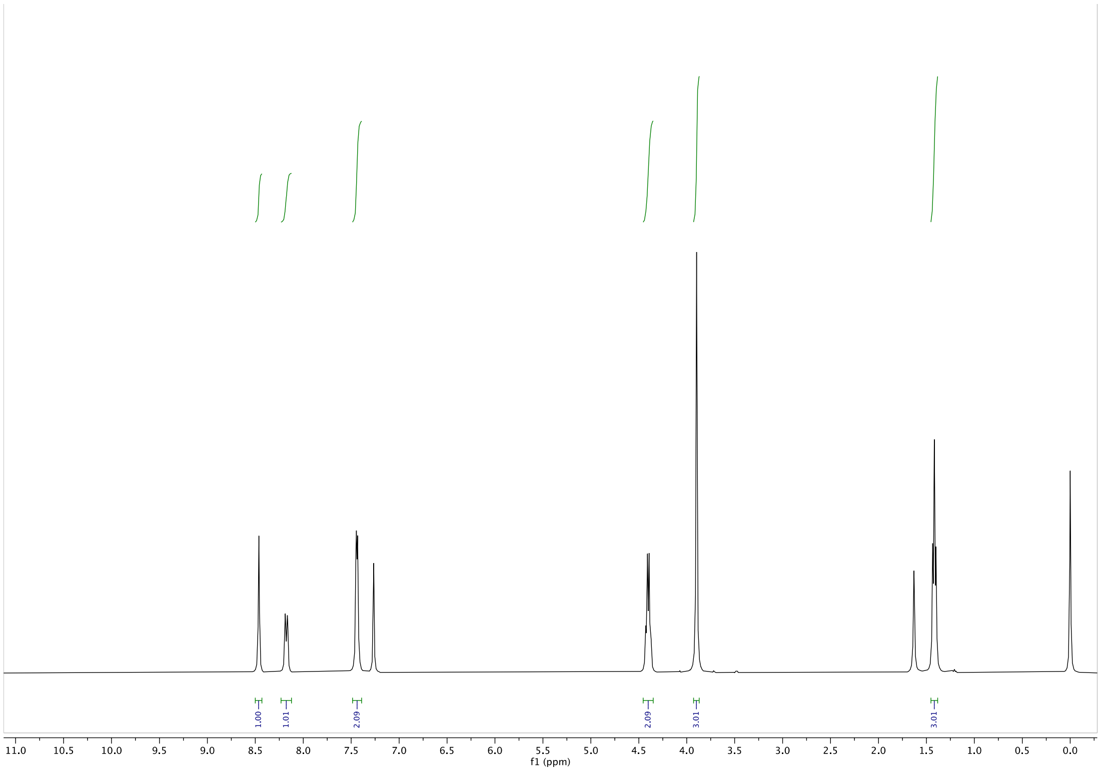

^{13}C NMR for Ethyl 6-fluoro-1-methyl-4-oxo-1,4-dihydroquinoline-3-carboxylate (**19a**)

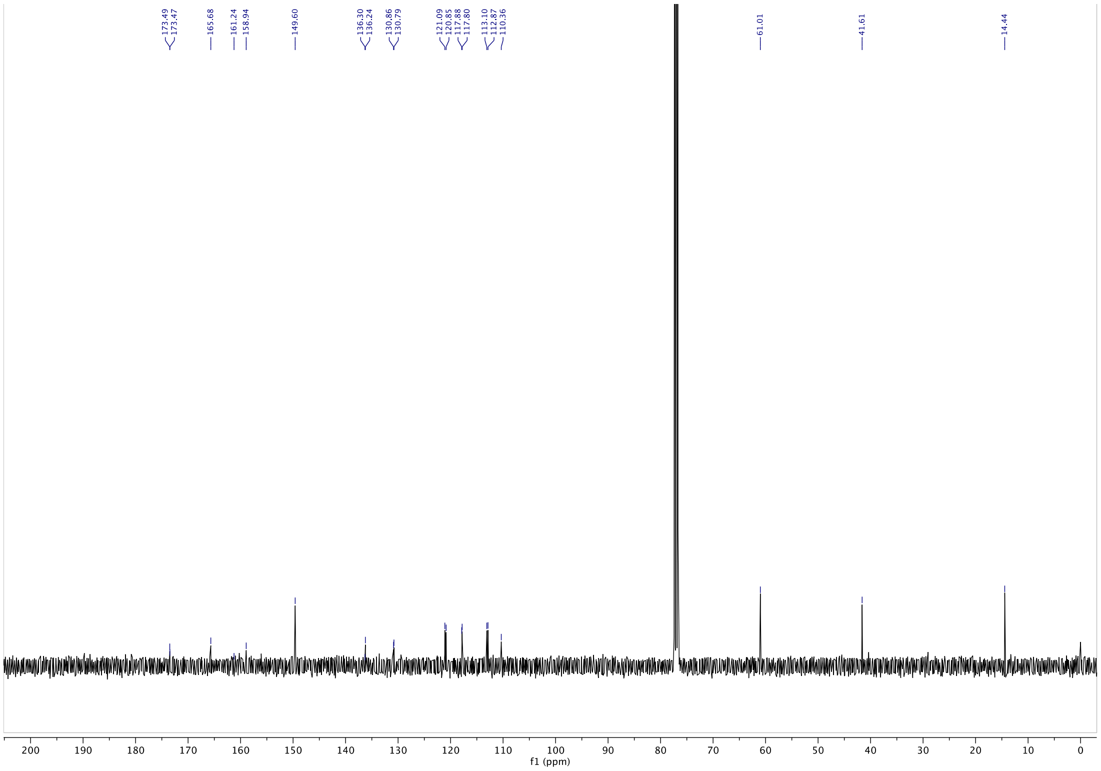

^{1}H NMR for Ethyl 6-fluoro-1-isobutyl-4-oxo-1,4-dihydroquinoline-3-carboxylate (**19c**)

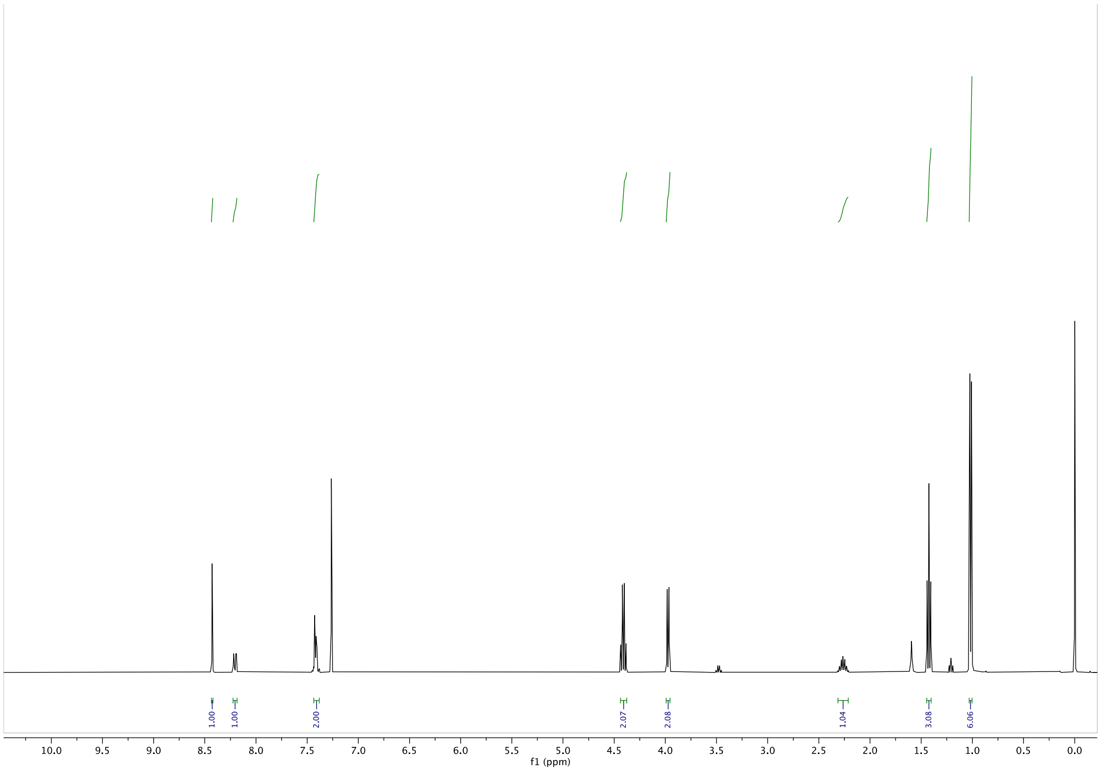

^{13}C NMR for Ethyl 6-fluoro-1-isobutyl-4-oxo-1,4-dihydroquinoline-3-carboxylate (**19c**)

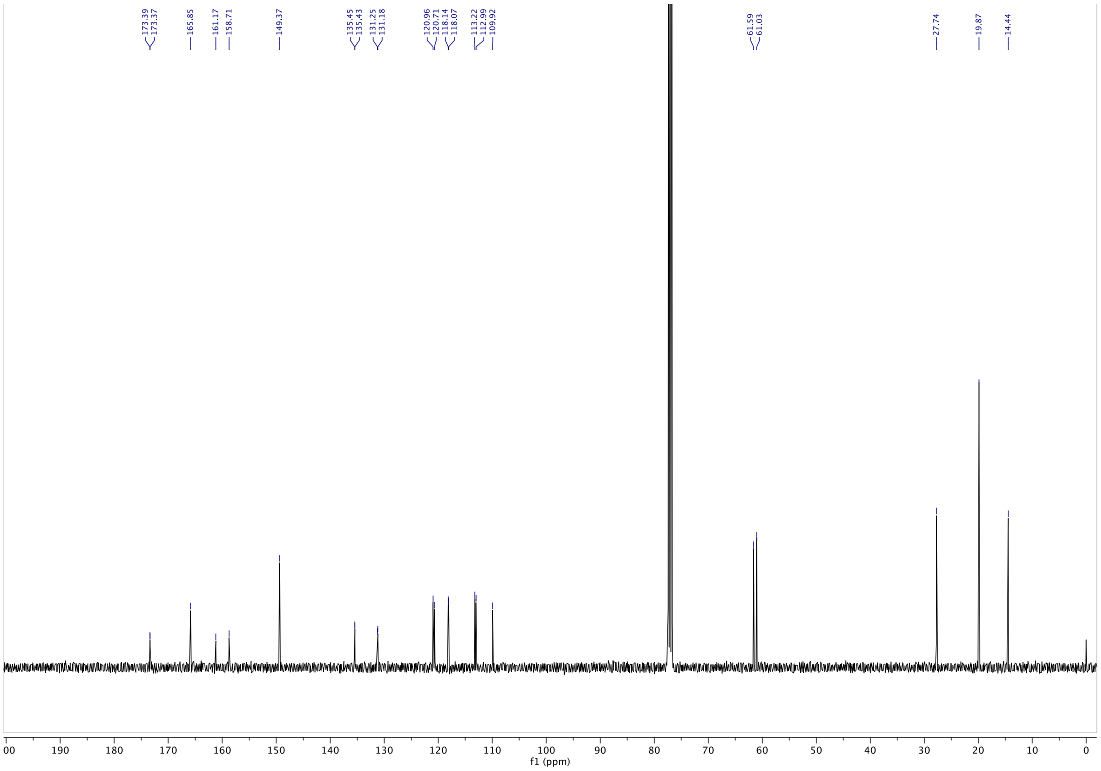

^{1}H NMR for Ethyl 1-benzyl-6-fluoro-4-oxo-1,4-dihydroquinoline-3-carboxylate (**19d**)

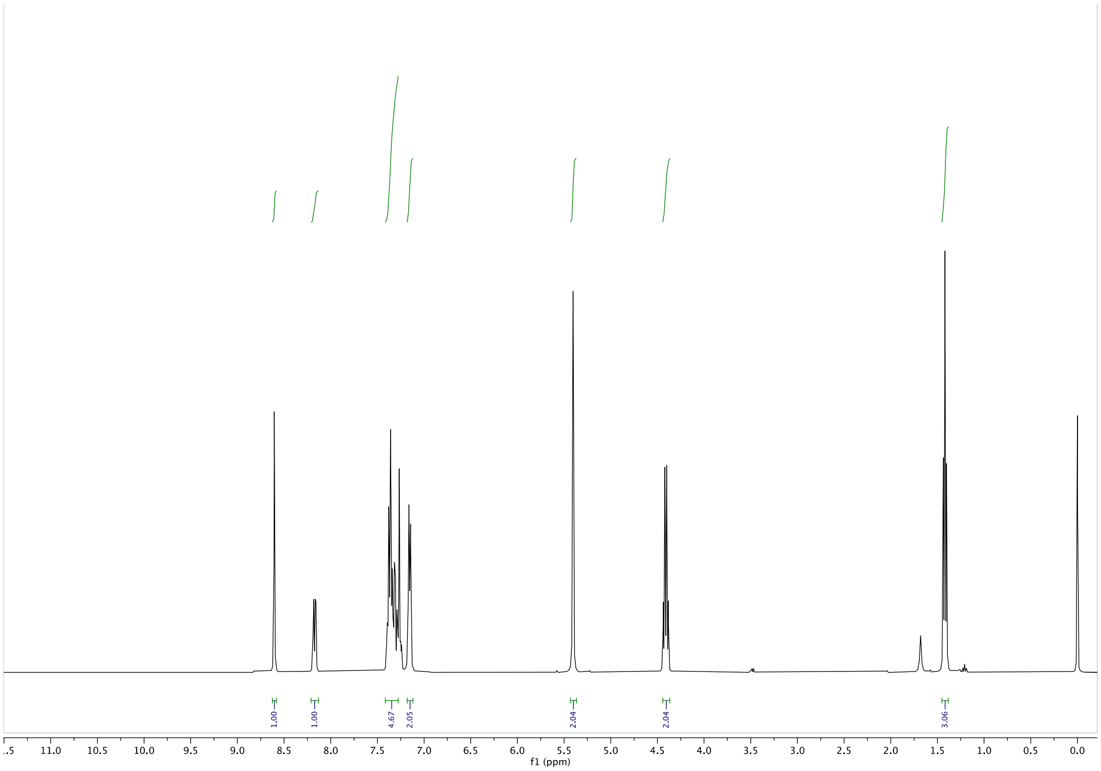

^{13}C NMR for Ethyl 1-benzyl-6-fluoro-4-oxo-1,4-dihydroquinoline-3-carboxylate (**19d**)

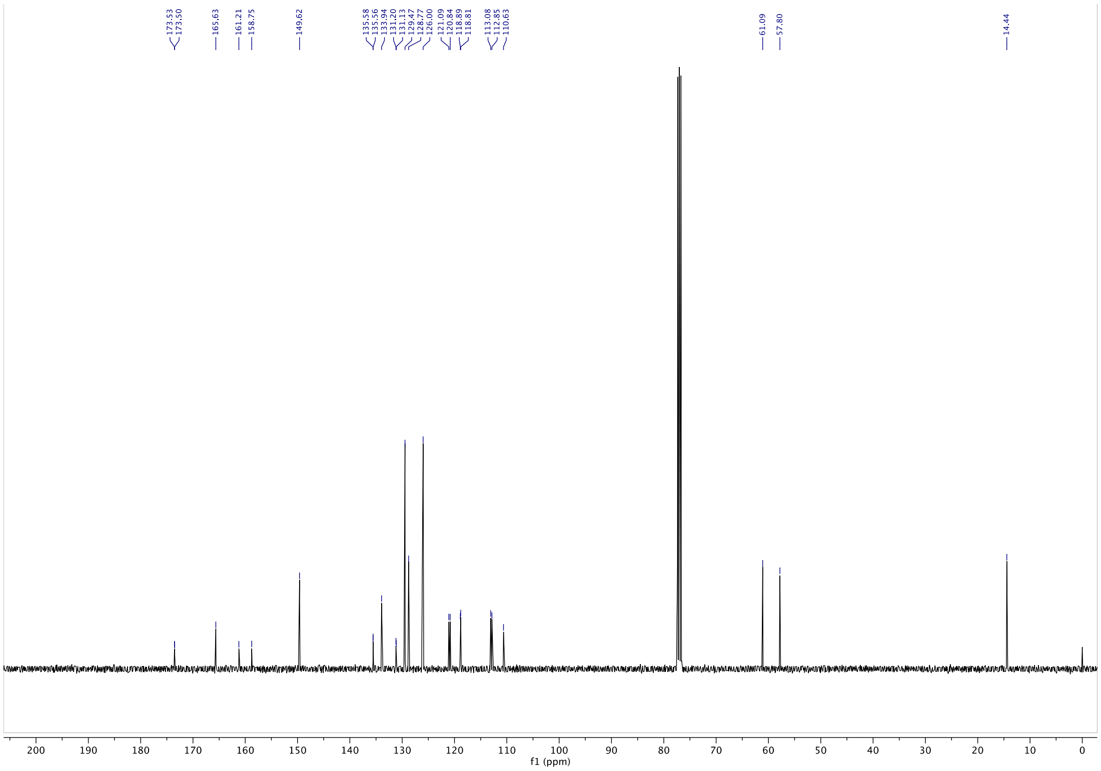

^{1}H NMR for Ethyl 6-fluoro-4-oxo-1-phenethyl-1,4-dihydroquinoline-3-carboxylate (**19e**)

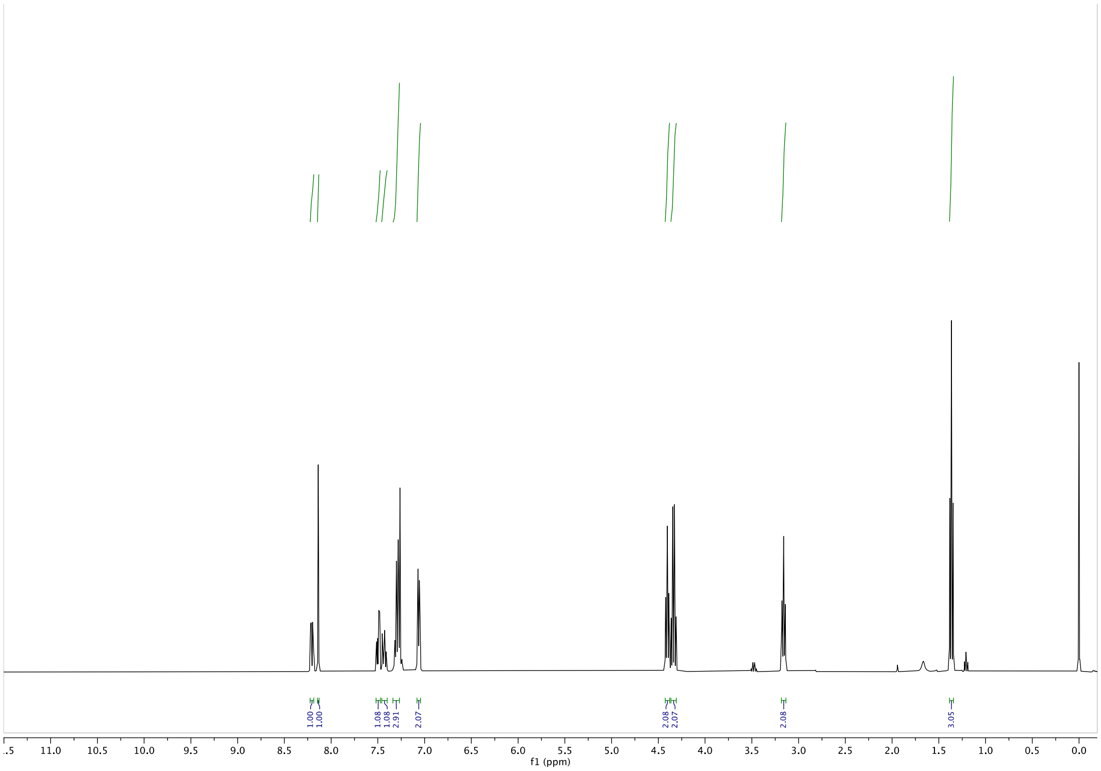

^{13}C NMR for Ethyl 6-fluoro-4-oxo-1-phenethyl-1,4-dihydroquinoline-3-carboxylate (**19e**)

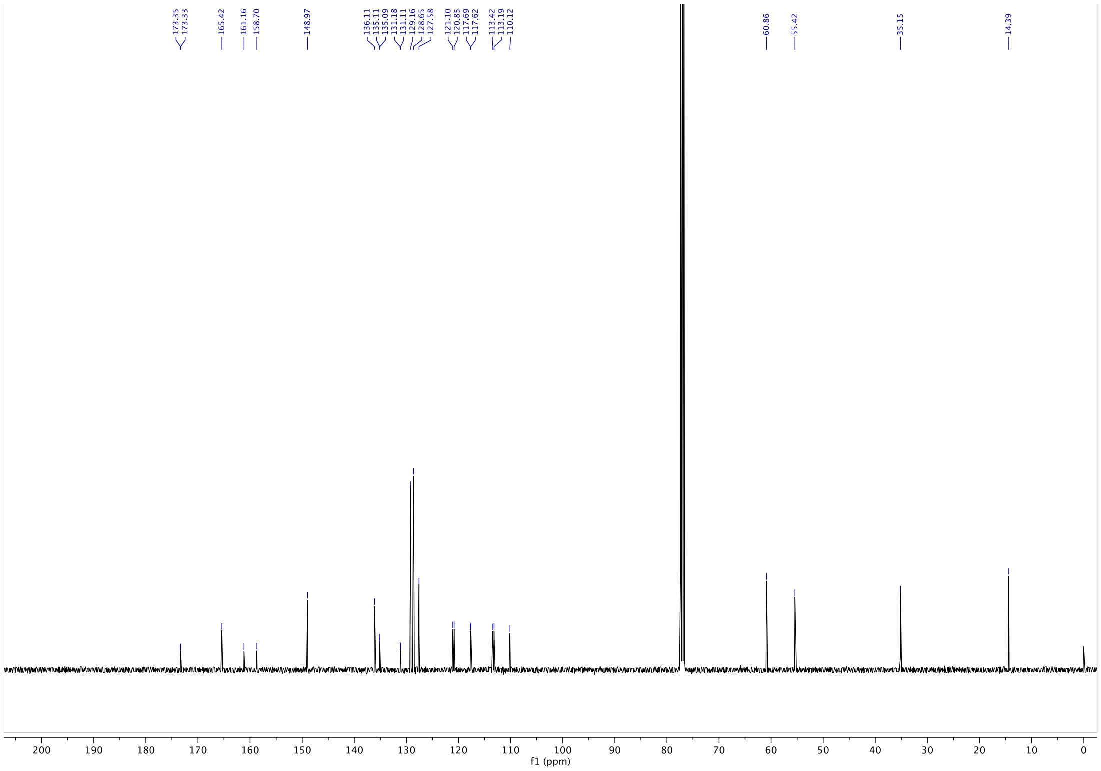

1-Benzyl-1,4-dihydroquinoline-3-carbonitrile (**15d**, EB-1-INT-6, CCDC 2035027)

Comment

The displacement ellipsoids were drawn at the 50% probability level.

Experimental

A colorless, plate-shaped crystal of dimensions 0.044 x 0.196 x 0.258 mm was selected for structural analysis. Intensity data for this compound were collected using a D8 Quest κ-geometry diffractometer with a Bruker Photon II CMOS area detector [1,2] and an Incoatec Iμs microfocus Mo Kα source (λ = 0.71073 Å). The sample was cooled to 100(2) K. Cell parameters were determined from a least-squares fit of 7723 peaks in the range 2.85 < θ < 28.73°. A total of 21066 data were measured in the range 2.848 < θ < 28.786° using ϕ and ω oscillation frames. The data were corrected for absorption by the empirical method [3] giving minimum and maximum transmission factors of 0.6236 and 0.7458. The data were merged to form a set of 3242 independent data with R(int) = 0.0490 and a coverage of 99.8%.

The orthorhombic space group $P2_12_12_1$ was determined by systematic absences and statistical tests and verified by subsequent refinement. The structure was solved by direct methods and refined by full-matrix least-squares methods on F^2 [4,5]. The positions of hydrogens bonded to carbons were initially determined by geometry and were refined using a riding model. Non-hydrogen atoms were refined with anisotropic displacement parameters. Hydrogen atom displacement parameters were set to 1.2 times the isotropic equivalent displacement parameters of the bonded atoms. A total of 172 parameters were refined against 3242 data to give wR(F^2) = 0.0766 and S = 1.007 for weights of $w = 1/[\sigma^2(F^2) + (0.0220\ P)^2 + 0.4000\ P]$, where $P = [Fo^2 + 2Fc^2] / 3$. The final R(F) was 0.0352 for the 2932 observed, [$F > 4\sigma(F)$], data. The largest shift/s.u. was 0.000 in the final refinement cycle. The final difference map had maxima and minima of 0.149 and -0.174 e/Å^3, respectively. The absolute structure was determined by refinement of the Hooft parameter [7].

Thermal Elipsoid Plot of Structure for **15d**

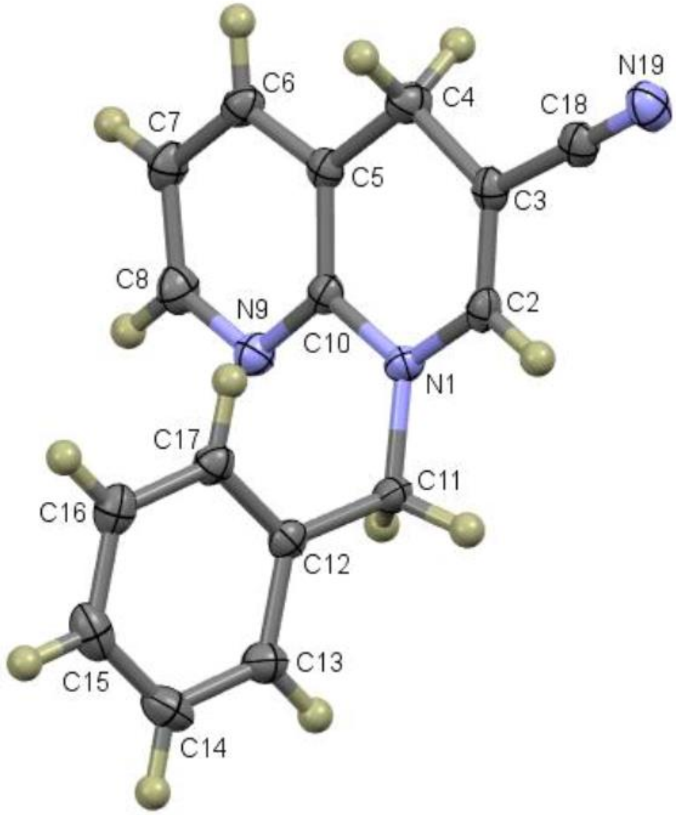

Table 1. Crystal data and structure refinement for 1-benzyl-1,4-dihydroquinoline-3-carbonitrile (**15d**, EB-1-UNK-Bn, CCDC 2035027).

Empirical formula	$C_{16} H_{13} N_3$	
Formula weight	247.29	
Crystal system	orthorhombic	
Space group	$P2_12_12_1$	
Unit cell dimensions	*a* = 6.1666(2) Å	α= 90°
	b = 14.1911(5) Å	β= 90°
	c = 14.3040(5) Å	γ= 90°
Volume	1251.76(7) $Å^3$	
Z, Z'	4, 1	
Density (calculated)	1.312 Mg/m^3	
Wavelength	0.71073 Å	
Temperature	100(2) K	
F(000)	520	
Absorption coefficient	0.080 mm^{-1}	
Absorption correction	semi-empirical from equivalents	
Max. and min. transmission	0.7458 and 0.6236	
Theta range for data collection	2.848 to 28.786°	
Reflections collected	21066	
Independent reflections	3242 [R(int) = 0.0490]	
Data / restraints / parameters	3242 / 0 / 172	
$wR(F^2$ all data)	$wR2$ = 0.0766	
$R(F$ obsd data)	$R1$ = 0.0352	
Goodness-of-fit on F^2	1.007	
Observed data [I > 2σ(I)]	2932	
Absolute structure parameter	-0.2(8)	
Largest and mean shift / s.u.	0.000 and 0.000	
Largest diff. peak and hole	0.149 and -0.174 $e/Å^3$	

Table 2. Atomic coordinates and equivalent isotropic displacement parameters for EB-1-UNK-Bn. U(eq) is defined as one third of the trace of the orthogonalized U_{ij} tensor.

	x	y	z	U(eq)
N(1)	0.4747(2)	0.47351(10)	0.40286(10)	0.0212(3)
C(2)	0.6301(3)	0.40799(12)	0.42218(12)	0.0212(3)
C(3)	0.8070(3)	0.39319(12)	0.36868(12)	0.0213(3)
C(4)	0.8535(3)	0.44924(13)	0.28136(12)	0.0262(4)
C(5)	0.6740(3)	0.51879(12)	0.26254(11)	0.0196(3)
C(6)	0.6822(3)	0.57730(12)	0.18511(12)	0.0235(4)
C(7)	0.5140(3)	0.63979(13)	0.16644(12)	0.0259(4)
C(8)	0.3415(3)	0.64166(13)	0.22777(13)	0.0273(4)
N(9)	0.3288(3)	0.58799(11)	0.30511(10)	0.0246(3)
C(10)	0.4922(3)	0.52853(12)	0.32112(11)	0.0200(3)
C(11)	0.3063(3)	0.49375(12)	0.47214(12)	0.0230(4)
C(12)	0.3518(3)	0.57988(12)	0.53217(12)	0.0211(3)
C(13)	0.1914(3)	0.60788(13)	0.59508(13)	0.0266(4)
C(14)	0.2197(3)	0.68820(15)	0.64924(14)	0.0328(4)
C(15)	0.4066(3)	0.74173(14)	0.64036(14)	0.0314(4)
C(16)	0.5677(3)	0.71390(13)	0.57895(13)	0.0277(4)
C(17)	0.5404(3)	0.63256(13)	0.52550(12)	0.0228(4)
C(18)	0.9555(3)	0.32150(13)	0.39676(12)	0.0239(4)
N(19)	1.0777(3)	0.26444(11)	0.41882(11)	0.0305(4)

Table 3. Bond lengths [Å] and angles [°] for EB-1-UNK-Bn.

N(1)-C(2)	1.363(2)	C(8)-H(8)	0.9500
N(1)-C(10)	1.410(2)	N(9)-C(10)	1.334(2)
N(1)-C(11)	1.464(2)	C(11)-C(12)	1.520(2)
C(2)-C(3)	1.349(2)	C(11)-H(11A)	0.9900
C(2)-H(2)	0.9500	C(11)-H(11B)	0.9900
C(3)-C(18)	1.427(2)	C(12)-C(17)	1.386(2)
C(3)-C(4)	1.508(2)	C(12)-C(13)	1.395(2)
C(4)-C(5)	1.508(2)	C(13)-C(14)	1.389(3)
C(4)-H(4A)	0.9900	C(13)-H(13)	0.9500
C(4)-H(4AB)	0.9900	C(14)-C(15)	1.386(3)
C(5)-C(6)	1.385(2)	C(14)-H(14)	0.9500
C(5)-C(10)	1.406(2)	C(15)-C(16)	1.384(3)
C(6)-C(7)	1.391(3)	C(15)-H(15)	0.9500
C(6)-H(6)	0.9500	C(16)-C(17)	1.395(3)
C(7)-C(8)	1.379(3)	C(16)-H(16)	0.9500
C(7)-H(7)	0.9500	C(17)-H(17)	0.9500
C(8)-N(9)	1.345(2)	C(18)-N(19)	1.150(2)
C(2)-N(1)-C(10)	119.47(14)	N(9)-C(8)-C(7)	123.86(18)
C(2)-N(1)-C(11)	119.67(14)	N(9)-C(8)-H(8)	118.1
C(10)-N(1)-C(11)	120.46(14)	C(7)-C(8)-H(8)	118.1
C(3)-C(2)-N(1)	124.01(15)	C(10)-N(9)-C(8)	117.09(16)
C(3)-C(2)-H(2)	118.0	N(9)-C(10)-C(5)	124.18(15)
N(1)-C(2)-H(2)	118.0	N(9)-C(10)-N(1)	115.78(15)
C(2)-C(3)-C(18)	118.06(16)	C(5)-C(10)-N(1)	120.04(15)
C(2)-C(3)-C(4)	122.80(16)	N(1)-C(11)-C(12)	114.17(14)
C(18)-C(3)-C(4)	119.14(16)	N(1)-C(11)-H(11A)	108.7
C(5)-C(4)-C(3)	110.69(15)	C(12)-C(11)-H(11A)	108.7
C(5)-C(4)-H(4A)	109.5	N(1)-C(11)-H(11B)	108.7
C(3)-C(4)-H(4A)	109.5	C(12)-C(11)-H(11B)	108.7
C(5)-C(4)-H(4AB)	109.5	H(11A)-C(11)-H(11B)	107.6
C(3)-C(4)-H(4AB)	109.5	C(17)-C(12)-C(13)	119.07(17)
H(4A)-C(4)-H(4AB)	108.1	C(17)-C(12)-C(11)	123.35(15)
C(6)-C(5)-C(10)	116.53(16)	C(13)-C(12)-C(11)	117.55(16)
C(6)-C(5)-C(4)	120.55(16)	C(14)-C(13)-C(12)	120.27(18)
C(10)-C(5)-C(4)	122.92(15)	C(14)-C(13)-H(13)	119.9
C(5)-C(6)-C(7)	120.56(17)	C(12)-C(13)-H(13)	119.9
C(5)-C(6)-H(6)	119.7	C(15)-C(14)-C(13)	120.21(19)
C(7)-C(6)-H(6)	119.7	C(15)-C(14)-H(14)	119.9
C(8)-C(7)-C(6)	117.74(16)	C(13)-C(14)-H(14)	119.9
C(8)-C(7)-H(7)	121.1	C(16)-C(15)-C(14)	119.92(18)
C(6)-C(7)-H(7)	121.1	C(16)-C(15)-H(15)	120.0

C(14)-C(15)-H(15)	120.0	C(12)-C(17)-C(16)	120.66(17)
C(15)-C(16)-C(17)	119.84(19)	C(12)-C(17)-H(17)	119.7
C(15)-C(16)-H(16)	120.1	C(16)-C(17)-H(17)	119.7
C(17)-C(16)-H(16)	120.1	N(19)-C(18)-C(3)	179.0(2)

Table 4. Anisotropic displacement parameters ($Å^2 x 10^3$) for EB-1-UNK-Bn. The anisotropic displacement factor exponent takes the form: $-2 \pi^2[h^2 a^{*2} U_{11} + ... + 2 h k a^* b^* U_{12}]$

	U_{11}	U_{22}	U_{33}	U_{23}	U_{13}	U_{12}
N(1)	26(1)	20(1)	17(1)	1(1)	4(1)	0(1)
C(2)	30(1)	16(1)	17(1)	0(1)	-3(1)	-4(1)
C(3)	28(1)	18(1)	18(1)	-1(1)	-4(1)	-1(1)
C(4)	28(1)	30(1)	21(1)	4(1)	2(1)	5(1)
C(5)	23(1)	19(1)	17(1)	-2(1)	-2(1)	-3(1)
C(6)	28(1)	25(1)	17(1)	0(1)	1(1)	-4(1)
C(7)	34(1)	24(1)	20(1)	6(1)	-4(1)	-3(1)
C(8)	30(1)	25(1)	26(1)	5(1)	-4(1)	4(1)
N(9)	27(1)	24(1)	23(1)	2(1)	0(1)	1(1)
C(10)	25(1)	18(1)	17(1)	0(1)	0(1)	-3(1)
C(11)	25(1)	24(1)	21(1)	1(1)	5(1)	-4(1)
C(12)	25(1)	21(1)	17(1)	4(1)	0(1)	2(1)
C(13)	25(1)	30(1)	25(1)	1(1)	3(1)	2(1)
C(14)	35(1)	36(1)	28(1)	-5(1)	3(1)	11(1)
C(15)	42(1)	24(1)	28(1)	-4(1)	-10(1)	7(1)
C(16)	33(1)	24(1)	26(1)	2(1)	-5(1)	-5(1)
C(17)	26(1)	25(1)	18(1)	2(1)	1(1)	-2(1)
C(18)	31(1)	21(1)	19(1)	-3(1)	-2(1)	-2(1)
N(19)	39(1)	27(1)	26(1)	2(1)	0(1)	7(1)

Table 5. Hydrogen coordinates and isotropic displacement parameters for EB-1-UNK-Bn.

	x	y	z	U(eq)
H(2)	0.612701	0.370455	0.476662	0.025
H(4A)	0.992419	0.483411	0.288797	0.031
H(4AB)	0.867954	0.405888	0.227490	0.031
H(6)	0.803791	0.574721	0.144410	0.028
H(7)	0.517750	0.679827	0.113240	0.031
H(8)	0.224822	0.683300	0.214639	0.033
H(11A)	0.289970	0.438268	0.513499	0.028
H(11B)	0.166798	0.503150	0.439214	0.028
H(13)	0.062213	0.571871	0.600909	0.032
H(14)	0.110704	0.706507	0.692498	0.039
H(15)	0.424092	0.797444	0.676395	0.038
H(16)	0.696622	0.750117	0.573201	0.033
H(17)	0.652263	0.613078	0.484104	0.027

Table 6. Torsion angles [°] for EB-1-UNK-Bn.

C(10)-N(1)-C(2)-C(3)	1.8(2)
C(11)-N(1)-C(2)-C(3)	-170.98(16)
N(1)-C(2)-C(3)-C(18)	-179.37(16)
N(1)-C(2)-C(3)-C(4)	0.8(3)
C(2)-C(3)-C(4)-C(5)	-2.0(2)
C(18)-C(3)-C(4)-C(5)	178.16(15)
C(3)-C(4)-C(5)-C(6)	-179.43(16)
C(3)-C(4)-C(5)-C(10)	0.8(2)
C(10)-C(5)-C(6)-C(7)	-1.9(2)
C(4)-C(5)-C(6)-C(7)	178.36(16)
C(5)-C(6)-C(7)-C(8)	0.5(3)
C(6)-C(7)-C(8)-N(9)	1.3(3)
C(7)-C(8)-N(9)-C(10)	-1.4(3)
C(8)-N(9)-C(10)-C(5)	-0.1(3)
C(8)-N(9)-C(10)-N(1)	179.76(15)
C(6)-C(5)-C(10)-N(9)	1.7(2)
C(4)-C(5)-C(10)-N(9)	-178.50(16)
C(6)-C(5)-C(10)-N(1)	-178.15(15)
C(4)-C(5)-C(10)-N(1)	1.6(2)
C(2)-N(1)-C(10)-N(9)	177.11(15)
C(11)-N(1)-C(10)-N(9)	-10.1(2)
C(2)-N(1)-C(10)-C(5)	-3.0(2)
C(11)-N(1)-C(10)-C(5)	169.74(15)
C(2)-N(1)-C(11)-C(12)	97.36(18)
C(10)-N(1)-C(11)-C(12)	-75.4(2)
N(1)-C(11)-C(12)-C(17)	-2.9(2)
N(1)-C(11)-C(12)-C(13)	175.32(16)
C(17)-C(12)-C(13)-C(14)	0.9(3)
C(11)-C(12)-C(13)-C(14)	-177.45(16)
C(12)-C(13)-C(14)-C(15)	0.8(3)
C(13)-C(14)-C(15)-C(16)	-1.5(3)
C(14)-C(15)-C(16)-C(17)	0.6(3)
C(13)-C(12)-C(17)-C(16)	-1.7(3)
C(11)-C(12)-C(17)-C(16)	176.48(17)
C(15)-C(16)-C(17)-C(12)	1.0(3)

Table 7. Hydrogen bonds for EB-1-UNK-Bn [Å and °].

D-H...A	d(D-H)	d(H...A)	d(D...A)	<(DHA)
C(2)-H(2)...N(19)#1	0.95	2.44	3.356(2)	162.4

Symmetry transformations used to generate equivalent atoms:
#1 x-1/2, -y+1/2, -z+1

(*Z*)-2-((Benzylamino)methyl)-3-(2-fluoro-5-nitrophenyl)acrylonitrile (**16**, EB-1-INT-6, CCDC 2035028)

Comment

The displacement ellipsoids were drawn at the 50% probability level.

Experimental

A yellow, plate-shaped crystal of dimensions 0.028 x 0.152 x 0.248 mm was selected for structural analysis. Intensity data for this compound were collected using a D8 Quest κ-geometry diffractometer with a Bruker Photon II CMOS area detector [1,2] and an Incoatec Iμs microfocus Mo Kα source (λ = 0.71073 Å). The sample was cooled to 100(2) K. Cell parameters were determined from a least-squares fit of 7081 peaks in the range 2.76 < θ < 25.64°. A total of 21764 data were measured in the range 2.758 < θ < 25.821° using ϕ and ω oscillation frames. The data were corrected for absorption by the empirical method [3] giving minimum and maximum transmission factors of 0.5330 and 0.6463. The data were merged to form a set of 2763 independent data with R(int) = 0.0762 and a coverage of 99.9%.

The monoclinic space group *Cc* was determined by systematic absences and statistical tests and verified by subsequent refinement. The structure was solved by dual-space methods and refined by full-matrix least-squares methods on F^2 [4,5]. The positions of hydrogens bonded to carbons were initially determined by geometry and were refined using a riding model. The hydrogens bonded to N3 was located on a difference map, and its position was refined independently. Non-hydrogen atoms were refined with anisotropic displacement parameters. Hydrogen atom displacement parameters were set to 1.2 times the isotropic equivalent displacement parameters of the bonded atoms. A total of 212 parameters were refined against 2 space group restraints and 2763 data to give $wR(F^2) = 0.1163$ and S = 1.046 for weights of $w = 1/[\sigma^2(F^2) + (0.0600\ P)^2 + 0.8600\ P]$, where $P = [Fo^2 + 2Fc^2] / 3$. The final R(*F*) was 0.0462 for the 2261 observed, $[F > 4\sigma(F)]$, data. The largest shift/s.u. was 0.000 in the final refinement cycle. The final difference map had maxima and minima of 0.241 and -0.229 e/Å^3, respectively. The absolute structure could not be determined by refinement of the Flack parameter [6].

Thermal Elipsoid Plot of Structure **16**

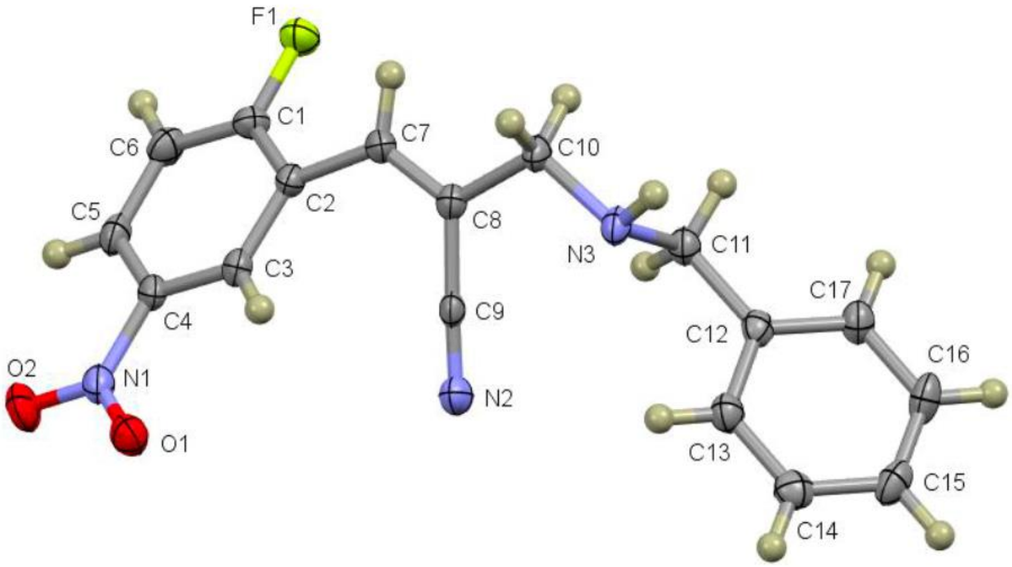

Table 1. Crystal data and structure refinement for (*Z*)-2-((benzylamino)methyl)-3-(2-fluoro-5-nitrophenyl)acrylonitrile (**16**, EB-1-INT-6, CCDC 2035028).

Empirical formula	$C_{17} H_{14} F N_3 O_2$	
Formula weight	311.31	
Crystal system	monoclinic	
Space group	Cc	
Unit cell dimensions	*a* = 6.5638(10) Å	α= 90°
	b = 29.540(5) Å	β= 102.722(5)°
	c = 7.6292(13) Å	γ= 90°
Volume	1442.9(4) $Å^3$	
Z, Z'	4, 1	
Density (calculated)	1.433 Mg/m^3	
Wavelength	0.71073 Å	
Temperature	100(2) K	
F(000)	648	
Absorption coefficient	0.105 mm^{-1}	
Absorption correction	semi-empirical from equivalents	
Max. and min. transmission	0.6463 and 0.5330	
Theta range for data collection	2.758 to 25.821°	
Reflections collected	21764	
Independent reflections	2763 [R(int) = 0.0762]	
Data / restraints / parameters	2763 / 2 / 212	
$wR(F^2$ all data)	$wR2$ = 0.1163	
$R(F$ obsd data)	$R1$ = 0.0462	
Goodness-of-fit on F^2	1.046	
Observed data [I > 2σ(I)]	2261	
Absolute structure parameter	-0.1(7)	
Extinction coefficient	0.013(2)	
Largest and mean shift / s.u.	0.000 and 0.000	
Largest diff. peak and hole	0.241 and -0.229 e/$Å^3$	

$wR2 = \{ \Sigma[w(Fo2 - Fc2)2] / \Sigma[w(Fo\ 2)2] \}^{1/2}$

$R1 = \Sigma||Fo| - |Fc|| / \Sigma|Fo|$

Table 2. Atomic coordinates and equivalent isotropic displacement parameters for EB-1-INT-6. U(eq) is defined as one third of the trace of the orthogonalized U_{ij} tensor.

	x	y	z	U(eq)
F(1)	-0.0126(4)	0.60772(8)	0.3358(4)	0.0319(7)
O(1)	0.9058(5)	0.54806(11)	0.6265(5)	0.0335(8)
O(2)	0.7879(5)	0.49044(10)	0.4594(5)	0.0408(9)
N(1)	0.7638(6)	0.52817(12)	0.5233(5)	0.0283(9)
N(2)	0.7929(6)	0.69387(12)	0.5406(6)	0.0303(9)
N(3)	0.5005(6)	0.77335(11)	0.6854(5)	0.0242(8)
C(1)	0.1789(7)	0.58866(14)	0.3822(6)	0.0253(10)
C(2)	0.3378(6)	0.61292(13)	0.4932(6)	0.0227(10)
C(3)	0.5324(7)	0.59176(14)	0.5399(6)	0.0244(10)
C(4)	0.5585(7)	0.54932(13)	0.4720(6)	0.0228(9)
C(5)	0.3987(7)	0.52601(14)	0.3596(6)	0.0280(11)
C(6)	0.2034(7)	0.54618(15)	0.3144(7)	0.0295(11)
C(7)	0.2920(7)	0.65763(13)	0.5595(6)	0.0232(9)
C(8)	0.4216(6)	0.69304(14)	0.6024(6)	0.0225(9)
C(9)	0.6294(7)	0.69282(12)	0.5714(6)	0.0221(9)
C(10)	0.3513(7)	0.73599(13)	0.6815(7)	0.0257(10)
C(11)	0.4777(7)	0.79601(13)	0.5127(6)	0.0247(10)
C(12)	0.6472(7)	0.83085(14)	0.5184(6)	0.0227(9)
C(13)	0.8526(7)	0.82210(14)	0.6069(6)	0.0269(10)
C(14)	1.0095(7)	0.85372(15)	0.6089(6)	0.0291(10)
C(15)	0.9624(8)	0.89512(14)	0.5209(7)	0.0323(11)
C(16)	0.7594(8)	0.90415(15)	0.4344(7)	0.0334(11)
C(17)	0.6032(7)	0.87252(14)	0.4333(7)	0.0278(10)

Table 3. Bond lengths [Å] and angles [°] for EB-1-INT-6.

F(1)-C(1)	1.351(5)	C(7)-H(7)	0.9500
O(1)-N(1)	1.229(5)	C(8)-C(9)	1.435(6)
O(2)-N(1)	1.241(5)	C(8)-C(10)	1.519(6)
N(1)-C(4)	1.458(5)	C(10)-H(10A)	0.9900
N(2)-C(9)	1.149(6)	C(10)-H(10B)	0.9900
N(3)-C(11)	1.456(6)	C(11)-C(12)	1.509(6)
N(3)-C(10)	1.471(5)	C(11)-H(11A)	0.9900
N(3)-H(3N)	0.96(5)	C(11)-H(11B)	0.9900
C(1)-C(6)	1.380(6)	C(12)-C(17)	1.392(6)
C(1)-C(2)	1.389(6)	C(12)-C(13)	1.392(6)
C(2)-C(3)	1.396(6)	C(13)-C(14)	1.388(7)
C(2)-C(7)	1.469(5)	C(13)-H(13)	0.9500
C(3)-C(4)	1.382(6)	C(14)-C(15)	1.396(7)
C(3)-H(3)	0.9500	C(14)-H(14)	0.9500
C(4)-C(5)	1.383(6)	C(15)-C(16)	1.377(7)
C(5)-C(6)	1.387(7)	C(15)-H(15)	0.9500
C(5)-H(5)	0.9500	C(16)-C(17)	1.386(6)
C(6)-H(6)	0.9500	C(16)-H(16)	0.9500
C(7)-C(8)	1.342(6)	C(17)-H(17)	0.9500
O(1)-N(1)-O(2)	122.5(4)	C(1)-C(6)-H(6)	120.8
O(1)-N(1)-C(4)	119.5(3)	C(5)-C(6)-H(6)	120.8
O(2)-N(1)-C(4)	118.0(4)	C(8)-C(7)-C(2)	128.2(4)
C(11)-N(3)-C(10)	113.1(4)	C(8)-C(7)-H(7)	115.9
C(11)-N(3)-H(3N)	109(3)	C(2)-C(7)-H(7)	115.9
C(10)-N(3)-H(3N)	105(3)	C(7)-C(8)-C(9)	122.0(4)
F(1)-C(1)-C(6)	117.3(4)	C(7)-C(8)-C(10)	121.1(4)
F(1)-C(1)-C(2)	118.3(4)	C(9)-C(8)-C(10)	116.9(3)
C(6)-C(1)-C(2)	124.3(4)	N(2)-C(9)-C(8)	177.1(5)
C(1)-C(2)-C(3)	116.5(4)	N(3)-C(10)-C(8)	112.0(3)
C(1)-C(2)-C(7)	119.4(4)	N(3)-C(10)-H(10A)	109.2
C(3)-C(2)-C(7)	124.0(4)	C(8)-C(10)-H(10A)	109.2
C(4)-C(3)-C(2)	119.5(4)	N(3)-C(10)-H(10B)	109.2
C(4)-C(3)-H(3)	120.2	C(8)-C(10)-H(10B)	109.2
C(2)-C(3)-H(3)	120.2	H(10A)-C(10)-H(10B)	107.9
C(3)-C(4)-C(5)	123.0(4)	N(3)-C(11)-C(12)	111.1(3)
C(3)-C(4)-N(1)	118.1(4)	N(3)-C(11)-H(11A)	109.4
C(5)-C(4)-N(1)	118.9(4)	C(12)-C(11)-H(11A)	109.4
C(4)-C(5)-C(6)	118.3(4)	N(3)-C(11)-H(11B)	109.4
C(4)-C(5)-H(5)	120.9	C(12)-C(11)-H(11B)	109.4
C(6)-C(5)-H(5)	120.9	H(11A)-C(11)-H(11B)	108.0
C(1)-C(6)-C(5)	118.3(4)	C(17)-C(12)-C(13)	118.0(4)

C(17)-C(12)-C(11)	120.9(4)	C(16)-C(15)-H(15)	120.4
C(13)-C(12)-C(11)	121.1(4)	C(14)-C(15)-H(15)	120.4
C(14)-C(13)-C(12)	121.2(4)	C(15)-C(16)-C(17)	120.6(4)
C(14)-C(13)-H(13)	119.4	C(15)-C(16)-H(16)	119.7
C(12)-C(13)-H(13)	119.4	C(17)-C(16)-H(16)	119.7
C(13)-C(14)-C(15)	119.9(4)	C(16)-C(17)-C(12)	121.1(4)
C(13)-C(14)-H(14)	120.0	C(16)-C(17)-H(17)	119.5
C(15)-C(14)-H(14)	120.0	C(12)-C(17)-H(17)	119.5
C(16)-C(15)-C(14)	119.3(4)		

Table 4. Anisotropic displacement parameters ($Å^2 x 10^3$) for EB-1-INT-6. The anisotropic displacement factor exponent takes the form: $-2 \pi^2 [h^2 a^{*2} U_{11} + ... + 2 h k a^* b^* U_{12}]$

	U_{11}	U_{22}	U_{33}	U_{23}	U_{13}	U_{12}
F(1)	21(1)	29(1)	43(2)	0(1)	1(1)	1(1)
O(1)	29(2)	27(2)	41(2)	1(2)	2(2)	3(1)
O(2)	44(2)	20(2)	59(3)	-6(2)	11(2)	7(1)
N(1)	33(2)	19(2)	36(2)	2(2)	13(2)	1(2)
N(2)	27(2)	20(2)	44(3)	-2(2)	9(2)	0(2)
N(3)	31(2)	16(2)	27(2)	0(2)	9(2)	-1(2)
C(1)	18(2)	26(2)	31(3)	2(2)	5(2)	-1(2)
C(2)	23(2)	16(2)	30(3)	0(2)	10(2)	-2(2)
C(3)	27(2)	17(2)	30(3)	3(2)	7(2)	-1(2)
C(4)	25(2)	16(2)	28(3)	5(2)	8(2)	1(2)
C(5)	35(3)	17(2)	33(3)	1(2)	9(2)	-5(2)
C(6)	27(2)	25(2)	35(3)	-2(2)	3(2)	-7(2)
C(7)	21(2)	18(2)	31(3)	3(2)	7(2)	1(2)
C(8)	23(2)	17(2)	27(2)	2(2)	5(2)	0(2)
C(9)	26(2)	12(2)	28(2)	2(2)	4(2)	0(2)
C(10)	30(2)	14(2)	35(3)	-1(2)	13(2)	-2(2)
C(11)	27(2)	19(2)	27(3)	1(2)	4(2)	0(2)
C(12)	28(2)	18(2)	24(3)	-3(2)	9(2)	0(2)
C(13)	28(2)	20(2)	32(3)	-1(2)	6(2)	1(2)
C(14)	30(2)	29(2)	29(3)	-4(2)	9(2)	1(2)
C(15)	41(3)	22(2)	35(3)	-4(2)	12(2)	-10(2)
C(16)	48(3)	17(2)	34(3)	1(2)	6(2)	-8(2)
C(17)	33(3)	20(2)	28(3)	2(2)	2(2)	0(2)

Table 5. Hydrogen coordinates and isotropic displacement parameters for EB-1-INT-6.

	x	y	z	U(eq)
H(3N)	0.469(7)	0.7944(16)	0.771(7)	0.029
H(3)	0.646092	0.606443	0.617769	0.029
H(5)	0.422162	0.496935	0.314607	0.034
H(6)	0.089185	0.531124	0.238681	0.035
H(7)	0.153256	0.662035	0.573902	0.028
H(10A)	0.213181	0.745193	0.609248	0.031
H(10B)	0.335582	0.729775	0.805392	0.031
H(11A)	0.339540	0.810970	0.480911	0.030
H(11B)	0.484475	0.773321	0.418620	0.030
H(13)	0.885882	0.793997	0.666876	0.032
H(14)	1.148708	0.847212	0.670148	0.035
H(15)	1.069233	0.916799	0.520820	0.039
H(16)	0.726100	0.932310	0.374789	0.040
H(17)	0.463777	0.879395	0.373575	0.033

Table 6. Torsion angles [°] for EB-1-INT-6.

F(1)-C(1)-C(2)-C(3)	179.1(4)
C(6)-C(1)-C(2)-C(3)	-1.3(7)
F(1)-C(1)-C(2)-C(7)	1.4(6)
C(6)-C(1)-C(2)-C(7)	-179.0(4)
C(1)-C(2)-C(3)-C(4)	1.5(6)
C(7)-C(2)-C(3)-C(4)	179.1(4)
C(2)-C(3)-C(4)-C(5)	-0.7(7)
C(2)-C(3)-C(4)-N(1)	180.0(4)
O(1)-N(1)-C(4)-C(3)	1.1(6)
O(2)-N(1)-C(4)-C(3)	-178.6(4)
O(1)-N(1)-C(4)-C(5)	-178.3(4)
O(2)-N(1)-C(4)-C(5)	2.0(6)
C(3)-C(4)-C(5)-C(6)	-0.4(6)
N(1)-C(4)-C(5)-C(6)	178.9(4)
F(1)-C(1)-C(6)-C(5)	179.8(4)
C(2)-C(1)-C(6)-C(5)	0.2(7)
C(4)-C(5)-C(6)-C(1)	0.7(6)
C(1)-C(2)-C(7)-C(8)	-150.8(5)
C(3)-C(2)-C(7)-C(8)	31.7(7)
C(2)-C(7)-C(8)-C(9)	6.1(8)
C(2)-C(7)-C(8)-C(10)	-175.8(4)
C(11)-N(3)-C(10)-C(8)	80.4(5)
C(7)-C(8)-C(10)-N(3)	-168.8(4)
C(9)-C(8)-C(10)-N(3)	9.5(6)
C(10)-N(3)-C(11)-C(12)	-174.3(3)
N(3)-C(11)-C(12)-C(17)	-140.0(4)
N(3)-C(11)-C(12)-C(13)	40.8(5)
C(17)-C(12)-C(13)-C(14)	-0.6(7)
C(11)-C(12)-C(13)-C(14)	178.6(4)
C(12)-C(13)-C(14)-C(15)	-0.1(7)
C(13)-C(14)-C(15)-C(16)	0.6(7)
C(14)-C(15)-C(16)-C(17)	-0.4(7)
C(15)-C(16)-C(17)-C(12)	-0.4(7)
C(13)-C(12)-C(17)-C(16)	0.9(7)
C(11)-C(12)-C(17)-C(16)	-178.3(4)

Table 7. Hydrogen bonds for EB-1-INT-6 [Å and °].

D-H...A	d(D-H)	d(H...A)	d(D...A)	<(DHA)
N(3)-H(3N)...N(2)#1	0.96(5)	2.59(5)	3.430(6)	146(4)
C(6)-H(6)...O(2)#2	0.95	2.65	3.562(6)	162.2

Symmetry transformations used to generate equivalent atoms:
#1 x-1/2, -y+3/2, z+1/2 #2 x-1, -y+1, z-1/2

Ethyl 6-fluoro-4-oxo-1-phenethyl-1,4-dihydroquinoline-3-carboxylate (**19e** KEVJAG-1-64, CCDC 2057847)

Comment

The displacement ellipsoids were drawn at the 50% probability level.

Experimental

A colorless, block-shaped crystal of dimensions 0.106 x 0.272 x 0.280 mm was selected for structural analysis. Intensity data for this compound were collected using a D8 Quest κ-geometry diffractometer with a Bruker Photon II CMOS area detector [1,2] and an Incoatec Iμs microfocus Mo Kα source (λ = 0.71073 Å). The sample was cooled to 110(2) K. Cell parameters were determined from a least-squares fit of 9898 peaks in the range $2.91 < \theta < 32.61°$. A total of 39073 data were measured in the range $2.468 < \theta < 32.644°$ using ϕ and ω oscillation frames. The data were corrected for absorption by the empirical method [3] giving minimum and maximum transmission factors of 0.7806 and 0.8623. The data were merged to form a set of 6040 independent data with R(int) = 0.0288 and a coverage of 99.9 %.
The orthorhombic space group $P2_12_12_1$ was determined by systematic absences and statistical tests and verified by subsequent refinement. The structure was solved by direct methods and refined by full-matrix least-squares methods on *F*2 [4,5]. The positions of hydrogens were initially determined by geometry and were refined using a riding model. Non-hydrogen atoms were refined with anisotropic displacement parameters. Hydrogen atom displacement parameters were set to 1.2 (1.5 for methyl) times the isotropic equivalent displacement parameters of the bonded atoms. A total of 227 parameters were refined against 6040 data to give $wR(F^2) = 0.0896$ and S = 1.005 for weights of $w = 1/[\sigma^2 (F^2) + (0.0580\ P)^2 + 0.2000\ P]$, where $P = [Fo^2 + 2Fc^2] / 3$. The final R(*F*) was 0.0322 for the 5695 observed, $[F > 4\ \sigma (F)]$, data. The largest shift/s.u. was 0.000 in the final refinement cycle. The final difference map had maxima and minima of 0.357 and -0.192 e/Å3, respectively. The absolute structure was determined by refinement of the Hooft parameter [8], Hooft y = 0.21(12), P2(true) = 1.000, P3(true) = 0.816, P3(rac-twin) = 0.184, P3(false) = 0.2E-08.

Thermal Elipsoid Plot of **19e**

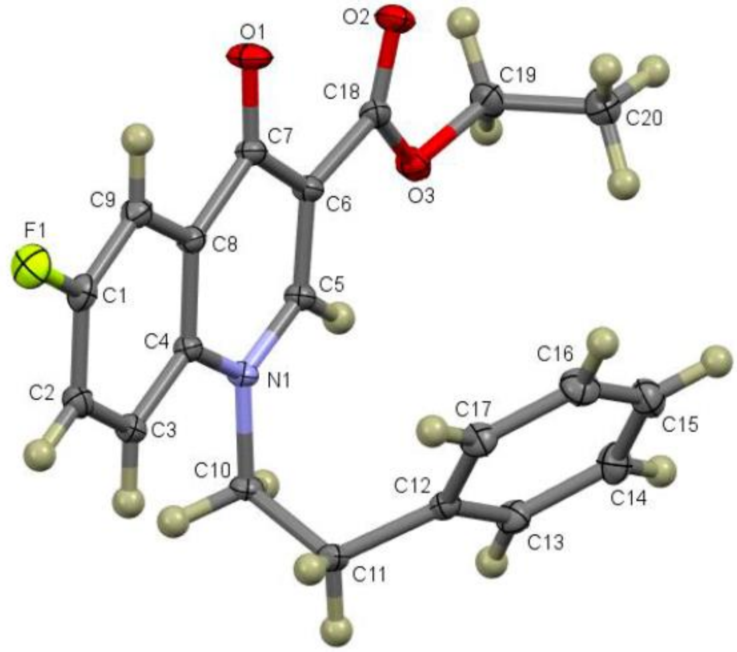

Table 1. Crystal data and structure refinement for ethyl 6-fluoro-4-oxo-1-phenethyl-1,4-dihydroquinoline-3-carboxylate (**19e**, KEVJAG-1-64, CCDC 2057847).

Empirical formula	$C_{20}H_{18}FNO_3$	
Formula weight	339.35	
Crystal system	orthorhombic	
Space group	$P2_12_12_1$	
Unit cell dimensions	*a* = 5.5713(2) Å	α= 90°
	b = 14.9835(4) Å	β= 90°
	c = 19.7736(5) Å	γ= 90°
Volume	650.65(8) $Å^3$	
Z, Z'	4, 1	
Density (calculated)	1.366 Mg/m^3	
Wavelength	0.71073 Å	
Temperature	110(2) K	
F(000)	712	
Absorption coefficient	0.099 mm^{-1}	
Absorption correction	semi-empirical from equivalents	
Max. and min. transmission	0.8623 and 0.7806	
Theta range for data collection	2.468 to 32.644°	
Reflections collected	39073	
Independent reflections	6040 [R(int) = 0.0288]	
Data / restraints / parameters	6040 / 0 / 227	
$wR(F^2$ all data)	$wR2 = 0.0896$	
$R(F$ obsd data)	$R1 = 0.0322$	
Goodness-of-fit on F^2	1.005	
Observed data [I > 2σ(I)]	5695	
Absolute structure parameter	0.29(13)	
Extinction coefficient	0.013(2)	
Largest and mean shift / s.u.	0.000 and 0.000	
Largest diff. peak and hole	0.357 and -0.192 e/$Å^3$	

$wR2 = \{ \Sigma[w(Fo^2 - Fc^2)^2] / \Sigma[w(Fo\ 2)2] \}^{1/2}$

$R1 = \Sigma||Fo| - |Fc|| / \Sigma|Fo|$

Table 2. Atomic coordinates and equivalent isotropic displacement parameters for KEVJAG-1-64. U(eq) is defined as one third of the trace of the orthogonalized U_{ij} tensor.

	x	y	z	U(eq)
F(1)	1.40527(15)	0.51268(6)	0.13182(4)	0.02518(17)
O(1)	0.83616(19)	0.32814(6)	0.26570(5)	0.0241(2)
O(2)	0.4676(2)	0.28816(6)	0.36053(5)	0.0253(2)
O(3)	0.22921(17)	0.40520(6)	0.38484(5)	0.01994(18)
N(1)	0.60123(18)	0.58733(6)	0.28431(5)	0.01392(17)
C(1)	1.2051(2)	0.53118(8)	0.16864(6)	0.0178(2)
C(2)	1.1310(2)	0.61959(8)	0.17376(6)	0.0179(2)
C(3)	0.9293(2)	0.63859(7)	0.21193(6)	0.0165(2)
C(4)	0.8050(2)	0.56957(7)	0.24507(5)	0.01350(18)
C(5)	0.4932(2)	0.52046(7)	0.31744(6)	0.01432(19)
C(6)	0.5600(2)	0.43207(7)	0.31470(6)	0.01496(19)
C(7)	0.7637(2)	0.40551(7)	0.27365(6)	0.0155(2)
C(8)	0.8851(2)	0.48092(7)	0.23940(6)	0.01425(19)
C(9)	1.0886(2)	0.46228(8)	0.20000(6)	0.0169(2)
C(10)	0.5137(2)	0.67913(7)	0.29476(6)	0.01584(19)
C(11)	0.6580(2)	0.72932(7)	0.34897(6)	0.0172(2)
C(12)	0.6691(2)	0.67848(7)	0.41471(6)	0.01520(19)
C(13)	0.4835(2)	0.68335(8)	0.46163(6)	0.0184(2)
C(14)	0.4897(2)	0.63222(9)	0.52050(7)	0.0221(2)
C(15)	0.6833(3)	0.57615(9)	0.53343(7)	0.0235(2)
C(16)	0.8705(2)	0.57144(9)	0.48726(7)	0.0236(2)
C(17)	0.8624(2)	0.62199(8)	0.42812(6)	0.0195(2)
C(18)	0.4205(2)	0.36674(7)	0.35465(6)	0.0171(2)
C(19)	0.0901(3)	0.34871(9)	0.42981(7)	0.0243(3)
C(20)	0.2063(3)	0.34486(9)	0.49878(7)	0.0278(3)

Table 3. Bond lengths [Å] and angles [°] for KEVJAG-1-64.

F(1)-C(1)	1.3605(14)	C(10)-H(10A)	0.9900
O(1)-C(7)	1.2377(13)	C(10)-H(10B)	0.9900
O(2)-C(18)	1.2120(14)	C(11)-C(12)	1.5078(16)
O(3)-C(18)	1.3505(15)	C(11)-H(11A)	0.9900
O(3)-C(19)	1.4519(15)	C(11)-H(11B)	0.9900
N(1)-C(5)	1.3398(14)	C(12)-C(13)	1.3915(16)
N(1)-C(4)	1.4005(14)	C(12)-C(17)	1.3952(16)
N(1)-C(10)	1.4738(13)	C(13)-C(14)	1.3940(18)
C(1)-C(9)	1.3680(17)	C(13)-H(13)	0.9500
C(1)-C(2)	1.3911(16)	C(14)-C(15)	1.3908(19)
C(2)-C(3)	1.3835(17)	C(14)-H(14)	0.9500
C(2)-H(2)	0.9500	C(15)-C(16)	1.3879(19)
C(3)-C(4)	1.4068(15)	C(15)-H(15)	0.9500
C(3)-H(3)	0.9500	C(16)-C(17)	1.3939(18)
C(4)-C(8)	1.4058(14)	C(16)-H(16)	0.9500
C(5)-C(6)	1.3768(14)	C(17)-H(17)	0.9500
C(5)-H(5)	0.9500	C(19)-C(20)	1.511(2)
C(6)-C(7)	1.4507(16)	C(19)-H(19A)	0.9900
C(6)-C(18)	1.4788(15)	C(19)-H(19B)	0.9900
C(7)-C(8)	1.4810(15)	C(20)-H(20A)	0.9800
C(8)-C(9)	1.4036(15)	C(20)-H(20B)	0.9800
C(9)-H(9)	0.9500	C(20)-H(20C)	0.9800
C(10)-C(11)	1.5365(16)		
C(18)-O(3)-C(19)	116.30(10)	C(6)-C(5)-H(5)	117.3
C(5)-N(1)-C(4)	119.53(9)	C(5)-C(6)-C(7)	119.82(10)
C(5)-N(1)-C(10)	118.74(9)	C(5)-C(6)-C(18)	118.27(10)
C(4)-N(1)-C(10)	121.53(9)	C(7)-C(6)-C(18)	121.90(10)
F(1)-C(1)-C(9)	118.53(11)	O(1)-C(7)-C(6)	125.68(11)
F(1)-C(1)-C(2)	118.43(11)	O(1)-C(7)-C(8)	120.49(11)
C(9)-C(1)-C(2)	123.03(11)	C(6)-C(7)-C(8)	113.83(9)
C(3)-C(2)-C(1)	118.46(11)	C(9)-C(8)-C(4)	119.27(10)
C(3)-C(2)-H(2)	120.8	C(9)-C(8)-C(7)	118.10(10)
C(1)-C(2)-H(2)	120.8	C(4)-C(8)-C(7)	122.63(10)
C(2)-C(3)-C(4)	120.19(10)	C(1)-C(9)-C(8)	118.98(11)
C(2)-C(3)-H(3)	119.9	C(1)-C(9)-H(9)	120.5
C(4)-C(3)-H(3)	119.9	C(8)-C(9)-H(9)	120.5
N(1)-C(4)-C(8)	118.77(9)	N(1)-C(10)-C(11)	112.43(9)
N(1)-C(4)-C(3)	121.17(10)	N(1)-C(10)-H(10A)	109.1
C(8)-C(4)-C(3)	120.06(10)	C(11)-C(10)-H(10A)	109.1
N(1)-C(5)-C(6)	125.35(11)	N(1)-C(10)-H(10B)	109.1
N(1)-C(5)-H(5)	117.3	C(11)-C(10)-H(10B)	109.1

H(10A)-C(10)-H(10B)	107.9	C(15)-C(16)-H(16)	120.0
C(12)-C(11)-C(10)	112.07(9)	C(17)-C(16)-H(16)	120.0
C(12)-C(11)-H(11A)	109.2	C(16)-C(17)-C(12)	120.93(11)
C(10)-C(11)-H(11A)	109.2	C(16)-C(17)-H(17)	119.5
C(12)-C(11)-H(11B)	109.2	C(12)-C(17)-H(17)	119.5
C(10)-C(11)-H(11B)	109.2	O(2)-C(18)-O(3)	122.91(11)
H(11A)-C(11)-H(11B)	107.9	O(2)-C(18)-C(6)	125.47(12)
C(13)-C(12)-C(17)	118.61(11)	O(3)-C(18)-C(6)	111.62(9)
C(13)-C(12)-C(11)	121.18(10)	O(3)-C(19)-C(20)	110.26(11)
C(17)-C(12)-C(11)	120.14(10)	O(3)-C(19)-H(19A)	109.6
C(12)-C(13)-C(14)	120.63(11)	C(20)-C(19)-H(19A)	109.6
C(12)-C(13)-H(13)	119.7	O(3)-C(19)-H(19B)	109.6
C(14)-C(13)-H(13)	119.7	C(20)-C(19)-H(19B)	109.6
C(15)-C(14)-C(13)	120.32(12)	H(19A)-C(19)-H(19B)	108.1
C(15)-C(14)-H(14)	119.8	C(19)-C(20)-H(20A)	109.5
C(13)-C(14)-H(14)	119.8	C(19)-C(20)-H(20B)	109.5
C(16)-C(15)-C(14)	119.50(12)	H(20A)-C(20)-H(20B)	109.5
C(16)-C(15)-H(15)	120.2	C(19)-C(20)-H(20C)	109.5
C(14)-C(15)-H(15)	120.2	H(20A)-C(20)-H(20C)	109.5
C(15)-C(16)-C(17)	120.00(12)	H(20B)-C(20)-H(20C)	109.5

Table 4. Anisotropic displacement parameters ($Å^2 x 10^3$) for KEVJAG-1-64. The anisotropic displacement factor exponent takes the form: $-2\pi^2[h^2 a^{*2} U_{11} + ... + 2 h k a^* b^* U_{12}]$

	U_{11}	U_{22}	U_{33}	U_{23}	U_{13}	U_{12}
F(1)	21(1)	30(1)	25(1)	-1(1)	8(1)	3(1)
O(1)	29(1)	10(1)	33(1)	-2(1)	5(1)	2(1)
O(2)	39(1)	12(1)	26(1)	2(1)	6(1)	-2(1)
O(3)	22(1)	18(1)	21(1)	3(1)	3(1)	-2(1)
N(1)	17(1)	9(1)	15(1)	1(1)	0(1)	1(1)
C(1)	17(1)	22(1)	15(1)	-2(1)	1(1)	1(1)
C(2)	21(1)	18(1)	15(1)	1(1)	2(1)	-2(1)
C(3)	21(1)	12(1)	16(1)	1(1)	1(1)	0(1)
C(4)	16(1)	11(1)	13(1)	-1(1)	-1(1)	0(1)
C(5)	17(1)	11(1)	15(1)	0(1)	0(1)	-1(1)
C(6)	19(1)	10(1)	16(1)	1(1)	-1(1)	-1(1)
C(7)	18(1)	11(1)	17(1)	-1(1)	-2(1)	0(1)
C(8)	17(1)	12(1)	14(1)	-1(1)	-2(1)	1(1)
C(9)	19(1)	16(1)	16(1)	-2(1)	0(1)	2(1)
C(10)	20(1)	10(1)	17(1)	0(1)	0(1)	3(1)
C(11)	22(1)	11(1)	18(1)	-1(1)	1(1)	-1(1)
C(12)	16(1)	13(1)	16(1)	-2(1)	0(1)	-1(1)
C(13)	17(1)	19(1)	19(1)	-2(1)	1(1)	2(1)
C(14)	20(1)	27(1)	19(1)	0(1)	3(1)	0(1)
C(15)	26(1)	24(1)	21(1)	4(1)	-2(1)	-1(1)
C(16)	22(1)	23(1)	26(1)	2(1)	-2(1)	6(1)
C(17)	17(1)	20(1)	22(1)	-1(1)	2(1)	3(1)
C(18)	23(1)	14(1)	15(1)	-1(1)	-1(1)	-4(1)
C(19)	31(1)	22(1)	20(1)	2(1)	4(1)	-9(1)
C(20)	42(1)	22(1)	20(1)	2(1)	2(1)	1(1)

Table 5. Hydrogen coordinates and isotropic displacement parameters for KEVJAG-1-64.

	x	y	z	U(eq)
H(2)	1.217003	0.665844	0.151551	0.021
H(3)	0.874620	0.698431	0.215784	0.020
H(5)	0.359545	0.535257	0.345089	0.017
H(9)	1.144612	0.402728	0.195190	0.020
H(10A)	0.342888	0.677085	0.308438	0.019
H(10B)	0.523916	0.712218	0.251518	0.019
H(11A)	0.823167	0.739584	0.332217	0.021
H(11B)	0.583082	0.788269	0.357059	0.021
H(13)	0.351176	0.721884	0.453463	0.022
H(14)	0.361095	0.635699	0.551942	0.026
H(15)	0.687320	0.541340	0.573572	0.028
H(16)	1.004075	0.533760	0.495958	0.028
H(17)	0.990365	0.617922	0.396513	0.023
H(19A)	0.079435	0.287785	0.410675	0.029
H(19B)	-0.074802	0.372690	0.434038	0.029
H(20A)	0.360889	0.313940	0.495409	0.042
H(20B)	0.101376	0.312490	0.530087	0.042
H(20C)	0.232162	0.405636	0.515577	0.042

Table 6. Torsion angles [°] for KEVJAG-1-64.

F(1)-C(1)-C(2)-C(3)	-179.16(10)	F(1)-C(1)-C(9)-C(8)	178.54(10)
C(9)-C(1)-C(2)-C(3)	-0.47(18)	C(2)-C(1)-C(9)-C(8)	-0.15(18)
C(1)-C(2)-C(3)-C(4)	0.58(17)	C(4)-C(8)-C(9)-C(1)	0.64(16)
C(5)-N(1)-C(4)-C(8)	2.49(15)	C(7)-C(8)-C(9)-C(1)	-179.20(10)
C(10)-N(1)-C(4)-C(8)	177.34(10)	C(5)-N(1)-C(10)-C(11)	95.41(12)
C(5)-N(1)-C(4)-C(3)	-177.22(10)	C(4)-N(1)-C(10)-C(11)	-79.48(12)
C(10)-N(1)-C(4)-C(3)	-2.37(15)	N(1)-C(10)-C(11)-C(12)	-53.66(13)
C(2)-C(3)-C(4)-N(1)	179.60(10)	C(10)-C(11)-C(12)-C(13)	-83.29(13)
C(2)-C(3)-C(4)-C(8)	-0.10(16)	C(10)-C(11)-C(12)-C(17)	93.64(13)
C(4)-N(1)-C(5)-C(6)	-2.56(17)	C(17)-C(12)-C(13)-C(14)	-0.55(18)
C(10)-N(1)-C(5)-C(6)	-177.56(11)	C(11)-C(12)-C(13)-C(14)	176.42(11)
N(1)-C(5)-C(6)-C(7)	0.36(18)	C(12)-C(13)-C(14)-C(15)	0.61(19)
N(1)-C(5)-C(6)-C(18)	179.98(10)	C(13)-C(14)-C(15)-C(16)	0.0(2)
C(5)-C(6)-C(7)-O(1)	-177.80(12)	C(14)-C(15)-C(16)-C(17)	-0.6(2)
C(18)-C(6)-C(7)-O(1)	2.59(18)	C(15)-C(16)-C(17)-C(12)	0.7(2)
C(5)-C(6)-C(7)-C(8)	1.65(15)	C(13)-C(12)-C(17)-C(16)	-0.10(18)
C(18)-C(6)-C(7)-C(8)	-177.95(10)	C(11)-C(12)-C(17)-C(16)	-177.11(11)
N(1)-C(4)-C(8)-C(9)	179.77(10)	C(19)-O(3)-C(18)-O(2)	4.70(17)
C(3)-C(4)-C(8)-C(9)	-0.52(16)	C(19)-O(3)-C(18)-C(6)	-175.23(10)
N(1)-C(4)-C(8)-C(7)	-0.40(16)	C(5)-C(6)-C(18)-O(2)	-175.00(12)
C(3)-C(4)-C(8)-C(7)	179.31(10)	C(7)-C(6)-C(18)-O(2)	4.61(19)
O(1)-C(7)-C(8)-C(9)	-2.30(17)	C(5)-C(6)-C(18)-O(3)	4.93(15)
C(6)-C(7)-C(8)-C(9)	178.22(10)	C(7)-C(6)-C(18)-O(3)	-175.46(10)
O(1)-C(7)-C(8)-C(4)	177.88(11)	C(18)-O(3)-C(19)-C(20)	82.56(14)
C(6)-C(7)-C(8)-C(4)	-1.61(15)		

Table 7. Hydrogen bonds for KEVJAG-1-64[Å and °].

D-H...A	d(D-H)	d(H...A)	d(D...A)	<(DHA)
C(2)-H(2)...O(2)#1	0.95	2.55	3.4406(16)	156.2
C(3)-H(3)...O(1)#1	0.95	2.55	3.1574(15)	121.9
C(10)-H(10B)...O(2)#2	0.99	2.49	3.4796(15)	176.3

Symmetry transformations used to generate equivalent atoms:
#1 -x+2, y+1/2, -z+1/2 #2 -x+1, y+1/2, -z+1/2

References

[1] Data Collection: APEX3 (2018) Bruker Inc., Madison, Wisconsin, USA.
[2] Data Reduction: SAINT (2016) Bruker Inc., Madison, Wisconsin, USA.
[3] Krause, L.; Herbst-Irmer, R.; Sheldrick, G. M.; Stalke, D. Comparison of silver and molybdenum microfocus X-ray sources for single-crystal structure determination *J. Appl. Cryst.* **2015**, *48*, 3–10. https://doi.org/10.1107/S1600576714022985
[4] Sheldrick, G. M. SHELXT– Integrated space-group and crystal-structure determination. *Acta Cryst.* **2015**, *A71*, 3–8. doi:10.1107/S2053273314026370
[5] Sheldrick, G. M. SHELXT – Integrated space-group and crystal-structure determination. *Acta Cryst.* **2015**, *C71*, 3–8. https://doi.org/10.1107/S2053273314026370
[6] Parsons, S.; H. D. Flack, H. D.; Wagner, T. Use of intensity quotients and differences in absolute structure refinement. *Acta Cryst.*, **2013**, *B69*, 249-259. https://doi.org/10.1107/S2052519213010014
[7] Hooft, R. W. W.; Straver, L. H.; Spek, A. L. Determination of absolute structure using Bayesian statistics on Bijvoet differences. *J. Appl. Cryst.* **2008**, *41*, 96-103. https://doi.org/10.1107/S0021889807059870
[8] Spek, A.L. *PLATON* SQUEEZE: a tool for the calculation of the disordered solvent contribution to the calculated structure factors. Acta Cryst. **2015**, *C71*, 9-19. https://doi.org/10.1107/S2053229614024929
